# Phylogenetic-Derived Insights into the Evolution of Sialylation in Eukaryotes: Comprehensive Analysis of Vertebrate β-Galactoside α2,3/6-Sialyltransferases (ST3Gal and ST6Gal)

**DOI:** 10.3390/ijms17081286

**Published:** 2016-08-09

**Authors:** Roxana E. Teppa, Daniel Petit, Olga Plechakova, Virginie Cogez, Anne Harduin-Lepers

**Affiliations:** 1 Bioinformatics Unit, Fundación Instituto Leloir, Av. Patricias Argentinas 435, C1405BWE Buenos Aires, Argentina; elinteppa@gmail.com; 2 Laboratoire de Génétique Moléculaire Animale, UMR 1061 INRA, Université de Limoges Faculté des Sciences et Techniques, 123 avenue Albert Thomas, 87060 Limoges, France; daniel.petit@unilim.fr; 3 FRABio-FR3688 CNRS, Univ. Lille, bât. C9, 59655 Villeneuve d’Ascq cedex, France; olga.plechakova@univ-lille1.fr; 4 Univ. Lille, CNRS, UMR 8576-UGSF-Unité de Glycobiologie Structurale et Fonctionnelle, 59000 Lille, France; virginie.cogez@univ-lille1.fr; 5 UGSF, Bât. C9, Université de Lille-Sciences et Technologies, 59655 Villeneuve d’Ascq, France

**Keywords:** evolution, sialyltransferases, sialic acid, molecular phylogeny, functional genomics

## Abstract

Cell surface of eukaryotic cells is covered with a wide variety of sialylated molecules involved in diverse biological processes and taking part in cell–cell interactions. Although the physiological relevance of these sialylated glycoconjugates in vertebrates begins to be deciphered, the origin and evolution of the genetic machinery implicated in their biosynthetic pathway are poorly understood. Among the variety of actors involved in the sialylation machinery, sialyltransferases are key enzymes for the biosynthesis of sialylated molecules. This review focus on β-galactoside α2,3/6-sialyltransferases belonging to the ST3Gal and ST6Gal families. We propose here an outline of the evolutionary history of these two major ST families. Comparative genomics, molecular phylogeny and structural bioinformatics provided insights into the functional innovations in sialic acid metabolism and enabled to explore how ST-gene function evolved in vertebrates.

## 1. Introduction

**Figure 1:**
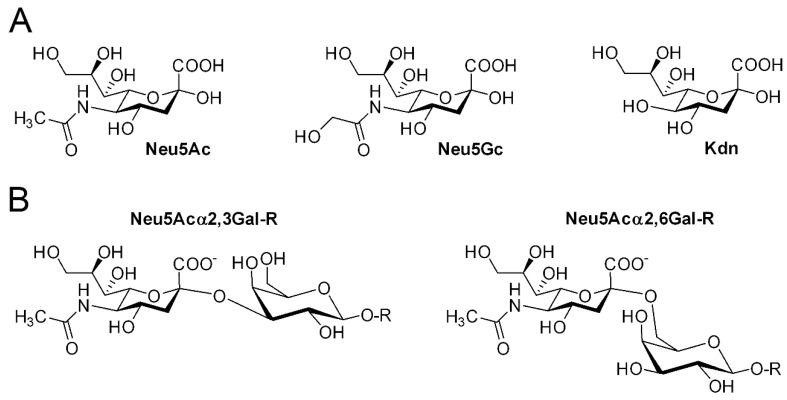
Sialic acids and sialylated molecules. (**A**) *N*-acetylneuraminic acid (Neu5Ac) is the major sialic acid molecule found in human tissues. Other commonly described sialic acids in vertebrates are *N*-glycolylneuraminic acid (Neu5Gc) and 2-keto-3-deoxy-nonulosonic acid (Kdn); (**B**) In vertebrates, the sialic acid hydroxyl group at position 2 is most frequently glycosidically-linked to either the 3- or 6-hydroxyl group of galactose (Gal) residues. These glycosidic linkages are formed by the β-galactoside α2,3/6-sialyltransferases described in this review.

Sialic acids (SA) represent a broad family of nine-carbon electro-negatively charged monosaccharides commonly described in the deuterostomes and some microorganisms [1,2,3,4,5]. Interestingly, SA show a discontinuous distribution across evolutionary metazoan lineages. Outside the deuterostome lineage (vertebrates, urochordates, echinoderms), SA are rarely described in some ecdysozoa and lophotrochozoa protostomes like in the *Drosophila melanogaster* nervous system during embryogenesis [6,7,8,9] or in larvae of the cicada *Philaenus spumarius* [10], or on glycolipids of the common squid and pacific octopus [11]. They are notably absent from plants, archaebacteria or the ecdysozoan *Caenorhabditis elegans* [12]. SA exhibit a huge structural diversity and species specific modifications. This family of compounds encompasses *N*-acetylneuraminic acid (Neu5Ac) and over 50 derivatives showing various substituents on carbon 4, 5, 7, 8 or 9, like Neu5Gc and Kdn, with Neu5Ac being the most prominent SA found in higher vertebrates (Figure 1A). In vertebrates, the SA hydroxyl group at position 2 is most frequently glycosidically-linked to either the 3- or 6-hydroxyl group of galactose (Gal) residues (Figure 1B) or the 6-hydroxyl group of *N*-acetylgalactosamine (GalNAc) residues and can form to a lesser extent di-, oligo- or poly-SA chains via their 8-hydroxyl group. In deuterostome lineages, sialoglycans are found in cellular secretions and on the outer cell surface, essentially as terminal residues of the glycan chains of glycoproteins and glycolipids [13,14] constituting the so-called siaLome [15], which varies according to animal species.

Owing to their anionic charge and their peripheral position in glycans, SA play major roles in the various vertebrate biological systems ranging from protecting proteins from proteolysis, modulating cell functions to regulating intracellular communication [16]. For instance, the α2,3-linked SA contribute to the high viscosity of the mucin-type *O*-glycosylproteins found on the intestine endothelia or on the surface of fish or frog eggs [17]. Besides, some endogenous proteins specifically recognize sialylated molecules at the cell surface that act as receptors. Examples include selectin on endothelial cells mediating leucocytes and platelets trafficking, and siglecs playing a role in immune cell regulation [18,19]. Likewise, a number of pathogenic agents like toxins (cholera toxin), protozoa (Plasmodium), viruses (influenza virus), bacteria (*Helicobacter pylori*) use cell surface SA as ligands for cell adhesion [20] and have evolved this ability to distinguish a specific sialylated sugar code [21,22] distinguishing α2,3- or α2,6-linked SA in vertebrate tissues [23]. One of the most notable examples is the flu virus tropism: human strains of influenza A virus bind selectively to SA*α*2,6-Gal epitopes that prevail in the human tracheal mucosal epithelium, whereas chimpanzee strains bind selectively to SA*α*2,3-Gal epitopes primarily expressed in their tracheal mucosal epithelium [24,25,26], suggesting that the switch to *α*2,6-linked SA could give the human ancestor some resistance towards influenza viruses, which later on could have evolved and adapted to the modern humans.

**Figure 2:**
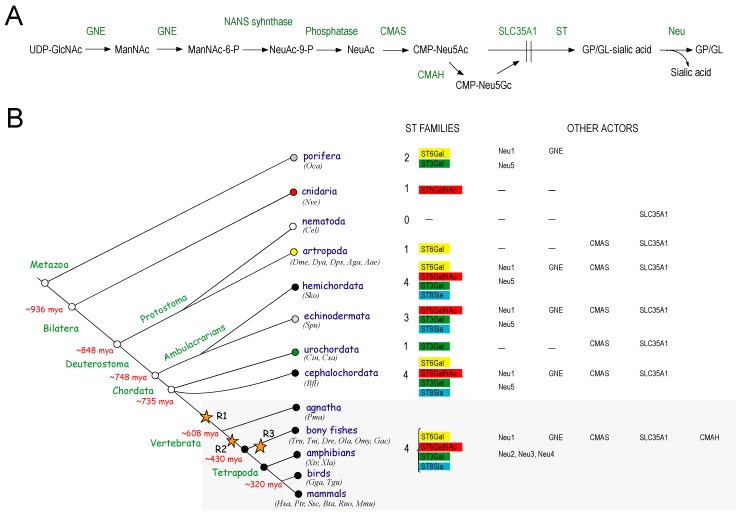
Evolution of the biosynthetic pathway of sialic acids in Metazoa. (**A**) Schematic representation of the vertebrate biosynthetic pathway of sialylated molecules. Key enzymes implicated in the biosynthetic pathway of sialic acids are indicated as follows: GNE: UDP-GlcNAc2epimerase/ManNAc kinase; NANS: Neu5Ac9-phosphate synthase; CMAS: CMP-Neu5Ac synthase: CMAH: CMP-Neu5Ac hydroxylase; SLC35A1: CMP-Neu5Ac transporter; ST: sialyltransferases, Neu: neuraminidase; (**B**) Illustration of the evolutionary history of the sialic acid biosynthetic pathway in the metazoans. Evidences of the occurrence of the biosynthetic pathway of sialylated molecules across the metazoans have been obtained based on BLAST search analysis of the various actors in genomic databases. Yellow stars indicate the two whole genome duplication events (WGD R1–R2) that took place at the base of vertebrates and the teleostean whole genome duplication event (WGD R3) that occurred in the stem of bony fish.

The SA metabolism is complex and requires a large panel of enzymes with various subcellular localization including the nuclear CMP-Neu5Ac synthase (CMAS), the cytosolic UDP-GlcNAc 2-epimerase/*N*-acetylmannosamine kinase (GNE), the cytosolic cytidine monophosphate-*N*-acetylneuraminic acid hydroxylase (CMAH), the Golgi CMP-Neu5Ac transporter (SLC35A1), the Golgi sialyltransferases (ST) and sialidases (Neu) (Figure 2A) [27]. The distribution of SA in the metazoans further suggests that this sialylation machinery has evolved at least in the last common ancestor (LCA) of the metazoans, well before the divergence of protostomes (Ecdysozoa and Lophotrochozoa) and deuterostomes. Very little is known pertaining to the evolutionary history of each orthologous gene. However, these genes show also an unusual and patchy phylogenetic distribution with a huge gene families’ expansion observed in the deuterostome lineages indicative of the prominent role of sialoglycoconjugates in the deuterostome ancestor [28,29,30,31] and selective loss in most non-deuterostome lineages as well as in some vertebrate lineages (e.g., *CMAH* gene). The humans cannot synthesize CMP-Neu5Gc from CMP-Neu5Ac because the human *CMAH* gene was inactivated 2 million years ago [32,33], an activity that was independently lost in the ferrets [34], birds and reptiles [35] (Figure 2B)**.** Interestingly, a *cmas* gene was identified and characterized in the *D. melanogaster* genome [36,37] and moreover, *1 gne*, *2 st* and *2 neu* genes were identified in the porifera *Oscarella carmella* [28,29,38,39], and a *SLC35A1*-related gene was identified in the tunicate *Ciona intestinalis* and *C. elegans* genomes (personal data) suggesting the ancient occurrence and subsequent divergent evolution of the sialylation machinery.

The structural diversity of sialylated glycoconjugates is further ensured by a diverse set of STs consisting of 20 members described in the human tissues [40,41]. The STs reside and are strictly organized in the *trans*-Golgi network of eukaryotic cells as type II transmembrane proteins with a similar topology showing a short *N*-terminal cytoplasmic tail, a single transmembrane domain, a stem domain and a large C-terminal catalytic domain oriented in the Golgi lumen [42]. The STs use CMP-β-Neu5Ac, CMP-β-Neu5Gc or CMP-β-Kdn as activated sugar donors for the sialylation at terminal positions of oligosaccharide chains of glycoconjugates. These STs are categorized into 4 families (ST6Gal, ST3Gal, ST6GalNAc and ST8Sia) [41,43] found in the GT-29 of the Carbohydrate-Active enZYme (CAZy) database [44] and named according to the glycosidic linkage formed and the monosaccharide acceptor [45]. Each family catalyzes the formation of different glycosidic linkages, α2–3, or α2–6 to the terminal Gal residue in *N*- or *O*-glycans, α2–6 to the terminal GalNAc residue in *O*-glycans and glycolipids and α2–8 to terminal SA residues in *N*- or *O*-glycans or glycolipids). The ST enzymatic activities have been documented mainly in mouse and human tissues and more recently in chicken [46,47], and to a lesser extent in the invertebrates like the fly *D. melanogaster* [48], the silkworm *Bombyx mori* [49], the amphioxus *Branchiostoma floridae* [1] and the tunicate *C. intestinalis* [5]. Each member of the mammalian ST3Gal and ST6Gal families shows exquisite acceptor specificities (for reviews, see [41,50,51]. However, as most of the STs have not been experimentally characterized, it remains unclear how these diverse biochemical functions evolved and what were the biological consequences of the functional diversification of STs.

In the post-genomic era, a major biological question remains to elucidate the multi-level protein function of STs (i.e., biochemical, cellular or developmental functions) which can be achieved through the simultaneous study of different levels of biological organization and the use of computational means. The most represented vertebrate β-galactoside α2,3/6-sialyltransferases (ST3Gal and ST6Gal) offer the unique opportunity to understand deuterostome innovations and the STs functional evolution. The ST3Gal and ST6Gal are well studied enzymes catalyzing the transfer of sialic acid residues to the terminal galactose residues of either the type-I, type-II or type-III disaccharides (Galβ1,3GlcNAc; Galβ1,4GlcNAc or Galβ1,3GalNAc, respectively) resulting in the formation of α2–3 or α2–6 glycosidic linkages on terminal galactose (Gal) residues. In previous reports, we deciphered key genetic events, which led to the various ST3Gal and ST6Gal subfamilies described in the vertebrates, we established the evolutionary relationships of newly described STs and provided insights into the structure-function relationships of STs [39,52] and into their various biological functions [38,53]. Focusing on β-galactoside α2,3/6-sialyltransferases (ST3Gal and ST6Gal), we explore in this review the molecular evolution of β-galactoside α2,3/6 sialyltransferases with the goal of bringing an evolutionary perspective to the study of SA-based interactions and contributing a powerful approach for a better understanding of sialophenotype in vertebrates.

## 2. Genome-Wide Search of STs Genes Decline or Expansion?

A general strategy using conventional BLAST search approaches [54] was adopted for homologous ST sequences identification in the transcriptomic and genomic databases like NCBI or ENSEMBL to reconstruct the animal ST genes repertoire and assign orthologies [39]. Although the vertebrate ST amino acid sequences show very limited overall sequence identity (around 20%), conserved peptide motifs have been described within their catalytic domain, which are very useful hallmark for ST identification. Different sets of protein regions considering three levels of amino acid sequence conservation have been described in the past that are retrieved from multiple sequence alignments (MSA) of (1) all animal ST called sialylmotifs L (large), S (small), III and VS (very small); (2) each family of ST, called family motifs a, b, c, d and e; (3) in each vertebrate subfamily [55].

This strategy led to the identification of a total number of 750 *st3gal*- and *st6gal*-related sequences in the genome of 127 metazoan species that represent a significant sampling of metazoan diversity illustrated in Figure 2B. The *st6gal* and *st3gal* gene families show a broad phylogenetic distribution in Metazoan from sponges to mammals. The mRNA fragments identified from the Homosclerophore sponge *O. carmella* in the Porifera phylum suggested that ancestral *st6gal1/2* and *st3gal1/2/8* genes were already present in the earliest metazoans [38,53] and could represent the most ancient ST described in animals. This observation pointed also the early divergence of *st3gal* groups GR1 (*st3gal1/2/8*), GR2 (*st3gal4/6/9*), GR3 (*st3gal3/3-r/5/7*) and GRx (*st3gal4/6/9/3/3-r/5/7*) that far predates the divergence of protostomes and deuterostomes [53]. Interestingly, ST-related sequences possessing a conserved GT-29 protein domain Pfam00777 with sialylmotifs L, S, III and VS and no family motif could be identified in plants, in the green marine microalga *Bathycoccus prasinos* [56], in the haptophyte *Emiliana huxleyi* (XM_005778044) [57], in the cryptophyte alga *Guillarda theta* and in the red tide dinoflagelate *Alexandrium minutum* [29] suggesting the presence of an ancestral protist ST gene set. However, the evolutionary relationships of these more distantly related ST sequences are not yet clearly established and the origin of ST-related sequences in Metazoan remains enigmatic [13,31,43]. Since no ST-related sequence was identified in Choanoflagellates, the closest known relatives of metazoans [58], nor in fungi, it can be deduced the ancient origin of ST sequences and their subsequent disappearance in some metazoan branches like in the Nematoda *C. elegans* for both ST families, in protostome for the *st3gal* family, and echinoderms and tunicates for the *st6gal* family [38,53].

A large data set of β-galactoside α2,3/6-sialyltransferase related sequences was identified in vertebrate genomes and orthologs of the 8 known mammalian β-galactoside α2,3/6-sialyltransferase genes could be identified in fish and amphibian genomes with the notable exception of the *st3gal6* gene that disappeared from fish genome (Table 1). An important indication of innovation was obtained in the genome of vertebrates suggesting the occurrence of as-yet not described ST-related homologs in fish and tetrapods. The ST sequence identification in vertebrate genomes with key phylogenetic position like the sea lamprey *Petromyzon marinus* [59] at the stem of vertebrates or the spotted gar *Lepisosteus oculatus* at the base of teleosts [60] was helpful to propose an evolutionary scenario. These novel ST-related sequences could originate from gene duplication events like the two whole-genome duplications (WGD R1–R2) that occurred deep in the ancestry of the vertebrate lineage (2R hypothesis) [61].

## 3. Molecular Phylogeny of β-Galactoside α2,3/6-Sialyltransferases

**Figure 3:**
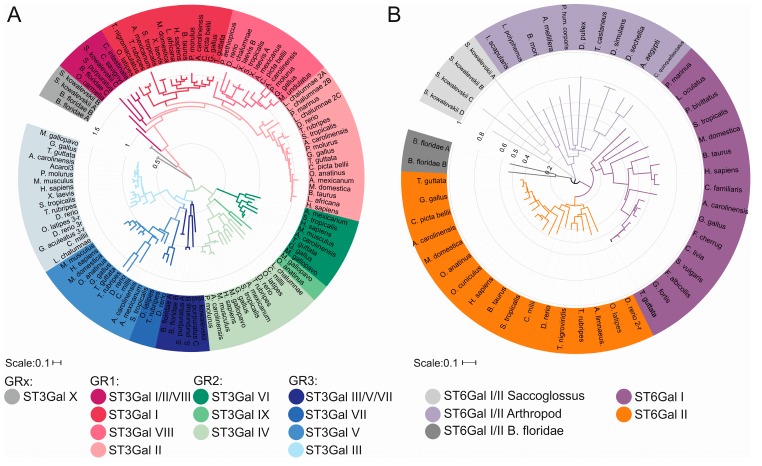
Maximum Likelihood phylogenetic trees of protein sequences of β-galactoside α2,3/6-sialyltransferases (ST3Gal and ST6Gal). In both cases, the phylogenetic trees were inferred using the Maximum Likelihood method based on the Whelan and Goldman method with options G (gamma distribution) and I (Invariant sites present). Alignments were performed using Clustal Omega, available at the site http://www.ebi.ac.UK/Tools/msa/clustalo/ (Data S1). Evolutionary analyses were conducted in MEGA6. The trees were re-drawn using iTOL 3.2 [63] available at the URL http://itol.embl.de/. (**A**) ST3Gal tree was obtained from 124 sequences and 228 positions in the final data set; (**B**) ST6Gal tree was inferred from 50 sequences and 256 positions and 50 sequences in the final data set.

Molecular phylogeny, with the construction of phylogenetic trees has been used to get further insight into the orthology and structure/function relationships of the identified β-galactoside α2,3/6-sialyltransferase sequences. As a first step, multiple sequence alignments (MSA) using predicted protein sequences, clustal Omega or MUSCLE algorithms evidenced several informative amino acid sites in the catalytic domain of ST to construct phylogenetic trees [39]. Among these conserved motifs, sialylmotifs and family motifs detection helped establishing the global evolutionary relationships between the identified ST sequences and enabled sequence-based prediction of their molecular function. Phylogeny of β-galactoside α2,3/6-sialyltransferase sequences was reconstructed using various methods implemented in the Molecular Evolutionary Genetics Analysis (MEGA) software and the reliability of the branching pattern was assessed by the bootstrap method [39,62]. The topology of the trees indicated that the ST6Gal and ST3Gal sequences identified in invertebrates are orthologous to the common ancestor of vertebrate subfamily members as they branch out from the tree before the split into vertebrate ST subfamilies with the exception of the ST3Gal members of the GRx group, which disappeared during vertebrate evolution [38,53]. The molecular phylogeny of β-galactoside α2,3/6-sialyltransferases is displayed in Figure 3 using iTOL [63].

## 4. When β-Galactoside α2,3/6-Sialyltransferase Evolutionary Studies Meet Genome Reconstruction

Gene organization and gene localization studies were used to assign the newly described *st3gal* and *st6gal* orthologs and to reconstruct the genetic events that have led to ST functional diversification in vertebrates.

At the gene level, β-galactoside α2,3/6-sialyltransferase genes are polyexonic with an overall conserved exon/intron organization in each family from fish to mammals, which support the model of the common ancestral origin of each family (ST3Gal and ST6Gal) [55]. Interestingly, analysis of exon/intron organization and composition in the *st6gal* gene family showed that the *st6gal1* genes encoded by frogs and fish have independently undergone different insertion events inside the first exon. These genetic events have led to an extended stem region of fish and frog ST6Gal I with potential impact on their enzyme activities [38].

It is speculated that during metazoan evolution, β-galactoside α2,3/6-sialyltransferase genes were subject to several duplication events affecting single genes or chromosomes or whole genomes. As far as the *st3gal* genes are concerned, a first series of tandem duplication of an ancestral *st3gal* gene in proto-Metazoa stem led to the GR1 and GR2/GR3/GRx groups of α2,3-sialyltransferases before the Porifera emergence. As previously reported for α2,8-sialyltransferases [30], a second series of tandem duplication took place after the Porifera radiation that gave rise to the full diversity of α2,3-sialyltransferase groups, as confirmed using ancestral genome reconstruction data from Putnam et al. [53,64]. This further indicates that the functional diversity of *st3gal* groups was acquired well before vertebrate divergence. In addition, gene copy number variants (CNV) were described within various animal genomes that might have contributed to evolutionary novelties, although not much is known about the functional impact of CNVs [65]. For instance, 2 and 3 copies of the ancestral *st6gal1/2* gene were identified in the amphioxus (*B. floridae*) and in the sea lamprey (*P. marinus*) genome, respectively. Similarly, 4 copies of the *st3gal1* gene named *st3gal1A*, *st3gal1B*, *st3gal1C* and *st3gal1D*, which are not shared among other fish species could be identified in close chromosomal location in the zebrafish genome.

**Figure 4:**
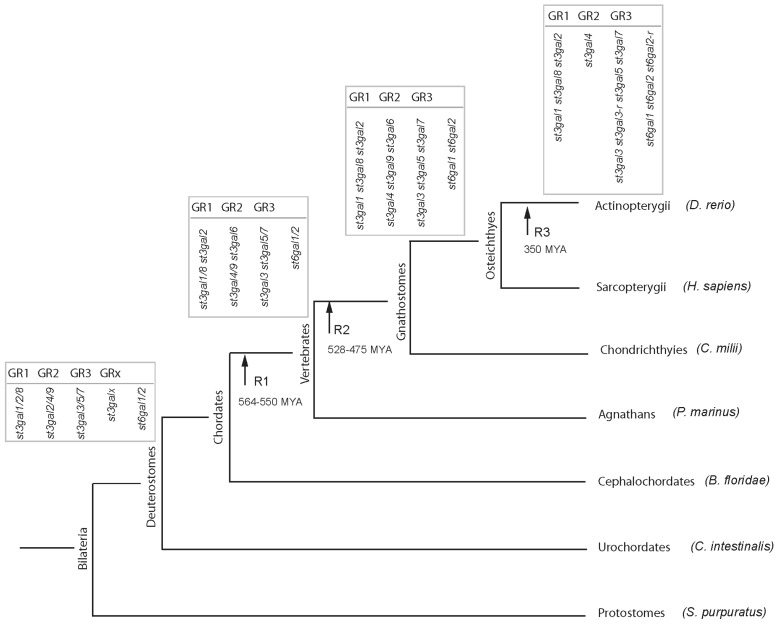
Hypothetical scenario of the evolutionary history of β-galactoside α2,3/6-sialyltransferase genes in the WGR context. This drawing illustrates the proposed evolutionary scenario of *st3gal* and *st6gal* genes drawn in line with the 2R hypothesis [61]. The arrows indicate the two vertebrate whole genome duplication events (WGD-R1: ~555 MYA and WGD-R2: ~500 MYA) and the teleosts specific whole genome duplication events (WGD-R3: ~350 MYA). A single *st3gal1/2/8* (GR1), *st3gal3/5/7* (GR3), *st3gal4/6/9* (GR2) and *st6gal1/2* gene in the stem bilaterian was duplicated twice before and after the emergence of agnathans, raising 11 *st3gal* and *st6gal* subfamilies at the base of gnathostomes. 3 *st3gal* gene subfamilies (*st3gal7*, *st3gal8* and *st3gal9*) were further lost in the mammalian lineage. In Actinopterygii, after WGD-R3 the two duplicated genes *st3gal3*-*r* and *st6gal2*-*r* are maintained in the zebrafish genome, whereas the *st3gal6* and *st3gal9* genes are secondarily lost. *S. purpuratus* = *Strongylocentrotus purpuratus*; *C. intestinalis* = *Ciona intestinalis*; *B. floridae* = *Branchiostoma floridae*; *P. marinus* = *Petromyzon marinus*; *C. milii* = *Calorhinchus milii*; *D. rerio* = *Danio rerio*.

In the vertebrate genomes, detection of conserved synteny (i.e., set of orthologous genes born by a chromosomal segment in different genomes) and of large sets of paralogons (i.e., pair of chromosomes bearing a set of paralogous genes in a given genome resulting from WGD) provided strong evidence of the 2 rounds of WGD, which likely occurred about 500 and 555 million years ago (MYA). These large scale genetic events generated a class of paralogs known as ohnologs [66] and the various *st3gal* and *st6gal* gene subfamilies described in Table 1. Interestingly, 5 out of the 16 β-galactoside α2,3/6-sialyltransferase genes subfamilies generated after the 2 WGD events were immediately lost in the early vertebrate genome, while 4 other *st3gal* subfamilies were independently lost later on, in various vertebrate genomes like *st3gal6* in teleosts or *st3gal7* in tetrapods and *st3gal8* in mammals (Table 1). Similarly, almost all the ST duplicates generated after the teleost specific third WGD event at the base of Actinopterygii were lost with the exception of *st6gal2*-*r* and *st3gal3*-*r* genes conserved in the zebrafish genome. Finally, chromosomal localization and genome reconstruction studies [67] of the ST gene loci in the various vertebrate genomes indicated several major chromosomal rearrangement and translocations of ST genes like *ST3GAL5* in the human genome or *st6gal1* in the zebrafish genome, which likely have undergone chromosomal translocation from Hsa2 to Hsa4 and from Dre15 to Dre21, respectively [38,53] (Figure 4).

To account for ST gene novelties found in vertebrates, a refined nomenclature was proposed in Petit et al [39] based on the gene symbols and names assigned by the HUGO Gene Nomenclature Committee (HGNC; http://www.genenames.org/cgi-bin/genefamilies/set/438) and the ST nomenclature initially established by Tsuji et al. [45]. As described above, the vertebrate genomes contain numerous ST-related genes that result from various duplication events. The newly identified ST subfamilies were named according to their phylogenetic relationship with previously described ST subfamilies as follows: (1) A genome-wide duplication event known as WGD-R3 took place in the ray fin fish lineage leading to two copies of a gene that is otherwise found as a single copy in tetrapods. The symbols used for these specific fish duplicated genes are identical to those used for the mouse ST orthologs followed by “-r” meaning “-related” (Table 1); (2) Genes resulting from lineage-specific small scale duplications are named according to the mouse ST orthologs symbol followed by A, B, C, D (Table 1); (3) Finally, duplicates that resulted from whole genome duplication events WGD-R1 and R2 before the emergence of the teleosts branch are given a new ST subfamily number and no additional suffix is attributed. For instance, see in Table 1 the newly described vertebrate *st3gal7*, *st3gal8* and *st3gal9* gene subfamilies. The invertebrate ST genes are orthologous to the common ancestor of the vertebrate subfamilies and are named accordingly. For instance, the *D. melanogaster st6gal1/2* gene (also known as DSiaT) described in [48] and the *C. intestinalis st3gal1/2* gene [5].

## 5. Conservation versus Changes in the β-Galactoside α2,3/6-Sialyltransferase Sequences

**Figure 5:**
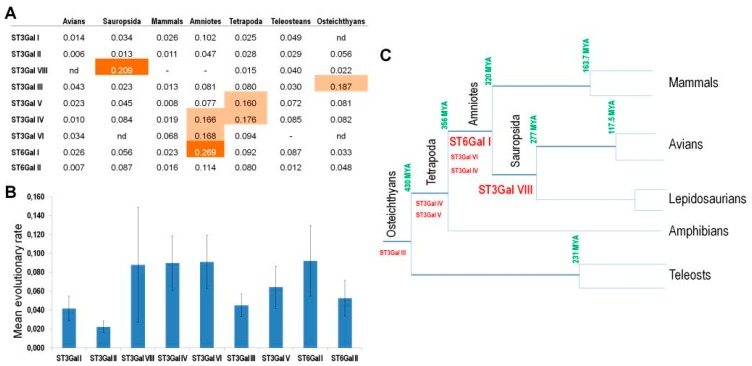
Evolutionary rates of β-galactoside α2,3/6-sialyltransferase subfamilies in Vertebrates. (**A**) Trees obtained from Minimum Evolution and JTT model implemented in MEGA 6.0 [69] using 91 ST3Gal and 27 ST6Gal sequences allowed calculation of the evolutionary rates of β-galactoside α2,3/6-sialyltransferase subfamilies for the major divisions in Vertebrates (Data S2). For Amniotes, 2 or 3 sequences were taken, including at least Man and a Marsupial in Mammals, Chicken and Ostrich in Avians, and Caroline Anole and Burmese Python in Lepidosaurians. Orange background denotes the highest values; (**B**) Mean evolutionary rates in the different β-galactoside α2,3/6-sialyltransferase subfamilies (±standard error). The mean evolutionary rates of ST3Gal and ST6Gal subfamilies were calculated from Teleosteans to Amniotes. The standard errors show variations in the different subfamilies, from the highest in ST3Gal VIII and ST6Gal I, to the lowest in ST3Gal II, ST3Gal I, ST3Gal III and ST6Gal II; (**C**) Highest evolution rates of β-galactoside α2,3/6-sialyltransferase in vertebrate evolutionary tree. They correspond to the cases where an elevated value is observed in one or two lineages. During the differentiation of Amniotes, we record three subfamilies particularly evolving their catalytic sequences, ST6Gal I and at a lesser extent ST3Gal III and ST3Gal IV. In the lineage of Tetrapods, ST3Gal IV and ST3Gal V present high evolutionary rates. In the ancestors of birds and Lepidosaurians (snakes and lizards, i.e., Sauropsides), there is only one subfamily where numerous changes occur in the catalytic domain, the ST3Gal VIII, as mentioned later on.

Even though a phylogenetic tree might not be adequate to reflect relatedness between all sequences and may not provide sufficient resolution, the branch lengths are indicative of the sequence changes. To deduce the evolutionary rates, the branch lengths have to be divided by the elapsed corresponding time, calculated from the calibrations available in Hedges et al. [68]. As illustrated in Figure 5, ST3Gal I, ST3Gal II, ST3Gal III and ST6Gal II have the most conserved sequences across the vertebrate lineages, whereas ST6Gal I and to a lesser extent ST3Gal VI and ST3Gal IV show a particularly high evolutionary rate in their catalytic domain during Amniotes differentiation [38,53].

**Figure 6:**
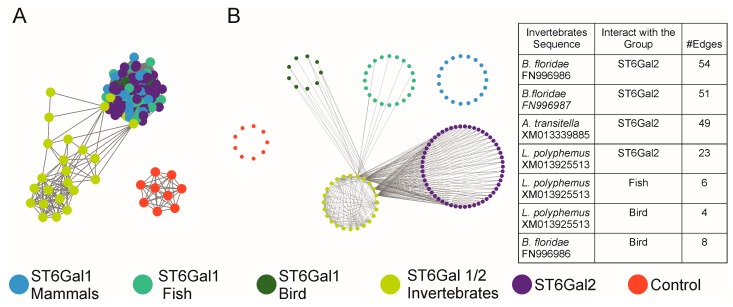
Sequence similarity network of ST6Gal sequences. The Figure represents ST sequences as nodes (circles) and all pairwise sequence relationships (alignments) better than a BLAST *E*-value threshold of 1E-83 as edges (lines). The network is composed by 126 ST6Gal sequences and 9 ST3Gal sequences as control group (red circles). (**A**) The network is visualized using a Force Direct layout, where the length of the edges is inversely proportional to the sequence similarity. Sequences belonging to Invertebrates form a separate group from all ST6Gal sequences, pointing out the dissimilarity with ST6Gal I and ST6Gal II sequences. To better visualize the relationships of invertebrates sequences, we show only the edges that involve the Invertebrate sequences; In panel (**B**) sequences are clustered by groups without using edges information (the edges are not proportional to sequence similarity). The network shows that seven invertebrate sequences are related with sequences of ST6Gal2 and ST6Gal1 of the Bird and Fish groups. The names of the invertebrate sequences related to other groups and the number of edges are shown in the table. It is important to note that the Invertebrate sequences do not show relationships with the mammalian ST6Gal1 sequences at this threshold.

It is useful to substantiate the proximity/divergence of sequences between the different subfamilies using other approaches like similarity network. Orthology inference and evolutionary relationships were analyzed using protein sequences and the approach of similarity network visualization in which the nodes represent proteins and the edges indicate similarity in amino acid sequence [70]. The generated network can be visualized in Cytoscape [71]. The similarity network of a larger set of β-galactoside α2,3/6-sialyltransferase protein sequences demonstrated a high degree of similarity between ST3Gal sequences belonging to the GR1 group (ST3Gal I/II/VIII) with the notable exception of the fish ST3Gal sequences and a lower degree of similarity for the sequences belonging to the GR2 and GR3 groups [53]. Similar analysis conducted for ST6Gal sequences illustrated in Figure 6 highlighted a higher degree of similarity between the invertebrate ST6Gal I/II and vertebrate ST6Gal II sequences and pointed to a stronger conservation of ST6Gal II sequence at a stringent threshold (*E*-value).

## 6. Fate of Vertebrate Duplicated ST Genes

**Figure 7:**
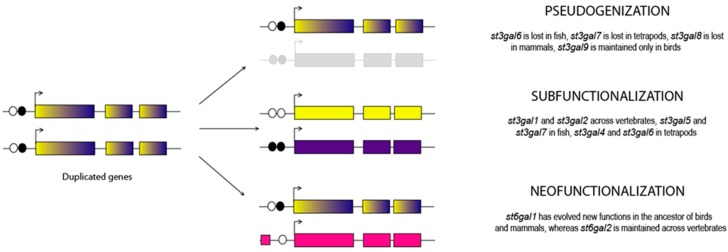
Evolutionary fate of ST gene duplicates after WGD events. On the left side, schematic representation of ancestral polyexonic ST genes duplicates (exons are represented by colored boxes and genomic regulatory elements are represented by black and white circles). On the right side, the three major evolutionary fates of the various newly created ST gene subfamilies are indicated (1) pseudogenization: gene loss; (2) subfunctionalization: coding sequences and regulatory elements evolve and are partitioned according to specific molecular functions; (3) neofunctionalization: one of the newly duplicated gene accumulates mutations in its coding region and/or in its regulatory genomic elements giving rise to new molecular function.

After a gene duplication event, the two paralogous genes are identical. Non-functionalization and loss of one of the duplicates by accumulation of deleterious mutations is the most frequent outcome [66,72] while the parental gene is maintained active (Figure 7). As mentioned previously, 5 out of the 16 β-galactoside α2,3/6-sialyltransferase genes subfamilies generated after the 2 WGD events that took place at the root of the vertebrate lineage were immediately lost in the early vertebrate genome. Similarly, only 2 duplicated ST genes, namely *st3gal3*-*r* and *st6gal2*-*r* were maintained in the ray-finned fish genome after the teleost-specific round of WGD that occurred about 350 MYA. A pseudogenization process can occur at larger evolutionary scales by the accumulation of loss-of-function mutations in previously established genes and might also influence the fate of the surviving paralogs [73,74]. Interestingly, the inactivation of 4 *st3gal* subfamilies occurred independently, in various vertebrate genomes like *st3gal6* in teleosts or *st3gal7* in tetrapods and *st3gal8* in mammals, while *st3gal9* was maintained mainly in birds. Substitution rate analysis in each *st3gal* gene subfamily indicated a weaker selective pressure on the *st3gal7*, *st3gal8* and *st3gal9* genes and acquisition of mutations that compromised their function in mammals [53]. Indeed, several *st3gal* pseudogenes could be identified in the human genome that likely result from pseudogenization of a once active gene like *ST3GAL8P* on human chromosome 20 (ENSG00000242507). It is suggested that inactivation of the *st3gal8* gene in the mammalian ancestor became possible after alternative or more beneficial glycosyltransferase activity evolved in the stem lineage of mammals, which could have resulted in major adaptive changes in SA metabolism.

As illustrated in Figure 7, the function of the duplicated genes may diverge either because one or both evolved new function (neofunctionalization) [75] or because both duplicates partition the ancestral gene function (subfunctionalization) and several models have been proposed [76]. To understand the evolutionary forces that have influenced the ST gene number and their functional fate, the expression profile of the various β-galactoside α2,3/6-sialyltransferase genes was studied across vertebrates. As a first step, screening of various tissue EST libraries and statistical analysis using principal component analysis (PCA) of the expression profile accessible from the Unigene data base were undertaken [39]. The data pointed to a wider expression of *st3gal* and *st6gal1* genes in mammals and birds, whereas teleost and amphibian *st6gal1* genes showed a restricted profile of expression comparable to the one of *st6gal2* genes suggesting a change in the expression profile of *st6gal1* genes in amniotes. The expression pattern of the various β-galactoside α2,3/6-sialyltransferase genes analyzed by means of RT-PCR in adult vertebrate tissues or using whole mount in situ hybridization (ISH) in the developing zebrafish embryo and comparative genomics approaches confirmed a relative conservation of the *st6gal2* gene expression in vertebrate tissues, in particular in the central nervous system, and the expansion of *st6gal1* gene expression in mammalian tissues [38]. In addition, rapid amplification of cDNA ends (5’-RACE) conducted in fish and frog tissues demonstrated the occurrence of a unique *st6gal1* transcript [38], whereas numerous studies highlighted the 5’-untranslated region heterogeneity of the mammalian *st6gal1* genes leading to several mRNA isoforms [55,77,78,79]. These data confirmed the increasing complexity in the *st6gal1* gene expression profile in higher vertebrates and suggested that phenotypic differences in the siaLome between organisms could have arisen from changes in gene regulation and from alterations in the protein coding region of *st6gal1* gene (e.g., a neofunctionalization of the *st6gal1* gene in birds and mammals) [38]. As far as the *st3gal* genes are concerned, their functional fate could not be predicted on the basis of gene expression profile alone. However, *st3gal* gene losses were tentatively linked with relaxed gene evolution and reduced gene expression. These studies indicated that the most widely expressed *st3gal* genes like *st3gal2* and *st3gal3* were also the most evolutionary conserved, whereas *st3gal* genes losses were linked to high substitution rates and to restricted tissue expression [53].

## 7. Functional Divergence and Molecular Evolution of STs

To better understand the molecular basis of STs functional divergence after WGD, evolution of the function of the various β-galactoside α2,3/6-sialyltransferases was analyzed from a structural perspective. Despite sharing primary and secondary structural similarities, ST have different acceptor substrate specificities that can be ascribed to amino acid sites. A general method to predict functionally important sites or structural role of amino acid positions in a protein is to analyze their conservation level based on the assumption that highly conserved positions among member of the same family.

**Figure 8:**
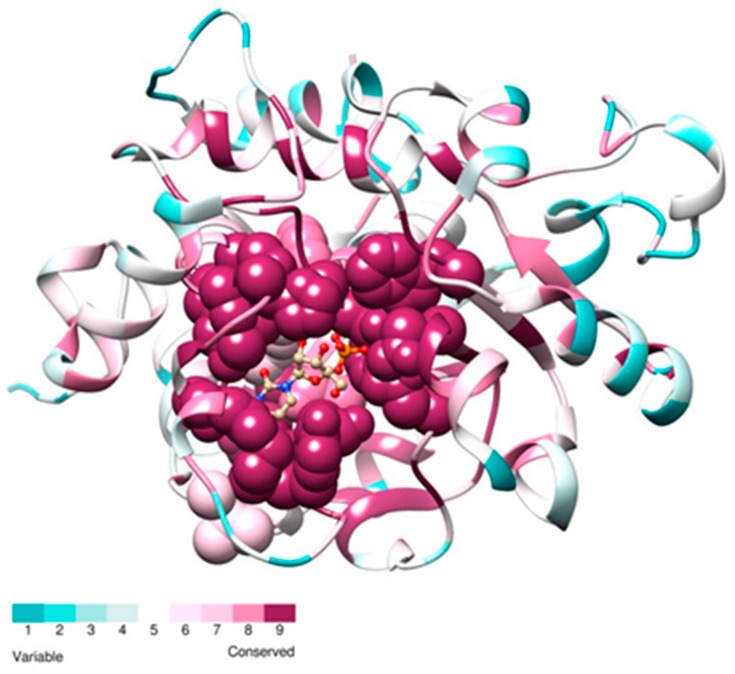
High sequence conservation near the binding site in ST6Gal. ST6Gal I and ST6Gal II show a high level of conservation in the region surrounding the ligand binding site. MSA were obtained using using profile-profile mode with clustalX and 101 vertebrate ST6Gal I and ST6Gal II sequences (Data S3). Sequence conservation was calculated with ConSurf server, the result was mapped into the PDB structure 4JS1, a crystal structure of human β-galactoside α2,6-sialyltransferase I (ST6Gal I) in a complex with cytidine (CTN) and phosphate (PO_4_). The structure is depicted in cartoon, colored by the conservation score. Ligand molecules are shown in ball and stick representation, residues at contact distance (<5 Å) from ligands are shown in sphere.

To analyze the conservation level in the ST6Gal family sequences of ST6Gal I and ST6Gal II, protein sequences were aligned separately to build a profile and a MSA comprising the two subfamilies was obtained using profile-profile mode with ClustalW. The sequence conservation was calculated using ConSurf server [80] and mapped into the crystal structure of human ST6Gal I in complex with cytidine and phosphate [81]. As shown in Figure 8, the highest conserved residues of ST6Gal are mostly located in the active site.

On the other hand, it is useful to decipher the specificity-determining positions (SDPs) of a family protein, i.e., the critical amino acids determining their functional specificity. These positions often play critical roles as they are involved in the molecular mechanisms ensuring functional diversity.

**Figure 9:**
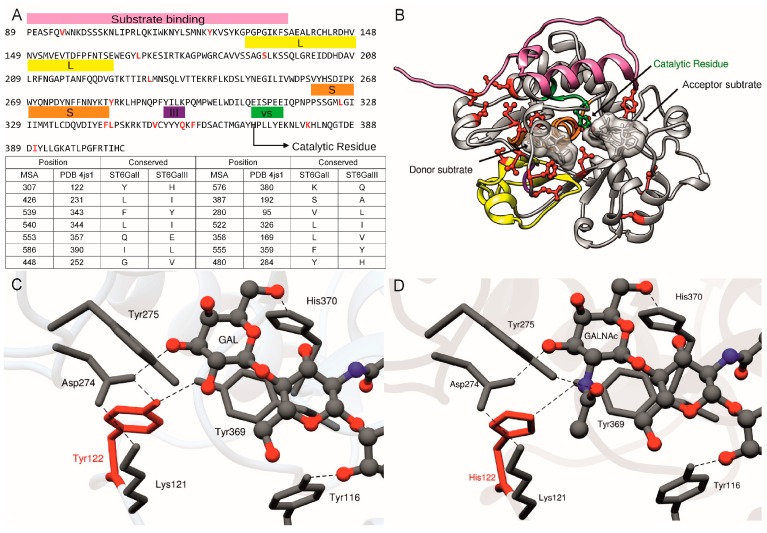
Sequence and structural mapping of SDPs between ST6GalI and ST6GalII. (**A**) The human protein sequence of ST6Gal I corresponding to PDB 4js2 is shown as a reference sequence, at the top of the panel. The functionally important regions are indicated with colored boxes and 14 SDPs of type II are highlighted in red. SDP prediction between ST6Gal I and ST6Gal II was carried out using SPEER server [82] using the MSA described previously that was composed by 48 sequences belonging to ST6Gal I and 53 of ST6Gal II (Data S3). A total of 78 and 69 SDPs of Type I and Type II respectively were predicted (Data S4). At the bottom of the panel, a table is shown with the 14 Type II SDPs with a reliable score. The first column indicates the position in the multiple sequence alignment (MSA), the second column the position in the reference sequence, the 3rd and 4th columns indicate the conserved amino acid in the ST6Gal I and ST6Gal II sequences, respectively. The SDPs are sorted according to the predictive score, from higher to lower; from top to bottom and from left to right; (**B**) The functionally important regions and SDPs are shown in the structure of the reference human ST6Gal I sequence. In addition, the surfaces of the substrates are shown; (**C**) Close-up of the glycan binding site of ST6Gal I, hydrogen bonds are denoted by dashed lines; (**D**) Close-up of the glycan binding site, where the Gal was modeled to GalNAc to represent ST6Gal II binding site.

Within a MSA, SDPs are amino acid positions that show a pattern of conservation in agreement with subfamily divergence. Two types of SDPs can be distinguished: a Type I corresponds to diverse amino acids in one group and a conserved one in the other(s) reflecting different levels of functional constraints between duplicated genes, whereas Type II positions are characterized by different conserved amino acids among groups associated to divergent constraints [82,83]. SDP prediction between the three vertebrate ST3Gal groups (GR1–GR3) led to the identification of five SDP, namely S197, Y233, V234, W304 and N307 in the reference porcine ST3Gal I structure (PDB: 2WNB) that are located in the active site [53]. These SDPs in close contact to the ST3Gal substrates are indicative of the functional divergence of each group of ST3Gal sequences in early vertebrates. As illustrated in Figure 9, SDP prediction between ST6Gal I and ST6Gal II was carried out using SPEER server [82,84]. Six SDPs corresponding to positions 95, 122, 169, 357, 359 and 380 in the reference sequence human ST6Gal I (PDB: 4js2) [81] localized at the protein surface and could reflect protein-protein interaction evolution. Two type II SDPs corresponding to L326 and F346 were found in the sialylmotif S, which is involved in acceptor and donor binding [85] and three others V352, Q357 and F359 were found in the mobile loop nearby sialylmotif III (Figure 9A,B). Interestingly, the highest scored SDP prediction corresponds to position Y122 in the reference structure that is located near the *N*-glycan binding site. A Tyr residue is conserved in ST6Gal I, whereas a His is conserved in ST6Gal II sequences. The hydrogen bonds involving the residues Y122, D274 and Y369 and the α1,3Man branch of the *N*-glycan, place the galactose (Gal) in the vicinity of CMP and the catalytic residue H370 [81] (Figure 9C). To visualize the impact of the amino acid change at this position, we performed in silico mutation Y122H and the Gal residue was modified to *N*-acetylgalactosamine (GalNAc), the monosaccharide acceptor for the ST6Gal II activity [86]. Point mutation was performed using the Dunbrack backbone-dependent rotamer library [87], the most probable rotamer was chosen and changes in the structure were followed by structure minimization (Figure 9D). The ST6Gal II H122 residue can participate in hydrogen bond with the Y369, whereas the D274 can interact with K121 and GalNAc. The model also shows that the N atom of GalNAc can be involved in a hydrogen bond with Y275. In summary they are no dramatic changes at the substrate binding site between ST6Gal I and ST6Gal II, however a semi-conservative mutation, such as Y122H, can impact in the substrate stabilization.

## 8. Conclusion

This review has reported an overview of the evolutionary history of the β-galactoside α2,3/6-sialyltransferases. The human/mouse ST3Gal and ST6Gal families are comprised of eight members with low overall sequence similarities except for the conserved sialylmotif and family motifs in the catalytic domain that are hallmarks for homologs identification in databases. Genetics, molecular phylogeny and functional genomics approaches have been used to decipher their evolutionary relationships in the context of the dynamic remodeling of genome content (gene loss/gain, segmental and whole genome duplication events). Interestingly, the *st3gal* and *st6gal* genes could be identified in the sponge *O. carmela* suggesting their ancient occurrence in the metazoans and their expansion in deuterostome lineages. The 8 human *st3gal* and *st6gal* orthologs were also identified in all vertebrate genomes with the notable exception of the *st3gal6* gene, which has been lost in bony fish. In addition, several novel and less conserved *st3gal* subfamilies have been described in non-mammalian vertebrates, some of which are restricted to birds and duck-billed platypus (e.g., *st3gal9*) or to fish (e.g., *st3gal7*) that could be associated with specialized or species-specific tasks. Finally, protein sequence and structural analyses shed light into the functional evolution of ST3Gal and ST6Gal, their enzymatic specificities and their role in cell-cell interactions and diseases.

## Figures and Tables

**Table 1:** Vertebrate β-galactoside α2,3/6-sialyltransferases ohnologs: Vertebrate β-galactoside α2,3/6-sialyltransferase sequences belonging to the ST3Gal and ST6Gal families are grouped in 4 clades with distinct evolutionary origins (GR1, GR2 and GR3 for the ST3Gal and a unique group for the ST6Gal) encompassing 9 *st3gal* and 2 *st6gal* ohnologs. Acceptor substrate preferences of the mammalian enzymes and predicted acceptor substrate preference (in blue) of the novel vertebrate enzymes lost in mammals are indicated.

Group	Ancestral (Before 2nd WGDR)	Ohnologs (After 2nd WGDR)	Fish (After 3rd WGDR)	Tetrapods			Acc. Substrate
				Amphibians	Birds	Mammals	
GR1	*st3gal1/2/8*	*st3gal1*	*st3gal1*	*st3gal1*	*st3gal1*	*st3gal1*	Galβ1,3GalNAc-Ser
		*st3gal2*	*st3gal2*	*st3gal2*	*st3gal2*	*st3gal2*	Galβ1,3GalNAc-Ser
		*st3gal8*	*st3gal8*	*st3gal8*	*st3gal8*	lost	Galβ1,3GalNAc-Ser
GR2	*st3gal3/5/7*	*st3gal3*	*st3gal3*	*st3gal3*	*st3gal3*	*st3gal3*	Galβ1,3GalNAc-R
			*st3gal3-r*				
		*st3gal5*	*st3gal5*	*st3gal5*	*st3gal5*	*st3gal5*	GM3 synthase
		*st3gal7*	*st3gal7*	lost	lost	lost	GM4 synthase
GR3	*st3gal4/6/9*	*st3gal4*	*st3gal4*	*st3gal4*	*st3gal4*	*st3gal4*	Galβ1,4GlcNAc-R
		*st3gal6*	lost	*st3gal6*	*st3gal6*	*st3gal6*	Galβ1,4GlcNAc-R
		*st3gal9*	lost	lost	*st3gal9*	lost (except in platypus)	Galβ1,4GlcNAc-R
–	*st6gal1/2*	*st6gal1*	*st6gal1*	*st6gal1*	*st6gal1*	*st6gal1*	Galβ1,4GlcNAc-R
		*st6gal2*	*st6gal2*	*st6gal2*	*st6gal2*	*st6gal2*	GalNAcβ1,4GlcNAc-R
			*st6gal2-r*				

## Supplementary Materials

Supplementary materials can be found at www.mdpi.com/1422-0067/17/8/1286/s1.

## References

[1] Guerardel Y., Chang L.Y., Fujita A., Coddeville B., Maes E., Sato C., Harduin-Lepers A., Kubokawa K., Kitajima K. (2012). SiaLome analysis of the cephalochordate branchiostoma belcheri, a key organism for vertebrate evolution. Glycobiology.

[2] Schauer R. (2004). Sialic acids: Fascinating sugars in higher animals and man. Zoology (Jena).

[3] Schauer R., Kamerling J., Montreuil J., Vliegenthart J., Schachter H. (1997). Chemistry, biochemistry and biology of sialic acids. Glycoproteins II.

[4] Varki A. (1992). Diversity in the sialic acids. Glycobiology.

[5] Lehmann F., Kelm S., Dietz F., von Itzstein M., Tiralongo J. (2008). The evolution of galactose α2,3-sialyltransferase: Ciona intestinalis ST3GAL I/II and takifugu rubripes ST3GAL II sialylate Galbeta1, 3GalNAc structures on glycoproteins but not glycolipids. Glycoconj. J..

[6] Corfield A.P., Berry M. (2015). Glycan variation and evolution in the eukaryotes. Trends Biochem. Sci..

[7] Gagneux P., Varki A. (1999). Evolutionary considerations in relating oligosaccharide diversity to biological function. Glycobiology.

[8] Koles K., Repnikova E., Pavlova G., Korochkin L.I., Panin V.M. (2009). Sialylation in protostomes: A perspective from drosophila genetics and biochemistry. Glycoconj. J..

[9] Roth J., Kempf A., Reuter G., Schauer R., Gehring W.J. (1992). Occurrence of sialic acids in drosophila melanogaster. Science.

[10] Malykh Y.N., Krisch B., Gerardy-Schahn R., Lapina E.B., Shaw L., Schauer R. (1999). The presence of *N*-acetylneuraminic acid in malpighian tubules of larvae of the cicada philaenus spumarius. Glycoconj. J..

[11] Saito M., Kitamura H., Sugiyama K. (2001). Occurrence of gangliosides in the common squid and pacific octopus among protostomia. Biochim. Biophys. Acta.

[12] Kim C. (2014). Sialic acid (*N*-acetylneuraminic acid) as the functional molecule for differentiation between animal and plant kingdom. J. Glycom. Lipidom..

[13] Angata T., Varki A. (2002). Chemical diversity in the sialic acids and related aketo acids: An evolutionary perspective. Chem. Rev..

[14] Corfield A., Schauer R. (1982). Sialic acids: Chemistry, metabolism and function. Cell Biology Monograph.

[15] Cohen M., Varki A. (2010). The siaLome—Far more than the sum of its parts. J. Integr. Biol..

[16] Schauer R. (2009). Sialic acids as regulators of molecular and cellular interactions. Curr. Opin. Struct. Biol..

[17] Corfield A.P. (2015). Mucins: A biologically relevant glycan barrier in mucosal protection. Biochim. Biophys. Acta.

[18] Angata T., Brinkman-Van der Linden E. (2002). I-type lectins. Biochim. Biophys. Acta.

[19] Kelm S., Schauer R. (1997). Sialic acids in molecular and cellular interactions. Int. Rev. Cytol..

[20] Schnaar R.L., Gerardy-Schahn R., Hildebrandt H. (2014). Sialic acids in the brain: Gangliosides and polysialic acid in nervous system development, stability, disease, and regeneration. Physiol. Rev..

[21] Gabius H.J. (2000). Biological information transfer beyond the genetic code: The sugar code. Naturwissenschaften.

[22] Gabius H.J., Andre S., Kaltner H., Siebert H.C. (2002). The sugar code: Functional lectinomics. Biochim. Biophys. Acta.

[23] Lehmann F., Tiralongo E., Tiralongo J. (2006). Sialic acid-specific lectins: Occurrence, specificity and function. Cell. Mol. Life Sci..

[24] Gagneux P., Cheriyan M., Hurtado-Ziola N., van der Linden E.C., Anderson D., McClure H., Varki A., Varki N.M. (2003). Human-specific regulation of α2–6-linked sialic acids. J. Biol. Chem..

[25] Hayakawa T., Varki A., Hirai H., Imai H., Go Y. (2012). Human-specific changes in sialic acid biology. Post-Genome Biology of Primates.

[26] Varki N.M., Strobert E., Dick E.J., Benirschke K., Varki A. (2011). Biomedical differences between human and nonhuman hominids: Potential roles for uniquely human aspects of sialic acid biology. Annu. Rev. Pathol..

[27] Li Y., Chen X. (2012). Sialic acid metabolism and sialyltransferases: Natural functions and applications. Appl. Microbiol. Biotechnol..

[28] De Mendoza A., Ruiz-Trillo I. (2011). The mysterious evolutionary origin for the GNE gene and the root of bilateria. Mol. Biol. Evol..

[29] Giacopuzzi E., Bresciani R., Schauer R., Monti E., Borsani G. (2012). New insights on the sialidase protein family revealed by a phylogenetic analysis in metazoa. PLoS ONE.

[30] Harduin-Lepers A., Petit D., Mollicone R., Delannoy P., Petit J.M., Oriol R. (2008). Evolutionary history of the α2,8-sialyltransferase (*ST8Sia*) gene family: Tandem duplications in early deuterostomes explain most of the diversity found in the vertebrate *ST8Sia* genes. BMC Evol. Biol..

[31] Simakov O., Kawashima T., Marletaz F., Jenkins J., Koyanagi R., Mitros T., Hisata K., Bredeson J., Shoguchi E., Gyoja F. (2015). Hemichordate genomes and deuterostome origins. Nature.

[32] Chou H.H., Hayakawa T., Diaz S., Krings M., Indriati E., Leakey M., Paabo S., Satta Y., Takahata N., Varki A. (2002). Inactivation of CMP-*N*-acetylneuraminic acid hydroxylase occurred prior to brain expansion during human evolution. Proc. Natl. Acad. Sci. USA.

[33] Varki A. (2001). Loss of *N*-glycolylneuraminic acid in humans: Mechanisms, consequences, and implications for hominid evolution. Am. J. Phys. Anthropol..

[34] Ng P.S., Bohm R., Hartley-Tassell L.E., Steen J.A., Wang H., LUKowski S.W., Hawthorne P.L., Trezise A.E., Coloe P.J., Grimmond S.M. (2014). Ferrets exclusively synthesize Neu5Ac and express naturally humanized influenza a virus receptors. Nat. Commun..

[35] Schauer R., Srinivasan G.V., Coddeville B., Zanetta J.P., Guerardel Y. (2009). Low incidence of *N*-glycolylneuraminic acid in birds and reptiles and its absence in the platypus. Carbohydr. Res..

[36] Sellmeier M., Weinhold B., Münster-Kühnel A. (2013). Cmp-sialic acid synthetase: The point of constriction in the sialylation pathway. Sialoglyco Chemistry and Biology I.

[37] Viswanathan K., Tomiya N., Park J., Singh S., Lee Y.C., Palter K., Betenbaugh M.J. (2006). Expression of a functional drosophila melanogaster CMP-sialic acid synthetase: Differential localization of the drosophila and human enzymes. J. Biol. Chem..

[38] Petit D., Mir A.M., Petit J.M., Thisse C., Delannoy P., Oriol R., Thisse B., Harduin-Lepers A. (2010). Molecular phylogeny and functional genomics of β-galactoside α2,6-sialyltransferases that explain ubiQuitous expression of *st6gal1* gene in amniotes. J. Biol. Chem..

[39] Petit D., Teppa R.E., Petit J.M., Harduin-Lepers A., Brockausen I. (2013). A practical approach to reconstruct evolutionary history of animal sialyltransferases and gain insights into the sequence-function relationships of golgi-glycosyltransferases. Glycosyltransferases: Methods and Protocols.

[40] Harduin-Lepers A., Tiralongo J., Martinez-Duncker I. (2013). Vertebrate sialyltransferases. Sialobiology: Structure, Biosynthesis and Function; Sialic Acid Glycoconjugates in Health and Diseases.

[41] Harduin-Lepers A., Vallejo-Ruiz V., Krzewinski-Recchi M.A., Samyn-Petit B., Julien S., Delannoy P. (2001). The human sialyltransferase family. Biochimie.

[42] Paulson J.C., Colley K.J. (1989). Glycosyltransferases. Structure, localization, and control of cell type-specific glycosylation. J. Biol. Chem..

[43] Harduin-Lepers A., Mollicone R., Delannoy P., Oriol R. (2005). The animal sialyltransferases and sialyltransferase-related genes: A phylogenetic approach. Glycobiology.

[44] Cantarel B.L., Coutinho P.M., Rancurel C., Bernard T., Lombard V., Henrissat B. (2009). The carbohydrate-active enzymes database (CAZy): An expert resource for glycogenomics. Nucleic Acids Res..

[45] Tsuji S., Datta A.K., Paulson J.C. (1996). Systematic nomenclature for sialyltransferases. Glycobiology.

[46] Kidani S., Kaneoka H., Okuzaki Y., Asai S., Kojima Y., Nishijima K.I., Iijima S. (2016). Analyses of chicken sialyltransferases related to *O*-glycosylation. J. Biosci. Bioeng..

[47] Kojima Y., Mizutani A., Okuzaki Y., Nishijima K., Kaneoka H., Sasamoto T., Miyake K., Iijima S. (2015). Analyses of chicken sialyltransferases related to *N*-glycosylation. J. Biosci. Bioeng..

[48] Koles K., Irvine K.D., Panin V.M. (2004). Functional characterization of drosophila sialyltransferase. J. Biol. Chem..

[49] Kajiura H., Hamaguchi Y., Mizushima H., Misaki R., Fujiyama K. (2015). Sialylation potentials of the silkworm, Bombyx mori; B. Mori possesses an active α2,6-sialyltransferase. Glycobiology.

[50] Harduin-Lepers A., Recchi M.A., Delannoy P. (1995). 1994, the year of sialyltransferases. Glycobiology.

[51] Takashima S. (2008). Characterization of mouse sialyltransferase genes: Their evolution and diversity. Biosci. Biotechnol. Biochem..

[52] Audry M., Jeanneau C., Imberty A., Harduin-Lepers A., Delannoy P., Breton C. (2011). Current trends in the structure-activity relationships of sialyltransferases. Glycobiology.

[53] Petit D., Teppa E., Mir A.-M., Vicogne D., Thisse C., Thisse B., Filloux C., Harduin-Lepers A. (2015). Integrative view of α2,3-sialyltransferases (ST3Gal) molecular and functional evolution in deuterostomes: Significance of lineage-specific losses. Mol. Biol. Evol..

[54] Altschul S.F., Madden T.L., Schaffer A.A., Zhang J., Zhang Z., Miller W., Lipman D.J. (1997). Gapped blast and PSI-blast: A new generation of protein database search programs. Nucleic Acids Res..

[55] Harduin-Lepers A. (2010). Comprehensive analysis of sialyltransferases in vertebrate genomes. Glycobiol. Insights.

[56] Moreau H., Verhelst B., Couloux A., Derelle E., Rombauts S., Grimsley N., van Bel M., Poulain J., Katinka M., Hohmann-Marriott M.F. (2012). Gene functionalities and genome structure in bathycoccus prasinos reflect cellular specializations at the base of the green lineage. Genome Biol..

[57] Read B.A., Kegel J., Klute M.J., Kuo A., Lefebvre S.C., Maumus F., Mayer C., Miller J., Monier A., Salamov A. (2013). Pan genome of the phytoplankton emiliania underpins its global distribution. Nature.

[58] King N., Westbrook M.J., Young S.L., Kuo A., Abedin M., Chapman J., Fairclough S., Hellsten U., Isogai Y., Letunic I. (2008). The genome of the choanoflagellate monosiga brevicollis and the origin of metazoans. Nature.

[59] Smith J.J., Kuraku S., Holt C., SaUKa-Spengler T., Jiang N., Campbell M.S., Yandell M.D., ManoUSAki T., Meyer A., Bloom O.E. (2013). Sequencing of the sea lamprey (petromyzon marinus) genome provides insights into vertebrate evolution. Nat. Genet..

[60] Braasch I., Postlethwait J.H. (2011). The teleost agouti-related protein 2 gene is an ohnolog gone missing from the tetrapod genome. Proc. Natl. Acad. Sci. USA.

[61] Ohno S. (1970). Evolution by Gene Duplication.

[62] Kumar S., Stecher G., Tamura K. (2016). Mega7: Molecular evolutionary genetics analysis version 7.0 for bigger datasets. Mol. Biol. Evol..

[63] Letunic I., Bork P. (2016). Interactive tree of life (iTOL) v3: An online tool for the display and annotation of phylogenetic and other trees. Nucleic Acids Res..

[64] Putnam N.H., Butts T., Ferrier D.E., Furlong R.F., Hellsten U., Kawashima T., Robinson-Rechavi M., Shoguchi E., Terry A., Yu J.K. (2008). The amphioxus genome and the evolution of the chordate karyotype. Nature.

[65] Henrichsen C.N., Chaignat E., Reymond A. (2009). Copy number variants, diseases and gene expression. Hum. Mol. Genet..

[66] Wolfe K.H. (2001). Yesterday’s polyploids and the mystery of diploidization. Nat. Rev. Genet..

[67] Yegorov S., Good S. (2012). Using paleogenomics to study the evolution of gene families: Origin and duplication history of the relaxin family hormones and their receptors. PLoS ONE.

[68] Hedges S.B., Marin J., Suleski M., Paymer M., Kumar S. (2015). Tree of life reveals clock-like speciation and diversification. Mol. Biol. Evol..

[69] Tamura K., Stecher G., Peterson D., Filipski A., Kumar S. (2013). Mega6: Molecular evolutionary genetics analysis version 6.0. Mol. Biol. Evol..

[70] Atkinson H.J., Morris J.H., Ferrin T.E., Babbitt P.C. (2009). Using sequence similarity networks for visualization of relationships across diverse protein superfamilies. PLoS ONE.

[71] Shannon P., Markiel A., Ozier O., Baliga N.S., Wang J.T., Ramage D., Amin N., Schwikowski B., Ideker T. (2003). Cytoscape: A software environment for integrated models of biomolecular interaction networks. Genome Res..

[72] Lynch M., Conery J.S. (2000). The evolutionary fate and consequences of duplicate genes. Science.

[73] Albalat R., Canestro C. (2016). Evolution by gene loss. Nat. Rev. Genet..

[74] Canestro C., Albalat R., Irimia M., Garcia-Fernandez J. (2013). Impact of gene gains, losses and duplication modes on the origin and diversification of vertebrates. Semin. Cell Dev. Biol..

[75] Force A., Lynch M., Pickett F.B., Amores A., Yan Y.L., Postlethwait J. (1999). Preservation of duplicate genes by complementary, degenerative mutations. Genetics.

[76] Innan H., Kondrashov F. (2010). The evolution of gene duplications: Classifying and distinguishing between models. Nat. Rev. Genet..

[77] Lo N.W., Lau J.T. (1996). Transcription of the β-galactoside α2,6-sialyltransferase gene in B lymphocytes is directed by a separate and distinct promoter. Glycobiology.

[78] Svensson E.C., Soreghan B., Paulson J.C. (1990). Organization of the β-galactoside α2,6-sialyltransferase gene. Evidence for the transcriptional regulation of terminal glycosylation. J. Biol. Chem..

[79] Wang X., O'Hanlon T.P., Young R.F., Lau J.T. (1990). Rat β-galactoside α2,6-sialyltransferase genomic organization: Alternate promoters direct the synthesis of liver and kidney transcripts. Glycobiology.

[80] Ashkenazy H., Abadi S., Martz E., Chay O., Mayrose I., Pupko T., Ben-Tal N. (2016). ConSurf 2016: An improved methodology to estimate and visualize evolutionary conservation in macromolecules. Nucleic Acids Res..

[81] Kuhn B., Benz J., Greif M., Engel A.M., Sobek H., Rudolph M.G. (2013). The structure of human α2,6-sialyltransferase reveals the binding mode of complex glycans. Acta. Crystallogr. D Biol. Crystallogr..

[82] Chakrabarti S., Bryant S.H., Panchenko A.R. (2007). Functional specificity lies within the properties and evolutionary changes of amino acids. J. Mol. Biol..

[83] Gu X. (2001). Maximum-likelihood approach for gene family evolution under functional divergence. Mol. Biol. Evol..

[84] Chakraborty A., Mandloi S., Lanczycki C.J., Panchenko A.R., Chakrabarti S. (2012). Speer-server: A web server for prediction of protein specificity determining sites. Nucleic Acids Res..

[85] Datta A.K. (2009). Comparative sequence analysis in the sialyltransferase protein family: Analysis of motifs. Curr. Drug Targets.

[86] Rohfritsch P.F., Joosten J.A., Krzewinski-Recchi M.A., Harduin-Lepers A., Laporte B., Juliant S., Cerutti M., Delannoy P., Vliegenthart J.F., Kamerling J.P. (2006). Probing the substrate specificity of four different sialyltransferases using synthetic β-d-Galp-(1 → 4)-β-d-GlcpNAc-(1 → 2)-α-d-Manp-(1 → O) (CH_2_)_7_CH_3_ analogues general activating effect of replacing *N*-acetylglucosamine by *N*-propionylglucosamine. Biochim. Biophys. Acta.

[87] Dunbrack R.L. (2002). Rotamer libraries in the 21st century. Curr. Opin. Struct. Biol..

