# Supplementary material for: Phylogenetic-Derived Insights into the Evolution of Sialylation in Eukaryotes: Comprehensive Analysis of Vertebrate β-Galactoside α2,3/6-Sialyltransferases (ST3Gal and ST6Gal)
Source: Int J Mol Sci. 2016 Aug 9;17(8):1286. doi: 10.3390/ijms17081286 (PMC5000683; doi:10.3390/ijms17081286)
Supplement: Supplementary file 1 [file ijms-17-01286-s001.pdf]

Supplementary Materials: Phylogenetic-Derived Insights into the Evolution of Sialylation in EUKaryotes: Comprehensive Analysis of Vertebrate β-Galactoside α2,3/6-Sialyltransferases (ST3Gal and ST6Gal)

Roxana E. Teppa, Daniel Petit, Olga Plechakova, Virginie Cogez and Anne Harduin-Lepers

Data S1. 124 ST3Gal sequences used in Figure 3A: N-terminal part were manually removed and multiple sequence alignment obtained with clustal Omega at the http://www.ebi.ac.uk/Tools/msa/clustalo/web site with 228 informative positions. Output format is clustal.50 ST6Gal sequences used in figure 3B: N-terminal part were manually removed and multiple sequence alignment obtained with clustal omega at the http://www.ebi.ac.uk/Tools/msa/clustalo/ web site with 256 informative positions. Output format is clustal.

```
124
Oscarel2        PRRDSTICPNIGTLLAN--PNLPRILKSKF-HPDVKV-MLTQDTKTLDEPI--NSWWK-K
Spur1/2         -GGQRCRRLWK--------KGTSEWFDEKY-NDSLLP-VWMKENKKMSEEI--GKWWL-S
Bflo1/2         -TPPVVVQPTC---KRIWQKGRSSWFDSRF-DDNIRP-VWSRANIELPADA--RKWWM-S
Csav1/2         FPNKKCGRDLD--------ASEKRWFKGRF-NPEIQP-VWTESTLEIDYLV--YDWWL-S
Cint1/2         EIQQNNKCGHQ------LDASQTRWFRARF-NPEIEP-VWTQSALEIDYLV--YDWWL-S
Sacc12          ------MPVWT--------KGEYKFFDDRY--SNIST-IWSSKHKEMTPEA--LQWWL-R
Dreri8          -RGHSCSCTSC---IAY--IGVSEWLDQRY-DQKQKP-YLTGRDDDVDPLS--LKWWL-S
Ambmex1         -PIRPCSCDAC---ISE--SKLSPWFDERF-NDTIPT-LLTKENNEIPEDV--YQWWLE-
Siltro1         --RRQCGCDTC-----VAEPEASTWFDERF-NLSVLP-LLSKQNNVIPDTV--YKWWL-T
Xlaev1          --RRQCGCDTC-----VAEPEASTWFDERF-NLSVLP-LLSKQNNVIPDTV--YKWWL-T
Mondom1         -PQRPCSCSQC---ISE--TGFAPWFDERF-NHTMQP-LLNRQNAFLENDT--YTWWM-K
Loxafr1         -PPRSCTCSHC---VNE--LRVSHWFDERF-NETMQP-LLTLHNSLMEEET--YSWWL-K
HSA1            IKHRPCTCTHC---IGQ--RKLSAWFDERF-NQTMQP-LLTAQNALLEDDT--YRWWL-R
Btaurus1        -SNRPCTCTRC---IGQ--RRVSSWFDERF-NRSMQP-LLTAKNALLEEDT--YNWWL-R
Ggallus1        -PRRPCSCSTC---ISE--LGHSLWFDQRF-NSTMQP-FLTSQNALIPEDS--YRWWL-K
Taegut1         -PRRPCSCRTC---ISE--LGHSLWFDQRF-NSTMQP-FLTSQNALIPEDS--YRWWL-K
Pytmor1         -SRRPCSCNTC---ILE---QTSAWFDERF-NITMQP-FLTTQNAFIPQES--YRWWL--
Chrypic1        YPERPCSCNSC---VPE--LGLSFWFDERF-NQTIQP-FLTTRNTFIPEES--YKWWL-K
Acarol1         -SRRPCSCSTC---ISE--QGVSLWFDGRF-NQTMQP-FLTTRNAFVPEES--YKWWK--
Latcha8         -DKKLCSCEKC---VSE--KEESAWFDERF-DPNFQP-ILMTEVQDIPSHA--LQWWL-V
Acarol8         LEQRICNCNRC-------IDHNPQWFCDHY-DASVNC-LLTRESQRIPPEV--LFWWL-K
Pytmol8         MKEEMCHHNRC------IGLSSWDWFCDHF-DASVNC-LLTPENSDIPPEV--LQWWL-V
Chrpic8         SEAKPCGCASC-----IVELSSSAWFNERY-DSAINP-LLTVHTSKIAPDV--LLWWL-K
Galgal8         -SLPTVRCTAS--------ANASAWFSARY-DAAAGP-LLTGPAHELSPDV--VHWWL-T
Melund8         LDAPAAYCTTS--------TNGSMWFNARY-NRTMGP-LLTGRAHELPSDV--VQWWL-T
Latcha2A        SRKKLCSCKLC---TSE--SGLSLWFDERY-NRTVRP-LLTKSNAFISKDI--YEWWLVS
Latcha2B        SSVKRCGCRAC-----ISEPGVSPWFDERY-NRTVRP-LLMKSNAFISEDT--YVWWL-S
Dreri2          -QQKTCACSRC---VGD--PGVSDWFDENY-DPDISP-VWIRDNIQLPSDV--YYWWV-M
Takrub2         -QQRTCECPRC---VGD--PGVSDWFDENY-DPDVSP-VWTRDNGQLPPDV--YYWWV-M
Pmar_l2         --------------------------------------LSLQ-----------------S
Latcha2C        -LLKMCTCARC-----LGDLGVSDWFDENY-NMDVLP-VWTKENINLPPDV--YYWWL-M
Strop2          RLPKTCSCRQC---LRQ--PGFSAWFDSHY-DASVSP-VWTLENRELSPDV--LHWWM-M
Ambmex2         ALPKHCACSKC-----VWEAGVSEWFDGTMIPNQISP-IWTLENMDLAPDV--QRWWM-M
Acarol2         LFVKSCSCHRC-----QVGAEDAEWFRSRY-DARISP-VWTRENMELPLDV--QRWWMER
Pytmol2         --VKRCACHRC---AIE--SIDTSWFDNRY-DSNISP-VWTKENVDLPLDV--QRWW--M
Ggallus2        --PRGCACRRC---PEDAAAAAAAWFDSRY-DGGVSP-VWTKENMELPPDV--QRWWM-M
Taegut2         --PRGCSCRRC----PAEEPGAADWFDGRY-DGTVSP-VWTKENMDLPPDV--QRWWM-M
Chrpic2         --GEGCACRRC---TED--AGALAWFTSHY-DSNISP-VWTKENMDLLPDV--QRWWM-M
Ornana2         --AKSCACRRC---MGD--TGASDWFDSHY-DSNISP-VWTRENMALPPDV--QRWWM-M
Loxafr2         --GKSCACRRC---MGD--TGASSWFDSHF-DSNISP-VWTRENMDLPPDV--QRWWM-M
Mondom2         --SKSCACRRC---MGD--AGASDWFDSHY-DGNISP-VWTKENMDLPLDV--QRWWM-M
Hasa2           -SGKSCACRRC---MGD--AGASDWFDSHF-DGNISP-VWTRENMDLPPDV--QRWWM-M
Btaurus2        LAGKSCACRRC-----MGDTGASEWFDSHF-NSNISP-VWTRENMDLPPDV--QRWWM-M
Strop8          -DNQGCVCGRC---RQEKRKQTEFFEKRF-NETVEP-LLLGKEQEMPDHV--KKWWL-R
Xlaev8a         -NSQGCICGLC---RQENRKRTEFFETRF-NGTIEP-LLLGKDQDMPDHV--KKWWL-R
Xlaev8b         INSQGCMCGHC---RQEKRKRTEFFESRF-NGTVEP-LLLGKYQYMPDHV--KKWWL-R
Ambmex8         -RHKPCSCKQC---ISE--VGVSDWFDRHF-NSSVNP-LLTAKNPEIPLSV--QRWWL-K
Protopt2        -------------------------------DPGVNP-LLTIRNAFLYNAV--KKWWL-N
Drerio1C        PSERCCACKDC-----VREKSHSTWFNELF-DPSIQL-LLTRGNYVLNKDV--YKYWK-G
Orylat1         FSKDICACDSC---MAD--LGEDAWLADHF-NLSILP-LMTRDSSALSEDT--YRWWQ-W
Tetnig1         FSRGPCACGQC---MTE--PEDDPWFTERF-NQSIQP-LMSRENSALSDET--FKWWQ-W
Takrub1         FARGPCACGQC-----MTEMDDDPWFTERF-NQSIHP-LMSRENSALSDET--FKWWQ-V
Drerio1D        HIFGVCACGQC-----VADIFYDAWFRMHY-DPNIEL-LLTKKNSILSDAL--YRWWQ-K
Drerio1A        STGGLCACNKC-----VMDLNDEDWFFIRF-NPTVPT-LLNRRNSAMRSDV--FEWWQ-G
Drerio1B        -KRAACGCNTC---VAS--RENGTWFSERY-NRTIPF-LLNSTNSELNEDI--LKWWK-G
Sacc2/6_1       ----PCTKGYT--------HRAIKRLVPTF--DPDLP-LFLNRDFRKWKDF---DRLKET
Saccl1          ----PCTKGYT--------HRAIKRLVPTF--DPDLP-LFLNRDFRKWKDF---DRLKET
bflo210         ------------------------------------------------------------
Bfl_12648       -EGDTCVPGYA--------RSKVTEMNPKF--NTNLP-LFVKKGFREWKGM--MDDGHDK
Bfl_13015       -QFGIHKVSIRWKDLRKPLYSSLVAGMKILTVPHEIP-PSIPAQAAFWKNY--CNSAL-D
```

```
Dre7          -LSQSCHSGWS--------VERMRTMLSTPAGLLKIP-VFLENGTGDWA-----------
Tru7          -LSDPCRPRWC----LNR--LKR-LPCSEG--LLDIP-VFVQQDVPSS--------WD--
Orylat7       -LSHPCQPHWC----LNH--LKPLSFSKRL---LDIP-VFVQQGRPAE--------WA--
Cmi5          VLGEKCRLSFA-------HQKMAILFPHKY-DLNLSP-FLKRNSGLTGNVF--------K
Strop5        -LQAQCRPAFA-------RTQMDRLFSSKY--TKNLS-SFIKKDKSLNESL-----YK--
Ambmex5       VLEGQCRPAFV-------KREMDRLFQERY--SMSLA-SFMRKSPHLNESS-------FK
Mondom5       VLQGECRPDFA-------KKEMHRLFAQQY-DVNASP--FINENMELEEDL-------YR
Ornana5       VLQGECRPSFV-------KKGMDRLFKEKY-SLGLSP--FVKQNSEANEAA-------YQ
Hsa5          VLQKECRPKFA-------KTSMALLFEHRY-SVDLLP-FVQKAPKDSEAES--------K
Mmu5          VLQKECRPRYA-------KTAMALLFEDRY-SINLEP-FVQKVPTASEAEL--------K
Anocar5       VLQAPCRPSFV-------KKEMSRLFGEKY--NINTS-SFIRKDINAHEDI-------FK
Taegut5       ALQEQCRPSYA-------KKEMRKLFAEKY-NMDISP--FVRKNMNEDEAL-------FK
Galgal5       VLQEQCRPSYV-------KKAMGKLFAEKY--SMDIP-PFVGKNIDDDEAL-------FK
Dre5          ILDKECRPSFA----------RQRMVTEHHGSTPTID-PFLNKNMKLDEQI-------FQ
Tru5          VLGAQCHLSRT----------RKSLLPLLPSSSHMTQ-PFLWKDVPLSDDL-------FL
Melgal9       ----------------------------------DRP-FKQELEEVLWLHK---------
Ornana4       ---TPC--------------------------THLTP-LCSSPSVFLAAEV---------
Cmi6          ------------------------------------------------------------
Hsa6          NKIQPCLSKPA-----------F-ASLLRF--HQFHP-FLCAADFRKIASL--YGSDK--
Mmu6          -MRTRNNVNNC---------FKKPAFANLLRFPQLYP-FLCRADFIKVAAM----SGINN
Anocar6       -KEAPRILDQC-------LPAVMGWMRA----NKLYP-FLCPHNFQQVSEL--HGSFS--
Taegut6       -VERRNALDTC---------LLKPAFESLLGVDKIYP-FLCANDFIRVAEF--QGSNK--
Gga6          -VERRNALNTC---------ILKPAFESLLDVEKIYP-FLCASDFIRVAEY--HGSDK--
Melgal6       -VERRNALDTC---------LLKPAFESLLDVDKIYP-FLCANDFIRVAEF--HGSDK--
Ambmex6       -RKDDHFADSC----------FNQNFKSLLQMPNVRP-FICPEYAKQATEEGSKK-----
Str6          PTDAVKEFKSC----------YNEKFRLILNTSEIEP-FLCQDFRKQSVNL-----GSNK
Spu2/6_3      -SNVTCRKGYA-------RTRLQNLYGDRF--DPDIP-LFVDSSFLDRPNA------V-N
Spu2/6_6      ------------------------------------------------------------
Spur6b        ------------------------------------------------------------
Bfl8275       -EEEVCRPHHA--------SSTIVNTHPKF--RKDIP-LFVTNDYKSYSSV------M-N
Bfl8276       -AEEQCRPHHA--------SSTIVNTHPKF--RKDIP-LFVTNDYKSYSSV--MN-----
Sacc3_l0      ------------------------------------------------------------
Latcha3       -TEGACKPHYA-------ATQMTAIFPRRF--MKPAP-MFLDISFKRWARI------K-D
Oryla3-r      -SEGACKPGYA--------AAKMTSIYPKF--MKLAP-MFLDPNFKRLGKV------S-G
Dre3A         -SEGACKPGYA--------AAKMTAIYPKF--SKPAS-MFLDRNFKRLAKV------I-N
Takrub3       -SDGVCKPGYA--------AAKMTAIYPKF--IKPAP-MFLDRNFKRLAKV-------NS
Cmi3          -TEGGCRKGYA-------SAQMTAIFPKSL------------------------------
Siltr3        -SEGVCKPGYA--------SALMNAIYPKF--SKPAP-MFLDDSFRKWAKI------R-E
Xenlae3       -SEGVCKPGYA--------SALMNAIYPKF--SKPAP-MFLDDSFRKWAKI-------RE
Hsa3          -SEGACKPGYA--------SALMTAIFPRF--SKPAP-MFLDDSFRKWARI-------RE
Mmu3          -SEGACKPGYA--------SAMMTAIFPRF--SKPAP-MFLDDSFRKWARI-------RE
Pytmol3       -SEGVCKPGYA-------SALMTAIFPKRF--SKPAP-MFLDDSFRKWARI------R-D
Acarol3       -SEGVCKPGYA--------SALMTSIFPKF--SKSAP-MFLDDSFKKWARI------R-D
Anocar3       -SEGVCKPGYA--------SALMTSIFPKF--SKSAP-MFLDDSFKKWARI------R-D
Galgal3       -SEGACKPGYA--------SAVMTAIFPRF--SKPAP-MFLDDSFRRWARI------R-D
Taegut3       -SEGMCKPGYA--------SALMTVIFPKF--SKPAP-MFLDDSFRKWARI------R-D
Melgal3       FSEGVCKPGYA--------SALMTVIFPKF--SKPAP-MFLDDSFRRWARI--RD-----
Gasac3-r      -SEGACKPGFA--------AAKMTAIYPKF--IKLAP-MFLDPNYKRYSKI------G-G
Dre3B         -SKGECKQGFA--------QAKMTLIYPKF--SKPAP-MFLDPNYKRLSKI-------SS
Pmar4         ------------------------------------------------------------
Ambmex4       DTRIACSPGSV--------ETMAAAFIENY--TRGHQ-AFLQPSNYFWVKS-----NS-T
Str4          NVHEMCIPGDV--------ASKAEILISNY--SRDHP-VFLQLSDYFWVKN-----QS-K
Ornana9       ------------------------------------------------------------
Hsa4          -KKEPCLQGEA--------ESKASKLFGNY--SRDQP-IFLRLEDYFWVKT-----PS-A
Mmu4          -KKEPCFQGEA-------ERQASKIFGNRS---RDQP-IFLQLKDYFWVKT------PST
Gga4          -NKTVCPIGEV--------ERKAAQLIGNY--TRDRP-LFLQLKDYFWVRT-----PS-L
Melgal4       -NKTVCPSGEV--------ERKAAQLIGNY--TRDRP-LFLQLKDYFWVRT--PSL----
Anocar4       DERMTCPLGEV--------EKKAAQLIANY--TRDHP-LFLQLKDYFWVRT-----PS-P
Pytmol4       DERMTCPLGEV--------EKKAAQLIANY--TRDHP-LFLQLKDYFWVRT------PSP
Latcha4       ------------------------------------------------------------
Cmi4          ------------------------------------------------------------
Orylat9       ----------------------------RI--NKTTK-LFLKLEDFFWRKY------VSQ
Dre4          PHPTLALCPNY--------RLQKKWENLNLKSMSRKPELFLKLEDFFWKDH------LSA
Tru4          ----------S--------V------------SRRTQ-LFLKLNDYFWQER-----LS-T
     LQPF---DNKRDFTE-LAEDLFTV-IPGE-NPFSPG-----VCRRCAVVGTAGRLKGARQ
     LQR----RENSDYIG-ALDKAFEV-IPNP-QRFLTRNVS--RCLRCAVVGNSGNLRNSGY
     LQSH----KDEDPAP-LLNALFDMGAPDV-DPWASRNLT--GCLRCAVVGNSGNLRQSNY
     LQSS----RGENLDK-VFENLYKIGVPRNNPFARPNHDNEAICRRCAVVGNSGNLINSKY
     LQSS----EAENLDK-TFEALYKEGVPRKDPFARLTHDREAGCRSCAVVGNSGNILNSNY
     IQQSKDTFSTSDLRN-LVGKLLEI-IPDV-MPYATNVSN--RCLQCAVVGNSGNLRDSRY
     LQA-----SDGTIKD-VTQKMFKI-ISPP-HEDETPRQN--QCRKCAVVGNSGNLLKSKY
     ----------------MVKKLFET-ISGE-EDFLEASPY--RCRRCAVVGNSGNLKNSGY
     LQGE---TKPKDIYE-VMEELFEV-IPGD-LDLMDQGPY--RCRTCAVVGNSGNLKSSNY
     LQGE---TKPKDIYE-VMEELFEV-IPGD-LDLMDQGPY--RCRTCAVVGNSGNLKSSNY
     LQRE---RTPKRLNE-TFMDLFSI-IPGDVDPLLQKGPL--ICRRCAVVGNSGNLKESHY
     LQRE---KNPKNLND-TIKELFSV-VPGDVDPMLENESV--GCRRCAVVGNSGNLKQSQY
     LQRE---KKPNNLND-TIKELFRV-VPGNVDPMLEKRSV--GCRRCAVVGNSGNLRESSY
     LQRE---KQPNNLND-TIKELFQV-VPGDVDPLLEKGSV--GCRRCAVVGNSGNLRESWY
     LQGE---KSPKNIND-TLKELFGI-IPGDRDPLQERGTF--SCRRCAVVGNSGNLRQSQY
     LQGE---KAPRSLNA-TLAELFGV-IPGDGDPLQERGSD--SCRRCAVVGNSGNLRQSQY
     LQPQ---FKSHNLNE-VLSKLFQI-VPGE-NPYSSRESQ--SCRRCAVVGNSGNLRGSSY
     LQGE---SNPKNIND-TMKELFEI-IPGDGEQLLERDAS--RCRRCAVVGNSGNLRQSQY
     LQGE---RNPKGVNE-TIQEMFEI-IPGNGDQLLERSSS--QCRRCAVVGNSGNLKQSQY
     QAG----NKNYNLSE-SIAKLFTV-VPRT-NHSGIRDPA--HCRKCAVVGNSGNLKGSNH
     LQRS---QDGKQVQK-IIKHLFEI-LPS--STAKVQDTT--QCGTCAVVGNSGRLKGSKY
     LQRS---QDGSQIQK-IIKKLFDL-LQSPTARVQGVA----QCGTCAVVGNSGRLRGSKY
     LQGS---KSRAQLQG-IIQQMFDI-LPSP--MVDAWDPS--RCRTCAVVGNSGRLRGSHY
     LQGT---PSIVPLRA-ILQKLFAV-VPAP--NGSVWDPS--HCRTCAVVGNSGRLKGSRH
     LQGP---SRGVQLQA-ILWQLFTV-LPAP--TGSLWDPN--HCRTCAVVGNSRWLKDSGH
     LQKE---GNPKNITT-VIQDLFET-IPGA-DLYTHEEPD--RCRKCAVVGNSGNLKGSGY
```

```
LQKE---GNPKNITT-VIQDLFET-IPGA-DLYTHEEPD--RCRKCAVVGNSGNLKGSGY
LQPQ---FKPHNIQQ-VLQRLFQV-IPGR-SPYGSWDPA--RCLRCAVVGNSGNLRGAGY
LQPQ---FKPHSIQQ-VLLKLFQV-VPGR-SPYGSWDPG--RCLRCAVVGNSGNLRGAGY
LQSQ---YKAQDLRT-VLNHLFEI-IPGQ-NPYQDHGWA--RCRRCAVVGNSGNLLGAGG
LQPQ---FKPYNLKN-VLGKLFEI-IPGM-NPYNSWDPQ--SCRRCAVVGNSGNLHGSGY
LQPQ---FRSHNTPE-ILSQLFQI-IPGQ-NPYQQQDPL--TCRRCAVVGNSGNLRGSSY
LQPQ---FKSQNTNE-VLEKLFQI-VPGT-------------------------------
GERS-----FMGVLNEVLSKLFLI-IPGE-NPYRSRPSP--QCLRCAVVGNSGNLRGSGY
LQPQ---FKSHNLNE-VLSKLFQI-VPGE-NPYSSRESQ--SCRRCAVVGNSGNLRGSSY
LQPQ---FKSHNTQE-VLSKLFQI-VPGE-NPYRWRDPR--HCRRCAVVGNSGNLRGSGY
LQPQ---FKSHNTHE-VLSKLFQI-VPGE-DPYRSRDPR--RCRRCAVVGNSGNLRGSGY
LQPQ---FKSHNTNE-VLGKLFQI-VPGE-NPYGSRDPR--KCRRCAVVGNSGNLRGSGY
LHPQ---FKSHNTNE-VLAKLFQI-VPGE-NPYGSRDPH--KCQRCAVVGNSGNLRGSGY
LQPQ---FKSHNTNE-VLEKLFQI-VPGE-NPYRFRDPH--QCRRCAVVGNSGNLRGSGY
LQPQ---FKSHNTNE-VLEKLFQI-VPGE-NPYRFRDPH--KCRRCAVVGNSGNLRGSGY
LQPQ---FKSHNTNE-VLEKLFQI-VPGE-NPYRFRDPH--QCRRCAVVGNSGNLRGSGY
LQPQ---FKSHNTNE-VLEKLFQI-VPGE-NPYRFRDPR--QCRRCAVVGNSGNLRGSGY
LQGR---NNGSDIQK-VLEQTFKV-IPTK-NLYEEQDNR--PCRRCAVVGNSGNLKGSRH
LQGR---NNGSDIQK-VLEQMFKV-VSTE-NLYLEQDNR--PCRRCAVVGNSGNLKGSRY
LQGR---NNASDIQK-VLEQTFKV-VPTE-NLYLEQDNR--PCRRCAVVGNSGNLKGSRY
LQGS---SNPEQVQE-ALDKLFDV-FPAE-DIYSVRDET--MCRTCAVVGNSGNLKGSKY
LQGV---HKNSNVDA-IMTKLFEV-IPNT-DPYQSKNET--QCRRCAIVGNSGNLKDSSY
LQKN---KKDSDYRA-VVEKMFSL-FPDK-TRYTNASPN--RCRTCAIIGNSGNLKGSRY
LQRE---ENPANYTQ-VVEELFRI-IPEE-GVFMDAGPQ--RCRTCSVVGNSGNLKGSNY
LQSE---RQPANFSW-VVEELFQL-IPDG-ALYMDAGPE--RCRTCAVVGNSGNLKGSQY
LQSE---RQPANFSG-VVEELFQV-IPDE-VLYMDASPE--RCRTCAVVGNSGNLKGSQY
LQ-----YKSRVNYKAVSEMLFGI-FPNE-EDYSDAGPG--RCRTCAVVGNSGNLNGSHY
LQSD---SQKANFSD-VVDILFSL-FPDE-EHYVVTDPN--RCRICAVVGNSGNILGSHY
LQGA---ADVINYTY-VVDILFSL-FPDE-DHFSDAGPD--RCRTCAVVGNSGNLLRSNY
TEP----YGFKYHVS-FVEKLMQA-LPGN-TPNIMGNDM--KCRRCVIIASSGGSIGKHL
TEP----YGFKYHVS-FVEKLMQA-LPGN-TPNIMGNDM--KCRRCVIIASSGGSIGKHL
------------------------------------------------------------
LP-----FYLSDQDCKKIDQLLDL-LPNS-EPALLTGGG--KCRRCIVVGSSGLVSGENL
KSPNDYVTAVEEVAGKKIDQLLDL-LPNS-EPALLTGGG--KCRRCIVVGSSGLVSGENL
LPPP---LGLQGSEE-MAEQALKA-LPFT-AVPTGPET----CRRCVVVGSRGILHSKNL
LPPP---LGLRGSEE-HLALALAS-LPQL-GLPPSLRGE-GGCRRCVVVGNGGVLHGSHL
LGPP---LGLHGSEE-HLALTLAS-MPEP-GLPPSLKTK-DSCRRCVVVGNGGVLQGSHL
YHPP---FGFKKHNL-TFSELIGL-LPKV-SLSEELERK--PCKRCVILGSGGIL-----
YEPP---FGFRHYVD-ELSDLLEM-MPED-GLPKELHSK--HCKRCIVIGSGGILHGLEL
YGPP---FGFHDYMY-HLQELLRL-MPAQ-DLPQALKAK--QCKRCIVLGSGSILHGLQI
YDPP---FGFRKFSY-KLQSLLKL-LPEH-GLPENLKAK--PCKHCVVMGNGGVLNGLGL
YDPP---FGFRKFSR-KLQNLLEL-LPEH-DLPEHLKTK--SCKRCIVIGSGGILHGLGL
YDPP---FGFRKFSS-KVQTLLEL-LPEH-DLPEHLKAK--TCRRCVVIGSGGILHGLEL
YDPP---FGFRKFSS-KVQSLLDM-LPEH-DFPEHLRAK--ACKRCVVVGNGGILHGLEL
YEPP---FGFHKFSE-KLKDLLEL-LPEY-DLPEGLQSK--HCKHCVVVGNGGVLHGLEL
YEPP---FGFHKFSD-KLKDLLEL-LPEH-DLPEDLKSK--HCKRCVVVGSGGILHGSEL
YGPP---FGFHRFFD-KLKKLLEL-LPEH-DLPEDLKSK--HCKRCVVIGSGGILYGSEL
YPPP---FGFLDMKK-KLEEILNL-LPVS--SEQRLGER--DCRRCVVVGNGGILKGLGL
YPPP---FGFQGLRS-KVAELLKS-LPLP-GSAQELKNRSDKCRRCVVVGNGGILRGLEL
--LP---FGLHSSGC-PMEVIARLRVMVSAAIGSSLSCL--WCQRCIVVGNGYSVHGQHI
----------------LLL------LPQQ-GLPKSIKRL--PCRKCIVVGNSFSLRGRGL
-----------------LSKLFQT-VPGD-NPYEPWDRS--RCRRCAVVGNSGNLHGSQY
FDLP---YGMRTSAE-YFRLALSK-LQSC-DLFDEFDNI--PCKKCVVVGNGGVLKNKTL
FPLP---YGIKTFET-YFSSALSK-LQSC-DLFDEFDRV--PCKRCVVVGNGGVLKNKTL
FDLP---YGIRRAER-FFQLALSK-SNNC-GLFAEDDSP--SCKKCVVVGNGGVLRNKSL
FELP---YGIKRAEQ-FFRLALSR-LQNC-GLSSEEDSI--PCRRCVVVGNGGVLRNKTL
FELP---YGIKRAEQ-FFRSALSK-LQNC-GLSNKDDSV--ACRRCVVVGNGGVLRNKTL
FELP---YGIKRAEQ-FFRLALSK-LQNC-GLSNEDDSV--ACRRCVVVGNGGVLRNKTL
LDLP---YGIRNGER-YLRLVFSK-LKNC-NLPSEINVP---CRKCVVVGNGGVLRNKTL
LDLP---YGIRRGER-FFDLALKN-LPQC-TLPEEIKNI--SCKKCVVVGNGGVLRNSTL
LPMP---FGFRDAER-TVLNILKH-MPET-EIPEDIPQD--SCLRCVVQGNGGIASRTRL
----------RTASD-VVGK-----LPDT-DIPADIKKL--PCRRCIVIGNGGILKGSKL
-------------------------LP---------------CRRCIVIGNGGLLKGSKL
YSPP---FGLRGTDK-DLMNVLKI-LPQASTMPENIERI--PCKRCIVVGSGGILLGKKL
YSPP---FGLRGTDK-DLMNVLKI-LPQASTMPENIERI--PCKRCIVVGSGGILLGKKL
--------------D--------------------------GCRRCIIVGSGGILKNKGH
FPPP---FGIKGQDN-IIQRILET-TKEY-NLTPELNSR--SCKRCIVVGNGGVLANKSL
YMPP---FGFKTQEK-LISSILSE-TKYY-GLGEHLDSL--SCKRCIIVGNGGILSNKSL
YLPP---FGFRTQER-IIDVILSA-AKNY-GLGPELDSL--ICKRCIIVGNGGILSNKSL
YLPP---FGFKTQER-IIDSILTT-TKNY-GLGEQLDSR--SCKRCIIVGNGGILSNKSL
-----------------------------------------TCKRCIIIGNGGIVANKSL
YVPP---FGIKGQDN-LIKAILSA-TKEY-RLKPALDSL--NCRRCIIVGNGGVLANTSL
YVPP---FGIKGQDN-LIKAILAA-TKEY-RLKPALDSL--NCRRCIIVGNGGVLANKSL
FVPP---FGIKGQDN-LIKAILSV-TKEY-RLTPALDSL--RCRRCIIVGNGGVLANKSL
FVPP---FGIKGQDN-LIKAILSV-TKEY-RLTPALDSL--HCRRCIIVGNGGVLANKSL
FLPP---FGIKGQDN-LIKAILSV-TKNY-HLTPALDSL--SCRRCVIVGNGGILANKSL
FVPP---FGIKGQDN-LIKAILSA-TKDY-RLTPALDSL--SCRRCIIVGNGGILANKSL
FVPP---FGIKGQDN-LIKAILSA-TKDY-RLTPALDSL--SCRRCIIVGNGGILANKSL
FVPP---FGIKGQDN-LIKAILSA-TKDY-RLTPALDSL--SCRRCIIVGNGGVLANKSL
FVPP---FGIKGQDN-LIKAILSA-TKDY-RLTPALDSL--SCRRCIIVGNGGVLANKSL
FVPP---FGIKGQDN-LIKAILSA-TKDY-RLTPALDSL--SCRRCIIVGNGGVLANKSL
YSPP---FGVKSQER-IIDILLSA-TKSY-GVGEELDSM--NCKTCIVVGNGGILANRSL
YPPP---FGVRTQER-IIDNILAA-TRSY-FLGPELDSI--PCKKCIIIGNGGILFNKSL
------------------------------------------------------------
YTLP---FGTKGIED-LILKILSI-TTSF-NLPPAMKSL--QCKTCVVVGNGNRLQNSSL
YRLP---YGTYGSEE-LLIKFLAL-TNRY-HVPEDIKRL--RCRRCVVVGNGHQLKNSSL
------------------------------------------------------------
YELP---YGTKGSED-LLLRVLAI-TSS--SIPKNIQSL--RCRRCVVVGNGHRLRNSSL
YELP---FGTKGSED-LLLRVLA--ITSY-SIPESIKSL--ECRRCVVVGNGHRLRNSSL
YELP---YGIKGSED-VLLRLLS--VTSY-SLPESIQSL--KCRRCVVVGNGHRLRNSSM
```

```
YELP---YGTKGSED-VLLRLLSV--TSY-SLPESIQSL--KCRRCVVVGNGHRLRNSSM
YELP---YGTKGAEE-ILLRLLA--VTSY-SLPESVQSL--KCRKCAVVGNGHRLRNSSM
YELP---YGTKGAEE-ILLRLLAI------TSYSLPESI--QCRKCAVVGNGHRLRNSSM
-------------------------------------SL--PCKTCVVVGNGYIIKNSSL
-------------------------LP---------------CKTCVVIGNGYILKNSSL
QPLP---YGIKGSEL-LLLKVLSA-TKSY-DMPAHIESL--ECRTCVVVGNGFAIKNTSL
EALP---YGIKGSEL-LLLKVLAA-ISSF-TMPANIELD---CRTCAVIGNGFALKNSSL
RALP---YGMKGSEL-MLLKVLAV-IPNY-EIPAKIESL--ECRTCVVIGNGFAIKNSSL
GKLIDSFDIVI-RMNRSPVKGYEVDVGSKTSYHLVYPESAVGYRGAES--SGKLVLFPFK
GTAIDKHDVVV-RINQAKVKGFEKDVGQKETHRLMYPESFMDIAP-----ETNFVLLSFK
GEEIDGYDLIF-RMNDAPTKGWEKDVGHRTTHHFMYPESATDLPD-----DVSFVLLNFK
GKNIDSHDFVI-RLNKGPTEGFENDVGRKTTHRFMYPATASSLAQ-----GVSLVLLPFQ
GNVIDGHDFVI-RMNKGPTYNYENDVGSKTTHRFMYPTTASSLPQGKLFSGVSLVLVPFQ
GQLIDSHDYIM-RMNTAKTVGFEKDVGSRTTHHFMYPESFVEVIG-----ETKFVLIPFK
GALIDSHSTVI-RMNKAVTVGYDEDVGYRTTHHFLYPESAIHLRP-----GVHLVLLPFK
GMWIDNHDFIF-RMNYAPTVGYEADVGGRTTHQFIYPESAAKLQE-----NATLIVIPFK
GPEIDEHDFVL-RMNHAPTARFEKDVGGKTTHHFVYPESVRDLQA-----NVSMILIPFK
GPEIDEHDFVL-RMNHAPTARFEKDVGGKTTHHFVYPESVRDLQA-----NVSMILIPFK
GKDIDSHDFVL-RMNRAPTAGFEVDVGRKTTHHLVYPESFRELAG-----NVSMILVPFK
GSQIDEHDFVL-RMNKAPTMGFEADVGSKTTHHLVYPESFRELGE-----NVSMILVPFK
GPEIDSHDFVL-RMNKAPTAGFEADVGTKTTHHLVYPESFRELGD-----NVSMILVPFK
GPQIDSHDFVL-RMNKAPTAGFEADVGRKTTHHLVYPESFRELAE-----NVSMVLVPFK
GQDIDSHDFVL-RMNRAPTIGYESDVGSKTTHHFVYPESYKELAE-----NVSMIVIPFK
GPDIDSHDFVL-RMNRAPTAGYESDVGGKTTHHFVYPESYRELAA-----NVSMILIPFK
GQEINGHDFIM-RMNQAPTVGFEPDVGSKTTHHFVYPESYRELGE-----EVSMILIPFK
GQDIDAHDFVL-RMNRAPTAGFESDVGSKTTHHLVYPESFRDLAE-----NVSMIVIPFK
GQEIDNHDFVF-RMNKAPTVGFESDVGSKTTHHFVYPESYKELGE-----NVSMVLIPFK
GKEIDAHHFVI-RMNRARTAGFEPDVGIKTTHHLMYPESSQDLQP-----GVHLVLLPFK
GENIDSHHFVL-RINTAPTAGFEEDVGAKTTHHFMYPESAMNLHA-----GVHLMLVPFK
GQKIDSHQFVM-RMNTAQIAGFQEDVGTRTTHHFMYPESAVNLHP-----GIHLVLVPFK
GREIDSHHWVL-RMNRAQTAGFEEDVGTRTTHRFMYPESAVDLQP-----GVHLVLVPFK
GLQIDAHHWVL-RMNRAKTAGFEMDVGARTTHHFMYPESAMNLWP-----GVHLVLVPFK
GLRIDAHDWVL-RMNRAKIAGFELDVGMRTTHHFMYPESAVDLEP-----GVHLILVPFK
GNQIDHNNFVM-RMNRAPTTGFESDVGSKTTHHFMYPESAKNLPA-----NISLVLIPFK
GNQIDHNDFVM-RMNHAPTTGFESDVGSKTTHHFMYPESAKNLPA-----NVSLVLIPFK
GPVIDGHDFIM-RMNLAPTVGYEEDAGSRTTHHFMYPESAKNLAA-----NVSFVLVPFK
GATIDGHNYIM-RINLAPTVGFEEDAGSHTTHHFMYPESAKNLAA-----NVSFVLVPFK
GKEIDQHDFVMRRINQAPTLGFETDVGARTTHHFMYPESAKNLPA-----NVSFVLVPFK
GKAIDMHDFIM-RINQAPTVGFEADVGSRTTHHFMYPESAKNLAP-----NVSFVLVPFK
GKDIDGHEFIM-RMNQAPTVGFEMDVGSRTTHHFMYPESAKNLPA-----NVSFVLVPFK
--------VPM-RMNQAPTAGFETDVGSRTTHHFMYPESAKNLPA-----NVSFVLVPFK
GREINGHDFVM-RMNQAPTVGFEADVGSRTTHHFMYPESAKNLPA-----NVSFVLVPFK
GQEINGHDFIM-RMNQAPTVGFEADVGSRTTHHFMYPESAKNLPA-----NVSFVLVPFK
GHEIDGHDFIM-RMNQAPTVGFEGDVGSRTTHHFMYPESAKNLPA-----NVSFVLVPFK
GPEIDGHDFVM-RMNQAPTVGFEGDVGGRTTHHFMYPESAKNLPA-----NVSFVLVPFK
GREIDGHNFIM-RMNQAPTVGFEADVGGRTTHHFMYPESAKNLPA-----NVSFVLVPFK
GRDIDGHNFIM-RMNQAPTLGFEQDVGSRTTHHFMYPESAKNLPA-----NVSFVLVPFK
GQDVDGHNFIM-RMNQAPTVGFEQDVGSRTTHHFMYPESAKNLPA-----NVSFVLVPFK
GRDVDGHNFIM-RMNQAPTVGFEQDVGSRTTHHFMYPESAKNLPA-----NVSFVLVPFK
GQDVDGHNFIM-RMNQAPTVGFEQDVGSRTTHHFMYPESAKNLPA-----NVSFVLVPFK
GPDVDGHNFIM-RMNQAPTVGFEQDVGSRTTHHFMYPESAKNLPA-----NVSFVLVPFK
GKNIDSHGLIF-RMNGARTYGFEKDVGSKTTHHFMYPESAVHVQP-----GVHLVLIPFK
GKKIDSHGLIF-RMNGARTYGFENDVGSRTTHHFMYPESAVNLQP-----GVHLVLIPFK
GKKIDSHRFIF-RMNGARTYGFENDVGSRTTHHFMYPESAVHLQP-----GVHLVLIPFK
GKNIDSHQIVL-RMNGAKTAGFEEDVGSRTTHHFMYPESAVNLPP-----GVHLVLISFK
GAKIDSHDFVM-GMNRAKTSGFEKDVGNKSTHYFMYPESAHHLPP-----GVHFVLIPFK
GRLIDAHNFVI-RINMGPTKGYEDDVGSKTTHRFIYPESAVDFDN-----STYLVLSPFK
GALIDTSDLVI-RMNKAPTKGFEKDVGAKTTHHVMYPESAVDLSN-----TTSLVLVPFK
GRLIDSSDFII-RMNQAPTLGFEEDVGTKTTHHVMYPESAKDLDN-----GTSLVLIPFK
GRLIDSSDFII-RMNQAPTSGFEDDVGTRTTHHIMYPESAKDLDN-----GTSLVLIPFK
GVLIDAHDLVI-RINKGPTEGFERDVGLKTTHRMIYPESAVDMDN-----STYLVLIPFK
GQLIDSHDFVI-RINKGPTKGYETDVGSKTTHRIMYPESAMDLDN-----STHLVLLPFK
GRLIDLHDFVL-RINKGPTKGFEKDVGSKTTHRIMYPESAVDLDN-----STHLVLFPYK
GSLIDRYDVVI-RMNDAPVRNYERDVGSITAFRMLYPESATAKRIDIS--DAYIVFLPYK
GSLIDRYDVVI-RMNDAPVRNYERDVGSITAFRMLYPESATAKRIDIS--DAYIVFLPYK
------------RMNDAPVKGYESDVGSRTDFRFFYPESSTLNQADLDS-GTYYVVVMYK
GSTIDKYDIVM-RMNEAPVHGYEKDIGSKTTFRFLYPESATSKEVDP---DADYIIVPYK
GSTIDKYDIVM-RMNDAPVHGYEKDIGSKTTFRFLYPESATSKEVDP---DADYIIVPYK
GAHIDHANIII-RVNNAPVFGFESDAGSRTTIRLIYPEGAPSHIQEYER-TEVVALVVFK
GAHIDQYDVII-RMNNAPVSGFERDAGSRTTVRLLYPEGAPHSGNEYRE-TTTVALVVFK
GPHIDQYDVII-RMNNAPVVGFERDVGSRTTIRLMYPEGAPHSANEYRK-TSMIALVVFK
------------RLNSAPIHGFTQDVGNKTTIRMSYPEGTPKSLHDYDP-HMLFVAVMYK
GQMVDQFDIVI-RLNNAPVHGYAQDVGNKTTIRMTYPEGAPVSEQEYQH-SSLFVTVLFK
GQAVDQFDIVI-RLNNAPVKGFEKDVGNKTTIRMTYPEGAPVSEHEYHH-NGLFVAVLFK
GHILNQFDVVI-RLNSAPVQGYTDHVGNKTTIRMTYPEGAPLSDLEYYS-NDLFVTVLFK
GPALNQFDIVI-RLNNAPVQGYADHVGNKTTIRMTYPEGAPLSEHEYYS-NGLFVAVLFK
GHTLNQFDVVI-RLNSAPVEGYSEHVGNKTTIRMTYPEGAPLSDLEYYS-NDLFVAVLFK
GHALNQFDVVI-RLNSAPVEGYSEHVGNKTTIRMTYPEGAPLSDVEYYA-NDLFVTVLFK
GYVLNQFDIVI-RLNSAPVQGYTDDVGNKTTIRMTYPEGAPLSQHEYYP-DSLFVAVLFK
GHLLNQFDIVI-RLNDAPVQGYTDHVGNKTTIRMTYPEGAPLSEHEYPP-ASLFVAVLFK
GHLLNQYDIVI-RLNDAPVQGYTDHVGNKTTIRMTYPEGAPLSEHEYPP-ASLFVAVLFK
GHLLNRFDIII-RLNSGPLQDFSADVGNRTTIRMSYPESCPKVWEDTDP-DLKYVAVIFK
GSLINRFDVVI-RLNSGPLGEYTADVGNRTTIRMSYPEGTPLHWVDSDP-DITFVAVVYK
GKMIDSHHVII-RLNDAPVKEYKKDVGERTSIRLFFPESALPDPLENSDNNTLMVFVPFK
GQKIDSFDIII-RLNDAPVKGFQEDVGEKTTIRLFFPESALPNPLDNNEEDTLMVLVPFK
GNQIDSHDFIM-RLNSGPVIGYENDVGNKTTFRLCYPESIISDPSQYDA-NTILVFVPFK
GEKIDSYDVII-RMNNGPVLGHEEEVGRRTTFRLFYPESVFSDPIHNDP-NTTVILTAFK
GATIDSYDVII-RMNNGPVLGHEEEVGTRTTFRLFYPESVFSDSSHYDP-NTTAVLVVFK
GETIDSFDVVI-RMNNGPVVGYEEDVGRRTTYRLFYPESVFSDPIHYDP-NTTAVLVVFK
GEKIDSYDVII-RMNNGPVIGYEEDVGRRTTFRLSYPESIFSDPIHYDP-NTTVVLIVFK
```

```
GGKIDSYDVII-RMNNGPVIGYEEDVGRRTTFRLSYPESIFSDPIHYDP-NTTVVIIVFK
GGKIDSYDVII-RMNNGPVIGYEEDVGRRTTFRLSYPESIFSDPIHYDP-NTTVVIIVFK
GSKIDSYDVII-RMNDAPVVGYEEDVGRKTTFRFCYPESIFSDPQHYDE-NTTIVLLAFK
GKKIDSYDIII-RMNDGPVLGYEDDVGRKTTFRLCYPESIFSNSLHYDP-NSTVVLLMFK
GEVIDNFDVVF-RLNSAPTINHENDVGRKTTFRMAYPESAFRRPEQYDP-GWTFVLMMFK
GSHIDQYDAVF-RLNVAPIKGYESDVGTKTTVRAVYPESSNIGSKAYTQ-NWTLLVVPFK
GSHIDQYDAVF-RLNIAPIKGYESDVGTKTTIRAVYPESSNIGSKAYTQ-NWTLLVVPFK
GPQIDDFDIVI-RMNNGPVKGYEEDVGHKTTIRMSYPEGALQDPGGYHQ-DSLFLMVPFK
GPQIDDFDIVI-RMNNGPVKGYEEDVGHKTTIRMSYPEGALQDPGGYHQ-DSLFLMVPFK
GRVIDEYDVVI-RMNVSPVKGYEKDVGTKTTIRITYPEGAPRKNNLYDV-DALLGIVVFK
GSKIDEYDVII-RLNGAPVKGYEKDVGAKTTIRITYPEGAIQKAEGYEK-DSLFVFAGFK
GSRIDQYDVVI-RLNEAPVKGYSKDVGSKTTMRITYPEGAIQKPENYED-NSLFVFSAFK
GSRIDEYDVVV-RLNEAPVSGYTRDVGSKTTMRITYPEGAIQKPERYEK-DSLFVFSAFK
GSRIDEYDVVV-RLNEAPVSGYGKDVGTKTTMRITYPEGAIQKPDSYEK-DSLFVFSAFK
GSKIDDYDVVI-RLNEAPVKGFEKDVGSKTTLRITYPEGAIQKPERYEK-DSLFVFAGFK
GSKIDEYEVVV-RLNSAPVKGFEKDVGSKTTLRITYPEGAIQKLEQYEK-DSIFVLAGFK
GSKIDEYEVVV-RLNSAPVKGFEKDVGSKTTLRITYPEGAIQKLEQYEK-DSIFVLAGFK
GSRIDDYDIVV-RLNSAPVKGFEKDVGSKTTLRITYPEGAMQRPEQYER-DSLFVLAGFK
GSRIDDYDIVI-RLNSAPVKGFERDVGSKTTLRITYPEGAMQRPEQYER-DSLFVLAGFK
GLKIDDYDIVI-RLNSAPVKGFEKDVGGKTTMRITYPEGAIQKPEQYEK-DSLFVLAGFK
GVKIDDYDIVI-RLNSAPVKGFEKDVGGKTTMRITYPEGAIQKPEQYEK-DSLFVLAGFK
GVKIDDYDIVI-RLNSAPVKGFEKDVGGKTTMRITYPEGAIQKPEQYEK-DSLFVLAGFK
GLKIDDYDVVV-RLNSAPVKGFEKDVGGKTTLRITYPEGAIQKMEQYEK-DSLFVLAGFK
GLKIDDYDVVV-RLNSAPVKGFEKDVGGKTTLRITYPEGAIQKMEQYEK-DSLFVLAGFK
GLKIDDYDVVV-RLNSAPVKGFEKDVGGKTTLRITYPEGAIQKMEQYEK-DSLFVLAGFK
GQRIDDFDVVV-RLNEAPVRGFERDVGSKTTMRITYPEGAIQKTERYEA-QSLFVLSAFK
GTKIDQYDVVV-RLNEAPVAGFEKDVGSKTTMRITYPEGAIQRAERYEK-SSLFVLSAFK
-------------MNDAPLRGHVGDVGSRTTMRLCYPESAHVRREDFDP-SALLAFVPFK
GHQINKYDIVI-RLNNAPVHQYQSDVGNKTTMRLFYPESADFDTTLENNPETLMVLVPFK
GETINKYDVVI-RINNAPVHKYEKDVGSKTTMRLFYPESADFDPQLDNNPDTLLVLVPFK
-----------------------------------------------------------K
GDAINKYDVVI-RLNNAPVAGYEGDVGSKTTMRLFYPESAHFDPKVENNPDTLLVLVAFK
GGVINKYDVVI-RLNNAPVAGYEGDVGSKTTIRLFYPESAHFDPKIENNPDTLLVLVAFK
GDTINTYDVVI-RLNNAPVHGYEQDVGSKTTMRLFYPESAHFDPQAENNPNTLLVLVPFK
GDTINTYDVVI-RLNNAPVHGYEQDVGSKTTMRLFYPESAHFDPQAENNPNTLLVLVPFK
GDVINKYDVVI-RLNNAPVHGYEHDVGSKTTMRLFYPESAHFNPKTENNPNTLLVLVAFK
GDVINKYDVVI-RLNNAPVHGYEHDVGSKTTMRLFYPESAHFNPKTENNPNTLLVLVAFK
GNEIDKYDVVI-RLNSGPVRGYEKDVGTKTTMRLFYPESAILDTYKHDTSDTLEVFVPYK
GAKINTYDVVI-RINNGPIRGFEKDVGNKTTLRLFYPESAIANPSREDNPDTLHVFVPFK
GRIINNYDVVI-RLNDAPVRGYEEDVGNKTTLRIFYPESASSNPRLHNEEDTLMVLVPFK
GEIINKYDVVI-RLNDAPVRGFEEDVGNKTTLRLFYPESASYNPGIHNDPDTLLVLVPFK
GNRINKYDVVI-RLNEGPVRDYEDDVGNKTTMRFFYPESASHNPDLHNDPDTLMVLVPFK
VLDIEWLKSIFTTHPI-------SKGWTHLPTNLGLKPT--DAMVIHPEFIYYVAKTW-L
VIDLQWARSAITTGEI-------TKTYTNVRRKIRVTPS--KILFYNPALMYHIHREW-I
PLDLKWMKTSLTDGSI-------TRTWTRVKGRIKANKT--KILVYNPAFFKYVNDKW-T
PQDVKWLLSALTTGEL-------TNTYQPVISRVTCDKS--KIVIISPTFIRYVHDRW-T
PLDIKWLLSALTTGEI-------TRTYQPLVRRVTCDKS--KITIISPTFIRYVHDRW-T
PLDVSWLISALTTGTI-------VRTYMPVRKQINVSKX--MIEVYNPAFMYYVHKTW-N
LKDMQWLSSALSTGEI-------KMTYMRVKNRIDADKD--KVMVVNPAFFKYTHDRW-T
VLDIEWVISALTTGTI-------SHTYAMVPSRIKTSRD--KILVYNPIFMKYVYENW-L
TLDLQWLASALTHGTI-------NRTYVQVPRKIRVSKD--KVLVYSPELMKYVYDKW-L
TLDLQWLASALTHGTI-------NRTYVQVPRKIRVSKD--KVLVYSPELMKYVYDKW-L
TMDLQWLISALTKGTI-------NFTYVPVPRKIHVNRE--KILIYHPAFIKYVFDSW-L
ITDLQWVVSATTKGTI-------SHTYVPVPMKIKVKKE--KILIYHPAFIKYVFDNW-L
TIDLEWVVSAITTGTI-------SHTYIPVPAKIRVKQD--KILIYHPAFIKYVFDNW-L
TVDLEWVISATTKGTI-------SHTYVPVPAKIKVKKN--KILIYHPAFIKYVFDSW-L
TLDLRWIVTALTTGTI-------NFTYVPVPRKIKVRKE--KVLIYNPSFIKYVYENW-L
TLDLRWVVTALTTGTI-------NFTYVPVPRKIKVKKE--KILVYNPTFMKYIYENW-L
ILDLRWVISALTNGTI-------NSTYIPVPRKIKANKD--KIMVYHPTFMKYVYDNW-L
TLDLRWIVSALTTGTI-------NSTYVPVPRKIKVKRD--QILVYHPVFIKYVYDHW-L
TLDLRWIISALTTGTI-------NSTYIPVPRKIKANKN--KILVYHPVFIKYVYDNW-L
IMDFEWIRSALTTGEI-------TRTYFRVQQFIKADKD--KVLIINPTFFKYVCDHW-T
PLDLKWLASAFSSGEI-------PSTYRRVKQFVEADKS--QVRIINPAFLKYIQDKW-T
PLDLKWLASALSTGDI-------RHTYKRVKQFIKADKS--KVRILYPGFLKYIQDKW-T
PLDLKWVTSAFSTGEL-------RYTYTRVKQFIEADRS--QVLILNPAFLKYIHDKW-T
PLDLKWVTSAFSTGEL-------THTYTRVKQFIKADRN--KVLILSPAFLKYIHENW-T
PLDLQWVASAFSTGEL-------THTYTRVKRFIKADRN--KVLILSPAFLKYIHDNW-T
TLDLQWVISALTTGSI-------KHTYIPVPAKIRVKQD--KILIYNPAFFKYIHDKW-T
TLDLQWVISALTTGSI-------KHTYIPVPAKIRVKQD--KILIYNPEFFKYVYHNW-S
TLDLLWITSALSTGQI-------RFTYAPVKQFLRVDKD--KVQIFNPAFFKYIHDRW-T
TLDLVWITSALSTGQI-------RLTYAPVKQFLRVDKD--KVQIFNPAFFKYVHDRW-T
VLDLMWIASALSTGEI------TRYGQKPLDVNIKTDHSRLQVLIYNPAFFKYIHDSW-T
TLDLLWITSALSTGEI-------RFTYAPVKQFLRVDKD--KVQIYNPAFFKYIHDKW-T
ALDLLWITSALSTGQI-------RFTYAPVKAFLRVDKE--KVQIYNPAFFKYIHDRW-T
ALDLLWIASSLSTGQI-------RFTYAPVKAFLRVDKE--KVQIYNPAFFKYIHDRW-T
ALDLLWIASALSTGQI-------VFTYAPVKPFLRVDKE--KVQIYNPAFFKYIHDRW-T
ALDLLWIASALSTGQI-------RFTYAPVKPFLRVDKE--RVQIYNPAFFKYIHDRW-T
TLDLLWIASALSTGQI-------RFTYAPVKPFLRVDKE--KVQIYNPAFFKYIHDRW-T
TLDLLWIASALSTGQI-------RFTYAPVKPFLRVDKE--KVQIYNPAFFKYIHDRW-T
ALDLLWITSALSTGQI-------RFTYAPVKPFLRVDKE--KVQIYNPAFFKYIHDRW-T
ALDLLWIASALSTGQI-------RFTYAPVKSFLRVDKE--KVQIYNPAFFKYIHDKW-T
ALDLLWIASALSTGQI-------RFTYAPVKSFLRVDKE--KVQIYNPAFFKYIHDRW-T
ALDLLWIASALSTGQI-------RFTYAPVKSFLRVDKE--KVQIYNPAFFKYIHDRW-T
VLDLLWIASALSTGQI-------RFTYAPVKSFLRVDKE--KVQIYNPAFFKYIHDRW-T
ALDLLWIASALSTGQI-------RFTYAPVKSFLRVDKE--KVQIYNPAFFKYIHDRW-T
LQDLKWITSALTTGEV-------TYTYTRMKKFIKADKD--KVLIFNPSFFKYVNDNW-T
LQDLKWITSALTTGEV-------TYTYTRVKKFIQADKD--KVLIFNPSFFKYVHDNW-T
LQDLKWITSALTTGEV-------TYTYKRVKKFIQADKD--KVLIFNPSFFKYVHDNW-T
PQDLKWVASAFSTGEL-------KFTYMRVKQFIKADRD--KVLIFNPSFLKYIHDNW-I
PLDMEWIVSALTTGNI-------KFTYVRVVSKIHLDQD--KVMILNPAFLKYIHDNW-T
```

```
VLDIEWLISSFTTKNI-------TRTYKKVRASINANRH--KVMILHPAFIKYVHEIW-L
TLDLQWIISALTTGTI-------SRTYMRVKERIQADKD--KVVVINPRFIKYVYESW-L
TLDLQWIISALTTGTI-------KHKYVPVMSRIKANKD--KVVIYSPTFFKYVYESW-L
TLDLQWIISALTTGAI-------KHTYVPVMSRIKANKD--KVVIYSPTFFKYVYESW-L
TLDLQWLISVFTTKHI-------DRTYIPVKPTIKANRN--KVMVLHPSFLKYVQEHW-L
VKDMQWLISVFTTKHI-------TSTYLKVRPTINADRE--KVMIIHPAFIKYVYESW-L
ILDMQWLISIFTTKHI-------TRTYMSVPSTINADRD--KVMILHPEFIKYVNDKW-A
LSDYQWLYDVIKGKNP--LKRIGAHFWKSVPKMFDKNVN--KIRILNPYIHEEAKVYH-L
LSDYQWLYDVIKGKNP--LKRIGAHFWKSVPKMFDKNVN--KIRILNPYIHEEAKVYH-L
SNDLNWLQAILTKVKP------TGQFWKGVAQKLDIDPA--QFLILNPQVLHVAARDV--
TDDLAWLLAVVQKEKP------EGSFWKEIGETLNVDSS--HIHILNPQIARSTSYEH--
TDDLAWLLAVVQKEKP------EGSFWKEIGETLNVDSS--HIHILNPQIARSTSYEH--
SLDLAWMTSVVTKEPL--VWWSKLWFWKDVIDSIPLQPE--NVRILNPEIMYRTGQVL-Q
SLDLDWLISVVTRRPL--GFWSKLWFWREVVEDIPLTRE--SFRILHPEIIRRTGQVL-Q
SLDLDWLTSVITKKPL--SFWSKMWFWREVVDDIPLSPE--SFKILHPEIIHKTGQVL-Q
GVDFSWLKAMVKKEEVVSIFIHKLWFWQSVVESIPLKVE--NFRIFNLDIIRETALNF-L
HADFRWLEAVLKNQKL--SAWNRFFFWKRVTDKIPLNSS--QFRVLNPLIIKETAIDI-L
SADFRWLQAMLKNESL--SIWSRMFFWKQVVENIPVQSK--QFRILNPLIVKETAIDI-L
SVDFNWLEAMLKNETM--PLWVQLFFWKHVAEKIPLQSK--QFRILNPVIIKNTAIDM-L
SVDFNWLQAMVKNESV--PLWVRLFFWKQVAEKIPLQSK--QFRILNPVIIKETALDI-L
SVDFNWLQAMVKKETL--PFWVRLFFWKQVAEKIPLQPK--HFRILNPVIIKETAFDI-L
SVDFKWLQAMVKNESL--PFWVRLFFWKQVAEKVPLQPK--HFRILNPVIIKETAFDI-L
SVDFGWIQAVVKNETL--PLWMRLFFWKQVAEKIPVMPK--QFRILNPAIIKETALDI-L
GVDFNWLQAMVKNETL--HLWVRLFFWKQVAEKIPFTPK--QFRILNPVIIKETALDI-L
SVDFNWLQAMVKNETL--SLWIRLFFWKEVAKKIPFTSK--QFRILNPVIVKETALDI-L
SVDFHWLRAMISRTPV--SLWDRLFFWQNVPMSVPVKTS--QFHLLNPQIIREMALDL-L
GVDVSWTTAMINRISV--PLWDWLFFWQKVPDHIPLDSH--RFRLLNPGVIRETALDL-L
PLDFLWLREVLLKTKN----KTKEGFWRQPPQEWNWNIS--QLRILNPYVTYEATYKL-L
AEDFIWVKEILRKTPS----KTSKGFWKPPPQVWNGKAS--HLRVLNPFVTYETTYII-L
PLDLRWLVDVIVKNKV------VRGFWKKPPASIIYRNN--QIRILNPSIVKRAAFDL-L
PHDLRWLLELLMGDKI-----NTNGFWKKPALNLIYKPY--QIRILDPFIIRTAAYEL-L
PQDLRWLVEILLGKKI-----NTQGFWKTPALKLIYKQY--QIRILDPYIISEAAFQM-L
PYDLKWLWDLLSGQRI-----KTSAFWKKPALQLIYNAQ--QIRILDPAITRRTAHDM-L
PRDLKWLWEILGGQKI-----SAKGFWKKPALNMIYKSS--QIRILDPSITRKTAYEW-L
PRDLKWLWEILGGQKI-----SAKGFWKKPALNMIYKSN--QIRILDPSITRKTAYDW-L
PRDLKWLWEILGGQKI-----SAKGFWKKPALNMIYKSN--QIRVLDPSITRKTAYDW-L
PHDVKWLSELLLHKRA-----STFGFWRKPALKMMYDLE--QIRILNPTLVRKVAHGL-L
PHDVKWLSELLMHKRV-----STYGFWRKPAMNLIYKPH--QLRVLNPFILRQASQNL-L
PLDLVWLETIIRGRKM----NSRDGFWKKIPLSVPKPTK--DFRIFNPEILQDTASM--M
TNDLKWFDTVIEGRDL--------GFWSTTVDFVPKPRK--DLRLYNPEITSELSFNL-I
ANDLKWLDTIIEGRDL--VSKKLKGFWSTTVDFVPKPRK--DLRLYNPEITSELSFNL-I
SLDFLWLQRIIGKQSV---GDIASRFWKSIAHAIPKNAD--KFRIVNPALLQETSFDL-I
SLDFLWLQKIIGKQSV---GDIASRFWKSIAHAIPKNAD--KFRIVNPALLQETSFDL-I
LADLNWLDSVLHRKTP-----STKGFWSKIAMSVPKNPR--QTRIINPLIIREASFDLBL
PQDFKWLKCIVYKEKV----SPSDGFWQEVATRIPINPS--QFRILNPLIVKETALDM-L
PLDFKWLKQMVYKEKV----RGMEGFWKSVAQYVPRAPA--DMRILNPYFIQEAAFQF-I
PLDFKWLRQMVYKEKL----VRLEGFWKSVARYVPREPS--EIRILNPYFIQEAAFQF-I
PLDFKWLRQMVFKEKL----KGTEGFWKSVAHYVPREPT--EIRILNPYFIQEAAFQF-I
PQDFKWLKYIVYKEKV----SPSDGFWKSVATAVPKEPK--DIRILNPYFIREAAFMF-I
WQDFKWLKYIVYKEKV----SAADGFWKSVATRVPREPH--EIRILNPYFIQEAAFSF-I
WQDFKWLKYIVYKEKV----SASDGFWKSVATRVPREPH--EIRILNPYFIQEAAFSF-I
WQDFKWLKYIVYKERV----SASDGFWKSVATRVPKEPP--EIRILNPYFIQEAAFTL-I
WQDFKWLKYIVYKERV----SASDGFWKSVATRVPKEPP--EIRILNPYFIQEAAFTL-I
WQDFKWLKYIVYKEKV----SASDGFWKSVATHVPREPH--EIRILNPYFIQEAAFSF-I
WQDFKWLKYIVYKEKV----SASDGFWKSVATHVPREPH--EIRILNPYFIQEAAFSF-I
WQDFKWLKYIVYKEKV----SASDGFWKSVATHVPREPH--EIRILNPYFIQEAAFSF-I
WQDFKWLKYIVYKEKV----SASDGFWKSVATRVPREPH--EIRILNPYFIQEAAFSF-I
WQDFKWLKYIVYKEKV----SASDGFWKSVATRVPREPH--EIRILNPYFIQEAAFSF-I
WQDFKWLKYIVYKEKV----SASDGFWKSVATRVPREPH--EIRILNPYFIQEAAFSF-I
ALDFKWLRHMVFNQRL----HSTDGFWKSVARHVPRDPG--DMRILNPYFIQEASFKL-I
SNDFKWLRHMVYKDRL----WRMDGFWKSVARVVPRAPQ--DMRILNPYFIQEASFRL-I
TRDLQWLLGVLNHHRV-----GPRGFWKKVPTELQFPAS--RIRILNPAFVREAATEF-L
VLDLQWLKSILNNEKR-----PAKGFWKKPPILLDVKAS--NLRILNPYFMEVTASKL-L
PLDIQWMKIILNNEKR-----VRKGFWKMPPIIWEVEPE--NIRILNPYYMSVTATQI-L
NQSIHYYEKVMLKAMR-----VYRGFWKRPPIIWKSNPE--YVRILNPFFMRMTATRL-L
AMDFHWIETILSDKKR-----VRKGFWKQPPLIWDVNPK--QIRILNPFFMEIAADKL-L
AMDFHWIETILSDKKR-----VRKGFWKQPPLIWDVNPK--QVRILNPFFMEIAADKL-L
PVDFQWMEAILSDRKR-----VRKGFWKQPPLIWDANPE--QVRILNPYYMEVTAAKL-L
PMDFQWMEAILNDKKR-----VRKGFWKQPPLIWDANPE--QVRILNPYYMEVTAAKL-L
PMDFLWMETILHDKKR-----IRKGFWKQPPLIWDANPE--QVRILNPYFMEVTAAKL-L
PMDFLWMETILHDKKR-----IRKGFWKQPPLIWDANPE--QVRILNPYFMEVTAAKL-L
ENDLRWLKSNLYNEQR---VQIVQGLWKSAPIVWNVSPT--KIRILHPYMNLKAGTEL-L
VGDIQWLKTILYHEPA------VQGFWKLPPSVFRTNSS--NFRLLNPYYMHQAAKDF-L
PDDLRWLKEILYDEKR-----VRKGFWKPPPLIWLGQSS--KVRVLDPYFMQQTANKL-L
QQDLRWLKEILYDEKR-----VSKGFWKPPPQIWLGRAS--QIRVLDPYFLRITARKF-L
QQDLRWLKEILYNEKR-----VRRGFWRPPPQVWLGDVS--KIRVLDPHFLHQTAEML-L
---------------EGKGRWASAGALSVVWALHICNEVDVFGFGA---NKYGNWDHYYE
---------------DRKGRYPSSGTLAVFFALQFCDEVSVYGMGA---NSKGFWDHYWE
---------------EHHGRYSSTGSLVILFAVHVCDEVDVYGYGA---DKLGNWNHYWT
---------------QHHGRYPSTGLIAIIYALHECDQVDLYGFGA---DSAGNWHHYWE
---------------QHHGRYPSTGLLALIYALHECDEVDVYGFGA---NRAGNWHHYWE
---------------ELHGRYPSTGMLVLLFAMHICDQVNVFGFGA---TSKGNWDHYYQ
---------------ERHGRYPSTGIVAIIFALHLCDEVSVFGYGA---DAQGNWHHYWE
---------------NRHGKYPSTGIMCIVFALHVCDQVDVYGFGA---DANGNWHHYFE
---------------LNHGRYPSTGLLAVIFALHVCDKVDLYGFGA---DSKGHWHHYWE
---------------LNHGRYPSTGLLAVIFALHVCDKVDLYGFGA---DSKGHWHHYWE
---------------QAHGRYPSTGILSVILSLHICDKVDLYGFGA---DSKGNWHHYWE
---------------QGHGRYPSTGILSVIFSLHICDEVDLYGFGA---DSKGNWHHYWE
---------------QGHGRYPSTGILSVIFSMHVCDEVDLYGFGA---DSKGNWHHYWE
---------------QGHGRYPSTGILSVIFSLHVCDEVDLYGFGA---DSKGNWHHYWE
```

```
---------------QNHGRYPSTGLLSVIFALHVCDEVNVYGFGA---DSKGHWHHYWE
---------------EHHGRYPSTGLLSLMFALHVCDEVNVYGFGA---DSKGHWHHYWE
---------------QRHGRYPSTGFLSIIFALHLCDEVDLYGFGA---DSKGNWHHYWE
---------------QNHGRYPSTGILSVIFALHLCDEVDVYGFGA---DSNGNWHHYWE
---------------QHHGRYPSTGFLSVIFALHLCDEVDLYGFGA---DSKGNWHHYWE
---------------EHHGRYPSTGMTALVFALHICDEVSVFGYGA---DSNGNWHHYWE
---------------KHHGRYPSTGFTALIFAIHTCQQVSAFGFGA---DNNGNWHHYWE
---------------KYHGRYPSTGLIALIFAIHTCTQVSAFGFGA---NSNGNWHHYWE
---------------QHHGRYPSTGLTALLFALHICDQVSVRVGGA---DSRGNWHHYWE
---------------ERHGRYPSTGFTALLFALHACQQVSVFGFGA---DSKGNWHHYWE
---------------QHHGRYPSTGFTALLFALHACQQVSVFGFGA---DTKGNWHHYWE
---------------EHHGRYPSTGMLVLFFALHVCDEVDVYGFGA---DSRGNWHHYWE
---------------KKHGRYPSTGMLTLIFALHICDEVNLYGFGA---DSRGNWHHYWE
---------------RHHGRYPSTGMLVLFFALHVCDEVNVFGFGA---DSRGNWHHYWE
---------------RHHGRYPSTGMLVLFFALHVCDEVNVFGFGA---DGRGNWHHYWE
---------------EHHGRYPSTGMVALFFALHVCDEVDVYGFGA---DSRGNWHHYWE
---------------EHHGRYPSTGMLVLFFALHVCDEVDVYGFGA---DSRGNWHHYWE
---------------EHHGRYPSTGMLVLFFALHVCDEVNVFGFGA---DSRGNWHHYWE
---------------EHHGRYPSTGMLVLFFALHVCDEVNVYGFGA---DSRGNWHHYWE
---------------EHHGRYPSTGMLVLFFALHVCDE-NVFGFGA---DSRGNWHHYWE
---------------EHHGRYPSTGMLVLFFALHVCDEVNVFGFGA---DSHGNWHHYWE
---------------EHHGRYPSTGMLVLFFALHVCDEVNVFGFGA---DSRGNWHHYWE
---------------EHHGRYPSTGMLVLFFALHVCDEVNVFGFGA---DSRGNWHHYWE
---------------EHHGRYPSTGMLVLFFALHVCDEVNVFGFGA---DSRGNWHHYWE
---------------EHHGRYPSTGMLVLFFALHVCDEVNVYGFGA---DSRGNWHHYWE
---------------EHHGRYDTHGCPAPFLHCHTSPQVNVYGFGA---DSRGNWHHYWE
---------------EHHGRYPSTGMLVLFFALHVCDEVNVYGFGA---DSRGNWHHYWE
---------------EHHGRYPSTGMLVLFFALHVCDEVNVYGFGA---DSRGNWHHYWE
---------------EHHGRYPSTGMLVLFFALHVCDEVNVYGFGA---DSRGNWHHYWE
---------------NHHGKYPSTGMLALVFALHICDEISVFGFGA---DQHGNWHHYWE
---------------NHHGKYPSTGMLALVFALHICDEISVFGFGA---DQHGNWHHYWE
---------------NHHGKYPSTGMLALVFALHICDEISVFGFGA---DQHGNWHHYWE
---------------VHHGKYPSTGMLALFFAIHICDEVSVFGYGA---DSNGDWHHYWE
---------------NHHGKYPSTGMIAILFALHICEEVSVFGFGA---DRKGSWHHYWE
---------------VKHGKYPSTGFLAIIFALHICDQVSTFGFGA---DQYGNWYHYFE
---------------EGRGHYPSTGFLTLMFALHICDEVSVFGFGA---DQHGNWHHYWE
---------------EGHGRYPSTGFLSLLLAVHICDKVSVFGFGA---DQYGNWHHYWE
---------------DGHGRYPSTGFLSLLLAIHICDQVSVFGFGA---DHYGNWHHYWE
---------------QKHGRYPSTGFITLIFALHICDQVSVFGFGA---DKDGNWHHYFE
---------------RKHGSYPSTGFITLIFALHICDQVSVFGFGA---KLDGNWHHYFD
---------------EGHGGYPSTGFLTLIFALHICDEVNVFGFGA---TSEGQWHHYFD
KNNVSTS--------MKTHQKPSCGIVGIVMALHYCDEVDVFGFGA---NKYGNWDHYYE
KNNVSTS--------MKTHQKPSCGIVGIVMALHICNEVDVFGFGA---NKYGNWDHYYE
---------------RLQGKWPTTGLIATTFALHYCDVVDLAGFGY----GSSIFSHYYE
---------------VGMINIPTIGTISIITAFHFCDMVDIVGFGF----RHDKLYHYYE
---------------VGMINIPTIGTISIITAFHFCDMVDIVGFGL----RHDKLYHYYE
TYAEQ----------QRKVMVPTLGITAVVVALQVCDEVSIAGFGY-DLQHPGAPLHYYG
RYTL-----------TRGKMVPTLGASAVVMALQVCDQVSLAGFGY-DMQHPQSQLHYYE
KYTL-----------KQGNMNPTLGASALVMAMQLCDQVSLAGFGY-NMQHPEARLHYYE
SYPDPHSSQW-----KWMVNIPTLGVSAVVLATHLCDEVSLAGFGY-NLGEPDTPLHYYE
GFPKPQKRWL-----GWDKNVPTIGMTAVILATHLCDEVNLAGFGY-DLSQPDASLHYYD
QYPEPQNKWW-----GWEKNIPTLGVTAVVLATHLCDEVSLAGFGY-DLRQPRAPLHYYD
HYSEPQPRIW-----GWSKNVPTMGVVAVVLATHLCDEVSLAGFGY-DLSRPKTPLHYFD
QYPEPRSKIW-----GWDKNVPTIGVTAVVLATHLCDEVSLVGFGY-DLSQPNTPLHYFD
QYSEPQSRFW-----GRDKNVPTIGVIAVVLATHLCDEVSLAGFGY-DLNQPRTPLHYFD
QYSEPQSRFW-----GHDKNIPTIGVIAVVLATHLCDEVSLAGFGY-DLSQPRTPLHYFD
EYPEPRPKFW-----GWDKNIPTIGVTAIVLATHLCDEVSLAGFGY-DLSQPNTPLHYYD
HFPEPRSKFW-----GWDKNVPTIGVTAVVLATHLCDEVSLAGFGY-DLDQPSTPLHYYN
EFPKPRSIFW-----GWDKNVPTIGVMAVVLATHLCDEVSIAGFGY-DLNQPSTPLHYYN
NYPEPKKRLW-----SWDQNIPTLGLTALNLATYICDEVSLAGFGY-NLSQKEAPLHYYD
QFPPPRSQLW-----GNQQNVPTIGMSALNLASLLCDEVSLAGFGY-NFSVNSARLHYYD
QLNASMP--------HKGKRYATTGIIALNLALHLCQEVNIAGFGYPGNHDTTTPIHYYN
KLR------------KSSQKWSTTGFIALNLALHICHEVSIVGFGYPDINDNVTPVHYYK
KLPMIDP--------LQPPQYPTTGIIAISMALTMCNEVHVAGFKY-DFINMNSTLHYYS
HFPKVFPK-------NQKPKHPTTGIIAITLAFYICHEVHLAGFKY-NFSDLKSPLHYYG
RFPRVFPK-------DQKPKHPTTGIIAITMAFHICSEVHLAGFKY-NFYSPNSPLHYYG
HLPTKFSR-------HERPRHPTTGLIAITFALQICSEVHLAGFKY-NLNDRNSSLHYYG
HFPTRFPK-------KEKPKHPTTGLIAITLAFHICHEVHLAGFKY-DFTDRNSSLHYYG
HFPTRFPK-------KEKPKHPTTGLIAITLAFHICHEVHLAGFKY-DFTDRNSSLHYYG
RFPTRFPK-------KEKPKHPTTGLIAITLAFHICHEVHLAGFKY-DFTDRNSSLHYYG
HFPKRFPK-------KEKPKHPTTGIIGITFALHICNEVHIAGFKY-NLTSQNSSLHYYG
NFPFSFPK-------TEKPKHPTTGIIAITLAFHICNEVHIAGFKY-NLTSLNSSLHYYG
GYQLNDG--------KANKNIPTSGMLAIMMGVRLCDEVAVAGFGY-DPNKPGAALHYYD
GMKSG----------KGSKVIPTTGAMVIMVAIRVCDEVSVAGFGY-DMKRLDTPIHYYS
GMKSGKGFID-----IHLQNVPTTGAMVIMVAIRVCDEVSVAGFGY-DMKRLDTPIHYYS
GFPTMGG--------KMGKNVPTIGSLAIIWAINYCDEVTVAGFGY-DLSQPTVWLHYYK
GFPTMGG--------KMGKNVPTIGSLAIIWAINYCDEVTVAGFGY-DLSQPTVWLHYYK
GYPTNLG--------KMNKNVPTSGTISIMLALRLCDEVDVVGFGY-NTKEPEALIHYYE
QYPFNNGL-------LGRENIPTLGTVAITMALHNCDEVAVAGFGY-DLNNPNAPLHYYE
GLPFNNGL-------MGKGNIPTLGTVAITMALHNCDEVAVAGFGY-DMNMPYAPLHYYE
GLPQNNGL-------MGKGNIPTLGTVAITMALHNCDEVAVAGFGY-DMNTPHAPLHYYE
GLPFNNGL-------MGKGNIPTLGTVAITMALHNCDEVAVAGFGY-DMNTPHAPLHYYE
GLPVN----------NGMMGRPTLGTVAITMALHNCDEVAVAGFGY-DMNTPNAPLHYYE
GLPFNNGL-------MGRGNIPTLGSVAITMALHNCDEVAVAGFGY-DMNLPNAPLHYYE
GLPFNNGL-------MGRGNIPTLGSVAITMALHNCDEVAVAGFGY-DMNLPNAPLHYYE
GLPFNNGL-------MGRGNIPTLGSVAVTMALHGCDEVAVAGFGY-DMSTPNAPLHYYE
GLPFNNGL-------MGRGNIPTLGSVAVTMALHGCDEVAVAGFGY-DMNTPNAPLHYYE
GLPFNNGL-------MGRGNIPTLGSVAITMVLHNCDEVAVAGFGY-DMNSPNAPLHYYE
GLPFNNGL-------MGRGNIPTLGSVAITMVLHNCDEVAVAGFGY-DMNSPNAPLHYYE
GLPFNNGL-------MGRGNIPTLGSVAITMVLHNCDEVAVAGFGY-DMNSPNAPLHYYE
```

```
GLPFNNGL-------MGRGNIPTLGSVAITMALHNCDEVAVAGLGY-DMSSPNAPLHYYE
GLPFNNGL-------MGRGNIPTLGSVAITMALHNCDEVAVAGFGY-DMSSPNAPLHYYE
GLPFNNGL-------MGRGNIPTLGSVAITMALHNCDEVAVAGFGY-DMSSPNAPLHYYE
GLPHNRGQ-------MGRGNIPTLGAVAITMALHNCDEVAVAGFGY-NMSTPHAPLHYYE
GLPHNNGL-------MGRGNIPTLGTVAITMALHNCDEVAVAGFGY-DMNTPHAPLHYYE
NISSDTKSGKV----GTRLRCPTTGVIAITLALHLCDEVHLAGFGY-EVNSPGSPVHYYG
NVSVKPS--------KKVTQKPTSGLLAITFALHFCDVVHIAGFGYPELSNKKMPIHYYE
KSKKMI---------PKMIPKPTTGLLAITFALHFCDMVHIAGFGYPALTNKKQPIHYYE
GLPNKPS--------QKVRQKPTTGLVAITLALHLCNMVHIAGFGYPDTSNKNQSIHYYE
SLPMQQP--------RKIKQKPTTGLLAITLALHLCDLVHIAGFGYPDAYNKKQTIHYYE
SLPIQQP--------RKIKQKPTTGLLAITLALHLCDLVHIAGFGYPDASNKKQTIHYYE
SLPMKQP--------RKVKQKPTTGLLAITLALHFCDLVHIAGFGYPDSANKKQTIHYYE
SLPMKQP--------RKVKQKPTTGLLAITLALHFCDLVHIAGFGYPDSANKKQTIHYYE
NIPMKQL--------RKFKQKPTTGLVAITLALHFCDMVDIAGFGYPDSANKKQSIHYYE
NIPMKQL--------RKFKQKPTTGLVAITLALHFCDMVDIAGFGYPDSANKKQSIHYYE
GSSLEVSLKLVLSTFHQRPTRPTIGFLAITAALHYCDVVHIAGFGYPKQHDKLQLVHYYD
SLPL-----------SQRGHQPTTGFLAIIMALNYCDQVHIAGFGY-PFNDLNGLIHYYD
QFPLAPKK-------GQNHFHPTTGLLAVFVALNYCDMVHIAGFGYPPKSDKRQPIHYYG
Q--------------IPVQVHPTTGLLAVFVALNYCDVVHVAGFGYPASRNQNQPIHYYG
HMRQQIKG-------RPNLVHPTTGILAVIVALNYCDEVHIAGFGYPNSKRESQPIHYFG
KFSSK---EKDPFRR------TGVHDANIEE-----------------------------
VNDGS---RNSAFLK------THVHDSVHEFEVIKKLADEKIIT--MFQGVR--------
THYSG-----AAHRS------TGVHDSEFERVLLEKLQSEGIIK--IHRGNAANK-----
DLPPH---IAGAFRQ------TGVHDSDKESSMINQLHIHRLLT--LHVPGKDL------
DLPPH---VAGAFRK------TGVHDSAQENEIIDQLHIHGLLR--VHRSEQSS------
PPVEPSLEKDSAFKL------TGVHNAEFETDIVKQLEEIGKIT--VYRGSRV-------
YNRYA-----GAFRK------TGVHNADFETEIIQRLSAEGKIK--LYR-----------
NTTKP-----SGFRK------SGVHDADFQANITENLSSINKIN--FFTGR---------
NNASA-----GAFRL------TGVHDGDFEASILANLTSINKVL--MFRGR---------
NNASA-----GAFRL------TGVHDGDFEASILANLTSINKVL--MFRGR---------
NNPSA-----GAFRK------TGVHDGDFESNVTSTLASLNKIR--IFKGR---------
NNPSA-----GAFRK------TGVHDGDFESNVTATLASIHKIR--IFKGR---------
NNPSA-----GAFRK------TGVHDADFESNVTATLASINKIR--IFKGR---------
NNPSA-----GAFRK------TGVHDGDFESNVTATLASINKIR--IFKGR---------
NNASA-----GAFRQ------TGVHDGDFEFNVTLTLASIEKIK--FFKGR---------
NNTSG-----GAFRK------TGVHDGDFEFNITLTLASIEKIK--FFKGR---------
NNPSA-----GAFRQ------TGVHDGDFESNVTHTLASVAKVR--LFKGR---------
NNASG-----GAFRK------TMVHDGDFESNITLTLASIDKIR--FFKGR---------
NNPSA-----GAFRQ------TGVHDGDFESNITLTLASIDKIR--FFKGR---------
NNRNG-----GAFRR------TGVHSGDFESQIIKKLADEGKII--FYK-----------
ENHYA-----GAFRK------TGVHDANFELGLIERLADEGKVL--FYK-----------
DNRYG-----GAFRK------TGVHNATFELQLIERLAIEGKLS--FYK-----------
ANRYP-----GAFRK------TGVHNAGFELTLIKKLAAEGKIS--FYN-----------
ENRWS-----GAFRR------TRVHDADVEFSLIQRLAAEGRIL--FYQ-----------
KNRWS-----GAFRR------TGVHDADTEFSLIERLAAEGRIL--FHR-----------
NNRYA-----GEFRK------TGVHDADFEAHIIDSLVQIGKIR--VYRGK---------
NNQHA-----GAFRA------TMVHDGDFEANITRALASIKKVN--IFKG----------
QNRYS-----GEFRK------TGVHDADYEAQIIDKLAKAGKIS--VFPGK---------
QNRYS-----GEFRK------TGVHDADYEAQIIQRLAKAGKIT--VFPGK---------
NNRYS-----GEFRK------TGVHDADHEEKIIKQLTEAGKIS--VHR-----------
NNRYA-----GEFRK------TGVHDADFEAHIIDSLVQIGKIR--VYRGK---------
NNRYA-----GEFRK------TGVHDADFEAQIIDNLAKEGKIK--VYRST---------
NNRYA-----GEFRK------TGYDDADFEAQIISLLAASGKIQ--VYSGK---------
NNRYA-----GEFRK------TGVHDADFEAHVVGVLEKTGKID--VYRGN---------
NNRYA-----GEFRK------TGVHDADFEAHIVDILEKTNKIS--VYRGN---------
NNRYA-----GEFRK------TGVHDADFEAHIIDMLAKTSRIE--GVPGQ---------
NNRYA-----GEFRK------TGVHDADFEAHIIDMLAKTSKIE--VYRGN---------
NNRYA-----GEFRK------TGVHDADFEAHIIDMLAKTSKIQ--VYRGN---------
NNRYA-----GEFRK------TGVHDADFEAHIIDMLAKASKIE--VYRGN---------
NNRYA-----GEFRK------TGVHDADFEAHIIDMLAKASKIE--VYRGN---------
NNRYA-----GEFRK------TGVHDADFEAHIIDMLAKASKIE--VYRGN---------
NNRYA-----GEFRK------TGVHDADFEAHIIDMLAKASKIE--VYRGN---------
NNRYA-----GEFRK------TGVHDADFEAHIIDMLAKASKIE--VYRGN---------
RNRFA-----GAFRW------TGVHNAEFESDLIAKLAEEGRLK--LYK-----------
RNRFA-----GAFRW------TGVHNADFESELITKLAEEGRLK--LYK-----------
RNRFA-----GAFRW------TGVHNAEFESELITELVEEGRLK--LYK-----------
RNRFA-----GAFRR------TRVHNAEFELDLIRQLAYEGKVT--YH------------
NNKHA-----GAFQK------TGVHDAKFEMEIIRKLVEEGKIT--FYNETKVKY-----
KTSSR--------VR------TGAHSGSFEFDTMMQLYLESKIR--VFRGR---------
HNQLA-----GAFRH------TGVHDGDYEYNLTLTLADKHKIQ--MFRGR---------
ENSLA-----GAFRH------TGVHDGDYEYNVTLLLADKHKIQ--MFKGR---------
ENSLA-----GAFRH------TGVHDGDYEYNVTLLLADKHKIQ--MFKGR---------
HKKHP--------RN------AGNHGGSYEYLIALKLHEKQGIR--LYKGW---------
ESLAH-------FNR-------GRHGGDYENRTIHKLLLMNKIA--LHKGL---------
DSMTS-------F--------SNLHGGDFENQTIHFLHQQNKIS--LHKGWT--------
KFSSK---EKDPFRR------TGVHDANIEETVRMELHKEKIIR--FHPGNPA-------
KFSSK---EKDPFRR------TGVHDANIEETVRMELHKEKIIR--FHPGNPA-------
RDKTG------GLPAG-----GTQHNVTVEKEYLIRLLDEGVISHDLTGVYKNSSKTRSS
NKTIE-----GEFEG-LFSYFTLIHDFAKEKEYIKDLVRNAVVN-DLTHGYED-------
NKTIQ-----GEFEG-LFSYFTVIHDFAKEKEYIKDLVRNAVIN-DLTHGYED-------
SLRMD------AMKT------QVVHDVSAETVFLKELVKAGAVR-DLTGAL---------
AVPMD------AMKA------QVVHDIGAEKLFLRDLVAAGATT-DLTGAL---------
AIRMD------AMKA------QVVHDVSAEKLFLRDLVAAGAVT-DLTGAL---------
NVRME------AMKA------QTMHNVETERKFLAGLVEKGVVT-DLSGGI---------
NRCMN------SMND------QPMHDVTKEKKMLRTLVKEGVVR-DLSGGIHCAFCDTQH
DLCMS------EMKA------QKMHDVTKETKLLQKLIKEGVVK-DITGGI---------
NLCMA------AMNI------QTMHNLTTETRFLQKLVKEGIVK-DLSGGIHCEF-----
TLCMA------AMNG------QTMHNVTRETLFLQKLVQEEVVK-DLSGGIHCDFCKKES
SQCMA------AMNF------QTMHNVTTETKFLLKLVKEGVVK-DLSGGIDREF-----
SQCMG------AMHW------QVMHNVTTETKFLLKLLKEGVVE-DLSGGIH--------
```

```
     NLCMA------AMNE------QTMHNVTTETKFLQKLIKEGVVK-DLSGGIFCTYCKKSS
     NLCMA------AMKE------QTMHNVTSETKFLQKLIKEKIVK-DLTGGIYCEF-----
     NLCMA------AMNR------QTMHNVTGETKLLQKLVKEKVVK-DLTGGIH--------
     SVPMT------TILK------EAMHNVQKETVFLKRLVASGSIT-DLTGGIHCSFC----
     KLPMR------VMQQ------QTSHNVKEETAFLQKLIQDGAVT-DLTGGIHCSFCPR--
     TGLSR-------KNE------LFQHNLTAERNWLLKMIEWGVIA-DLTSH----------
     NDYVS------SL-R------LMPHDIPTEQMWLLKLVEKGLIS-DLSLT----------
     NDTMS------SMGK-------------------------------------------
     NATMS------LMNK------NAYHNVTAEQLFLKDIIEKNLVI-NLTQD----------
     NATMS------LMKQ------NAYHNLTAEQLFLNDIIKKKMVI-NLTQN----------
     NDTMS------QMMK------NAYHDIAVEQRFLKELIDLKILI-NLT------------
     NETMS------QMMQ------NEYHNITAEQKFLKKLIDKNFVV-NLT------------
     NETMS------QMMQ------NEYHDISAEQKFLKKLIDKNFVV-NLT------------
     NETMS------QMMQ------NEYHDISAEQKFLKKLIDKNFVV-NLT------------
     NDTMF------SMTK------TEYHNITAE-QMLLFIEKNNTIV----------------
     NETMS------VMAQ------NEYHNISAEQMFIRDLIEHKTIK-NLT------------
     SLPMR------EIWK------SWTHDIHHERGILRLLKQQGIIT-DLSGGI---------
     SQKTK------AILS------SFTHDIDHETKFLHKLVEQGVIN-DITGGLRTKL-----
     SQKTK------AILS------SFTHDIDHETKFLHKLVEQGVI-----------------
     DAKMS------SIAK------SWTHDINKEKEFLKTLVRNGVIT-DLTGGIIGHV-----
     DAKMS------SIAK------SWTHDINKEKEFLKTLVRNGVIT-DLTGGIIGHV-----
     KVKMK------IITS------LFTHDINHETAFLKKLVEF--------------------
     SIKML------AIKE------SWTHNIGREKEFLRKLVKGQVIS-DLTNGI---------
     TVRMS------TIKE------SWTHNIPKEKEFLRKLVKANVIT-DLTNGI---------
     SVKMS------AIKE------SWTHNISKEKEFLRKLVKANIIT-DLTNGI---------
     TFKMS------AIKE------SWTHNIAKEKEFLRKLVRANVIT-DLTNGI---------
     TIKMS------AIKE-------------------------------------------
     TIKMA------AIKE------SWTHNIQKEKEFLKKLVKARVIN-DLTNGI---------
     TIKMA------AIKE------SWTHNIQKEKEFLKKLVKARVIN-DLTNGI---------
     TVRMA------AIKE------SWTHNIQREKEFLRKLVKARVIT-DLSSGI---------
     TVRMA------AIKE------SWTHSIQREKEFLRKLVKARVIT-DLSSGI---------
     NIKMS------AIKE------SWTHNIQREKEFLRKLVKARVIT-DLTSGI---------
     TIKMS------AIKE------SWTHNIQREKEFLRKLVKARVIT-DLTSGI---------
     TIKMS------AIKE------SWTHNIQREKEFLRKLVKARVIT-DLTSGI---------
     NIKMS------AIKE------SWTHNIQREKEFLRKLVKARVIT-DLTSGI---------
     NIKMS------AIKE------SWTHNIQREKEFLRKLVKARVIT-DLTSGI---------
     NIKMS------AIKE------SWTHNIQREKEFLRKLVKARVIT-DLTSGI---------
     KIRMA------AIKE------SWTHNILREKEFLLKLVKAGVIQ-DLTNGI---------
     KLRMS------AIKE------SWTHNISREKEFLRKLVKAGVIE-DLTHGI---------
     QDTMS------SIIK------STYHNITIERTFLKKLKEGEAIV-DLTQLAGT-------
     KATLK------SM-A------VSDHNIS-------------------------------
     KVTLK------SMF-------ASEHNITVEAQAIKNLFQQNIIH-NLTYF----------
     KVMLK------TMRP------SG-HNISQEALAIKRLLDIKAIK-NLTYF----------
     QITLK------SMAG------SG-HNVSQEALAIKRMLEMGAIK-NLTSF----------
     QITLK------SMAG------SG-HNVSQEAIAIKRMLEMGAVK-NLTYF----------
     QITLK------SM-A------ASEHNISHEAVAIKRMLELGLVK-NLTYF----------
     QITLK------SM-A------ASEHNISHEAVAIKRMLELGLVK-NLTYF----------
     QITVK------SMAT------SG-HNVSHEASAIKKMLVMGLIK-NLTYF----------
     QITVK------SMAV------SG-HNVSHEAFAIKKMLELGLVK-NLTYF----------
     SSTML------------------------------------VM---------------
     QRNMK---------------------------------TMSVS----------------
     YHTMK------SM-K------NSPHDVSREGRALKRLEDSGAVM-YLH------------
     QQTMK------SMFQ------NSYHDLNQEAQILHRLEEQGVIL-YLHPHS---------
     RDTMK------SM-Q------NSYHDLGHEAEALKRLQDSGAIL-KLH------------
50
Skow_ST6GAL_02430        VCTLR-TA-A----LKILDRQTSPFNKLG-LSRYLPN-----GSAFLEM-----KTYKWC
Skow_ST6GAL_02428        VCKFKLSA-N----ASVLDKSTRPYQDLG-LSKYFPK-----KNIFNAH----EKRYNTC
Skow_ST6GAL_02427        LCDLKRRA-K----YDVLKRDTTPFKEIG-ISKYFPE-----ISLYEKF-----KGFESC
Skow_ST6GAL_02429        ----------------MMGYDTTPFKEMG-ISKYFPG-----RNIYEKY-----N-YSRC
Dsim_ST6GAL_02400        TCLVMEA--G----VRVLRRKDAPFNKLP-FGRLFPR-----QKLFRN-----VKDIKTC
Dsec_ST6GAL_02401        TCLVMEA--G----VRVLRRKDAPFNKLP-FGRLFPR-----QKLFRN-----VKDIKTC
Aaeg_ST6GAL_02407        LCRLKRA--D----VRTLSWRDPPFNWNE-IGSYFPL-----NPLFKE-----R--NASC
Cqui_ST6GAL_02409        LCRLTRA--D----VRTVTWRDSPFDWNE-IGRYIPP-----GPLFRE-----R--NATC
Bflo_ST6GAL_02425        LCELKNRV-W----FKTLPSTEGPFKALN-YSLLEPK----NTLYQLH-----HDGFKTC
Bflo_ST6GAL_02426        LCSLQSA-AR----FRTLPADESPWKQLG-YGDIEAR----PGLLQLY-----AGGFRTC
Phumcor_ST6GAL_02412     KCRLKKSEL------RLLTKKDLSFKTLN-NLRNDKN---EKEEEFLE-----GMQYKTC
Apis_ST6GAL_02408        TCDMASSVEK----FRTLEKND---IDHM-LTKNIPD-----VPLFDD-----NEVFGTC
Tcas_ST6GAL_02413        MCELEKI--Q----VKTLKRGDVAGGPHN-LGDFLPK-----RGLFE------NRNFNSC
Bmor_ST6GAL_02414        MCALKRI--K----VNTVTKRDEPFAR---YDFQIPE-----GPLQD------GTFYNTC
Dpul_ST6GAL_02410        TPSMLLENL------QNSLEKSHFHTITS-LTTVFPKEKQLLKNSQAF-----QKRYKSC
Isca_ST6GAL_02411        VCSLSRLFP--------TPRDTEPFKRMG-YSRYFPT-----TNLY-------ESCYNSC
Lpol_ST6GAL_02415        VCSAKHDVPV-----SMLSADDTFFKEIG-LAPLFPN-----KNFNQV-----LGLYNSC
Pmar_ST6GAL_02423        LCLVAERV-TALRSTLTLGGSEPPFSAMGAWREALDA-----GGLERA--VSGGRSLERC
Cmil_ST6Gal2             LCELKKRV-R----LRTLDGNETPFSLLG-WKKYVPK-----IPLSKIKLKL--RVFRKC
Stro_ST6Gal2             LCVLKDRA-Q----VKTLDGKDAPFSSLG-WEKYFPK-----IALNKL--YP--HGFSTC
Oana_ST6Gal2             LCQLRDRV-H----VRTLDGKEAPFSALG-WDKHVPR-----TPLAKL--YP--RGFGSC
Mdom_ST6Gal2             LCELRDRV-S----VRTLDGKEAPFSDLG-WEKHVPQ-----EPLSKL--YP--QGLGSC
Acar_ST6Gal2             FCELKARV-N----IRTIDGKEAPFSILG-WEKHVPQ-----ISLNKL--YP--NGFGSC
Cpicbel_ST6GAL2          ICELKDRV-H----VKTIDGKEDPFSALG-WEKHVPQ-----IPLGKL--YP--HGFGSC
Ggal_ST6Gal2             FCELKERV-D----VKTIDGKEAPFSTLG-WEKHVPQ-----IPLGKL--YT--HGFGSC
Tgut_ST6Gal2             FCELKERV-N----VKTIDGKEAPFSTLG-WEKHVPQ-----IPLVKL--YT--HGFGSC
Btau_ST6Gal2             LCALRGRV-Q----VRTLDGTEPPFSALG-WRALVPP-----VPLSRL--LP--RRLRTC
Hsap_ST6Gal2             LCQLRSRA-R----VRTLDGTEAPFSALG-WRRLVPA-----VPLSQL--HP--RGLRSC
Ocun_ST6Gal2             LCELRRRA-Q----VRTLNGTEAPFAALG-WRRLVPA-----VPLSQL--HP--RGLRTC
Drer_ST6Gal2-r           LCQMKEQA-Q----IRTLNGTEQPFADLG-FKQLVSP-------------PLQQKYRTC
Drer_ST6Gal2             LCELKKRE-K----IRTLDGAEMPFSKLG-WQKIVPA-----LPLSQIHR----PGLKTC
Olat_ST6Gal2             LCQMKNQS-Q----LRTVDGSEQPFSSLG-WADFVPL-----VPLQRWNKQRGGRSFRTC
Alim_ST6GAL2             LCQMKAQA-Q----LRTLDGSEEPFSSLG-WVDFVPP-----LPLERLSRRQEKSSLKTC
Trub_ST6Gal2             LCEMKAQA-R----LQMVDGTQQPFSSLG-WASLVPS-----LPLEQLHKRPDQGSFKSC
Tnig_ST6Gal2             LCELQAQA-R----LQTVDGTEPPFSSLG-WARLVPA-----LPLDQLHQQPARGSFNSC
```

```
Locu_ST6GAL1           LCELKDKV-A----VTSLTPDLPPFSELS-WGSQLPP-----HLVTAH-----LGPYKSC
Pbiv_ST6GAL1           LCQLKNRV-N----VSTVLGNEGPFAEPE-WRGMLPQ-----RSLAED-----LGHLETC
Stro_ST6Gal1           LCAIKTKV-N----ISTLVTADLPQNADS-WSQYLPR-----KTLQEE-----VKSLGKC
Acar_ST6Gal1           LCQLRNRL-D----FRMITATDSPFDISE-WEPYVPK-----GNISI--------KLGRC
Gfor_ST6GAL1           LCQLRDRV-N----VTMIQRSDGPFGTSE-WLQYLPG-----KSFSEA-----VGHLGRC
Tgut_ST6Gal1           LCQLRDRV-N----VTMIQRSDGPFGTSE-WQQYLPG-----KSLSEA-----VGHLGCC
Svul_ST6GAL1           LCQLRDRV-N----VTMIQRSDGPFGTSE-WQQYLPG-----KSLSET-----VGHLGHC
Falb_ST6GAL1           LCQLRDRV-N----VTMIQRSDGPFGTSE-WQQYLPG-----KSLSET-----VGHLGRC
Ggal_ST6Gal1           LCRLRDRV-N----VTMIRGSDGPFNSSE-WQHYLPD-----KSLNET-----VGRLGRC
Fche_ST6GAL1           LCQLRDRV-N----VTMIQGSDGPFGTSE-WQQYLPG-----KSLNET-----VGSLGRC
Cliv_ST6GAL1           LCQLRDRV-N----VTMIEGSDGPFDTSE-WQQYIPG-----KSLNET-----VGRLGRC
Mdom_ST6Gal1           LCQLANRV-K----VTMIQGTDFPFNTSE-WEKSLPP-----KNIRES-----FGRLGTC
Btau_ST6Gal1           LCHLRDHV-N----ISMIEASDFPFNTSD-WEGYLPQ-----EDIRSK-----AGPWGRC
Hsap_ST6Gal1           RCHLRDHV-N----VSMVEVTDFPFNTSE-WEGYLPK-----ESIRTK-----AGPWGRC
Cfam_ST6Gal1           HCHLRDHV-N----VSMVEATDFPFNTSE-WEGFLPK-----ENIRTK-----AGPWGRC
Skow_ST6GAL_02430      AVIGSSYFLLGSKLGKEID--SHDAVLRFNDALV-TGFERDVGSKTTIRLINSQMLVK-K
Skow_ST6GAL_02428      AIVSSSSFMTDSGLGEEID--SHEAVMRFNNAPT-VGFERDVGNKTTMRVINLDIMLL-P
Skow_ST6GAL_02427      AIVASAAYFKNATLGKEID--SHDAVLRFNDAPT-EGFEENVGRKTTIRLMNTLIFEE-E
Skow_ST6GAL_02429      AVVLSSFYMNETGFGDEID--SHEAVLRLNDAPT-VGYEKSVGRKTTIRLLNSKCFND-S
Dsim_ST6GAL_02400      AIVSSAGSLAGSKLGRFID--THDIVMRFNHAPT-QGHEVDVGSKTTIRVVNSQVVTK-P
Dsec_ST6GAL_02401      AIVSSAGSLAGSKLGRFID--THDIVMRFNHAPT-QGHEVDVGSKTTIRVVNSQVVTK-P
Aaeg_ST6GAL_02407      AVIASAGSLKGSRLGDFID--DHDVVMRFNHAPT-KGFEVDVGSKTTVRVVNSQVVTK-K
Cqui_ST6GAL_02409      AIVSSAGSLKGSSLGSFID--GHDIVMRFNHAPT-VGYEHDVGSKTSVRVVNSQVVVK-P
Bflo_ST6GAL_02425      AAVMNSAAILGSNLGEEID--SHDAVMRFNNAPLSEEYRADIGVKTTLRLMNSQLLAD-P
Bflo_ST6GAL_02426      AAVMSSGAMLGSRLGKDIL-DSHDAVLRYNSAPT-KRYQSDVGSKTTLRLMNSQVVVKQS
Phumcor_ST6GAL_02412   AIVSSAGSLLNSKLGHLID--QHEIVVRFNHAPT-VTYEEDVGSKTTVRLVNSQVVSK-K
Apis_ST6GAL_02408      AIISNAATLRNSNLGYFID--QHDLVLRFNNAPT-KGYEKDVGSKTTIRILNSQVVTK-P
Tcas_ST6GAL_02413      AIVASSGALKDSNLGKKID--AHDLVLRFNHAPT-KGFEPDVGRKTTIRVLNSQVVTR-K
Bmor_ST6GAL_02414      AVVSSAGALLGSRLGDFID--SHDMVLRFNNAPT-DNYTDDVGSKTTFRILNSQVVSK-P
Dpul_ST6GAL_02410      AIVSSAGALLNSDLGTFID--GHDYVVRFNNAPT-LGFEVDVGNKTSLRILNSQILSK-S
Isca_ST6GAL_02411      ALVSSAGSLLGSRLGNEIGKYAHQAVLRFNDAPT-QGFEQDVGSRTTVRLLNSQLLSR-P
Lpol_ST6GAL_02415      AIVSSSGSMYKSGLGKEID--THDVVLRFNDAPT-KGYEKDVGNKTTVRILNSQVVSK-P
Pmar_ST6GAL_02423      AVVSSAGAMLGSGLGEID--AHDAVLRFNAAPT-EHFEKDVGTKTTIRLVNSQILAR-P
Cmil_ST6Gal2           AVVASAGAILNSSLGDEID--SHDAVMRFNAAPT-KLYEQDVGSKTTIRILNSQILAN-S
Stro_ST6Gal2           AVVSSAGAILNSSLGAEID--SHDAVLRFNSAPT-RNYEKDVGNKTTLRIINSQILTN-P
Oana_ST6Gal2           AVVMSAGAILNSSLGAEID--AHDAVLRFNSAPT-RGYEKDVGNKTSLRIINSQILTN-P
Mdom_ST6Gal2           AVVMSAGAILNSSLGEEID--SHDAVLRFNSAPT-HGYERDVGNKTTMRIINSQILTN-P
Acar_ST6Gal2           AVVMSAGAILNSSLGKEID--SHEAVLRFNSAPT-RGYEKDVGNKTTMRIINSQILTN-P
Cpicbel_ST6GAL2        AVVMSAGAILNSSLGDEID--SHDAVLRFNSAPT-RGYEKDVGNKTTMRIINSQILTN-P
Ggal_ST6Gal2           AVVMSAGAILNSSLGDEID--SHDAVLRFNSAPT-RGYEKDVGNKTTMRIINSQILTN-P
Tgut_ST6Gal2           AVVMSAGAILNSSLGDEID--SHDAVLRFNSAPT-RGYEKDVGNKTTMRIINSQILTN-P
Btau_ST6Gal2           AVVTSAGAILNSSLGEEID--SHDAVLRFNSAPT-RGYEKDVGNKTTVRIINSQILTN-P
Hsap_ST6Gal2           AVVMSAGAILNSSLGEEID--SHDAVLRFNSAPT-RGYEKDVGNKTTIRIINSQILTN-P
Ocun_ST6Gal2           AVVMSAGAILNSSLGEEID--SHDAVLRFNSAPT-RGYEKDVGNRTTMRIINSQILTN-P
Drer_ST6Gal2-r         AVVASAGAILNSSLGHEID--SHDAVLRFNAAPS-EGYERDVGSKTTIRIMNSQILAN-P
Drer_ST6Gal2           AVVTSAGAMLHSGLGKEID--SHDAVLRFNTAPT-VGYERDVGNKTTIRIINSQILAN-P
Olat_ST6Gal2           AVVSSAGAILHSGLGKEID--SHDAVLRFNAAPT-EGYEQDVGTKTTIRIINSQILAN-P
Alim_ST6GAL2           AVVTSAGAMLRSRLGKEID--SHDAVLRFNSAPT-DGYERDVGNTTTIRIINSQILAN-P
Trub_ST6Gal2           AVVTSAGAILRSGLGREID--AHDAVLRFNAAPT-EGYERDVGNKTTIRIINSQILAN-P
Tnig_ST6Gal2           AVVTSAGAILRSGLGKEID--SHDAVLRFNAAPT-EGYERDVGNKTTIRIINSQILAN-P
Locu_ST6GAL1           AVVSSAGSLKNSGLGREID--SHDAVLRFNAAPT-GGYEKDVGTKTTVRLINSQVMAS-E
Pbiv_ST6GAL1           AVVSSAGSMLGSGLGSEID--SHDAVLRFNGAPT-TGYEHDVGTKTTLRLVNSQLMAS-P
Stro_ST6Gal1           AVVSSAGSIKHSRLGQEID--THDAVLRFNAAPV-LGYQDDVGTKTTFRLLNSQLVSR-P
Acar_ST6Gal1           AVVSSAGSMKSSHLGEDID--SHDAVLRFNGAPI-KGFQADVGEKTTIRLVNSQLITV-E
Gfor_ST6GAL1           AVVSSAGSLKSSRLGQEID--SHDAVLRFNGAPV-RGFQDDVGQKTTIRLVNSQLVTV-E
Tgut_ST6Gal1           AVVSSAGSLKSSHLGQEID--SHDAVLRFNGAPV-RGFQDDVGQKTTIRLVNSQLVTV-E
Svul_ST6GAL1           AVVSSAGSLKSSRLGQEID--SHDAVLRFNGAPV-RGFQDDVGQKTTIRLVNSQLVTV-E
Falb_ST6GAL1           AVVSSAGSLKSSRLGHEID--SHDAVLRFNGAPV-RGFQDDVGQKTTIRLVNSQLVTV-E
Ggal_ST6Gal1           AVVSSAGSLKSSHLGPEID--SHDAVLRFNGAPV-KGFQEDVGQKTTIRLVNSQLVTV-E
Fche_ST6GAL1           AVVSSAGSLKSSHLGQEID--THDAVLRFNGAPI-SGFQEDVGQKTTIRLVNSQLVTV-E
Cliv_ST6GAL1           AVVSSAGSLKSSHLGQEID--SHDAVLRFNGAPI-KGFQEDVGQKTTIRLVNSQLVTV-E
Mdom_ST6Gal1           AVVSSAGSLKASYLGTEID--NHDAVLRFNGAPT-KKYEEHVGKKTTFRLVNSQLVTT-D
Btau_ST6Gal1           AVVSSAGSLKSSRLGREID--DHDAVLRFNGAPT-VKFQQDVGTKTTIRLVNSQLVTT-E
Hsap_ST6Gal1           AVVSSAGSLKSSQLGREID--DHDAVLRFNGAPT-ANFQQDVGTKTTIRLMNSQLVTT-E
Cfam_ST6Gal1           AVVSSAGSLKSSQLGREID--DHDAVLRFNGAPT-ASFQQDVGTKTTIRLMNSQLVTT-E
Skow_ST6GAL_02430      KKNASLLKALNVNSTLFVWKAGGRDENL-MKWYTV---SKDMFNNYIKWCIHRPEQCPYV
Skow_ST6GAL_02428      DF---YSNYIFNNVSLVAWYQAPYTFTP-DKWYEH--NGAAFYEKYIERRISRPEEKMYV
Skow_ST6GAL_02427      RF-GFETKDIFRNITLLVWKSGPYNGNL-YRFYSLYKDSKPFFDKYIQWREAHPWESFYI
Skow_ST6GAL_02429      TF-INDIEAMFVNTTLVTWKSGPYNGNL-YKWYQR-ESVKELLAKYIRWRMVQPSQDFYV
Dsim_ST6GAL_02400      EF-DFTRAPIFRNVTIAAWDPGKYNGTL-EDWLTS--ADYDLFTNYELYRRRYPKSRAFL
Dsec_ST6GAL_02401      EF-DFTRAPIFRNVTIAAWDPGKYNGTL-EDWLTS--ADYDLFTNYELYRRRYPKSRAFL
Aaeg_ST6GAL_02407      EF-KLLTAKHFRHVSIAAWDPGKFDQSL-EEWIKS--PDFNLFENFKKYREKYPGSNFHL
Cqui_ST6GAL_02409      EF-ELLTASLFRNVSIAAWDPGKFNQSL-KEWIET--PDFNLFENFKQYHQKYPGANFHL
Bflo_ST6GAL_02425      SF-NFFTDPIYRNITMIVWDPAAYTGDM-EEWYQN--PDANFFSSYEQRRRMFPRENLYI
Bflo_ST6GAL_02426      KY-HWFTDPRYRNVTMVLWDPMSYGGTMAEQWYKK--PDFNLFPAYEKRRRESPWEHVYI
Phumcor_ST6GAL_02412   EF-NFTTSSLYKKNVTLVVWDPSNYSATL-QEWYEK--PDFNFFERSEI-----------
Apis_ST6GAL_02408      QF-QFVSSPLYKRLKLLMWDPSNYTSSI-NEWITN--PEHNFIDNYISFRKSNPRSNFHI
Tcas_ST6GAL_02413      EF-NFLESDIYKNVTIVAWDPSNYKASL-DDWLEK--PEFNLFPTYVEYKKRNDKARFFL
Bmor_ST6GAL_02414      EY-KFLEDPLYKGVSIIIWDPANYSSTL-EEWYNH--PDFPLFPVYKKFLEETPNADAHL
Dpul_ST6GAL_02410      QF-NFVNSSLYKNVSLLVWDPSKYNATL-EQWSCN--PDFPFLDAFFSRRNQIPEEELHL
Isca_ST6GAL_02411      EF-DFNSPMFRNLTLIAWDPSRQGQSL-EQWVKK--PDFDLFPGYWLRREVLPEEPFYL
Lpol_ST6GAL_02415      EF-DFSHSPLYENMLLIVWDPPGYEDDI-KKWYQH--PDFDFFPFYWERRQRLPEENFFV
Pmar_ST6GAL_02423      QN-NFSRSTLYRDVTLLVWDPAPYSLNL-TQWFHD--PDYNFFPAFLRRRQRHPTQPAFV
Cmil_ST6Gal2           KH-NFINNALYKNIILVVWDPAPYSINL-IKWYKK--PDYNLFTPYLRYRRRNPAQPFYI
Stro_ST6Gal2           NH-HFTDSSLYKDVTLIAWDPSPYYADL-HMWYHK--PDYNLFPPYEKHRKRNPDQPFYI
Oana_ST6Gal2           SH-HFIDNSLYKDVILIAWDPAPYSANL-NVWYKK--PDYNLFTPYVQHRRRYPSQPFYI
Mdom_ST6Gal2           NH-HFIDSSLYKDVILVAWDPAPYSANL-HMWYKK--PDYNLFTPYIQHRRKNPNQPFYI
Acar_ST6Gal2           NH-HFIENSLYKDVILVAWDPAPYSANL-NVWFKK--PDYNLFTPYVQHRRKNPNQPFYI
Cpicbel_ST6GAL2        NH-HFIDSSLYKDVILVAWDPAPYSANL-NVWYKK--PDYNLFTPYVQHRRRNPNQPFYI
Ggal_ST6Gal2           NH-HFVDSSLYKDVILVAWDPAPYSANL--NWYKK--PDYNLFTPYVQHRKKNPNQPFYI
```

Tgut_ST6Gal2	NH-HFVDSSLYKDVILVAWDPAPYSANL-SQWYKK--PDYNLFTPYVQHRRKNPTQPFYI
Btau_ST6Gal2	SY-HFMDSALYKDVILVAWDPAPYSANL-NLRYKK--PDYNLFTPYVQHRQRNPNQPFYI
Hsap_ST6Gal2	SH-HFIDSSLYKDVILVAWDPAPYSANL-NLWYKK--PDYNLFTPYIQHRQRNPNQPFYI
Ocun_ST6Gal2	NH-HFVDSSLYKDVILVAWDPAPYSANL-NLWYKK--PDYNLFTPYIQHRQRNPNQPFYI
Drer_ST6Gal2-r	KY-RFGSSLLYKNITLVAWDPAPYNIDL-HKWFLH--PDYDLFTPYITHRKAFPEQPFYI
Drer_ST6Gal2	MH-RFNRSSLYKNVTLVAWDPAPYTLNL-HQWYSN--PDYNLFTPYMEYRMRFPSQPFYI
Olat_ST6Gal2	KH-EFKTSSIYKNITLVAWDPAPYTLNL-DEWFAS--PDYDLFGPYVEHRKNHAEQLFYI
Alim_ST6GAL2	TY-QFNTSSIYKNVTLVAWDPTPYSVNL-HKWYTS--PDYDLFGPYVDHRKVHPDQPFYI
Trub_ST6Gal2	NH-RFNTSSLYKDVVLVAWDPAPYTLDL-HKWYAS--PDYNLFGPYMEHRRAHPDQPFYI
Tnig_ST6Gal2	NH-RFNSSSLYKDVVLVAWDPAPYTVDL-HQWFAS--PDYNLFGPYVEHRRPHPEQPFYI
Locu_ST6GAL1	DH-HFLSSSLYSAGTLVAWDPAPFSSNL-AEWYNK--TDYPIFKQYQKYRLLHPQQPFYI
Pbiv_ST6GAL1	EQ-HFLDGQLYAQGTLVAWDPAPYPGDL-EEWYKT--PDYPLFEPFRTLRANRPWQLFHL
Stro_ST6Gal1	EL-KFLEDPLYKEGSLIMWDPAPYNANV-EEWHKN--PDYKFFERYSEYRRRNPEQMFYV
Acar_ST6Gal1	EK-KFLADPQYNFGTLILWDPAPYHSSI-KEWYKK--PDYNFYGSFKHYRKKYPKQPFYI
Gfor_ST6GAL1	EQ-QFLSEPLYNTGILIVWDPAPYHAEI-HEWYRK--PDYNFYESYKAYRRAHPEQPFYI
Tgut_ST6Gal1	EQ-QFLREPLYNTGILIVWDPAPYHAEI-REWYRK--PDYNFFESYKAYRRAHPEQPFYI
Svul_ST6GAL1	EQ-QFLRESLYNTGILIVWDPAPYHAEI-HEWYRK--PDYNFFESYKAYRRTHPEQPFYI
Falb_ST6GAL1	EQ-QFLREPLYNTGILIVWDPAPYHAEI-HEWYRK--PDYNFFESYKAYRRTHPEQPFYI
Ggal_ST6Gal1	EQ-QFLKDALYNTGILIVWDPAPYHAEI-HEWYRK--PDYKFFEAYKSYRIRHPEQPFYI
Fche_ST6GAL1	EQ-QFLREALYNTGILIVWDPAPYHAEI-HEWYRK--PDYNFFESYKSYRSAHPEQPFYI
Cliv_ST6GAL1	EQ-QFLREELYNTGILIVWDPAPYHAEI-HEWYRK--PDYDFFESYKLYRSIHPEQPFYI
Mdom_ST6Gal1	-K-NFFDDSLYKNGILIMWDPSPYHSEI-PEWYKK--PDYNFFHNYKLYRKLNPNQPFYI
Btau_ST6Gal1	-A-GFLKDSLYNEGILIVWDPSVYHSDI-PKWYRN--PDYSFFNNFKSYRKLHPDQPFYI
Hsap_ST6Gal1	-K-RFLKDSLYNEGILIVWDPSVYHSDI-PKWYQN--PDYNFFNNYKTYRKLHPNQPFYI
Cfam_ST6Gal1	-G-RFLKDSLYNEGILIVWDPSVYHSDI-PKWYQS--PDYSFFENYKSYRKLHPDQPFYI
Skow_ST6GAL_02430	INPSVLWKCWDIIQRYTK-ERMKNIVPTTGFTGIHLMHHICESIDVYGYVTP--TNTNCH
Skow_ST6GAL_02428	INPAWHWNIWDILQEYSE-ENINKHVPSSGFTGIILMQQICDQVNVYGFVTP--KTRLLH
Skow_ST6GAL_02427	MDPTPIWRTWDIIQENTPNITIRKTLPSSGIMGISLMQRMCDTVNVYGYLIPNGDPPLCH
Skow_ST6GAL_02429	INPTSLWDIWDDIMYFNKGKPIKKIVPSSGFTGLLMMLGLCDKVDVYGFGIN--DVSSCH
Dsim_ST6GAL_02400	IDPHSVWRLWQSLQMFAGNRPISRNPPSSGFIGLALLLPHCPQVDFVEYVPSTRLNGRCH
Dsec_ST6GAL_02401	IDPHSVWRLWQSLQMFAGNRPISRNPPSSGFIGLALLLPHCPQVDFVEYVPSTRLNGRCH
Aaeg_ST6GAL_02407	VDPRSIWRAWTALQDKT-HTKIMRNPPTSGFIGLGLLIPACRYIDMIEYIPSSRMNGLCH
Cqui_ST6GAL_02409	VDPRSIWRAWSALQDKT-KVKIIRNPPTSGFIGLGLLLPVCRYVHMIEYIPSSRMNGLCH
Bflo_ST6GAL_02425	TDPRFYWELWDVLQENT-EAMIHPNPPSSGFQGIAVMLSLCDQLDVYEFVPSERISMKCH
Bflo_ST6GAL_02426	MDPRYLWELWEVLHRNT-REKIVRNVPSSGFMGIHLLLSLCDTVDVYEYVPSKRETQRCH
Phumcor_ST6GAL_02412	-----HMESLGFFTKLT-SMRIRKNPPSSGFLGLALLMPYCDTIDFFEFVPSTRLTKKCH
Apis_ST6GAL_02408	VHPQYLWRLWDYIQDHT-TAHIRRNPPSSGFLGLAMLLPRCTVVNMFEFIPSERMTHRCH
Tcas_ST6GAL_02413	INPQSLWELWDFLQDNS-PSRLRRNPLSSGFLGLGILLPLCNFIDVFEYVPSTRVTKRCH
Bmor_ST6GAL_02414	LNPQVLWSLWAVLQNVS-PYRLRRNPPSSGFIGLWFSLNRCRRVRVFEYVPSTRASRRCH
Dpul_ST6GAL_02410	MDPRGLWNLWDYLSLHS-PLPIPPNPPSSGFIGLALTLSFCDYVDMIEFVPSLRMTKRCH
Isca_ST6GAL_02411	LHPDSVWEAWHFLDREN-PGPVVRNPPSSGFLGLLLLLGLCRRLDAYELVPSMRLTKRCH
Lpol_ST6GAL_02415	VHPTVIWKAWNFIQENT-AVPIKKNPPSSGFLGLLLLLQHCNTVDIYEYIPSMRLTKRCH
Pmar_ST6GAL_02423	LHPAYLWRIWELLQDSA-SEHIQPNPPSSGFLGVVLMMWLCDEVNVYEFLPSRRRTDLCH
Cmil_ST6Gal2	LHPRFLWQLWDIIQENT-QEKIQPNPPSSGFIGIVIMMALCDSINIYEYIPSIRQTDLCH
Stro_ST6Gal2	LHPKFTWELWKIIQENS-NEKIQPNPPSSGFIGILIMMSMCRTVHVYEYIPSYRQTDLCH
Oana_ST6Gal2	LHPKFIWQLWDIIQENT-REKIQPNPPSSGFIGILIMMSMCREVHVYEYIPSVRQTDLCH
Mdom_ST6Gal2	LHPKFIWQLWDIIQENT-KEKIQPNPPSSGFIGILIMMSLCKEVHVYEYIPSVRQTDLCH
Acar_ST6Gal2	LHPKFIWQLWDIIQENT-KEEIQPNPPSSGFIGILIMMSMCHEVHVYEYIPSVRQTDLCH
Cpicbel_ST6GAL2	LHPKFIWQLWDIIQENT-KEKIQPNPPSSGFIGILIMMSMCNEVHVYEYIPSVRQTDLCH
Ggal_ST6Gal2	LHPKFIWQLWDIIQENT-KEKIQPNPPSSGFIGILIMMSMCNEVHVYEYIPSVRQTDLCH
Tgut_ST6Gal2	LHPKFIWQLWDIIQENT-KEKIQPNPPSSGFIGSLIMMSMCNEVHVYEYIPSVRQTDLCH
Btau_ST6Gal2	LHPKFIWQLWDIIQENT-KEKIQPNPPSSGFIGILLMMNLCGEVHVYEYVPSVRQTDLCH
Hsap_ST6Gal2	LHPKFIWQLWDIIQENT-KEKIQPNPPSSGFIGILIMMSMCREVHVYEYIPSVRQTELCH
Ocun_ST6Gal2	LHPKFIWQLWDIIQENT-KEKIQPNPPSSGFIGILIMMSLCREVHVYEYIPSIRQTELCH
Drer_ST6Gal2-r	LHPSFIWKLWDIVQSIT-EENIQPNPPSSGFIGIIMMNTLCEKIHVYEYIPSMRQTDLCH
Drer_ST6Gal2	LHPKYIWQLWDVIQANN-LENIQPNPPSSGFIGILLMMSLCEEVHVYEYIPSLRQTDLCH
Olat_ST6Gal2	LHPSYLWQLWDLIQSNT-QEKIQPNPPSSGFIGILTMMALCDKLHVYEYIPSMRQTDLCH
Alim_ST6GAL2	LHPSYVWKLWDLIQSNT-QENIQPNPPSSGFIGILLMMALCEQVHVYEYIPSMRQTDLCH
Trub_ST6Gal2	LHPRYVWRLWDVIQGNT-QENIQPNPPSSGFIGILLMMTLCEQVHVYEYIPSMRQSDLCH
Tnig_ST6Gal2	LHPSYVWRLWNVIQGNT-QENIQPNPPSSGFIGILLMMTLCEQVHVYEYIPSVRQSDLCH
Locu_ST6GAL1	LHPKVEWQLWERIQENM-AEPIQKNPPSSGLLGTVLMMSVCEVVHLYEFLPSRRKTELCH
Pbiv_ST6GAL1	LHPRLQWELWQLVQEGA-DEQVQRNPPSSGLLGTVLMMSLCELVHVYEFLPSQRQSHQCH
Stro_ST6Gal1	LNPHAAWQLWDIIQQNA-PEDINPGPPSSGLLGILLMMNLCDQVNVYEFLPSRRQSDLCH
Acar_ST6Gal1	LNPHMQWQLWDILQENS-PEDIQPNPPSSGMLGKLIMMNFCDEVDVYEYLPSKRQTNICH
Gfor_ST6GAL1	LNPKMQWQLWDILQENS-LEHIQPNPPSSGMLGIVLMMTLCDQVDVYEFLPSKRQTDICH
Tgut_ST6Gal1	LNPKMQWQLWDILQENS-LEHIQPNPPSSGMPGIVLMMTLCDQVDVYEFLPSKRQTDICH
Svul_ST6GAL1	LNPKMQWQLWDILQENS-LEHIQPNPPSSGMLGIVLMMTLCDEVDVYEFLPSKRQTDICH
Falb_ST6GAL1	LNPKMQWQLWDILQENS-LEHIQPNPPSSGMLGIVLMMTLCDQVDVYEFLPSKRQTDICH
Ggal_ST6Gal1	LNPKMQWQLWDILQENS-LEHIQPNPPSSGMLGIVIMMTLCDEVDVYEFLPSKRQTDICH
Fche_ST6GAL1	LNPKMQWQLWDILQENS-LEHIQPNPPSSGMLGIVIMMTLCDEVDVYEFLPSKRQTDICH
Cliv_ST6GAL1	LNPKMQWQLWDILQENS-LEPIQPNPPSSGMLGIVIMMTLCDEVHVYEFLPSKRQTDICH
Mdom_ST6Gal1	LNPKMPWELWDILQENS-PEDIQPNPPSSGMIGIVIMMNFCDTVDIYEFLPSKRKTDICH
Btau_ST6Gal1	LKPQMPWELWDIIQEIS-SELIQPNPPSSGMLGIAIMMSLCDQVDIYEFLPSKRKTDVCY
Hsap_ST6Gal1	LKPQMPWELWDILQEIS-PEEIQPNPPSSGMLGIIIMMTLCDQVDIYEFLPSKRKTDVCY
Cfam_ST6Gal1	LKPQMPWELWDIIQEVS-PEEIQPNPPSSGMLGIIIMMTLCDQVDIYEFLPSKRKTDVCY
Skow_ST6GAL_02430	YYDV-HKCP---RLAWHPVEAEKNFLKTLHVG--NEN-DLSVKGKLSLYGFHHD--CVTC
Skow_ST6GAL_02428	YYSN-VLINAPTGYLWHPMVQEKRIAKIMNRG--PEE-EITNHGKITLPGFNQM--HCL-
Skow_ST6GAL_02427	YYDG-MC----SKATWHPVEYEKKLVRHINFG--STD-DILKHGRMTVPGFRTF--HCD-
Skow_ST6GAL_02429	YYDK-PGCNS-ASKRWHPLQEKA-IMAAMHDG--PIS-DL-DKHVVTVNGFKHI--NCNG
Dsim_ST6GAL_02400	YYSK-EMNSACTFGSWHPLAAEKLMALDMNMAEDDDM-SVFQFGILRIRRPDKL--LCGF
Dsec_ST6GAL_02401	YYSK-EMNSACTFGSWHPLAAEKLMALDMNMAEDDDM-SVFQFGILRIRRPDKL--LCGF
Aaeg_ST6GAL_02407	YYDS-EINSACTFGSWHPLAAEKLYVLQMNTA--DEF-TTFQRGVVRISLDSNS--GC--
Cqui_ST6GAL_02409	YYDD-EINSACTFGSWHPLAAEKLYVLRMNTA--TDF-TTFQHGIVRIGLDGPT--PSAD
Bflo_ST6GAL_02425	YYEKWKLNPACTLGWWHDLRAEKYFILRMNQG--TKD-DITKKGKVTLSGYRAN--DCKA
Bflo_ST6GAL_02426	YYGE-EMDIACTYGAYHPLRAEKELIKKLNIG--PEE-DVRNKGKITLPGFGSI--QC--
Phumcor_ST6GAL_02412	YFED-FEDDSCTFGVWHPLSAEKILSLAINEM--NDS-VVFNTGFIRIKGYKDL--KC--
Apis_ST6GAL_02408	YYHE-KVDVTCTFGIWHPLAAEKLLMLTANTM--PDQ-TVFHTGFLSIPGYKSP--ICTS
Tcas_ST6GAL_02413	YYDP-EDNPACTFGVWHPLAAEKLLTYYINTI--DDR-TVFQDGYVRILGFKNF--KC--
Bmor_ST6GAL_02414	YYAH-RDDVACTFGAWHPLAQEKALAETLRDN--SDI-DTFQRGFIDITGLATI--QC--
Dpul_ST6GAL_02410	YYDT-NEDVGCTMGDWHPLAAEKLLVLSLNPA--DDH-VIFVDGYVRITGFSSQ--LQQE

```
Isca_ST6GAL_02411         YYEV-HEDLGCTLGDWHPLAAEKLLALHMAAP--EVLDQVFAQGRLPLGGGSSA--AP--
Lpol_ST6GAL_02415         YYDV-HENLGCTLGDWHPLASEKLLSLAMNAA--SDL-QVFVNGKISIKGDVKC------
Pmar_ST6GAL_02423         YHED-YTDRACTEGAYHPLLYEKNFVKRLSTS--SDH-DLFWAGRATLPGLRRAMESCPP
Cmil_ST6Gal2              YHER-YYDSACTLGAYHPLLYEKLLVQRMNKG--TEA-DLYSKGRVSLPGFHSI--KCAG
Stro_ST6Gal2              YHEQ-YYDAACTLGAYHPLLYEKMLIQRINQG--TED-NLLRKGKVILPGFSSI--HCPI
Oana_ST6Gal2              YHEL-YYDAACTLGAYHPLLYEKLLVQRMNRG--ERD-DLYRKGKVILPGFRAL--RCPA
Mdom_ST6Gal2              YHEL-YYDAACTLGAYHPLLYEKLLVQRMNQG--IRD-DLYRKGKAILPGFRAI--SCPA
Acar_ST6Gal2              YHEL-YYDAACTLGAYHPLLYEKLLVQRMNKG--FQD-DLYRKGKVILPGFRSV--KCPG
Cpicbel_ST6GAL2           YHEL-YYDAACTLGAYHPLLYEKLLVQRMNKG--LQD-DLYRKGKVILPGFRSV--KCPG
Ggal_ST6Gal2              YHEL-YYDAACTLGAYHPLLYEKLLVQRMNKG--LQD-DLYRKGKVILPGFKSV--KCPE
Tgut_ST6Gal2              YHEL-YYDAACTLGAYHPLLYEKLLVQRMNKG--LQD-DLYRKGKVILPGFKAI--KCPK
Btau_ST6Gal2              YHEP-YHDAACTLGAYHPLLYEKLLVQRLNVG--THG-DLHRKGKVVLPGLQAV--RCPP
Hsap_ST6Gal2              YHEL-YYDAACTLGAYHPLLYEKLLVQRLNMG--TQG-DLHRKGKVVLPGFQAV--HCPA
Ocun_ST6Gal2              YHEL-YYDAACTLGAYHPLLYEKLLVQRLNMG--TQG-DLHRKGKVVLPGFQAV--QCPA
Drer_ST6Gal2-r            YHEL-QYDMACTFGAYHPLLYEKLLVKRMSTA--SEE-DLRKKGKVTLPGFSKI--KCLL
Drer_ST6Gal2              YHER-YYDAACTLGAYHPLLYEKMLIQRMNIG--SED-ELKRKGKVTLPGFNKV--HCEP
Olat_ST6Gal2              YHEN-YYDAACTLGAYHPLIYEKNLIRRINLG--SEK-DLLKKGRVTLPGFSTL--TCGA
Alim_ST6GAL2              YHER-YYDAACTLGAYHPLLYEKSLIQRINTG--PEN-DLRRKGRVTLPGFSTV--DCDI
Trub_ST6Gal2              YHER-YYDAACTLGAYHPLLYEKSLIQRINTG--PGS-DLRRKGRVTLPGFSTV--DCDI
Tnig_ST6Gal2              YHER-YYDAACTLGAYHPLLYEKSLIQRINTG--PES-DLRKKGRVTLPGFSAV--NCDI
Locu_ST6GAL1              YYQR-FFDAACTLGAYHPLMYEKNLVKRMNRG--TDK-EIYQYGRVTLPGLATL--NCTA
Pbiv_ST6GAL1              YYQD-FSDDACTLGAYHPLLFEKNLVRRMNRG--TQA-DLAQRGRVTLPGFQVL--NCTQ
Stro_ST6Gal1              YYER-YQDRACTLGAYHPLMYEKNLVKKLNLG--DDN-SIFHFGRITLPGLRGL--QC--
Acar_ST6Gal1              YYQK-IFDQACTMGAYHPLLFEKNIIKHLNIG--KDE-DIYNYGKATLPGLRNV--QC--
Gfor_ST6GAL1              YYQK-FHDHACTMGAYHPLLFEKNLVKHMNQG--TDE-DIYTLGKVTLPGFRKV--Q---
Tgut_ST6Gal1              YYQK-FHDHACTMGAYHPLLFEKNLVKHMNQG--TDE-DIYTHGKVTLPGFRKV--HC--
Svul_ST6GAL1              YYQK-FHDHACTMGAYHPLLFEKNLVKHMNQG--TDE-DIYTHGKVTLPGFRKV--HC--
Falb_ST6GAL1              YYQK-FHDHACTMGAYHPLLFEKNLVKHMNQG--TDE-DIYTHGKVTLPGFRQV--QC--
Ggal_ST6Gal1              YYQK-FHDHACTMGAYHPLLFEKNLVKHLNQG--TDE-DIYTHGKVTLPGFRNV--HC--
Fche_ST6GAL1              YYQK-FHDRACTMGAYHPLLFEKNLVKHINQG--TDE-DIYTHGKVTLPGFRKV--HC--
Cliv_ST6GAL1              YYQK-FHDRACTMGAYHPLLFEKNLVKHINQG--TDE-DIYMHGKVTLPGFRNV--HC--
Mdom_ST6Gal1              YYQQ-FFDSACTMGAYHPLLFEKNMVKHLNQG--TDE-DIYLKGKVTVPGLQGV--RC--
Btau_ST6Gal1              YYQR-YFDSACTMGAYHPLLFEKNMVKYLNLG--TDE-DIYLLGKATLPGFRTI--RCGA
Hsap_ST6Gal1              YYQK-FFDSACTMGAYHPLLYEKNLVKHLNQG--TDE-DIYLLGKATLPGFRTI--HC--
Cfam_ST6Gal1              YYQK-FFDSACTMGAYHPLLFEKNLVKHLNQG--TDE-DIYLLGKATLPGFRRI--RC—
```

Data S2. 27 vertebrate ST6Gal sequences used in Figure 5: poorly aligned regions in the N-terminal part of ST6Gal sequences were manually removed and multiple sequence alignment obtained with clustal Omega at the http://www.ebi.ac.uk/Tools/msa/clustalo/ web site. Output format is Phylip/interleaved. 91 vertebrate ST3Gal sequences used in Figure 5: N-terminal part were manually removed and multiple sequence alignment obtained with clustal omega at the http://www.ebi.ac.uk/Tools/msa/clustalo/ web site. Output format is Phylip/interleaved.

```
31 237
ST6Gal2_Daniorerio CAVVTSAGAM LHSGLGKEID SHDAVLRFNT APTVGYERDV GNKTTIRIIN SQILANPMHR FNRSSLYKNV TLVAWDPAPY
ST6Gal2_Olatipes   CAVVSSAGAI LHSGLGKEID SHDAVLRFNA APTEGYEQDV GTKTTIRIIN SQILANPKHE FKTSSIYKNI TLVAWDPAPY
ST6Gal2_Alligmissi CAVVTSAGAM LRSRLGKEID SHDAVLRFNS APTDGYERDV GNTTTIRIIN SQILANPTYQ FNTSSIYKNV TLVAWDPTPY
ST6Gal2_Takirubri  CAVVTSAGAI LRSGLGREID AHDAVLRFNA APTEGYERDV GNKTTIRIIN SQILANPNHR FNTSSLYKDV VLVAWDPAPY
ST6Gal2_Calomili   CAVVASAGAI LNSSLGDEID SHDAVMRFNA APTKLYEQDV GSKTTIRILN SQILANSKHN FINNALYKNI ILVVWDPAPY
ST6Gal2_Silutropi  CAVVSSAGAI LNSSLGAEID SHDAVLRFNS APTRNYEKDV GNKTTLRIIN SQILTNPNHH FTDSSLYKDV TLIAWDPSPY
ST6Gal2_Monoddomes CAVVMSAGAI LNSSLGEEID SHDAVLRFNS APTHGYERDV GNKTTMRIIN SQILTNPNHH FIDSSLYKDV ILVAWDPAPY
ST6Gal2_Anolcarol  CAVVMSAGAI LNSSLGKEID SHEAVLRFNS APTRGYEKDV GNKTTMRIIN SQILTNPNHH FIENSLYKDV ILVAWDPAPY
ST6Gal2_Chryspicta CAVVMSAGAI LNSSLGDEID SHDAVLRFNS APTRGYEKDV GNKTTMRIIN SQILTNPNHH FIDSSLYKDV ILVAWDPAPY
ST6Gal2_Taenopgut  CAVVMSAGAI LNSSLGDEID SHDAVLRFNS APTRGYEKDV GNKTTMRIIN SQILTNPNHH FVDSSLYKDV ILVAWDPAPY
ST6Gal2_Gallusgal  CAVVMSAGAI LNSSLGDEID SHDAVLRFNS APTRGYEKDV GNKTTMRIIN SQILTNPNHH FVDSSLYKDV ILVAWDPAPY
ST6Gal2_Struthcam  CAVVMSAGAI LNSSLGSEID SHDAVLRFNS APTRGYEKDV GNKTTMRIIN SQILTNPSHH FVDSSLYKDV ILVAWDPAPY
ST6Gal2_Bostaurus  CAVVTSAGAI LNSSLGEEID SHDAVLRFNS APTRGYEKDV GNKTTVRIIN SQILTNPSYH FMDSALYKDV ILVAWDPAPY
ST6Gal2_Homosap    CAVVMSAGAI LNSSLGEEID SHDAVLRFNS APTRGYEKDV GNKTTIRIIN SQILTNPSHH FIDSSLYKDV ILVAWDPAPY
ST6Gal2_Musmusc    CAVVMSAGAI LNSSLGEEID SHDAVLRFNS APTRGYEKDV GNKTTVRIIN SQILANPSHH FIDSALYKDV ILVAWDPAPY
ST6Gal1_Oryzlatip  CAVVSSAGSL RHSGLGKEID SHDAVMRFNA APTSGFEKDV GSKTTMRLIN SQVMASEEYR FLSSSLYSSG VLVAWDPAPF
ST6Gal1_Gasterost  CAVVSSAGSL RNSGLGKEID SHDAVLRFNA APTTGYEKDV GSKTTIRLIN SQVMASDDHR FLSSSLYSSG VLVAWDPAPF
ST6Gal1_Takirubri  CAVVTSAGSM RSSGLGKEID SHDAVLRFNA APTSGYENDV GSKTTIRLVN SQVMASEAHR FLSSSLYSSG TLVAWDPAPF
ST6Gal1_Amblymexi  CAVVSSAGSL RNSGLGKEID SHDAVVRFNA APTAGFEKDV GSKTTVRLIN SQVMASEDHH FLSSSLYSIG TLVAWDPAPY
ST6Gal1_Daniorerio CAVVSSAGSL KNSGLGKEID SHDAVIRFNA APTAGFETDV GSKTTVRLIN SQLMASEDHH FLSSSLYSAG ILVSWDPSPY
ST6Gal1_Silutropi  CAVVSSAGSI KHSRLGQEID THDAVLRFNA APVLGYQADV GTKTTFRLLN SQLVSRPELK FLEDPLYKEG SLIMWDPAPY
ST6Gal1_Alligmissi CAVVSSAGSL KSSHLGPEID GHDAVLRFNG APTKGFQQDV GEKTTIRLVN SQLVTVEEHN FLTESLYNTG ILIVWDPAPY
ST6Gal1_Taenopgut  CAVVSSAGSL KSSHLGQEID SHDAVLRFNG APVRGFQDDV GQKTTIRLVN SQLVTVEEQQ FLREPLYNTG ILIVWDPAPY
ST6Gal1_Gallusgal  CAVVSSAGSL KSSHLGPEID SHDAVLRFNG APVKGFQEDV GQKTTIRLVN SQLVTVEEQQ FLKDALYNTG ILIVWDPAPY
ST6Gal1_Struthcam  CAVVSSAGSL KSSHLGPEID SHDAVLRFNG APTRGFKEDV GEKTTVRLVN SQLVTVEEQQ FLTDALYNTG ILIVWDPAPY
ST6Gal1_Anolcarol  CAVVSSAGSM KSSHLGEDID SHDAVLRFNG APIKGFQADV GEKTTIRLVN SQLITVEEKK FLADPQYNFG TLILWDPAPY
ST6Gal1_Pythonmor  CAVVSSAGSM KQSHLGAEID SHDAVLRFNG APTKGFQVDV GEKTTLRLVN SQLITVEEKK FITDVQYNFG TIIVWDPSPY
ST6Gal1_Monoddomes CAVVSSAGSL KASYLGTEID NHDAVLRFNG APTKKYEEHV GKKTTFRLVN SQLVTTD-KN FFDDSLYKNG ILIMWDPSPY
ST6Gal1_Musmuscul  CAVVSSAGSL KNSQLGREID NHDAVLRFNG APTDNFQQDV GTKTTIRLVN SQLVTTE-KR FLKDSLYTEG ILILWDPSVY
ST6Gal1_Homosap    CAVVSSAGSL KSSQLGREID DHDAVLRFNG APTANFQQDV GTKTTIRLMN SQLVTTE-KR FLKDSLYNEG ILIVWDPSVY
ST6Gal1_Ovisaries  CAVVSSAGSL KSSRLGREID DHDAVLRFNG APTVKFQQDV GTKTTIRLVN SQLVTTE-AG FLKDSLYNEG ILIVWDPSVY
                   TLNLHQWYSN PDYNLFTPYM EYRMRFPSQP FYILHPKYIW QLWDVIQANN LENIQPNPPS SGFIGILLMM SLCEEVHVYE
                   TLNLDEWFAS PDYDLFGPYV EHRKNHAEQL FYILHPSYLW QLWDLIQSNT QEKIQPNPPS SGFIGILTMM ALCDKLHVYE
                   SVNLHKWYTS PDYDLFGPYV DHRKVHPDQP FYILHPSYVW KLWDLIQSNT QENIQPNPPS SGFIGILLMM ALCEQVHVYE
                   TLDLHKWYAS PDYNLFGPYM EHRRAHPDQP FYILHPRYVW RLWDVIQGNT QENIQPNPPS SGFIGILLMM TLCEQVHVYE
                   SINLIKWYKK PDYNLFTPYL RYRRRNPAQP FYILHPRFLW QLWDIIQENT QEKIQPNPPS SGFIGIVIMM ALCDSINIYE
                   YADLHMWYHK PDYNLFPPYE KHRKRNPDQP FYILHPKFTW ELWKIIQENS NEKIQPNPPS SGFIGILIMM SMCRTVHVYE
                   SANLHMWYKK PDYNLFTPYI QHRRKNPNQP FYILHPKFIW QLWDIIQENT KEKIQPNPPS SGFIGILIMM SLCKEVHVYE
                   SANLNVWFKK PDYNLFTPYV QHRRKNPNQP FYILHPKFIW QLWDIIQENT KEEIQPNPPS SGFIGILIMM SMCHEVHVYE
                   SANLNVWYKK PDYNLFTPYV QHRRRNPNQP FYILHPKFIW QLWDIIQENT KEKIQPNPPS SGFIGILIMM SMCNEVHVYE
                   SANLSQWYKK PDYNLFTPYV QHRRKNPTQP FYILHPKFIW QLWDIIQENT KEKIQPNPPS SGFIGSLIMM SMCNEVHVYE
```

```
          SANLN-WYKK PDYNLFTPYV QHRKKNPNQP FYILHPKFIW QLWDIIQENT KEKIQPNPPS SGFIGILIMM SMCNEVHVYE
          SANLNVWYKK PDYNLFTPYV QHRRKNPNQP FYILHPKFIW QLWDIIQENT KEKIQPNPPS SGFIGILIMM SMCSEVHVYE
          SANLNLRYKK PDYNLFTPYV QHRQRNPNQP FYILHPKFIW QLWDIIQENT KEKIQPNPPS SGFIGILLMM NLCGEVHVYE
          SANLNLWYKK PDYNLFTPYI QHRQRNPNQP FYILHPKFIW QLWDIIQENT KEKIQPNPPS SGFIGILIMM SMCREVHVYE
          SANLNLWYKK PDYNLFTPYI QHRRKYPTQP FYILHPKFIW QLWDIIQENT REKIQPNPPS SGFIGILIMM SMCKEVHVYE
          SSDLTQWLNR TDYPIFAQYQ RYRRLHPQQP FFILHPRFEW QVWQQVQENM AESIQKNPPS SGFLGTVLMM SLCEVVHVYE
          SADLTQWYNR TDYPIFTQYQ RYRKLHPMQP FYILHPRFEW QVWQRIQDNM AEPIQKNPPS SGLLGTVLMM SLCEVVHVYE
          SADLTQWFNR TDYPIFTQYQ RYRMLHPMQP FYILHPRFEW QVWQRIQDNM AEPIQKNPPS SGLLGTVMMM SLCEVVHVYE
          SSDLNEWYNK TDYPIFKQYQ RYRRLHPQQP FYILHPSVEW QLWKRIQDNM AEPIQKNPPS SGLLGTVLMM SLCDVVHVYE
          SSDLWEWFNK TDYPIFKQYQ RYRRLHPQQP FYIVHPRMEW QLWQRIQDNM GEAIQKNPPS SGLLGTVLMM SLCEVVHVYE
          NANVEEWHKN PDYKFFERYS EYRRRNPEQM FYVLNPHAAW QLWDIIQQNA PEDINPGPPS SGLLGILLMM NLCDQVNVYE
          HAEIHEWYRK LDYNFLETYK KYRSQHPEQL FYILNPKMEW ELWDILQENS LEDIQPNPPS SGMLGIIIMM TICDEVNVYE
          HAEIREWYRK PDYNFFESYK AYRRAHPEQP FYILNPKMQW QLWDILQENS LEHIQPNPPS SGMPGIVLMM TLCDQVDVYE
          HAEIHEWYRK PDYKFFEAYK SYRIRHPEQP FYILNPKMQW QLWDILQENS LEHIQPNPPS SGMLGIVIMM TLCDEVDVYE
          HADIHEWYRK PDYKFFENYK LYRIKHPEQP FYILNPKMQW QLWDILQENS LEHIQPNPPS SGMLGIVIMM TLCDEVHVYE
          HSSIKEWYKK PDYNFYGSFK HYRKKYPKQP FYILNPHMQW QLWDILQENS PEDIQPNPPS SGMLGKLIMM NFCDEVDVYE
          HANIDEWFRK PDYYFFESYK HYRKRHPKQP FYILNPFMQW QLWDILQENS PEDIQPNPPS SGMLGIVLMM NFCDEVDVYE
          HSEIPEWYKK PDYNFFHNYK LYRKLNPNQP FYILNPKMPW ELWDILQENS PEDIQPNPPS SGMIGIVIMM NFCDTVDIYE
          HADIPQWYQK PDYNFFETYK SYRRLHPSQP FYILKPQMPW ELWDIIQEIS PDLIQPNPPS SGMLGIIIMM TLCDQVDIYE
          HSDIPKWYQN PDYNFFNNYK TYRKLHPNQP FYILKPQMPW ELWDILQEIS PEEIQPNPPS SGMLGIIIMM TLCDQVDIYE
          HSDIPKWYRN PDYSFFNNFK SYRKLHPDQP FYILKPQMPW ELWDIIQEIS SELIQPNPPS SGMLGIAIMM SLCDQVDIYE
          YIPSLRQTDL CHYHERYYDA ACTLGAYHPL LYEKMLIQRM NIGSEDELKR KGKVTLPGFN KVHCEP---- -------
          YIPSMRQTDL CHYHENYYDA ACTLGAYHPL IYEKNLIRRI NLGSEKDLLK KGRVTLPGFS TLTCGA---- -------
          YIPSMRQTDL CHYHERYYDA ACTLGAYHPL LYEKSLIQRI NTGPENDLRR KGRVTLPGFS TVDCDI---- -------
          YIPSMRQSDL CHYHERYYDA ACTLGAYHPL LYEKSLIQRI NTGPGSDLRR KGRVTLPGFS TVDCDI---- -------
          YIPSIRQTDL CHYHERYYDS ACTLGAYHPL LYEKLLVQRM NKGTEADLYS KGRVSLPGFH SIKCAGEK-- -------
          YIPSYRQTDL CHYHEQYYDA ACTLGAYHPL LYEKMLIQRI NQGTEDNLLR KGKVILPGFS SIHCPIKDHI T------
          YIPSVRQTDL CHYHELYYDA ACTLGAYHPL LYEKLLVQRM NQGIRDDLYR KGKAILPGFR AISCPAPNHF PNP----
          YIPSVRQTDL CHYHELYYDA ACTLGAYHPL LYEKLLVQRM NKGFQDDLYR KGKVILPGFR SVKCPGHNRF PYT----
          YIPSVRQTDL CHYHELYYDA ACTLGAYHPL LYEKLLVQRM NKGLQDDLYR KGKVILPGFR SVKCPGQNHF PHL----
          YIPSVRQTDL CHYHELYYDA ACTLGAYHPL LYEKLLVQRM NKGLQDDLYR KGKVILPGFK AIKCPKRNNF PHL----
          YIPSVRQTDL CHYHELYYDA ACTLGAYHPL LYEKLLVQRM NKGLQDDLYR KGKVILPGFK SVKCPERNNF PPL----
          YIPSVRQTDL CHYHELYYDA ACTLGAYHPL LYEKLLVQRM NKGLQDDLYR KGKVILPGFK SVKCPERNNF PHL----
          YVPSVRQTDL CHYHEPYHDA ACTLGAYHPL LYEKLLVQRL NVGTHGDLHR KGKVVLPGLQ AVRCPPGA-- -------
          YIPSVRQTEL CHYHELYYDA ACTLGAYHPL LYEKLLVQRL NMGTQGDLHR KGKVVLPGFQ AVHCPAPSPV IPHS---
          YIPSVRQTEL CHYHELYYDA ACTLGAYHPL LYEKLLVQRL NTGTQADLHH KGKVVLPGFQ TLRCPVTSPN NTHS---
          FLPSKRKTEL CHYYQHFYDA ACTLGAYHPL LYEKNLVKRM NQGADRDIYT HGRVTLPGFN AINCTRAAQE PEGM---
          FLPSRRKTEL CHYYQRFHDA ACTLGAYHPL LYEKNLVKRM NRGPDRDIYT HGRVTLPGFG KMNCTEAAGG STSR---
          FLPSRRKTEL CHYYQRFFDA ACTLGAYHPL LYEKNLVKRM NQGPERDIYT HGRITLPGFN TLNCTGDAGG ALVDMRH
          FLPSRRKTEL CHYYQRFSDA ACTLGAYHPL LYEKNLVKRM NQGSDRDIYT HGRVTLPGFS TYNCTASILS KP-----
          FLPSRRKTEL CHYYQRFSDA ACTLGAYHPL LYEKNLVKRM NQGSDRDIYT LGRVTLPGFA TFNCTSSTHS KT-----
          FLPSRRQSDL CHYYERYQDR ACTLGAYHPL MYEKNLVKKL NLGDDNSIFH FGRITLPGLR GLQC------ -------
          FIPSKRQTDI CHYYQKFHDH ACTMGAYHPL MFEKNLVKYL NQGTDYNIYA HGKVTLPGFR NLRC------ -------
          FLPSKRQTDI CHYYQKFHDH ACTMGAYHPL LFEKNLVKHM NQGTDEDIYT HGKVTLPGFR KVHC------ -------
          FLPSKRQTDI CHYYQKFHDH ACTMGAYHPL LFEKNLVKHL NQGTDEDIYT HGKVTLPGFR NVHC------ -------
          FLPSKRQTDV CHYYQKFHDR ACTMGAYHPL LFEKNLVKHI NQGTDKDIYT HGKVTLPGFR NVHC------ -------
          YLPSKRQTNI CHYYQKIFDQ ACTMGAYHPL LFEKNIIKHL NIGKDEDIYN YGKATLPGLR NVQC------ -------
          FLPSKRQTDI CHYYQKFFDQ ACTLGAYHPL LFEKNMVKHL NQGTDEDIYN YGKVTLPGLR KAQC------ -------
          FLPSKRKTDI CHYYQQFFDS ACTMGAYHPL LFEKNMVKHL NQGTDEDIYL KGKVTVPGLQ GVRC------ -------
          FLPSKRKTDV CYYHQKFFDS ACTMGAYHPL LFEKNMVKHL NEGTDEDIYL FGKATLSGFR NNRC------ -------
          FLPSKRKTDV CYYYQKFFDS ACTMGAYHPL LYEKNLVKHL NQGTDEDIYL LGKATLPGFR TIHC------ -------
          FLPSKRKTDV CYYYQRYFDS ACTMGAYHPL LFEKNMVKHL NLGTDEDIYL LGKATLPGFR TIRCGA---- -------
91 276
Oscarcarm  CRRCAVVGTA GRLKGARQGK LIDSFDIVI- RMNRSPVKGY EVDVGSKTS- ------YHLV YPESAVGYRG AE--SSGKLV
Cionint12  CRSCAVVGNS GNILNSNYGN VIDGHDFVI- RMNKGPTYNY ENDVGSKTT- ------HRFM YPTTASSLPQ GKLFSGVSLV
BfloridA   CLRCAVVGNS GNLRQSNYGE EIDGYDLIF- RMNDAPTKGW EKDVGHRTT- ------HHFM YPESATDLP- ----DDVSFV
BfloridB   CLRCAVVGNS GNLRQANYGE EIDGYDLIF- RMNDAPTKGW EKDVGHRTT- ------HHFM YPESATDLP- ----DDVSFV
Saccogl12  CLQCAVVGNS GNLRDSRYGQ LIDSHDYIM- RMNTAKTVGF EKDVGSRTT- ------HHFM YPESFVEVI- ----GETKFV
Tgutta3g1  CRRCAVVGNS GNLRQSQYGP DIDSHDFVL- RMNRAPTAGY ESDVGGKTT- ------HHFV YPESYRELA- ----ANVSMI
Ggallus3g1 CRRCAVVGNS GNLRQSQYGQ DIDSHDFVL- RMNRAPTIGY ESDVGSKTT- ------HHFV YPESYKELA- ----ENVSMI
Strcamelg1 CRRCAVVGNS GNLRQSQYGH DIDSHDFVL- RMNRAPTAGF ESDVGSKTT- ------HHFV YPESYKELA- ----ENVSMI
Chrysemgl  CRRCAVVGNS GNLRQSQYGQ DIDAHDFVL- RMNRAPTAGF ESDVGSKTT- ------HHLV YPESFRDLA- ----ENVSMI
Acarolgal1 CRRCAVVGNS GNLKQSQYGQ EIDNHDFVF- RMNKAPTVGF ESDVGSKTT- ------HHFV YPESYKELG- ----ENVSMV
Pythongal1 CRRCAVVGNS GNLRRSQYGQ EI-------- RMNKAPTVGF EPDVGSKTT- ------HHFV YPESYRELG- ----EEVSMI
Hsapiensg1 CRRCAVVGNS GNLRESSYGP EIDSHDFVL- RMNKAPTAGF EADVGTKTT- ------HHLV YPESFRELG- ----DNVSMI
Oanatigall CRRCAVVGNS GNLKESRYGS DIDSHDFVL- RMNRAPTVGF EADVGSKTT- ------HHFM YPESFTELA- ----ENVSMI
Trubrigal1 CHTCAVVGNS GRLRQSGNGK IIDSHDSVI- RMNKAVTRGF AKDVGNRTT- ------HHFL YPESAVDVD- ----HGVSLV
Olatipgal1 CRSCAVVGNS GNLRESGHGE RIDSHSFVI- RMNKALTRGF EKDVGNRTT- ------HHFL YPESAVDVD- ----GGVSLV
Xenlaegal1 CRTCAVVGNS GNLKSSNYGP EIDEHDFVL- RMNHAPTARF EKDVGGKTT- ------HHFV YPESVRDLQ- ----ANVSMI
Acarolgal8 CGTCAVVGNS GRLKGSKYGE NIDSHHFVL- RINTAPTAGF EEDVGAKTT- ------HHFM YPESAMNLH- ----AGVHLM
Pythongal8 CGTCAVVGNS GRLRGSKYGQ KIDSHQFVM- RMNTAQIAGF QEDVGTRTT- ------HHFM YPESAVNLH- ----PGIHLV
Stropgal2A CRRCAVVGNS GNLKGSRHGK NIDSHGLIF- RMNGARTYGF EKDVGSKTT- ------HHFM YPESAVHVQ- ----PGVHLV
Chrysgal8  CRTCAVVGNS GRLRGSHYGR EIDSHHWVL- RMNRAQTAGF EEDVGTRTT- ------HRFM YPESAVDLQ- ----PGVHLV
Drerigal2A CRKCAVVGNS GNLLKSKYGA LIDSHSTVI- RMNKAVTVGY DEDVGYRTT- ------HHFL YPESAIHLR- ----PGVHLV
Pmargal2   CRRCAVVGNS GNLLGAGGGK EIDQHDFVM- RINQAPTLGF ETDVGARTT- ------HHFM YPESAKNLP- ----ANVSFV
Olatigal2B CLRCAVVGNS GNLRGSGYGK IIDEHNFIM- RINLAPTVGY EEDVGSRTT- ------HQFM YPESAKNLA- ----PNISFV
Drerigal2B CLRCAVVGNS GNLRGAGYGP VIDGHDFIM- RMNLAPTVGY EEDAGSRTT- ------HHFM YPESAKNLA- ----ANVSFV
Acarogal2B CLRCAVVGNS GNLRGSGYGR EINGHDFVM- RMNQAPTVGF EADVGSRTT- ------HHFM YPESAKNLP- ----ANVSFV
Stropgal2B CRRCAVVGNS GNLRGSSYGK DIDGHEFIM- RMNQAPTVGF EMDVGSRTT- ------HHFM YPESAKNLP- ----ANVSFV
Pythongal2 CRRCAVVGNS GNLRGSSYGQ EINGHDFIM- RMNQAPTVGF EADVGSRTT- ------HHFM YPESAKNLP- ----ANVSFV
Oanatgal2 CQRCAVVGNS GNLRGSGYGR DIDGHNFIM- RMNQAPTLGF EQDVGSRTT- ------HHFM YPESAKNLP- ----ANVSFV
Chrysegal2 CRRCAVVGNS GNLRGSGYGR EIDGHNFIM- RMNQAPTVGF EADVGGRTT- ------HHFM YPESAKNLP- ----ANVSFV
Ggallusga2 CRRCAVVGNS GNLRGSGYGH EIDGHDFIM- RMNQAPTVGF EGDVGSRTT- ------HHFM YPESAKNLP- ----ANVSFV
Hsapiegal2 CRRCAVVGNS GNLRGSGYGQ DVDGHNFIM- RMNQAPTVGF EQDVGSRTT- ------HHFM YPESAKNLP- ----ANVSFV
Tguttagal2 CRRCAVVGNS GNLRGSGYGP EIDGHDFVM- RMNQAPTVGF EGDVGGRTT- ------HHFM YPESAKNLP- ----ANVSFV
Strcamgal2 ---------- ---------- ---MCLTLP- RMNQAPTVGF EGDVGSRTT- ------HHFM YPESAKNLP- ----ANVSFV
Olatigal1A CRTCSVVGNS GNLKGSNYGA LIDTSDLVI- RMNKAPTKGF EKDVGAKTT- ------HHVM YPESAVDLS- ----NTTSLV
Tnigrgal1A CRTCAVVGNS GNLKGSQYGR LIDSSDFII- RMNQAPTLGF EEDVGTKTT- ------HHVM YPESAKDLD- ----NGTSLV
Trubrgal1A CRTCAVVGNS GNLKGSQYGR LIDSSDFII- RMNQAPTSGF EDDVGTRTT- ------HHIM YPESAKDLD- ----NGTSLV
Drerigal1C CRTCAIIGNS GNLKGSRYGR LIDAHNFVI- RINMGPTKGY EDDVGSKTT- ------HRFI YPESAVDFD- ----NSTYLV
```

```
Drerigal1D CRTCAVVGNS GNLNGSHYGV LIDAHDLVI- RINKGPTEGF ERDVGLKTT- ------HRMI YPESAVDMD- ----NSTYLV
Drerigal1A CRICAVVGNS GNILGSHYGQ LIDSHDFVI- RINKGPTKGY ETDVGSKTT- ------HRIM YPESAMDLD- ----NSTHLV
Drerigal1B CRTCAVVGNS GNLLRSNYGR LIDLHDFVL- RINKGPTKGF EKDVGSKTT- ------HRIM YPESAVDLD- ----NSTHLV
Sarcopgal3 CRRCIIIGNG GVLANKSLGA RIDDYDIVV- RLNSAPVKGF EKDVGGKTT- ------LRIT YPEGAMQR-P EEYEKDSLFV
Hsapiegal3 CRRCIIVGNG GVLANKSLGS RIDDYDIVV- RLNSAPVKGF EKDVGSKTT- ------LRIT YPEGAMQR-P EQYERDSLFV
Oanatgal3 CRRCIIVGNG GVLANKSLGA KIDDYDVVV- RLNSAPVKGF EKDVGSKTT- ------LRIT YPEGAIQK-P EQYERDSLFV
Btaurugal3 CRRCIIVGNG GVLANKSLGS RIDDYDIVV- RLNSAPVKGF EKDVGSKTT- ------LRIT YPEGAMQR-P EQYERDSLFV
Xlaevigal3 CRRCIIVGNG GVLANKSLGS KIDEYEVVV- RLNSAPVKGF EKDVGSKTT- ------LRIT YPEGAIQK-L EQYEKDSIFV
Stropigal3 CRRCIIVGNG GVLANKSLGS KIDEYEVVV- RLNSAPVKGF EKDVGSKTT- ------LRIT YPEGAIQK-L EQYEKDSIFV
Pythongal3 CRRCVIVGNG GILANKSLGL KIDDYDIVI- RLNSAPVKGF EKDVGGKTT- ------MRIT YPEGAIQK-P EQYEKDSLFV
Acarolgal3 CRRCIIVGNG GILANKSLGV KIDDYDIVI- RLNSAPVKGF EKDVGGKTT- ------MRIT YPEGAIQK-P EQYEKDSLFV
Chrysegal3 CRRCIIVGNG GVLANKSLGL KIDDYDVVV- RLNSAPVKGF EKDVGGKTT- ------LRIT YPEGAIQK-L EQYEKDSFFV
Ggallugal3 CRRCIIVGNG GVLANKSLGL KIDDYDVVV- RLNSAPVKGF EKDVGGKTT- ------LRIT YPEGAIQK-M EQYEKDSLFV
Strcamgal3 CRRCIIVGNG GVLANKSLGL KIDDYDVVV- RLNSAPVKGF EKDVGGKTT- ------LRIT YPEGAIQK-M EQYEKDSLFV
Tguttagal3 CRRCIIVGNG GVLANKSLGL KIDDYDVVV- RLNSAPVKGF EKDVGGKTT- ------LRIT YPEGAIQK-M EQYEKDSLFV
Drerigal3a CKKCIIIGNG GILFNKSLGT KIDQYDVVV- RLNEAPVAGF EKDVGSKTT- ------MRIT YPEGAIQR-A ERYEKSSLFV
Oryzlagal3 CKRCIIVGNG GILSNKSLGS RIDQYDVVI- RLNEAPVKGY SKDVGSKTT- ------MRIT YPEGAIQK-P ENYEDNSLFV
Tnigrogal3 CKRCIIVGNG GILSNKSLGS RIDEYDVVV- RLNEAPVFGY DKDVGSKTT- ------LRIT YPEGAIQK-P DVYERDSLFV
Dreriogal3 CKRCIIVGNG GILSNKSLGS RIDEYDVVV- RLNEAPVSGY TRDVGSKTT- ------MRIT YPEGAIQK-P ERYEKDSLFV
Dreriogal7 CRRCVVVGSR GILHSKNLGA HIDHANIII- RVNNAPVFGF ESDAGSRTT- ------IRLI YPEGAPSH-I QEYERTEVVA
Tnigrogal5 CRRCVVVGSG GVLHGSQLGT HIDQYDVII- RMNNAPVAGF ERDAGSHTT- ------VRLM YPEGAPRS-T NEYQDTTMVA
Oryzlagal5 CRRCVVVGNG GVLQGSHLGP HIDQYDVII- RMNNAPVVGF ERDVGSRTT- ------IRLM YPEGAPHS-A NEYRKTSMIA
Trubrigal5 CRRCVVVGNG GILRGLELGS LINRFDVVI- RLNSGPLGEY TADVGNRTT- ------IRMS YPEGTPLH-W VDSDPDITFV
Dreriogal5 CRRCVVVGNG GILKGLGLGH LLNRFDIII- RLNSGPLQDF SADVGNRTT- ------IRMS YPESCPKV-W EDTDPDLKYV
Pmaringal5 CRRCVVVGSG GNLRGTGLGP LLDNFDVVIR RLNNAPIIKF ENDVGSKTT- ------VRIT YPEGAPKS-M EEYKPDSIFA
Stropigal5 CKRCIVIGSG GILHGLELGQ MVDQFDIVI- RLNNAPVHGY AQDVGNKTT- ------IRMT YPEGAPVS-E QEYQHSSLFV
Hsapiegal5 CRRCVVIGSG GILHGLELGH TLNQFDVVI- RLNSAPVEGY SEHVGNKTT- ------IRMT YPEGAPLS-D LEYYSNDLFV
Oanatigal5 CKRCIVIGSG GILHGLGLGP ALNQFDIVI- RLNNAPVQGY ADHVGNKTT- ------IRMT YPEGAPLS-E HEYYSNGLFV
Acarolgal5 CKHCVVVGNG GVLHGLELGY VLNQFDIVI- RLNSAPVQGY TDDVGNKTT- ------IRMT YPEGAPLS-Q HEYYPDSLFV
Chrysegal5 CKRCVVVGSG GILHGLELGH VLNEFDIVI- RLNDAPVQGY TDHVGNKTT- ------IRMT YPEGAPLS-E NEYNPQSLFV
Ggallugal5 CKRCVVIGSG GILYGSELGH LLNQYDIVI- RLNDAPVQGY TDHVGNKTT- ------IRMT YPEGAPLS-E HEYPPASLFV
Strcamgal5 CKRCVVIGSG GILHGSELGH LLNQFDIVI- RLNDAPVQGY TDHVGNKTT- ------IRMT YPEGAPLS-E REYHPASLFV
Tguttagal5 CKRCVVVGSG GILHGSELGH LLNQFDIVI- RLNDAPVQGY TDHVGNKTT- ------IRMT YPEGAPLS-E HEYPPASLFV
Stropigal6 CKKCVVVGNG GVLRNSTLGK KIDSYDIII- RMNDGPVLGY EDDVGRKTT- ------FRLC YPESIFSN-S LHYDPNSTVV
Sarcopgal6 ---------- ---------- --------F- RINDGPVRGH EEDVGTKTT- ------FRIF YPESVFTN-P EDSDPESVAI
Hsapiegal6 CKKCVVVGNG GVLKNKTLGE KIDSYDVII- RMNNGPVLGH EEEVGRRTT- ------FRLF YPESVFSD-P IHNDPNTTVI
Acarolgal6 CKKCVVVGNG GVLRNKSLGE TIDSFDVVI- RMNNGPVVGY EEDVGRRTT- ------YRLF YPESVFSD-P IHYDPNTTAV
Chrysegal6 CRRCVVVGNG GVLRNKTLGE KIDSYDVII- RMNNGPVRGY EEDVGKRTT- ------FRLF YPESVFSD-P IHYDPDTTVV
Ggallugal6 CRRCVVVGNG GVLRNKTLGG KIDSYDVII- RMNNGPVIGY EEDVGRRTT- ------FRLS YPESIFSD-P IHYDPNTTVV
Strcamgal6 CRRCVVVGNG GVLRNKTLGE KIDSYDVII- RMNNGPVIGY EEDVGRRTT- ------FRLS YPESIFSD-P VHYDPNTTVV
Tguttagal6 CRRCVVVGNG GVLRNKTLGE KIDSYDVII- RMNNGPVIGY EEDVGRRTT- ------FRLS YPESIFSD-P IHYDPNTTVV
Pmaringal6 CKRCVVVGNG RVLKGSGLGK RIDSYDVVL- -MNDAPLRGH VGDVGSRTT- ------MRLC YPESAHVR-R EDFDPSALLA
Dreriogal4 CRTCAVIGNG FALKNSSLGE IINKYDVVI- RLNDAPVRGF EEDVGNKTT- ------LRLF YPESASYNPG IHNDPDTLLV
Oryzlagal4 CRTCVVVGNG FAIKNTSLGR IINNYDVVI- RLNDAPVRGY EEDVGNKTT- ------LRIF YPESASSNPR LHNEEDTLMV
Oranatgal4 CRRCVVVGNG YRLRNSSLGD VIDMYDVII- RYPLAELAEA WALPGCGSWV VPHATDDWQY WANGGGL--- ----------
Stropigal4 CRRCVVVGNG HQLKNSSLGE TINKYDVVI- RINNAPVHKY EKDVGSKTT- ------MRLF YPESADFDPQ LDNNPDTLLV
Xlaevigal4 CRRCVVVGNG HHLKNSSLGE TINKYDVVI- RLNNAPVHKY EKDVGSKTT- ------MRLF YPESADFDPQ LDNNPDTLLV
Pythongal4 CRRCAVVGNG HRLRNSSMGE VINKYDVVI- RLNNAPVHGY ERDVGSKTT- ------MRLF YPESAHFNPQ TENNPNTLLV
Mondomgal4 CLRCVVVGNG HRLKNSSLGN TIDKYDVVI- RLNNAPVVGY EGDVGSKTT- ------MRLF YPESAHFSPK VQNNPDTLLV
Hsapiegal4 CRRCVVVGNG HRLRNSSLGD AINKYDVVI- RLNNAPVAGY EGDVGSKTT- ------MRLF YPESAHFDPK VENNPDTLLV
Btaurugal4 CRRCVVVGNG HRLRNSSLGE AINKYDVVI- RLNSAPVAGY EQDVGSKTT- ------MRLF YPESAHFNPK VEDNPDTLLV
Strcamgal4 CRRCAVVGNG HRLRNSSMGE TINTYDVVI- RLNNAPVHGY EQDVGSKTT- ------MRLF YPESAHFNPR RENNPDTLLV
Chrysegal4 CRRCAVVGNG HQLRNSSMGE AINKYDVVI- RLNNAPVHGY EADVGSKTT- ------MRLF YPESAHFDPR RENNPDTLLV
Ggallugal4 CRRCVVVGNG HRLRNSSMGD TINTYDVVI- RLNNAPVHGY EQDVGSKTT- ------MRLF YPESAHFDPQ AENNPNTLLV
           LFPFKVLDIE WLKSIFTTHP ISK-----GW THLPTNLG-- LKPTDAMVIH PEFIYYVAKT WLEGK----- ----------
           LVPFQPLDIK WLLSALTTGE ITR-----TY QPLVRRVT-- CDKSKITIIS PTFIRYVHDR WTQHH----- ----------
           LLNFKPLDLK WMKTSLTDGS ITR-----TW TRVKGRIK-- ANKTKILVYN PAFFKYVNDK WTEHH----- ----------
           LLNFKPLDLK WMKTSLTDGS ITR-----TW TRVKGRIK-- ANKTKILVYN PAFFKYVNDK WTEHH----- ----------
           LIPFKPLDVS WLISALTTGT IVR-----TY MPVRKQIN-- VSKXMIEVYN PAFMYYVHKT WNELH----- ----------
           LIPFKTLDLR WVVTALTTGT INF-----TY VPVPRKIK-- VKKEKILVYN PTFMKYIYEN WLEHH----- ----------
           VIPFKTLDLR WIVTALTTGT INF-----TY VPVPRKIK-- VRKEKVLIYN PSFIKYVYEN WLQNH----- ----------
           MIPFKTLDLR WIVTALTTGT INF-----TY VPVPRKIK-- VKKEKILIYN PAFIKYVYEN WLQNH----- ----------
           VIPFKTLDLR WIVSALTTGT INS-----TY VPVPRKIKIK VKRDQILVYH PVFIKYVYDH WLQNH----- ----------
           LIPFKTLDLR WIISALTTGT INS-----TY IPVPRKIK-- ANKNKILVYN PVFIKYVYDN WLQHH----- ----------
           LIPFKILDLR WVISALTNGT INQ-----TY IPVPRKIK-- ANKDKIMVYH PTFMKYVYDN WLQRH----- ----------
           LVPFKTIDLE WVVSAITTGT ISH-----TY IPVPAKIR-- VKQDKILIYH PAFIKYVFDN WLQGH----- ----------
           LVPFKTTDLQ WVVSAITKGT IYF-----TY VPVPRKIK-- VSKEKILIYH PVFIKYVFDN WLQGH----- ----------
           LLPFKLRDIE WLTSALSTGK VKM-----TY MRVKDRVA-- ADKNKILVVN PVFFKYVNDH WTERH----- ----------
           LLPFKLRDLE WLTSALSTGH IKT-----TY MRVKERIQ-- ADKDKVLVVN PAFFKYVHDR WTEHH----- ----------
           LIPFKTLDLQ WLASALTHGT INR-----TY VQVPRKIR-- VSKDKVLVYS PELMKYVYDK WLLNH----- ----------
           LVPFKPLDLK WLASAFSSGE IPS-----TY RRVKQFVE-- ADKSQVRIIN PAFLKYIQDK WTKHH----- ----------
           LVPFKPLDLK WLASALSTGD IQR-----TY KRVKQFIK-- ADKSKVRILY PGFLKYIQDK WTKYH----- ----------
           LIPFKLQDLK WITSALTTGE VTY-----TY TRMKKFIK-- ADKDKVLIFN PSFFKYVNDN WTNHH----- ----------
           LVPFKPLDLK WVTSAFSTGE LRS-----TY TRVKQFIE-- ADRSQVLILN PAFLKYIHDK WTQHH----- ----------
           LLPFKLKDMQ WLSSALSTGE IKM-----TY MRVKNRID-- ADKDKVMVVN PAFFKYTHDR WTERH----- ----------
           LVPFKVLDLM WIASALSTGE IRF-----TY APVKRFLS-- VDKDKVLIYN PAFFKYIHDS WTEHH----- ----------
           LVPFKTLDLV WITSALSTGQ IRF-----TY APVKQFLR-- VDKDKVQIFN PAFFMYIHDH WTKHH----- ----------
           LVPFKTLDLL WITSALSTGQ IRF-----TY APVKQFLR-- VDKDKVQIFN PAFFKYIHDR WTRHH----- ----------
           LVPFKALDLL WIASALSTGQ IVF-----TY APVKPFLR-- VDKEKVQIYN PAFFKYIHDR WTEHH----- ----------
           LVPFKALDLL WITSALSTGQ IRF-----TY APVKAFLR-- VDKEKVQIYN PAFFKYIHDR WTEHH----- ----------
           LVPFKALDLL WIASALSTGQ IRF-----TY APVKPFLR-- VDKERVQIYN PAFFKYIHDR WTEHH----- ----------
           LVPFKALDLL WIASALSTGQ IRF-----TY APVKSFLR-- VDKEKVQIYN PAFFKYIHDK WTEHH----- ----------
           LVPFKALDLL WITSALSTGQ IRF-----TY APVKPFLR-- VDKEKVQIYN PAFFKYIHDR WTEHH----- ----------
           LVPFKTLDLL WIASALSTGQ IRF-----TY APVKPFLR-- VDKEKVQIYN PAFFKYIHDR WTEHH----- ----------
           LVPFKVLDLL WIASALSTGQ IRF-----TY APVKSFLR-- VDKEKVQIYN PAFFKYIHDR WTEHH----- ----------
           LVPFKTLDLL WIASALSTGQ IRF-----TY APVKPFLR-- VDKEKVQIYN PAFFKYIHDR WTEHH----- ----------
           LVPFKTLDLL WIASALSTGQ IRF-----TY APVKPFLR-- VDKEKVQIYN PAFFKYIHDR WTEHH----- ----------
           LVPFKTLDLQ WIISALTTGT IRS----RTY MRVKERIQ-- ADKDKVVVIN PRFIKYVYES WLEGR----- ----------
           LIPFKTLDLQ WIISALTTGT IKH-----KY VPVMSRIK-- ANKDKVVIYS PTFFKYVYES WLEGH----- ----------
           LIPFKTLDLQ WIISALTTGA IKH-----TY VPVMSRIK-- ANKDKVVIYS PTFFKYVYES WLDGH----- ----------
```

```
LSPFKVLDIE WLISSFTTKN ITR-----TY KKVRASIN-- ANRHKVMILH PAFIKYVHEI WLVKH----- ----------
LIPFKTLDLQ WLISVFTTKH IDR-----TY IPVKPTIK-- ANRNKVMVLH PSFLKYVQEH WLQKH----- ----------
LLPFKVKDMQ WLISVFTTKH ITS-----TY LKVRPTIN-- ADREKVMIIH PAFIKYVYES WLRKH----- ----------
LFPYKILDMQ WLISIFTTKH ITR-----TY MSVPSTIN-- ADRDKVMILH PEFIKYVNDK WAEGH----- ----------
LAGFKWQDFK WLKYIVYKEK VSA--SDGFW KSVATLVP-- KEPPEIRILN PYFIQEAAFS FIGLPFNHGL MGRGPLQAGM
LAGFKWQDFK WLKYIVYKER VSA--SDGFW KSVATRVP-- KEPPEIRILN PYFIQEAAFT LIGLPFNNGL MGRG------
LAGFKWQDFK WLKYIVYKEK VSA--TDGFW KSVATHVP-- KEPHEIRILN PYFIQEAAFS FIGLPFNNGL MGRG------
LAGFKWQDFK WLKYIVYKER VSA--SDGFW KSVATRVP-- KEPPEIRILN PYFIQEAAFT LIGLPFNNGL MGRG------
LAGFKWQDFK WLKYIVYKEK VSA--SDGFW KSVATRVP-- REPHEIRILN PYFIQEAAFS FIGLPFNNGL MGRG------
LAGFKWQDFK WLKYIVYKEK VSA--ADGFW KSVATRVP-- REPHEIRILN PYFIQEAAFS FIGLPFNNGL MGRG------
LAGFKWQDFK WLKYIVYKEK VSA--SDGFW KSVATHVP-- REPHEIRILN PYFIQEAAFS FIGLPFNNGL MGRG------
LAGFKWQDFK WLKYIVYKEK VSA--SDGFW KSVATHVP-- REPHEIRILN PYFIQEAAFS FIGLPFNNGL MGRG------
LAGFKWQDFK WLKYIVYKEK VSA--SDGFW KSVATRVP-- REPHEIRILN PYFIQEAAFS FIGLPFNNGL MGRG------
LAGFKWQDFK WLKYIVYKEK VSA--SDGFW KSVATRVP-- REPHEIRILN PYFIQEAAFS FIGLPFNNGL MGRG------
LAGFKWQDFK WLKYIVYKEK VSA--SDGFW KSVATRVP-- REPHEIRILN PYFIQEAAFS FIGLPFNNGL MGRG------
LAGFKWQDFK WLKYIVYKEK VSA--SDGFW KSVATRVP-- REPHEIRILN PYFIQEAAFS FIGLPFNNGL MGRG------
LSAFKSNDFK WLRHMVYKDR LWR--MDGFW KSVARVVP-- RAPQDMRILN PYFIQEASFR LIGLPHNNGL MGRG------
FSAFKPLDFK WLKQMVYKEK VRG--MEGFW KSVAQYVP-- RAPADMRILN PYFIQEAAFQ FIGLPFNNGL MGKG------
FSAFKPLDFK WLKQMVFKEK LKG--TEGFW KSVAHHVP-- REATEIRILN PFFIQEAAFQ FIGLPFNNGL MGKG------
FSAFKPLDFK WLRQMVYKEK LVR--LEGFW KSVARYVP-- REPSEIRILN PYFIQEAAFQ FIGLPQNNGL MGKG------
LVVFKSLDLA WMTSVVTKEP LVWWSKLWFW KDVIDSIP-- LQPENVRILN PEIMYRTGQV LQTYAEQQRK ----------
LVVFKSLDLD WLTSVVTKQP LGFWSKLWFW REVVDDIP-- LKPESFRILH PEIIHRTGQV LKRYTMTQG- ----------
LVVFKSLDLD WLTSVITKKP LSFWSKMWFW REVVDDIP-- LSPESFKILH PEIIHKTGQV LQKYTLKQG- ----------
AVVYKGVDVS WTTAMINRIS VPLWDWLFFW QKVPDHIP-- LDSHRFRLLN PGVIRETALD LLQFPPPRSQ LWGN------
AVIFKSVDFH WLRAMISRTP VSLWDRLFFW QNVPMSVP-- VKTSQFHLLN PQIIREMALD LLNYPEPKKR LWSW------
AVMYKGVDFQ WLESIIKKEH VVG--SVSFG KKVADGVP-- LGPSQFRILN LEIIGETSMD LLGYPYLLLI VFFW------
TVLFKHADFR WLEAVLKNQK LSAWNRFFFW KRVTDKIP-- LNSSQFRVLN PLIIKETAID ILGFPKPQKR WLGW------
AVLFKSVDFN WLQAMVKKET LPFWVRLFFW KQVAEKIP-- LQPKHFRILN PVIIKETAFD ILQYSEPQSR FWGR------
AVLFKSVDFN WLQAMVKNES VPLWVRLFFW KQVAEKIP-- LQSKQFRILN PVIIKETALD ILQYPEPRSK IWGW------
AVLFKSVDFG WIQAVVKNET LPLWMRLFFW KQVAEKIP-- VMPKQFRILN PAIIKETALD ILEYPEPRPK FWGW------
VVLFKSVDFN WLQAMVKNEA LPLWVRLFFW KQVAEKIP-- VTPKQFRILN PIIIKETALD ILQYPEPRSK FWGW------
AVLFKSVDFN WLQAMVKNET LSLWIRLFFW KEVAKKIP-- FTSKQFRILN PVIVKETALD ILEFPKPRSI FWGW------
AVLFKSVDFN WLQAMVKNET LPLWVRLFFW KEVAAKIP-- FTSKQFRILN PIIVKETALD ILQFPEPRSK FWGW------
AVLFKGVDFN WLQAMVKNET LHLWVRLFFW KQVAEKIP-- FTPKQFRILN PVIIKETALD ILHFPEPRSK FWGW------
LLMFKPHDVK WLSELLMHKR VS---TYGFW RKPAMNLIY- -KPHQLRVLN PFILRQASQN LLNFPFSFPK TEK-------
LAVFKPHDLK WLWELLSGK- -----VDHFW KKPALNLIY- -KPEKIRILD PIITRIAAFN MLHFPTTFPK KEK-------
LTAFKPHDLR WLLELLMGDK IN---TNGFW KKPALNLIY- -KPYQIRILD PFIIRTAAYE LLHFPKVFPK NQK-------
LVVFKPYDLK WLWDLLSGQR IK---TSAFW KKPALQLIY- -NAQQIRILD PAITRRTAHD MLHLPTKFSR HER-------
LLVFKPHDLK WLWNILNGHK IN---TNGFW KKPALKMIY- -KVNQIRILD PIIIRKTAHE WLQFPTKFPR KEK-------
IIVFKPRDLK WLWEILGGQK IS---AKGFW KKPALNMIY- -KSNQIRILD PSITRKTAYD WLHFPTRFPK KEK-------
LIVFKPRDLK WLWEILGGQK IS---AKGFW KKPALNMIY- -KSSQIRILD PSITRKTAYE WLHFPTRFPK KEK-------
LIVFKPRDLK WLWEILGGQK IS---AKGFW KKPALNMIY- -KSSQIRILD PSITRKTAYE WLHFPTRFPK KEK-------
FVPFKTRDLQ WLLGVLNHHR VVG--RPGFW KKVPTELQFS AHVRRIRILN PAFVREAATE FLINW----- --S-------
LVPFKQQDLR WLKEILYDEK RV---SKGFW KPPPQ--IWL GRASQIRVLD PYFLRITARK FLQIPV---- ----------
LVPFKPDDLR WLKEILYDEK RV---RKGFW KPPPL--IWL GQSSKVRVLD PYFMQQTANK LLQFPLAPKK GQN-------
--------GP WRKMTGSFSF WV---RKGFW RQPPL--IWE ADPTQVRVLN PFFMEVTAAK LLSLPMRQTR KV--------
LVPFKPLDIQ WMKIILNNEK RV---RKGFW KMPPI--IWE VEPENIRILN PYYMSVTATQ ILKSK-KMIP KM--------
LVPFKQLDIQ WMKIILNNEK RV---RKGFW KMPPI--IWE VEPENIRILN PYYMAVTATQ ILKSK-KM-- ----------
LVAFKPMDFM WMETILHDKK RI---RKGFW KQPPL--IWN ANPKQVRILN PYFMEVTAAK VLKLPVKQLW KL--------
LVPFKAIDFH WIETILRDKN RV---LKGFW KQPPL--IWD VNPKQVRILN PFFMEVTAAK LLSLPIRPSR KI--------
LVAFKAMDFH WIETILSDKK RV---RKGFW KQPPL--IWD VNPKQIRILN PFFMEIAADK LLSLPMQQPR KI--------
LVAFKAMDFH WIESILSDKK RV---RKGFW KQPPL--IWD VNPKQIRILN PFYMEIAADK LLSLPIHQPH KI--------
LVPFKPMDFQ WMEAILNDKK RV---RKGFW KQPPL--IWD ANPEQVRILN PYYMEVTAAK LLNLPMKQLR KV--------
LVPFKPMDFQ WLEMILNDKK RV---RKGFW KQPPL--IWD ANPEQVRILN PYFMEITAAK LLNLSMKQPR KI--------
LVPFKPVDFQ WMEAILSDRK RV---RKGFW KQPPL--IWD ANPEQVRILN PYYMEVTAAK LLSLPMKQPR KV--------
-------GRW ASAGALSVVW ALHIC---NE VDVFGFGANK YGN---WDHY YEKFS---SK EKDPFRRTGV HDANIEE---
-------GRY PSTGLLALIY ALHEC---DE VDVYGFGANR AGN---WHHY WEDLP---PH VAGAFRKTGV HDSAQENEII
-------GRY SSTGSLVILF AVHVC---DE VDVYGYGADK LGN---WNHY WTTHY----- SGAAHRSTGV HDSEFERVLL
-------GRY SSTGSLVILF AVHVC---DE VDVYGYGADK LGN---WNHY WTTHY----- SGAAHRSTGV HDSEFERVLL
-------GRY PSTGMLVLLF AMHIC---DQ VNVFGFGATS KGN---WDHY YQPPVEPSLE KDSAFKLTGV HNAEFETDIV
-------GRY PSTGLLSLMF ALHVC---DE VNVYGFGADS KGH---WHHY WENNT----- SGGAFRKTGV HDGDFEFNIT
-------GRY PSTGLLSVIF ALHVC---DE VNVYGFGADS KGH---WHHY WENNA----- SAGAFRQTGV HDGDFEFNVT
-------GRY PSTGLLSLIF ALHVC---DE VNVYGFGADS RGH---WHHY WENNL----- SAGAFRQTGV HDGDFEFNIT
-------GRY PSTGILSVIF ALHLC---DE VDVYGFGADS NGN---WHHY WENNA----- SGGAFRKTMV HDGDFESNIT
-------GRY PSTGFLSVIF ALHLC---DE VDLYGFGADS KGN---WHHY WENNP----- SAGAFRQTGV HDGDFESNIT
-------GRY PSTGFLSIIF ALHLC---DE VDLYGFGADS KGN---WHHY WENNP----- SAGAFRQTGV HDGDFESNVT
-------GRY PSTGILSVIF SMHVC---DE VDLYGFGADS KGN---WHHY WENNP----- SAGAFRKTGV HDADFESNVT
-------GRY PSTGILSVIF SLHVC---DE VDLYGFGADS KGN---WHHY WENNP----- SAGAFRKTGV HDGDFESNIT
-------GRY PSTGMLAIIF ALHIC---DQ VSVFGYGADE QGN---WHHY WEENR----- YAGAFRKTGV HSAEFETQII
-------GRY PSTGMLAIVF ALHVC---DQ VSVFGYGADR EGN---WHHY WEKNR----- HAGAFRKTGV HNADFETEII
-------GRY PSTGLLAVIF ALHVC---DK VDLYGFGADS KGH---WHHY WENNA----- SAGAFRLTGV HDGDFEASIL
-------GRY PSTGFTALIF AIHTC---QQ VSAFGFGADN NGN---WHHY WEENH----- YAGAFRKTGV HDANFELGLI
-------GRY PSTGLIALIF AIHTC---TQ VSAFGFGANS NGN---WHHY WEDNR----- YGGAFRKTGV HNATFELQLI
-------GKY PSTGMLALVF ALHIC---DE ISVFGFGADQ HGN---WHHY WERNR----- FAGAFRWTGV HNAEFESDLI
-------GRY PSTGLTALLF ALHIC---DQ VSVFGYGADN NGN---WHHY WEANR----- YPGAFRKTGV HNAGFELTLI
-------GRY PSTGIVAIIF ALHLC---DE VSVFGYGADA QGN---WHHY WEYNR----- YAGAFRKTGV HNADFETEII
-------GRY PSTGMVALFF ALHVC---DE VDVYGFGADS RGN---WHHY WENNR----- YSGEFRKTGV HDADHEEKII
-------GRY PSTGMLVLFF ALHVC---DE VNVFGFGADS RGN---WHHY WEQNR----- YSGEFRKTGV HDADYEAQII
-------GRY PSTGMLVLFF ALHVC---DE VNVFGFGADS RGN---WHHY WEQNR----- YSGEFRKTGV HDADYEAQII
-------GRY PSTGMLVLFF ALHVC---DE -NVFGFGADS RGN---WHHY WENNR----- YAGEFRKTGV HDADFEAHVV
-------GRY PSTGMLVLFF ALHVC---DE VNVFGFGADS RGN---WHHY WENNR----- YAGEFRKTGV HDADFEAQII
-------GRY PSTGMLVLFF ALHVC---DE VNVFGFGADS HGN---WHHY WENNR----- YAGEFRKTGV HDADFEAHIV
-------GRY PSTGMLVLFF ALHVC---DE VNVYGFGADS RGN---WHHY WENNR----- YAGEFRKTGV HDADFEAHII
-------GRY PSTGMLVLFF ALHVC---DE VNVFGFGADS RGN---WHHY WENNR----- YAGEFRKTGV HDADFEAHII
-------GRY PSTGMLVLFF ALHVC---DE VNVFGFGADS RGN---WHHY WENNR----- YAGEFRKTGV HDADFEAHII
-------GRY PSTGMLVLFF ALHVC---DE VNVYGFGADS RGN---WHHY WENNR----- YAGEFRKTGV HDADFEAHII
-------GRY PSTGMLVLFF ALHVC---DE VNVFGFGADS RGN---WHHY WENNR----- YAGEFRKTGV HDADFEAHII
-------GRY PSTGMLVLFF ALHVC---DE VNVFGFGADS RGN---WHHY WENNR----- YAGEFRKTGV HDADFEAHII
-------GHY PSTGFLTLMF ALHIC---DE VSVFGFGADQ HGN---WHHY WEHNQ----- LAGAFRHTGV HDGDYEYNLT
-------GRY PSTGFLSLLL AVHIC---DK VSVFGFGADQ YGN---WHHY WEENS----- LAGAFRHTGV HDGDYEYNVT
```

```
-------GRY PSTGFLSLLL AIHIC---DQ VSVFGFGADH YGN---WHHY WEENS----- LAGAFRHTGV HDGDYEYNVT
-------GKY PSTGFLAIIF ALHIC---DQ VSTFGFGADQ YGN---WYHY FEKTS----- S---RVRTGA HSGSFEFDTM
-------GRY PSTGFITLIF ALHIC---DQ VSVFGFGADK DGN---WHHY FEHKK----- H---PRNAGN HGGSYEYLIA
-------GSY PSTGFITLIF ALHIC---DQ VSVFGFGAKL DGN---WHHY FDESL----- A---HFNRGR HGGDYENRTI
-------GGY PSTGFLTLIF ALHIC---DE VNVFGFGATS EGQ---WHHY FDDSM----- T---SFS-NL HGGDFENQTI
LAGPLPLQNI PTLGTVAVTM ALHSC---DQ VAVAGFGYDM SSP-NAPLHY YESVR----- MAAI-KE--- ----------
--------NI PTLGSVAVTM ALHGC---DE VAVAGFGYDM STP-NAPLHY YETVR----- MAAI-KESWT HNIQREKEFL
--------NI PTLGSVAITM ALHNC---DE VAVAGFGYDM NSP-NTPLHY YEAIK----- MSAI-KET-- ----------
--------NI PTLGSVAVTM ALHGC---DE VAVAGFGYDM STP-NAPLHY YEAVR----- MAAI-RESWT HNIQREKEFL
--------NI PTLGSVAITM ALHNC---DE VAVAGFGYDM NLP-NAPLHY YETIK----- MAAI-KESWT HNIQKEKEFL
--------NI PTLGSVAITM ALHNC---DE VAVAGFGYDM NLP-NAPLHY YETIK----- MAAI-KESWT HNIQKEKEFL
--------NI PTLGSVAITM VLHNC---DE VAVAGFGYDM NSP-NAPLHY YENIK----- MSAI-KESWT HNIQREKEFL
--------NI PTLGSVAITM VLHNC---DE VAVAGFGYDM NSP-NAPLHY YETIK----- MSAI-KESWT HNIQREKEFL
--------NI PTLGSVAITM ALHNC---DE VAVAGFGYDM NSP-NAPLHY YESIK----- MSAI-KESWT HNIQREKEFL
--------NI PTLGSVAITM ALHNC---DE VAVAGLGYDM SSP-NAPLHY YENIK----- MSAI-KESWT HNIQREKEFL
--------NI PTLGSVAITM ALHNC---DE VAVAGFGYDM SSP-NAPLHY YENIK----- MSAI-KESWT HNIQREKEFL
--------NI PTLGSVAITM ALHNC---DE VAVAGFGYDM SSP-NAPLHY YENIK----- MSAI-KESWT HNIQREKEFL
--------NI PTLGTVAITM ALHNC---DE VAVAGFGYDM NTP-HAPLHY YEKLR----- MSAI-KESWT HNISREKEFL
--------NI PTLGTVAITM ALHNC---DE VAVAGFGYDM NMP-YAPLHY YETVR----- MSTI-KESWT HNIPKEKEFL
--------NI PTLGTVAITM ALHNC---DE VAVAGFGYDM NTP-HAPLHY YETVK----- MSAI-KEQWT HNIAREKEFL
--------NI PTLGTVAITM ALHNC---DE VAVAGFGYDM NTP-HAPLHY YESVK----- MSAI-KESWT HNISKEKEFL
-------VMV PTLGITAVVV ALQVC---DE VSIAGFGYDL QHP-GAPLHY YGSLR----- MDAM-KTQVV HDVSAETVFL
-------NMV PTLGASAVVM ALQVC---DQ VSLAGFGYDM RHP-ESRLHY YETIP----- MGAM-KAQVV HDISAEKLFL
-------NMN PTLGASALVM AMQLC---DQ VSLAGFGYNM QHP-EARLHY YEAIR----- MDAM-KAQVV HDVSAEKLFL
------QQNV PTIGMSALNL ASLLC---DE VSLAGFGYNF SVN-SARLHY YDKLP----- MRVM-QQQTS HNVKEETAFL
------DQNI PTLGLTALNL ATYIC---DE VSLAGFGYNL SQK-EAPLHY YDSVP----- MTTI-LKEAM HNVQKETVFL
------WQNI PTLGVSAVVF ATHLC---DE VSLAGFGYDL SRP-DAPLHY YENVR----- MDAM-KAQPM HNVDGEKLFL
------DKNV PTIGMTAVIL ATHLC---DE VNLAGFGYDL SQP-DASLHY YDNRC----- MNSM-NDQPM HDVTKEKKML
------DKNV PTIGVIAVVL ATHLC---DE VSLAGFGYDL NQP-RTPLHY FDSQC----- MAAM-NFQTM HNVTTETKFL
------DKNV PTIGVTAVVL ATHLC---DE VSLVGFGYDL SQP-NTPLHY FDTLC----- MAAM-NGQTM HNVTRETLFL
------DKNI PTIGVTAIVL ATHLC---DE VSLAGFGYDL SQP-NTPLHY YDNLC----- MAAM-NEQTM HNVTTETKFL
------DKNV PTIGVTAVVL ATHLC---DE VSLAGFGYDL RQP-NTPLHY YDNLC----- MAAM-EGQTM HNVTTETKFL
------DKNV PTIGVMAVVL ATHLC---DE VSIAGFGYDL NQP-STPLHY YNNLC----- MAAM-NRQTM HNVTGETKLL
------DKNV PTIGVTAVVL ATHLC---DE VSLAGFGYDL DQP-STPLHY YNNLC----- MAAM-NGQTM HNVTSETKFL
------DKNV PTIGVTAVVL ATHLC---DE VSLAGFGYDL DQP-STPLHY YNNLC----- MAAM-KEQTM HNVTSETKFL
-------PKH PTTGIIAITL AFHIC---NE VHIAGFKYNL TS-LNSSLHY YGNET----- MSVMAQN-EY HNISAEQMFI
-------PKH PTTGIIAITL AFYIC---HE VHLAGFKYNF TD-KDGPLHY YGNTT----- MSFMS---KY HNITAEQIFL
-------PKH PTTGIIAITL AFYIC---HE VHLAGFKYNF SD-LKSPLHY YGNAT----- MSLMNKN-AY HNVTAEQLFL
-------PRH PTTGLIAITF ALQIC---SE VHLAGFKYNL ND-RNSSLHY YGNDT----- MSQMMKN-AY HDIAVEQRFL
-------PKH PTTGLIAITL AFHIC---NE VHVAGFKYNF AD-RNSSLHY YGNET----- MSEMIEN-GY HNIIAEQKFL
-------PKH PTTGLIAITL AFHIC---HE VHLAGFKYDF TD-RNSSLHY YGNET----- MSQMMQN-EY HDISAEQKFL
-------PKH PTTGLIAITL AFHIC---HE VHLAGFKYDF TD-RNGSLHY YGNDT----- MSQMMQN-EY HNINAEQKFL
-------PKH PTTGLIAITL AFHIC---HE VHLAGFKYDF TD-RNSSLHY YGNET----- MSQMMQN-EY HNITAEQKFL
-------QKN PTTGVIAITL ALHLC---DE VHLAGFGYEV NSP-GSPVHY YGQDT----- MSSIIKKSTY HNITIERTFL
-------QVH PTTGLLAVFV ALNYC---DV VHVAGFGYPA SRNQNQPIHY YGQQT----- MKSMFQN-SY HDLNQEAQIL
-------HFH PTTGLLAVFV ALNYC---DM VHIAGFGYPP KSDKRQPIHY YGYHT----- MKSM-KN-SP HDVSREGRAL
-------KQK PTTGLLAITL ALHLC---DL VHIAGFGYPS SSNKKQTIHY YEQVT----- LKSM-AA-SE HNVSHEALAI
-------IPK PTTGLLAITF ALHFC---DM VHIAGFGYPA LTNKKQPIHY YEKVT----- LKSM-FA-SE HNITVEAQAI
-------TPK PTTGMLAITF ALHFC---DT VHIAGFGYPA LTNKKQPIHY YEKVT----- LKSM-AV-SG HNITLEAQAI
-------KQK PTTGLVAITL ALHFC---DV VHIAGFGYPS SDNKKQSIHY YEQIT----- VKSM-AV-SG HNVSHEAFAI
-------KQK PTTGLLAITL ALHLC---DL VHIAGFGYPD SSNKKQTIHY YEQIT----- LKSM-SV-SE HNVSHEALAI
-------KQK PTTGLLAITL ALHLC---DL VHIAGFGYPD AYNKKQTIHY YEQIT----- LKSM-AG-SG HNVSQEALAI
-------KQK PTTGLLAITL ALHLC---DL VHIAGFGYPD AHQKKQSIHY YEYIT----- LKSM-MW-SG HNVSQEALAI
-------KQV RVKDPAPPPR GRRWVHRPRS LRLGPVGAPV SPPVPFQ--F ---------- ---------- ----------
-------KQK PTTGLLAITL ALHFC---DL VHIAGFGYPD SANKKQTIHY YEQIT----- LKSM-AA-SE HNISHEALAI
-------KQK PTTGLLAITL ALHFC---DL VHIAGFGYPD SANKKQTIHY YEQIT----- LKSM-AA-SE HNISHEAVAI
---------- ---------- ---------- ------
DQLHIHGLLR VHRSEQSS-- ---------- ------
EKLQSEGIIK IHRGNAANK- ---------- ------
EKLQSEGIIK IHRGNAANK- ---------- ------
KQLEEIGKIT VYRGSRV--- ---------- ------
LTLASIEKIK FFKGR----- ---------- ------
LTLASIEKIK FFKGR----- ---------- ------
LTLASIEKIN FFKGR----- ---------- ------
LTLASIDKIR FFKGR----- ---------- ------
LTLASIDKIR FFKGR----- ---------- ------
HTLASVAKVR LFKGR----- ---------- ------
ATLASINKIR IFKGR----- ---------- ------
ASLASEDKIR IFKGR----- ---------- ------
HNLAKEGKIR LYL------- ---------- ------
QQLAIEGKIT LHR------- ---------- ------
ANLTSINKVL MFRGR----- ---------- ------
ERLADEGKVL FYK------- ---------- ------
ERLAIEGKLS FYK------- ---------- ------
AKLAEEGRLK LYK------- ---------- ------
KKLAAEGKIS FYN------- ---------- ------
QRLSAEGKIK LYR------- ---------- ------
KQLTEAGKIS VHRGR----- ---------- ------
QDLAKAGKIT VFPGK----- ---------- ------
DKLAKAGKIS VFPGK----- ---------- ------
GVLEKTGKID VYRGN----- ---------- ------
DNLAKEGKIK VYRST----- ---------- ------
DILEKTNKIS VYRGN----- ---------- ------
DMLAKASKIE VYRGN----- ---------- ------
DMLAKTSKIQ VYRGN----- ---------- ------
DMLAKTSRIE GVPGQ----- ---------- ------
DMLAKASKIE VYRGN----- ---------- ------
DMLAKTSKIE VYRGN----- ---------- ------
DMLAKTSKIE VYRGN----- ---------- ------
LTLADKHKIQ MFRGR----- ---------- ------
```

```
LLLADKHKIQ MFKGR----- ---------- ------
LLLADKHKIQ MFKGR----- ---------- ------
MQLYLESKIR VFRGR----- ---------- ------
LKLHEKQGIR LYKGW----- ---------- ------
HKLLLMNKIA LHKGL----- ---------- ------
HFLHQQNKIS LHKGWT---- ---------- ------
---------- ---------- ---------- ------
RKLVKARVIT DLSSGI---- ---------- ------
---------- ---------- ---------- ------
rklvkarvit dltsgi---- ---------- ------
KKLVKARVIN DLTNGI---- ---------- ------
KKLVKARVIN DLTNGI---- ---------- ------
RKLVKARVIT DLTSGI---- ---------- ------
RKLVKARVIT DLTSGI---- ---------- ------
RKLVKARVIT DLTSGI---- ---------- ------
RKLVKARVIT DLTSGI---- ---------- ------
RKLVKARVIT DLTSGI---- ---------- ------
RKLVKARVIT DLTSGI---- ---------- ------
RKLVKAGVIE DLTHGI---- ---------- ------
RKLVKANVIT DLTNGI---- ---------- ------
RKLVRANVIA DLTNGI---- ---------- ------
RKLVKANIIT DLTNGI---- ---------- ------
KELVKAGAVR DLTGAL---- ---------- ------
RDLVSAGAVT DLTGAL---- ---------- ------
RDLVAAGAVT DLTGAL---- ---------- ------
QKLIQDGAVT DLTGGIHCSF CPR------- ------
KRLVASGSIT DLTGGIHCSF C--------- ------
AGLINAGVIS DLTGGV---- ---------- ------
RTLVKEGVVR DLSGGIHCAF CDTQHYTE-- ------
LKLVKEGVVK DLSGGIDREF ---------- ------
QKLVQEEVVK DLSGGIHCDF CKKESEQSKR KMPLGI
QKLIKEGVVK DLSGGIFCTY CKKSS----- ------
QKLIKDGIVK DLSGGIQCEF CKKPS----- ------
QKLVKEKVVK DLTGGIHCEF ---------- ------
QKLIKEKVVK DLTGGIHCEF CIKDS----- ------
QKLIKEKIVK DLTGGIYCEF ---------- ------
RDLIEHKTIK NLT------- ---------- ------
KNIIEKNFVI NLTKD----- ---------- ------
KDIIEKNLVI NLTQD----- ---------- ------
KELIDLKILI NLT------- ---------- ------
KSLIEKQFVV SLT------- ---------- ------
KKLIDKNFVV NLT------- ---------- ------
KKLIDKNFVV NLT------- ---------- ------
KKLIDKNFVV NLT------- ---------- ------
KKLKEGEAIV DLTQLAGT-- ---------- ------
HRLEEQGVIL YLHPHS---- ---------- ------
KRLEDSGAVM YLHRHL---- ---------- ------
KRMLDIGAVK NLKDF----- ---------- ------
KNLFQQNIIH NLTYF----- ---------- ------
KSLLQQRIIS NLTYF----- ---------- ------
KKMLELGLVK NLTYF----- ---------- ------
KRMLEIGAIK NLTYF----- ---------- ------
KRMLEMGAIK NLTSF----- ---------- ------
KRMLEIGAVK NLTYF----- ---------- ------
---------- ---------- ---------- ------
KRMLELGVLK NLTHF----- ---------- ------
KRMLELGLVK NLTYF----- ---------- ------
```

Data S3. 101 ST6Gal sequences used in Figures 8 and 9: (48 ST6Gal I and 53 ST6Gal II. The alignment was build using profile-profile mode with clustalX, one profile comprises ST6Gal I and the other ST6Gal II sequences. Output format is FASTA.

```
>Hsap_ST6Gal1
--------------------MIHTNLKKK-----------FSCCVLVFLLFAVICVWKE-
----------------KKKGSYYDSFKLQ--------------------TKEFQV-----
-----LKSLGKLAMGSDSQSVSSSSTQD------------------PHRGRQTLGSLR--
------------------------------------------------------------
---------------------------GLAKAKPEASFQVWNKDSSSKNLIPRLQKIWKN
YLSMNKYKVSYKGPGPGIKFSA---EALRCHLRDHVNVSMVEVTDFPFNTSEWE-GYLPK
ESIR------TKAGPWGRCAVVSSAGSLKSSQLGREIDDHDAVLRFNGAPTANFQQDVGT
KTTIRLMNSQLVTTEKR-FLKDSLYNEGILIVWDPSVYHSDIPKWYQNPDYNFFNNYKTY
RKLHPNQPFYILKPQMPWELWDILQEISPEEIQPNPPSSGMLGIIIMMTLCDQVDIYEFL
PSKRKTDVCYYYQKFFDSACTMGAYHPLLYEKNLVKHLNQGTDEDIYLLGKATLPGFRTI
HC-------------
>Mdom_ST6Gal1
--------------------MVHINFFRR-----------IMYGLLAFLLFLTICLWNE-
----------------SKKG-YSVSFRLD--------------------SKGFQL-----
-----PRTLENLSRRLRTKPVNITAMVG------------------PQRESE---APR--
------------------------------------------------------------
---------------------------PRPKVDK-FSYKVWDKDSSSKNLPARLQKVRKN
YLHMNKYNVSFEGTRQVEKLST---SGLLCQLANRVKVTMIQGTDFPFNTSEWE-KSLPP
KNIR------ESFGRLGTCAVVSSAGSLKASYLGTEIDNHDAVLRFNGAPTKKYEEHVGK
KTTFRLVNSQLVTTDKN-FFDDSLYKNGILIMWDPSPYHSEIPEWYKKPDYNFFHNYKLY
RKLNPNQPFYILNPKMPWELWDILQENSPEDIQPNPPSSGMIGIVIMMNFCDTVDIYEFL
PSKRKTDICHYYQQFFDSACTMGAYHPLLFEKNMVKHLNQGTDEDIYLKGKVTVPGLQGV
RC-------------
>Cfam_ST6Gal1
```

```
--------------------MIHTNLKKK-----------FSCCVLAFLLFAVICVWKE-
----------------KKKGSYYDSLKLQ--------------------TKEFQM-----
-----VRG-------LEKQAATLSSTQN------------------PPRASQALGSPR--
------------------------------------------------------------
---------------------------GPVKAKSEASFQVWNKDSSSKNLIPRLQKIWRN
YLNMNKYKVSYKGPGPGVKFSA---EALHCHLRDHVNVSMVEATDFPFNTSEWE-GFLPK
ENIR------TKAGPWGRCAVVSSAGSLKSSQLGREIDDHDAVLRFNGAPTASFQQDVGT
KTTIRLMNSQLVTTEGR-FLKDSLYNEGILIVWDPSVYHSDIPKWYQSPDYSFFENYKSY
RKLHPDQPFYILKPQMPWELWDIIQEVSPEEIQPNPPSSGMLGIIIMMTLCDQVDIYEFL
PSKRKTDVCYYYQKFFDSACTMGAYHPLLFEKNLVKHLNQGTDEDIYLLGKATLPGFRRI
RC-------------
>Mmul_ST6Gal1
--------------------MIHTNLKKK-----------FSCCVLVFLLFAVICVWKE-
----------------KKKGSYYDSFKLQ--------------------TKEFQV-----
-----LKSLGKLAMGSDSQSVSSSSTQD------------------PHRGRQALGSLR--
------------------------------------------------------------
---------------------------DIPKAKPEAFFQVWNKDSSSKNLIPRLQKIWKN
YLSMNKYKVSYKGPGPGIKFSA---EALRCHLRDHVNVSMVEATDFPFNTSEWE-GYLPK
DNIR------TKAGPWGRCAVVSSAGSLKSSQLGREIDDHDAVLRFNGAPTANFQQDVGT
KTTIRLMNSQLVTTEKR-FLKDSLYNEGILIVWDPSVYHSDIPKWYQNPDYNFFNNYKSY
RKLHPDQPFYILKPQMPWELWDILQEISPEEIQPNPPSSGMLGIIIMMTLCDQVDIYEFL
PSKRKTDVCYYYQKFFDSACTMGAYHPLLYEKNLVKHLNQGTDEDIYLLGKATLPGFRTI
HC-------------
>Pabe_ST6Gal1
--------------------MIHTNLKKK-----------FSCCVLVFLLFAVICVWKE-
----------------KKKGSYYDSFKLQ--------------------TKEFQV-----
-----LKSLGKLAIGSDSQSVSSSSTQD------------------PHRGRQTLGSLR--
------------------------------------------------------------
---------------------------GLAKAKPEASFPVWNKDSSSKNLIPRLQKIWKN
YLSMNKYKVSYEGPGPGIKFSA---EALRCHLWDYVNVSMVEATDFPFNTSEWE-GYLPK
ESIR------TKAGPWGRCAVVSSAGSLKSSQLGREIDDHDAVLRFNGAPTANFQQDVGT
KTTIRLMNSQLVTTEKR-FLKDSLYNEGILIVWDPSVYHSDIPKWYQNPDYNFFNNYKSY
RKLHPNQPFYILKPQMPWELWDILQEISPEEIQPNPPSSGMLGIIIMMTLCDQVDIYEFL
PSKRKTDVCYYYQKFFDSACTMGAYHPLLYEKNLVKHLNQGTDEDIYLLGKATLPGFRTI
HC-------------
>Btau_ST6Gal1
--------------------MTRTSLKKKV----------FSCCVLIFLLFAIICVWKE-
----------------KKKGNYYEFLKLQ--------------------NKEYQV-----
-----LQGLEKLAVSSSSQPVSSSSTHN------------------PQRNIQALGGP---
------------------------------------------------------------
------------------------------KAKLKATFQVWDKDSSSKNLAPRLQTIRKN
YLNMNKYKVTYKGPGPGVKFSA---EALLCHLRDHVNISMIEASDFPFNTSDWE-GYLPQ
EDIR------SKAGPWGRCAVVSSAGSLKSSRLGREIDDHDAVLRFNGAPTVKFQQDVGT
KTTIRLVNSQLVTTEAG-FLKDSLYNEGILIVWDPSVYHSDIPKWYRNPDYSFFNNFKSY
RKLHPDQPFYILKPQMPWELWDIIQEISSELIQPNPPSSGMLGIAIMMSLCDQVDIYEFL
PSKRKTDVCYYYQRYFDSACTMGAYHPLLFEKNMVKYLNLGTDEDIYLLGKATLPGFRTI
RCGA-----------
>Mmus_ST6Gal1
--------------------MIHTNLKRK-----------FSCFVLVFLLFAIICVWK--
------------------KGSDYEALTLQ--------------------AKVFQM-----
-----PKSQEKVAVGPAPQAVFSNSKQD------------------PKEGVQILSYPR--
------------------------------------------------------------
---------------------------VTAKVKPQPSLQVWDKDSTYSKLNPRLLKIWRN
YLNMNKYKVSYKGPGPGVRFSV---EGLRCHLRDHVNVSMIEATDSPFNTTEWE-GYLPK
ETFR------TKAGPCTKCAVVSSAGSLKNSQLGREIDNHDAVLRFNGAPTDNFQQDVGT
KTTIRLVNSQLVTTEKR-FLKDSLYTEGILILWDPSVYHADIPQWYQKPDYNFFETYKSY
RRLHPSQPFYILKPQMPWELWDIIQEISPDLIQPNPPSSGMLGIIIMMTLCDQVDIYEFL
PSKRKTDVCYYHQKFFDSACTMGAYHPLLFEKNMVKHLNEGTDEDIYLFGKATLSGFRNN
RC-------------
>Rnor_ST6Gal1
--------------------MIHTNLKKK-----------FSLFILVFLLFAVICVWK--
------------------KGSDYEALTLQ--------------------AKEFQM-----
-----PKSQEKVAMGSASQVVFSNSKQD------------------PKEDIPILSYHR--
------------------------------------------------------------
---------------------------VTAKVKPQPSFQVWDKDSTYSKLNPRLLKIWRN
YLNMNKYKVSYKGPGPGVKFSV---EALRCHLRDHVNVSMIEATDFPFNTTEWE-GYLPK
ENFR------TKVGPWQRCAVVSSAGSLKNSQLGREIDNHDAVLRFNGAPTDNFQQDVGS
KTTIRLMNSQLVTTEKR-FLKDSLYTEGILIVWDPSVYHADIPKWYQKPDYNFFETYKSY
RRLNPSQPFYILKPQMPWELWDIIQEISADLIQPNPPSSGMLGIIIMMTLCDQVDIYEFL
PSKRKTDVCYYHQKFFDSACTMGAYDPLLFEKNMVKHLNEGTDEDIYLFGKATLSGFRNI
RC-------------
>Ttru_ST6Gal1
--------------------MIPTNLKKK-----------FSCCVLVFLLFAIICVWKE-
----------------KKKGSYYESLKLQ--------------------TKEFQV-----
-----LR--------SGSQSVSSSGTHN------------------PQRGSQALSGP---
------------------------------------------------------------
------------------------------KAKPEATFQVWNKDSSTKNLIPRLQKIWKN
YLSMNKYKVSYKGPGPGVKFSA---EALLCHLRDHVNVSMVEATDFPFNTSEWE-GYLPK
ENIR------TKAGPWGRCAVVSSAGSLKSSQLGREIDDHDAVLRFNGAPTANFQQDVGT
RTTIRLMNSQLVTTEGR-FLKDSLYNEGILIVWDPSIYHSDIPKWYKNPDYSFFDSFKSY
RKLHPDQPFYILKPQMPWELWDVIQEISPEQIQPNPPSSGMLGIIIMMTLCDQVDIYEFL
PSKRKTDVCYYYQKFFDSACTMGAYHPLLFEKNMVKHLNRGTDDDIYLLGKATLPGFRSI
RCG------------
>Ptro_ST6Gal1
--------------------MIHTNLKKK-----------FSCCVLVFLLFAVICVWKE-
----------------KKKGSYSDSFKLQ--------------------TKEFQV-----
-----LKSLGKLAMGSDSQSVSSSSTQD------------------PHRGRQTLGSLR--
------------------------------------------------------------
---------------------------GLAKAKPEASFQVWNKDSSSKNLIPRLQKIWKN
YLSMNKYKVSYKGPGPGIKFSA---EALRCHLRDHVNVSMVEVTDFPFNTSEWE-GYLPK
```

ESIR------TKAGPWGRCAVVSSAGSLKSSQLGREIDDHDAVLRFNGAPTANFQQDVGT
KTTIRLMNSQLVTTEKR-FLKDSLYNEGILIVWDPSVYHSDIPKWYQNPDYNFFNNYKTY
RKLHPNQPFYILKPQMPWELWDILQEISPEEIQPNPPSSGMLGIIIMMTLCDQLDIYEFL
PSKRKTDVCYYYQKFFDSACTMGAYHPLLYEKNLVKHLNQGTDEDIYLLGKATLPGFRTI
HC-------------
>Ggor_ST6Gal1
--------------------MIHTNLKKK-----------FSCCVLVFLLFAVICVWKE-
----------------KKKGSYYDSFKLQ--------------------TKEFQV-----
-----LKSLGKLAMGSDSQSVSSSSTQD------------------PHRGRQTLGSLR--

--------------------------GLAKAKPEASFQVWNKDSSS-NLIPRLQKIWKN
YLSMNKYKVSYKGPGPGIKFSA---EALRCHLRDHVNVSMVEATDFPFNTSEWE-GYLPK
ESIR------TKAGPWGRCAVVSSAGSLKSSQLGREIDDHDAVLRFNGAPTANFQQDXGT
KTTIRLMNSQLVTTEKR-FLKDSLYNEGILIVWDPSVYHSDIPKWYQNPDYNFFNNYKSY
RKLHPNQPFYILKPQMPWELWDILQEISPEEIQPNPPSSGMLGIIIMMTLCDQVDIYEFL
PSKRKTDVCYYYQKFFDSACTMGAYHPLLYEKNLVKHLNQGTDEDIYLLGKATLPGFRTI
HC-------------
>Ogar_ST6Gal1
--------------------MIHTNLKKK-----------FSCCVLAFLLFAVICLWKE-
----------------KKKGSFYNSFKLP--------------------AKEFQA-----
-----LKYLGKPAAGSESQSASLSSTQD------------------PHRGSQAPSGVR--

--------------------------GPAKAKPEASFFVWNKDTSSKNLIPRLQKIWKN
YLNMNKYKVSYKGPGPGVKFSA---EALHCQLRDHVNVSMVEATDFPFNTSLWE-GYLPK
ENIR------IKAGPWGRCAVVSSAGSLKSSQLGQEIDSHDAVLRFNGAPTANFQQDVGT
KTTIRLMNSQLVSTEKR-FLKDSLYNEGILIVWDPSVYHSDIPKWYQNPDYNFFDNYKSY
RKLHPNQPFYILKPQMPWELWDILQEVSPEKIQPNPPSSGMLGIIIMMTVCDQVDIYEFL
PSKRRTDICYYYQKFFDSACTMGAYHPLLFEKNLVKHLNQGTDEDIYLLGKATLPGFRTV
RC-------------
>Cpor_ST6Gal1
--------------------MIHTSLRKK-----------FSYCVLAFVLFAAICVWKE-
----------------RKKGSYYGSLKLQ--------------------TNEFQE-----
-----PKNLEKLTTGSGSQHVSSRSTQV------------------PHQLSRTL-SPR--

--------------------------VPHKTKPQPTFQVWNKDSSSKNLVPRLQKIWKN
YVRMNKYKVSYKGPGPGVKFSV---DALLCHLRDHVNVSMVDTTDYPFNTSEWE-GYLPR
ENIR------TKAGPWRRCAVVSSAGSLKSSQLGQEIDDHDAVLRFNGAPTANFQQDVGT
KTTIRLMNSQLVTTEGR-FLSDSLYNEGILIVWDPSMYHADIPKWYQNPDYSFFNNYKSY
RKLHPNQPFYILRPQMPWELWDILQEISPEEIQPNPPSSGMLGIIIMMTLCDQVDIYEFL
PSKRRTDVCYYHQKFFDMACTMGAYHPLLFEKNMVKYLNEGTDEDIYLLGKATLSGFRNL
RC-------------
>Tbel_ST6Gal1
--------------------MIHTNLKKK-----------FSCCVLAFLLFAVICVWKE-
----------------KKKGSYYDSLKLQ--------------------AKEFQV-----
-----LKSLEKLAIGSSSQTVSSSSTQD------------------PHR------SAK--

--------------------------DLVKAKPEASFQVWNKDSSSKNLIPRLQKIRKN
YLNMNKYKVSYKGPGPGVKFSA---EALRCHLRDHVNVSMVESTDFPFNTSEWE-GFLPT
ENIR------TKAGPWGKCAVVSSAGSLKSSQLGQEIDNHDAVLRFNGAPIANFQQDVGT
KTTIRLMNSQLVTTDKR-FLKDSLYSEGILIVWDPSVYHSDIAKWYKTPDYNFFNNYKSY
RKLHPNQPFYILKPQMPWELWDILQETAPEEIQPNPPSSGMLGIIIMLTLCDQVDIYEFL
PSKRKTDVCYYYQKFFDTACTMGAYHPLLFEKNLVKHLNEGTDEDIYLFGKATLSGFRTI
HC-------------
>Etel_ST6Gal1
--------------------MIQVILRKK-----------LSYCVLAFFLFAIICVWKE-
----------------RKKVGYYESLKLE--------------------TEGFQV-----
-----VSNQEKLLVGSGPQ--SSVGTQD------------------LHTGNPALSSTK--

--------------------------SSVKTKPPPSFQVWNKESSSRNLPSRLQSIRKN
YLNMNKYKVSYKGPGPGVKLTP---EALRCYLRDRVNVSMVEATDFPFNTSEWE-GYLPG
ENFR------TKAGPWGRCAVVSSAGSLKSSQLGQEIDDHDAVLRFNAAPTANFQQEVGT
KTTIRLVNSQLVTTAGR-FLKDSFYNEGIMIMWDPAXXHSDIPKWYKHPDYNFSSNYKSY
RRLHPEQPFYILRPQMPWELWDILQEISPEKIQPNPPSSGMLGIMIMMTLCDQVDVYEFL
PSKRKTDVCYYFQKFFDSACTMGAYHPLLFEKNMVKHLNVGTDEDIYLHGKATLLGLKNL
HC-------------
>Pvam_ST6Gal1
--------------------MIHTNLKKK-----------FSCCVLAFLLFAVICMWKE-
----------------KKKGSYYNTLKLE--------------------TKVVRS-----
-----LEKLS---IGFGSQPVPSSSTQD------------------LHRNSQALSSPK--

--------------------------ERVKTKLEPFFQVWNKDSSSKNLIPRLKKIWKN
YLSMNKYKVSYKGPGPGVKFSA---EALLCHLRDQVNVSMVEATDFPFNTSEWE-GYLPK
EKIR------TKAGPWGRCAVVSSAGSLKSSQVGQEIDNHDAVLRFNGAPTADFQQDVGT
KTTIRLMNSQLITTEGR-FLQDSLYNEGILIVWDPSVYHSDIPKWYQNPDYKFFDNYRSY
RKLHPDQLFYILKPQMPWELWDIIQEISPEEIQPNPPSSGMLGIIIMMTLCDQVDIYEFL
PSKRKTDMCYYYQKFFDSACTMGAYHPLLFEKNLVKHLNQGTDEDIYLLGKATLPGFRRI
HC-------------
>Eeur_ST6Gal1
--------------------MIHTSLKKK-----------FSYCVLFFLLFAVICVWKE-
----------------KKKGSDYNSLKLQ--------------------SKEVQV-----
-----LKNLA---MGSGSRSAASSSTRA------------------PPRASKALSSPR--

--------------------------GPAKAKLEPSYPVWNKESSSKNLNPRLQKIWKN
YQNMNKYKVAYKGPGPGVKFSA---KDLSCLLRNHVNVSMVEATDFPFNTSEWE-GYLPR
ENIR------TKAGPWGRCAVVSSAGSLKSSQLGREIDDHDAVLRFNGAPTANFQQDVGT
KTTVRLMNSQLVTTEKR-FLKDSLYNEGILIVWDPSVYHSDIPKWYQNPDYGFFDNYKTY
RKQNPHQPFYILKPQMPWELWDILQQIAPEEIQPNPPSSGMLGIIIMMTLCDQVDIYEFL
PSKRKTDVCYYYQKFFDSACTLGAYHPLLYEKNMVKHLNQGTDEDIYLLGKATLPGFRSI
QC-------------
>Dnov_ST6Gal1

```
--------------------MIHINLKKK-----------LSYCLLAFFLFGVICIWKE-
----------------MKKGGYYDSLKLE--------------------TKDFQV-----
-----LRSLER----LTTGSLSSSSTQD------------------PHGGSQAVSTLR--
------------------------------------------------------------
---------------------------SSAKAKPEFSFQVWNKDSSSKNLIPRLQKILRN
YLNMNKFKVSYKGPGPGVKFSA---EALYCHLRDHVNVSMVEATDFPFNTSEWE-GYLPS
ENIR------TKAGPWKKCAVVSSAGSLKSSQLGQEIDDHDAVMRFNGAPTANFQQDVGT
KTTIRLMNSQLVTTEKR-FLKDSFYNEGILIVWDPSVYHTDIPKWYQNPDYNFFNNYKSY
RKMHPDQPFYILKPQMPWELWDILQEVSPEAIQPNPPSSGMLGIIVMMTLCDQVDIYEFL
PSKRKTDVCYYYQKFFDSACTMGAYHPLLFEKNLVKHLNQGTDEDIYLHGKTTLPGFRSI
HC-------------
>Stro_ST6Gal1
--------------------MARCISKFGV----------ISIILCTFSFFLLDFYSKSK
CLESCTTVHGKVEVARDVKNNLNKALERENPLEREKD---------LEREKQLER-----
-----QKELEREKQLKRERELEREKQLERERELE---------REKQLEREKQLEREKQL
------EREKELERQKIAEAEHLHKIWLNQ------------------------------
------------------------RQGAQSRLRFANRYGVWNEHMSSKDILPRLVMVKKS
YQSENKYKVKYQG-KLGQLLTP---QELLCAIKTKVNISTLVTADLPQNADSWS-QYLPR
KTLQ------EEVKSLGKCAVVSSAGSIKHSRLGQEIDTHDAVLRFNAAPVLGYQDDVGT
KTTFRLLNSQLVSRPELKFLEDPLYKEGSLIMWDPAPYNANVEEWHKNPDYKFFERYSEY
RRRNPEQMFYVLNPHAAWQLWDIIQQNAPEDINPGPPSSGLLGILLMMNLCDQVNVYEFL
PSRRQSDLCHYYERYQDRACTLGAYHPLMYEKNLVKKLNLGDDNSIFHFGRITLPGLRGL
QC-------------
>Gacu_ST6Gal1
--------------------MDRVSLLWRLRRRARRGVLCMAFFCISMALLYAICAENS-
--------------VPVTDAIFGVRARTRAQPRAHS------------VVKVLRG--GAK
PMYIDPQKLPGVVPGDPQRPIPVLSSPNQTRD-----------AVDSPAKRKLKERDPPG
-FFARLLPRPFTRALETLFGGRRRGELSGR------------------------------
------------------------VGEAEFFGPHGLLGEVWDDEMSSSMLGSRLRKVVQN
YQAMNKYGVEFSGPGGVSSRPKLSGPKLLCQLRDKVKVTTLTNDLEPFSSLSWA-VQLPP
NTLT------SDLGPYRSCAVVSSAGSLRNSGLGKEIDSHDAVLRFNAAPTTGYEKDVGS
KTTIRLINSQVMASDDHRFLSSSLYSSGVLVAWDPAPFSADLTQWYNRTDYPIFTQYQRY
RKLHPMQPFYILHPRFEWQVWQRIQDNMAEPIQKNPPSSGLLGTVLMMSLCEVVHVYEFL
PSRRKTELCHYYQRFHDAACTLGAYHPLLYEKNLVKRMNRGPDRDIYTHGRVTLPGFGKM
NCTEAAGGSTSR---
>Tnig_ST6Gal1
--------------------MDRVSLLWRLRRRARRGALCMAFFCIAMALLYAICAENS-
--------------VPVTDAIFGVRARTRAQPRSHS------------VIKVLRG--GAK
PVYTDPQKLPGIIPGDPHRPIPVLSSLNHTLE-----------SWEPPSKQKPRERPPAG
-FLSGLLPRPFTRALETLFGGRRKGELSGR------------------------------
------------------------G-DAELFGPQGILKEVWDDETSVSMLGNRLKKVVQN
YQAMNKYGVKVSGPGGVASRAKLSGPKLLCRLKAEVDVSTVTSDLQPFSSLPWA-SQLPS
KQLT------SDLGPFRSCAVVSSAGSLRSSGLGKEIDAHDAVLRFNAAPTSGFENDVGS
KTTIRLVNSQVMASDAHRFLSSSLYSSGTLVAWDPAPFSADLTQWYNRTDYPIFTQYQRY
RMLHPLQPFYILQPRFEWQLWQQIQDNMAEPIQKNPPSSGLLGTVMMMSLCEVVHVYEFL
PSRRRTEFCHYYQRFYDAACTLGAYHPLLYEKNLVKRMNQGPDRDIYTHGRVTLPGFNRL
NCTGDAKGVPQGP--
>Trub_ST6Gal1
--------------------MDRVSLVWRLRRRARRGVLCMAFFCISMALLYAICAENS-
--------------VPVTDAIFGVRARTRAQPRAHS------------VIKVLRG--GAK
PMYIDPQKLPGIIPGDPHRPIPVLSSLNHTLE-----------SWDPPAKPKPKERTPSG
-FFSHLLPRPFTRALETLFGGRRKGELSGR------------------------------
------------------------GGDAALFGPRGILGEVWDDEMSSSMLGNRLKKVVQN
YQAMNKYGVKVSGPGGVSSRPKLSGPKLLCQMKIQVDVSTLTSDFQPFSSLPWA-SQLPS
KQLT------SNLGPYKSCAVVTSAGSMRSSGLGKEIDSHDAVLRFNAAPTSGYENDVGS
KTTIRLVNSQVMASEAHRFLSSSLYSSGTLVAWDPAPFSADLTQWFNRTDYPIFTQYQRY
RMLHPMQPFYILHPRFEWQVWQRIQDNMAEPIQKNPPSSGLLGTVMMMSLCEVVHVYEFL
PSRRKTELCHYYQRFFDAACTLGAYHPLLYEKNLVKRMNQGPERDIYTHGRITLPGFNTL
NCTGDAGGALVDMRH
>Drer_ST6Gal1
--------------------MDRVSLLWRLRRRARRGALCMALFCLTMALLYTLCAENS-
--------------IPVTDAIFGVKARTRAQPRAHT------------IVKVLRGTGGSK
PMYTDPQKLPGVIPGDPQKPIPILSSSNYSMES-----------TSKDHSLGGKKR-ERG
-LFYWLLAQPLT-----IFGGRRRGDMGTA------------------------------
------------------------IRDADVFKPNGALGEVWNEEMSSSMLGKRLRKVVQN
YQAMNKYGVKYPATVRAAHRYKLSGPEILCEIKEKVQVTTLTPDMEPFSGFPWG-SQLPP
RQIT------SDVGPFKTCAVVSSAGSLKNSGLGKEIDSHDAVIRFNAAPTAGFETDVGS
KTTVRLINSQLMASEDHHFLSSSLYSAGILVSWDPSPYSSDLWEWFNKTDYPIFKQYQRY
RRLHPQQPFYIVHPRMEWQLWQRIQDNMGEAIQKNPPSSGLLGTVLMMSLCEVVHVYEFL
PSRRKTELCHYYQRFSDAACTLGAYHPLLYEKNLVKRMNQGSDRDIYTLGRVTLPGFATF
NCTSSTHSKT-----
>Olat_ST6Gal1
MDAELFSRTFLLPLQIPGAAVDRVSLLWRLRRRARRGAMCMAFFCLCMALLYAICAENS-
--------------VPVTDAIFGVRARTRAQPRAHS------------VTKVLRG--GAK
HMPIDPQKLPGVVPGDPRRPIPVLSSLNRSHEG-----------RTSAPKQTSRTSGSSG
-FLASFLPRPLSRAIQTLFTGRRRGELSGK------------------------------
------------------------AREAEFFGPHGLLGEVWDDEMSSSRLSSRMKKVVQN
YQAMNKYGVKLSGPAGGTRRLKLSGPELLCQLKRKVEVLSLTSDLHPFSMLPWA-PQLPA
QQLT------SELGPLKSCAVVSSAGSLRHSGLGKEIDSHDAVMRFNAAPTSGFEKDVGS
KTTMRLINSQVMASEEYRFLSSSLYSSGVLVAWDPAPFSSDLTQWLNRTDYPIFAQYQRY
RRLHPQQPFFILHPRFEWQVWQQVQENMAESIQKNPPSSGFLGTVLMMSLCEVVHVYEFL
PSKRKTELCHYYQHFYDAACTLGAYHPLLYEKNLVKRMNQGADRDIYTHGRVTLPGFNAI
NCTRAAQEPEGM---
>Amex_ST6GAL1
-----------MGYKIPGAAMDRVSLLWRLRRRARRGALCMALFCMTMAVLYTLCAENS-
--------------VPVTDAIFGVRARTRAQPRAHS------------IVKVLRG-AGAK
PMYVDPQKLPGVIPGDPQKPIPVLSSSTHSLES-----------SHKEHSFRSRDREPHG
-LLSWLVGRPLRRALETILGGRRKSDSLQK------------------------------
------------------------G-DAELFGTHGALGEIWDDEMSSTMLGKRLKKVVQN
YQAMNKYGVKFSGSEGATRRRKLSGPELLCQLKDMVHITTLTPDMEPFAGFPWA-SQLPP
```

```
QPLS------SDLGPFKTCAVVSSAGSLRNSGLGKEIDSHDAVVRFNAAPTAGFEKDVGS
KTTVRLINSQVMASEDHHFLSSSLYSIGTLVAWDPAPYSSDLNEWYNKTDYPIFKQYQRY
RRLHPQQPFYILHPSVEWQLWKRIQDNMAEPIQKNPPSSGLLGTVLMMSLCDVVHVYEFL
PSRRKTELCHYYQRFSDAACTLGAYHPLLYEKNLVKRMNQGSDRDIYTHGRVTLPGFSTY
NCTASILSKP-----
>Char_ST6GAL1
--------------------MDRVSQLWRLRRRARRGALCMALFCMSMAVLYTLCAENS-
--------------VPVTDAIFGERARTRAQPRTHS------------LVKVLRG-GASK
PIYVDLQKLPGVIPGDPRRPIPVLSSLNHSLET-----------DHRDHRLRPKDRKPHG
GFMSWLP-RPLTRAIETLFGGRRKGELSGA------------------------------
------------------------GSDTEFFGPNGTLGEVWDDEMSSTMLGKRLRKVVQN
YQAMNKYGVQFPDVGAAARHPKRSGPELLCQLKDTVSITTLTPEMEPFVRFPWA-TQLPP
RHLS------MDIGPFKTCAVVSSAGSMKNSGLGKEIDAHDAVVRFNAAPTTGFEKDVGS
KTTIRLINSQVMASDDHRFLSSSLYSNGILVGWDPAPYSSDLNEWYNKTDYPIFLQYQRY
RRLHPQQPFYILHPRVEWQLWNRIQENMAEPIQKNPPSSGLLGTVLMMSLCEVVHVYEFL
PSRRKTELCHYYQRFIDAACTLGAYHPLLYEKNLVKRMNQGLDRDIYTHGRVSLPGFSSF
NCTRGLPSAMH----
>Mzeb_ST6GAL1
----------MAYKIPAAAMDRVSLLWRLRRRARRGVLCMTFFCISMALLYAICAENS-
--------------VPVTDAIFGVRARTRAQPHAHN------------VIKVLRG--GAK
PMYTDPQKLPGVIPGDPRRPIPILSSSNHSHE-----------ARDSSSKRK--QREPPG
-FFSRLLPRPFTRALETLFGGRRKGELSGR------------------------------
------------------------GSDAEFFGPNGLLGEVWDDEMSSSMLGTRMKKVVQN
YQAMNKYGVELSGPGGVSGTPKLSGPELLCQLKDKVEVATLTPDLQPFSLLPWA-TQLPP
QQLT------SDLGPYKSCAVVSSAGSLRYSGLGKEIDSHEAVLRFNAAPTTGYEKDVGS
KTTIRLINSQVMASDDHRFLSSSLYSSGVLVAWDPAPFSADLTKWFNRTDYPIFAQYQRY
RRLHPLQPFYILHPRFEWQIWQRIQDNMAEPIQKNPPSSGMLGTVLMMSLCEVVHVYEFL
PSRRKTELCHYYQRFYDAACTLGAYHPLLYEKNLVKRMNQGSDRDIYTSGRVTLPGFRKL
NCSQTAGGDR-----
>Ssal_ST6GAL1
----------MGYKYPGAAMDRVSLLWRLRRRARRGALCMGLFCISMAMLYALCAENS-
--------------VPVTDAIFGVRARTRAQPRAHS------------IIKVLRG--GAK
PMYLDPQKLPGIIPGNAHRPIPVLSSPNHTQEVT---------AKETHSKHPKRERESSG
-FFAWLLPRPFRRAMETLFGGRRRGELSGT------------------------------
------------------------VGDAEFFGPHRALGEVWDDEMSSNMLGKRLRKVVQN
YQAMNKYGVEFSGPGGVATRPKVSGSELLCQLKYKVEVATLTPDLEPFSSLPWA-YQLPL
QPIT------SDLGPYKSCAVVSSAGSLRYSGLGKEIDSHDAVLRFNAAPTVGYEKDVGS
KTTIRLLNSQVMASDDHRFLSSSLYSTGVLVAWDPSPFSADLSQWYNKTDYPIFTQYQRY
RRLHPLQAFYILHPSFEWQLWQRIQDNMAEPIQKNPPSSGLLGTVLMMSLCEVVHVYEFL
PSQRKTELCHYYQRFYDAACTLGAYHPLLYEKNLVKRMNQGLDRDIYTHGRVTLPGFSTL
NCTRGG---------
>Spar_ST6GAL1
--------------------MDRVSLLWRLRRRARRGALCMAFFCISMALLYALCAENS-
--------------VPVTDAIFGIRARTRAQPRAHS------------VIKVLRG--GAK
PMYIDPQKLPGVVPGDPHRPIPVLSSPNHTHD-----------GGESPSKRRSREREPSG
-FFSRLLPRPFTRALETLFGGRRRGELSGR------------------------------
------------------------GGDAEFFGPHGLLGEVWDDEMSSSMLGSRLRKVVQN
YQAMNKYGVQLSGPGGVSSRPKLSGPELLCQLKDNVEVMTLTADLQPFSALPWA-AQLPP
KPLT------SDLGPYKSCAVVSSAGSLRYSGLGKEIDSHDAVLRFNAAPTAGYEKDVGS
KTTIRLINSQVMASDDHRFLSSSLYSSGVLVAWDPAPFSADLTQWYNRTDYPIFTQYQRY
RRLHPLQPFYILHPRFEWQVWQRIQDNMAEPIQKNPPSSGLLGTVLMMSLCEVVHVYEFL
PSRRKTELCHYYQRFYDAACTLGAYHPLLYEKNLVKRMNQGSDRDIYTHGRVTLPGFSRL
NCTQAAGGEPDH---
>Lcro_ST6GAL1
--------------------MDRVSLLWRLRRRARRGVLCMAFFCISMALLYALCAENS-
--------------VPVTDAIFGVRARTRAQPRAHS------------VIKVLRG--GAK
PMYIDPQKLPGVVPGDPHRPIPVLSSPNHTHE-----------AVDSTPKRKPREREPSG
-FLSRMLPRPFTRALETLFGGRRKGELSGR------------------------------
------------------------GSDAEFFGPHGLLGEVWDDEMSSSMLGNRLRKVVQN
YQAMNKYGVEFSGPGGVSSRPKLSGPKLLCQIKDTVEVTTLTADLQPFSLLPWA-AQLPP
KQLT------SDLGPYKSCAVVSSAGSLRYSGLGREIDTHDAVLRFNAAPTTGYEKDVGS
KTTIRLINSQVMASDDHRFLSSSLYSSGALVAWDPAPFSADLTQWYNRTDYPIFTQYQRY
RRLHPMQPFYILHPRFEWQVWQRIQDNMAEPIQKNPPSSGLLGTVLMMSLCEVVHVYEFL
PSRRKTELCHYYQRFFDAACTLGAYHPLLYEKNLVKRMNQGSDRDIYTHGRITLPGFSKM
NCSQAAGGVPNH---
>Alim_ST6GAL1
----------MGYKIPGAAMDRVSLLWRLRRRARRGAVCMVFFCISMALLYAICAENS-
--------------VPVTDAIFGVRARTRAQPRAHS------------VIKVLRA--GGK
TLYIDPQKLPGVIPGDPHRPIPVLSSMNRTLE-----------DLDSPPKRAVKVREPSG
-FFARILPRPFTRALETLFGGRRRKGELSGK-----------------------------
------------------------SGDXEFFGPHGLLGEVWNDEMSSSMLGSRMKKVVQN
YQVMNKYGVKFSGPGGVSTRLKLTGPELLCQLKKKVEVSTLTSDLEPFSVLPWA-TQLPV
NQMT------SDLGPYKSCAVVSSAGSLRNSGLGKETDSHDAVVRFNGAPTIGFEKDVGS
KTTIRLINSQVMATDEHRFLSSSLYSSGVLVAWDPAPFSSDLTQWYNRTDYPIFAQYQRY
RRLHPQQPFYILHPRFEWQLWQRIQDNMAEPIQKNPPSSGFLGTVLMMSLCEVVHVYEFL
PSRRKTELCHYYQRFFDAACTLGAYHPLLYEKNLVKRMNLGSDRDIYMDGRVTLPGFRXM
NCTSDGVPEH-----
>Pfor_ST6GAL1
---------MLFKCDIPGAAMDRVSLLWRLRRRARRGAICMTFFCISMALLYALCAENS-
--------------VPVTDAIFGVRARTRAQPRTHS------------VIKVLRG--GAK
SIYADPQKHPGIIAGDPHRPIPVLSSPNHTNE-----------DWESPPKRATKAKESSG
-FFSHLLPGPFTRALETLFVGRRKGELSGK------------------------------
------------------------SGDSEFFGSKGILGDVWNDEMSSSMLGNRLKKVVQN
YQAMNKYGVKVSGPGGTSNRLKLSGRELLCQLKEKVEVTTLTSDHQPFSALAWAPLLLPP
QQLT------SDLGPYKSCAVVSSAGSLRNSGLGKEIDTHDAVLRFNAAPTSGFEKDVGS
KTTVRLINSQVMASDDHRFLSSSLYSSGVLVAWDPAPFFSNLTQWYNHTDYPIFNQYQRY
RRLHPQQPFYILHPRLEWQLWQRIQDNMAEPIQKNPPSSGLLGTILMMSLCEVVHVYEFL
PSRRKTPLCHYYQRFYDAACTFGAYHPLLYEKNLVKRMNQGSDRDIYNHGRVTLPGFSAM
NCTAEAVQEH-----
>Cvar_ST6GAL1
```

```
--------------------MDRVSLLWRLRRRARRGAVCMTLFCISMALLYALCAENS-
--------------VPVTDAIFGVRARTRAQPRTHS------------VIKVVRG--GAK
TPNVVPQKHP----NDPHRPIPVLS--NHTNE-----------DLESPPKRTS-AKESSG
-FFARLLPRPLTRVLAGLFSGRRKGELSGK------------------------------
------------------------SGDAEFFGPKGLLGEVWNEEMSSSMLGGRLQKVVQN
YQAMNKYGVKVSGPGGTSKRTKLSGHELLCQLKEKVQLSTLTPDFQPFSAMAWASLLLPP
QQLS------SDLGPYKSCAVVSSAGSLRNSGLGKEIDSHDAVIRFNAAPTSGFEKDVGS
KTTIRLINSQVMANEDLRFLSSSLYSSGVLVAWDPSPFSSDLSQWYNHTDYPIFSQYQRY
RRLHPQQPFYILHPRFEWQLWQRIQDNMAEPIQKNPPSSGLLGTVLMMSLCEVVHVYEFL
PSRRKTELCHYYQRFYDAACTLGAYHPLLYEKNLVKRMNQGSDQDIYVHGRVTLPGFRSL
NCTAGMVRER-----
>Locu_ST6GAL1
--------------------MDRVSVLWRLRRRVKRGFVCMALFCVAMGFLYAFCAENS-
--------------VTATDAIFGTKARTRAQLRHHWP---------G-VERVLRG-----
-----ASGNPGSSVKQGRSP-PSLPASNTSAK----------------APPHAVKGDGHG
-FLSRVVTPFLGHLLEIVRGTGQKKE----------------------------------
------------------------GAVGDFFRGQASLGQVWNEEMSTSMLSKRLKKVVQN
YQAMNKYGVHFERLAS-RRRQRLTGEELLCELKDKVAVTSLTPDLPPFSELSWG-SQLPP
HLVT------AHLGPYKSCAVVSSAGSLKNSGLGREIDSHDAVLRFNAAPTGGYEKDVGT
KTTVRLINSQVMASEDHHFLSSSLYSAGTLVAWDPAPFSSNLAEWYNKTDYPIFKQYQKY
RLLHPQQPFYILHPKVEWQLWERIQENMAEPIQKNPPSSGLLGTVLMMSVCEVVHLYEFL
PSRRKTELCHYYQRFFDAACTLGAYHPLMYEKNLVKRMNRGTDKEIYQYGRVTLPGLATL
NCTAPRVH-------
>Onil_ST6GAL1
-----------MAYKIPGAAMDRVSLLWRLRRRARRGALCMTFFCISMALLYAICAENS-
--------------VPVTDAIFGVRARTRAQPHAHN------------VIKVLRG--GAK
PMYTDPQKLPGVIPGDPRRPIPILSSSNHSHE-----------ARDSSSKRK--QREPPG
-FFSRLLPRPFTRALETLFGGRRKGELSGR------------------------------
------------------------GSDAEFFGPNGLLGEVWDDEMSSSMLGTRMKKVVQN
YQAMNKYGVELSGPGGVSGTPKLSGPELLCQLKDKVEVATLTPDLQPFSLLPWA-TQLPP
QQLT------SDLGPYKSCAVVSSAGSLRYSGLGKEIDSHEAVLRFNAAPTTGYEKDVGS
KTTIRLINSQVMASDDHRFLSSSLYSSGVLVAWDPAPFSADLTKWFNRTDYPIFTQYQRY
RRLHPLQPFYILHPRFEWQIWQRIQDNMAEPIQKNPPSSGMLGTVLMMSLCEVVHVYEFL
PSRRKTELCHYYQRFYDAACTLGAYHPLLYEKNLVKRMNQGSDRDIYTNGRVTLPGFRKL
NCSQTAGGDR-----
>Hbur_ST6GAL1
-----------MAYKIPAAAMDRVSLLWRLRRRARRGVLCMTFFCISMALLYAICAENS-
--------------VPVTDAIFGVRARTRAQPHAHN------------VIKVLRG--GAK
PMYKDPQKLPGVIPGDPRRPIPVLSSSNHSHE-----------ARDSSSKRK--QREPPG
-FFSRLLPRPFTRALETLFGGRRKGELSGR------------------------------
------------------------GSDAEFFGPNGLLGEVWDDEMSSSMLGTRMKKVVQN
YQAMNKYGVELSGPGGVSGTPKLSGPELLCQLKDKVEVATLTPDLQPFSLLPWA-TQLPP
QQLT------SDLGPYKSCAVVSSAGSLRDSGLGKEIDSHEAVLRFNAAPTTGYEKDVGS
KTTIRLINSQVMASDDHRFLSSSLYSSGVLVAWDPAPFSADLTKWFNRTDYPIFAQYQRY
RRLHPLQPFYILHPRFEWQIWQRIQDNMAEPIQKNPPSSGMLGTVLMMSLCEVVHVYEFL
PSRRKTELCHYYQRFYDAACTLGAYHPLLYEKNLVKRMNQGSDRDIYTNGRVTLPGFRKL
NCSQTAGGDR-----
>Pnye_ST6GAL1
-----------MAYKIPAAAMDRVSLLWRLRRRARRGVLCMTFFCISMALLYAICAENS-
--------------VPVTDAIFGVRARTRAQPHAHN------------VIKVLRG--GAK
PMYTDPQKLPGVIPGDPRRPIPVLSSSNHSHE-----------ARDSSSKRK--QREPPG
-FFSRLLPRPFTRALETLFGGRRKGELSGR------------------------------
------------------------GSDAEFFGPNGLLGEVWDDEMSSSMLGTRMKKVVQN
YQAMNKYGVELSGPGGVSGTPKLSGPELLCQLKDKVEVATLTPDLQPFSLLPWA-TQLPP
QQLT------SDLGPYKSCAVVSSAGSLRYSGLGKEIDSHEAVLRFNAAPTTGYEKDVGS
KTTIRLINSQVMASDDHRFLSSSLYSSGVLVAWDPAPFSADLTKWFNRTDYPIFAQYQRY
RRLHPLQPFYILHPRFEWQIWQRIQDNMAEPIQKNPPSSGMLGTVLMMSLCEVVHVYEFL
PSRRKTELCHYYQRFYDAACTLGAYHPLLYEKNLVKRMNQGSDRDIYTNGRVTLPGFRKL
NCSQTAGGDR-----
>Xmac_ST6GAL1
-----------MGYKIPGAAMDRVSLLWRLRRRARRGAICMTFFCISMALLYALCAENS-
--------------VPVTDAIFGVRARTRAQPRTHS------------VVKVLRG--GAK
SIYADPQKHPGIIAGDPHRPIPVLSSPNHTNE-----------DWASPPKRAAKMKESSG
-FFSHLLPGPFTRALETLFVGRRKGELSGK------------------------------
------------------------SGDSEFFGSKGILGDVWNDEMSSSMLGNRLKKVVQN
YQAMNKYGVKVSGPGGSSNRPKLSGRELLCQLKEKVAVTTLTSDHEPFSALAWAPLLLPP
QQLT------SDLGPYKSCAVVSSAGSLRNSGLGKEIDTHDAVLRFNSAPTSGFEKDVGS
KTTIRLINSQVMASDDHRFLSSSLYSSGVLVAWDPAPFSSNLSQWYNHTDYPIFNQYQRY
RRLHPQQPFYILHPRFEWQLWQRIQDNMAEPIQKNPPSSGLLGTILMMSMCEMVHVYEFL
PSRRKTPLCHYYQRFYDAACTFGAYHPLLYEKNLVKRMNQGSDRDIYNHGRVTLPGFSAM
NCTAEAVQEH-----
>Ggal_ST6Gal1
--------------------MVHINVLKK-----------FMCVLVVILIALTVCLWKE-
----------------TRGS-YYVPLK----------------------NDGTQV-----
-----HRALDKWNLLKSQGLLHEAAGEMGQMPKA---------LPNNQNKVKGITSGA--
------------------------------------------------------------
---------------------------VEKSRKAAEHVKVWDKDSSSRNLIPRLQKVRKN
YLSMNKYNVTYNGKMNAAKLSP---EQLLCRLRDRVNVTMIRGSDGPFNSSEWQ-HYLPD
KSLN------ETVGRLGRCAVVSSAGSLKSSHLGPEIDSHDAVLRFNGAPVKGFQEDVGQ
KTTIRLVNSQLVTVEEQQFLKDALYNTGILIVWDPAPYHAEIHEWYRKPDYKFFEAYKSY
RIRHPEQPFYILNPKMQWQLWDILQENSLEHIQPNPPSSGMLGIVIMMTLCDEVDVYEFL
PSKRQTDICHYYQKFHDHACTMGAYHPLLFEKNLVKHLNQGTDEDIYTHGKVTLPGFRNV
HC-------------
>Mnub_ST6GAL1
--------------------MVHINVLKK-----------LTCALVVILVALTVCLWRE-
----------------ARRS-YYVHFKTE--------------------NDDLQA-----
-----RRTSEKRNLLKSQGLLGEADSELGQIPKT---------WSGDHNKVKGSTSGT--
------------------------------------------------------------
---------------------------AEKFKKAGDFVKVWDKDSSSRNLIPRLQKVRKN
YLSMNKYNVTYNGKRNTAKLSP---EKLLCQLRDRVNVTMIQGSDDPFDTSEWQ-QYLPG
```

```
KSLS------LTVGHLGRCAVVSSAGSLKSSHLGQEIDSHDAVLRFNGAPTKGFEEDVGQ
KTTVRLVNSQLVTVEEQQFLKEALYNTGILIVWDPAPYHAEIHEWYRKPDYNFFESYKLY
RSMHPEQPFYILNPKMQWQLWDILQENSMEHIQPNPPSSGMLGILIMMTLCDEVDVYEFL
PSKRQTDICHYYQKFHDRACTMGAYHPLLFEKNLVKHLNQGTDEDIYTRGKVTLPGFRNV
HC-------------
>Svul_ST6GAL1
-----------MSHLDAPCTMVHINVLKK-----------FVCVIVVILVALTVCLWRE-
----------------TRRS-YYDPLKTR--------------------NDDFQG-----
-----HRSLEKWNSVKSQGLFREAASELGQVSKT---------WLASQNKG------T--
------------------------------------------------------------
---------------------------AEKSKKAAFG-KVWDKDSSSRNLIPRLQKVRKN
YLAMNKYNVTYNGKRG-AKLSP---EKLLCQLRDRVNVTMIQRSDGPFGTSEWQ-QYLPG
KSLS------ETVGHLGHCAVVSSAGSLKSSRLGQEIDSHDAVLRFNGAPVRGFQDDVGQ
KTTIRLVNSQLVTVEEQQFLRESLYNTGILIVWDPAPYHAEIHEWYRKPDYNFFESYKAY
RRTHPEQPFYILNPKMQWQLWDILQENSLEHIQPNPPSSGMLGIVLMMTLCDEVDVYEFL
PSKRQTDICHYYQKFHDHACTMGAYHPLLFEKNLVKHMNQGTDEDIYTHGKVTLPGFRKV
HC-------------
>Fche_ST6GAL1
--------------------MVHINVLKK-----------LMCVLVVVLVALTVCLWRE-
----------------TRRS-YYISFKTG--------------------NDDLQV-----
-----HRTLEKWNPLKSQGFFREAAGELGEMPKV---------WFGNHNKVKSSTSGM--
------------------------------------------------------------
---------------------------AEKSKKAADSVKVWDKDSSSRNLIPRLQKVRMN
YLSMNKYNVTYTGKRNAAKLSP---EKLLCQLRDRVNVTMIQGSDGPFGTSEWQ-QYLPG
KSLN------ETVGSLGRCAVVSSAGSLKSSHLGQEIDTHDAVLRFNGAPISGFQEDVGQ
KTTIRLVNSQLVTVEEQQFLREALYNTGILIVWDPAPYHAEIHEWYRKPDYNFFESYKSY
RSAHPEQPFYILNPKMQWQLWDILQENSLEHIQPNPPSSGMLGIVIMMTLCDEVDVYEFL
PSKRQTDICHYYQKFHDRACTMGAYHPLLFEKNLVKHINQGTDEDIYTHGKVTLPGFRKV
HC-------------
>Cliv_ST6GAL1
--------------------MVHINALKK-----------LMCVLVVILVALTVCLWRE-
----------------TRRS-YYVPFKTE--------------------NDDLQV-----
-----HRTSEKWTSLKSQGLFHEAVSELGHIPQT---------SFGNHNKVKGSTSGT--
------------------------------------------------------------
---------------------------AEKFKKAADSVKVWDKDSSSRNLIPRLQKVRIN
YLSMNKYNVTYNGKRNTAKLSP---EKLLCQLRDRVNVTMIEGSDGPFDTSEWQ-QYIPG
KSLN------ETVGRLGRCAVVSSAGSLKSSHLGQEIDSHDAVLRFNGAPIKGFQEDVGQ
KTTIRLVNSQLVTVEEQQFLREELYNTGILIVWDPAPYHAEIHEWYRKPDYDFFESYKLY
RSIHPEQPFYILNPKMQWQLWDILQENSLEPIQPNPPSSGMLGIVIMMTLCDEVHVYEFL
PSKRQTDICHYYQKFHDRACTMGAYHPLLFEKNLVKHINQGTDEDIYMHGKVTLPGFRNV
HC-------------
>Falb_ST6GAL1
-----------MSHLDAPCTMVHINVLKK-----------FMCVLVVILVALTVCLWRE-
----------------TRRS-YYDPLKTR--------------------NDDLQG-----
-----RRSLEKWSPVKSQGLFREAASELGQVPKM---------WLATQNKGT---SET--
------------------------------------------------------------
---------------------------AEKSKKAAFG-KVWDKDSSSRNLIPRLQKVRKN
YLAMNKYNVTYNGKRS-AKLSP---EKLLCQLRDRVNVTMIQRSDGPFGTSEWQ-QYLPG
KSLS------ETVGHLGRCAVVSSAGSLKSSRLGHEIDSHDAVLRFNGAPVRGFQDDVGQ
KTTIRLVNSQLVTVEEQQFLREPLYNTGILIVWDPAPYHAEIHEWYRKPDYNFFESYKAY
RRTHPEQPFYILNPKMQWQLWDILQENSLEHIQPNPPSSGMLGIVLMMTLCDQVDVYEFL
PSKRQTDICHYYQKFHDHACTMGAYHPLLFEKNLVKHMNQGTDEDIYTHGKVTLPGFRQV
QC-------------
>Gfor_ST6GAL1
--------------------MVHINVLKK-----------FMCVLVVVLVALTVCLWRE-
----------------TRRS-YYDPLKTG--------------------NDDFQG-----
-----HRSLEKWNTVKSQGLFHEAASELGQVSKI---------WLGTQNKVKGSTSET--
------------------------------------------------------------
---------------------------AEKSKKAAFG-KVWDKDSSSKNLIPRLQKVRKN
YLAMNKYNVTYNGKRN-AKLSP---EKLLCQLRDRVNVTMIQRSDGPFGTSEWL-QYLPG
KSFS------EAVGHLGRCAVVSSAGSLKSSRLGQEIDSHDAVLRFNGAPVRGFQDDVGQ
KTTIRLVNSQLVTVEEQQFLSEPLYNTGILIVWDPAPYHAEIHEWYRKPDYNFYESYKAY
RRAHPEQPFYILNPKMQWQLWDILQENSLEHIQPNPPSSGMLGIVLMMTLCDQVDVYEFL
PSKRQTDICHYYQKFHDHACTMGAYHPLLFEKNLVKHMNQGTDEDIYTLGKVTLPGFRKV
Q--------------
>Tgut_ST6Gal1
--------------------MVHINVLKK-----------FMCVLVVVLVALTVCLWRE-
----------------TRRN-YYDPLKTR--------------------NDDLQG-----
-----HRSLEKWNTVKSQGLFHEAASELGQVSKT---------WLGTQNKVKGSTSET--
------------------------------------------------------------
---------------------------DEKSKKAVFG-KVWDKDSSSRNLIPRLQKVRKN
YLAMNKYNVTYNGKRN-AKLSP---EQLLCQLRDRVNVTMIQRSDGPFGTSEWQ-QYLPG
KSLS------EAVGHLGCCAVVSSAGSLKSSHLGQEIDSHDAVLRFNGAPVRGFQDDVGQ
KTTIRLVNSQLVTVEEQQFLREPLYNTGILIVWDPAPYHAEIREWYRKPDYNFFESYKAY
RRAHPEQPFYILNPKMQWQLWDILQENSLEHIQPNPPSSGMPGIVLMMTLCDQVDVYEFL
PSKRQTDICHYYQKFHDHACTMGAYHPLLFEKNLVKHMNQGTDEDIYTHGKVTLPGFRKV
HC-------------
>Pbiv_ST6GAL1
---------------MPSKSMRKIFQGIQTSSEKNR---TLQYLSAAGSVNRNLEVTTQ-
--------------ALAEDSFFSRIRKFWQR-----------------LEKVFAG-----
-----FQQLR----RTSHSSLP--------------------------------------
------------------------------------------------------------
--------------------------------AQPLPWPAWQKELSSRVLGPRLQQVFQN
YQAMNKYRVP-SGAGQRPGNPHLTGMELLCQLKNRVNVSTVLGNEGPFAEPEWR-GMLPQ
RSLA------EDLGHLETCAVVSSAGSMLGSGLGSEIDSHDAVLRFNGAPTTGYEHDVGT
KTTLRLVNSQLMASPEQHFLDGQLYAQGTLVAWDPAPYPGDLEEWYKTPDYPLFEPFRTL
RANRPWQLFHLLHPRLQWELWQLVQEGADEQVQRNPPSSGLLGTVLMMSLCELVHVYEFL
PSQRQSHQCHYYQDFSDDACTLGAYHPLLFEKNLVRRMNRGTQADLAQRGRVTLPGFQVL
NCTQKD---------
>Acar_ST6Gal1
```

```
--------------------MVHVSSFRK-----------CIYVLLAFILVLTICLWKE-
----------------TRKG-VIFSLRLE--------------------GRSDHI-----
-----PWKMWRWGTSKEEPVQEVAVIATGKVP------------LLSNTEVN-----L--
------------------------------------------------------------
---------------------------STKVTRSNLIIKVWNKDSTSQSLHPRLQKARQT
YLKINKYKVNYSGPKHAHKLRP---KELLCQLRNRLDFRMITATDSPFDISEWE-PYVPK
GNIS------IKLG---RCAVVSSAGSMKSSHLGEDIDSHDAVLRFNGAPIKGFQADVGE
KTTIRLVNSQLITVEEKKFLADPQYNFGTLILWDPAPYHSSIKEWYKKPDYNFYGSFKHY
RKKYPKQPFYILNPHMQWQLWDILQENSPEDIQPNPPSSGMLGKLIMMNFCDEVDVYEYL
PSKRQTNICHYYQKIFDQACTMGAYHPLLFEKNIIKHLNIGKDEDIYNYGKATLPGLRNV
QC------------
>Ppro_ST6GAL2
----------------------MKSSLKQWRRLTLGLMLVWVLVFLGLLSYFMESRVDDP
HSAAALS-------YTDTRRLTSLQGNPR----------SIMTTHLGLATSSTPSA----
----SSNTQQEQSQEENPSADPQPSPLSQEAYP-----YPDLQS---LAAWSAFGTQDVG
S---RSAAT-SRNRERQ----EYNQDSTQQDEDEEE-VLGGEEEEEEEE---------ER
DEGRRRATKQAT---RRSEDSDQDEYY--VPRSKSVVHGLWKGSLSVGMLSPRLQRAVKD
YLNNNKHGVAYR--GHR-KAQQ-NGQQVLCELKKRQKVRTLDGAEKPFANLGWQ-KIVPA
LPLSQLYG-----SGFQTCAVVTSAGAMLHSGLGKEIDSHDAVLRFNTAPTKGYERDVGN
KTTVRIINSQILANPKHQFNTSSLYKNVTLVAWDPAPYTLNLHKWYSNPDYNLFTPYMEH
RMHFPAQPFYILHPKYIWQLWDVIQGNNLENIQPNPPSSGFIGILLMMSLCEEVHVYEYI
PSLRKTDLCHYHERYFDAACTLGAYHPLLYEKMLIQRMNVGSEEDLKRKGKVTLPGFKNV
HCEP----------
>Ipun_ST6GAL2
----------------------MKASMKQWR-LALGLMLTWALLFLALFSYFMESRVDE-
--AMALS-------HADTRRLTSVQGNTR----------SAMMIPR----QTLPAT----
----SASTTSRAQKERIPNSE-------QEAYV-----YPDEHN---LATWS------VR
S---RSGTV-IHTVEKQ----ESNQTFSQLVEEEDEEEVVEMIDEED-------------
---RSRRNQRKT---NNWFDSNPEEFY--TIKSTSVVQRLWQGRASLDMLSPRLQKAVKD
YMNVNKHGVSYR--GRR-SVGM-SRTRLLCEMKRKAKVKTLNGEERPFSSLGWQ-KIVPA
LPLERLYG-----PGFGSCAVVTSAGAILHSGLGKEIDSHDAVLRFNSAPTKGYERDVGN
KTTIRVINSQILANSRHQFNISSLYKNMTLVAWDPAPYTVNLHKWYKNPDYNLFTPYMKY
RKQFPDQPFYILHPKFIWQLWDVIQANTLENIQPNPPSSGFIGILLMMWLCDEVDVYEYI
PSLRQTDLCHYHERYYDAACTLGAYHPLLYEKVLIQRANSGSQSDLRRKGKVTLPGFRTI
HCNP----------
>Afim_ST6GAL2
----------------------MKSSMRQWRRLVLIGMLAWVLLFLALLSYFLDARVN-E
PLTSMGSLLSQ---HPDTRRLTSIQASHQ-QHANLGSRPELASTLTTTYPRDQPEP---E
PSASSTTPELSLDVSNSPNSAPYAIYGSQETSH---QDYLDPQS---LAAWSSFGTENVG
S---HSDPS-VQSRERTSQT-EYQNHVNTNRHHGGE-DEEDERDNEDE-------ELGN-
-RMRTTAGRRVG-----GDSSDLEEYY--FSKSASVVQRLWRGRVSSGMLSPRLQRAMKD
YVSANKHHVSYT--GHR-KNAQ-SAKELLCQMKAQAQLRTVDGSEQPFSSLGWA-RLVPS
RPLERLHRRQDQSS-FKTCAVVTSAGAILHSGLGKEIDNHDAVLRFNAAPTEGYERDVGN
KTTIRIINSQILANPRHRFNTSSIYKNVTLVAWDPAPYTVNLQKWYASPDYNLFGPYMEH
RKLHPSQPFYILHPSYVWRLWDVIQGNTQENIQPNPPSSGFIGILLMMALCEQVHVYEYI
PSMRQTDLCHYHERYYDAACTLGAYHPLLYEKSLIQRINTGPENDLRRKGRVTLPGLSTV
NCD-----------
>Spar_ST6GAL2
----------------------MKSSMRQWRRLVLVAMLAWVLLFLALLSYFLDARVD-E
PLTSAGSLLSQ---HPDTRRLASIQASHQ-QHAVLGSRPELALPLTTTYPRDKLEL---E
PSASSTTPETSMEVSHNPNSAPYAVYGSQEASR---QDYLDPQS---LAAWSSFGTENVG
S---HSDPV-VQSRERTSQTIEYQNQISATRYQGGEEEEDDER--EEE-------ELGN-
-RMRTTADRRAG-----GDSSDLEEYY--FSKSASVVQRLWRGRVSAGMLSPRLQRAMKD
YMSANKHHVSYK--GQR-RAVQ-SAKELLCQMKAQARLRTVDGSEQPFSSMGWA-RLVPS
VPLEQLQSRQDQSH-FKTCAVVTSAGAILRSGLGKEIDTHDAVLRFNAAPTEGYERDVGN
KTTIRIINSQILANPKHRFNTSSIYKNVTLVAWDPAPYTINLNKWYTSPDYNLFGPYVEH
RKLHPDQPFYILHPSYVWRLWDVIQGNTQENIQPNPPSSGFIGILLMMALCEQVHVYEYI
PSMRQTDLCHYHERYYDAACTLGAYHPLLFEKSLIQRINTGPESDLRRKGRVTLPGFSTV
NCDI----------
>Lcro_ST6GAL2
----------------------MKSSMRQWRRLVLVGMLAWALLFLALLSYFLDARVT-E
PLTSAGSLLSQ---HPDTRRLASIQATHQ-QNANLGSRPEQVSSLTTTNPRD--EP---E
PSASSTTPEFGLEASHSPNSAPYAMYGGNEASQ---QDYLDPQS---LAAWSSFGTENVG
S---HSDPI-VQSRERTSQTTEYQNHVSATRDHGGEEDEEDERENEDE-------ELEN-
-RMRTTAGSRAG-----GDSSDLEEYN--FSKSASVVQRLWRGRASASMLSPRLQRAMKE
YVSGNKHHVSYK--GHR-RAAQ-SAKELLCQMKAQARLRTVDGSEPPFSSLGWA-RLVPA
LPLEELHRRQDQSS-FKTCAVVTSAGAILRSGLGKEIDTHDAVLRFNAAPTEGYERDVGN
KTTIRIINSQILANPRHRFNTSSIYKNVTLVAWDPAPYTVNLQKWYASPDYNLFGPYTEH
RKLHPGQPFYILHPSYVWKLWDVIQGNTQENIQPNPPSSGFIGILLMMALCEKVHVYEYI
PSMRQTDLCHYHERYYDAACTLGAYHPLLYEKSLIQRINAGPESDLRRKGRVTLPGFSTV
NCDI----------
>Cvar_ST6GAL2
----------------------MKSSMKQWRRLLLVAMLAWVLVFLALLSYFLDARVD-E
PLTSAGSLLSQ---HPDTRRLTSIQTSNQ-QLAIFALRPEPALQLTTTYLRNKPGP---E
PSTSSIPSDTSMEVS----QSP---YGSQEANR---QDYLDPQS---LAAWSSFGTENVG
S---NSDPA-VQSRER-----IYQNLDSTTQYHGGE-DEEDGRENE-------------G
KNIRTAVDGRTG-----GESSDLEDYY--FSKSASVVQRLWKGRASAGMLSPRLQRAMKD
YMSANKHHVSYN--GLR-KGSQ-SAKELLCQMKAQGQLKTVDGLEEPFSSLGWA-DFVPS
HPLDHLN-KQLNKSNFKSCAVVTSAGAILRSGLGKEIDTHDAVLRFNTAPTEGYEKDVGN
KTTIRIINSQILANPSHEFNTSSIYKNVTLVAWDPAPYTVNLHKWYASPDYNLFGPYVDH
RKAHPDQPFYILHPSYVWRLWDLIQSNTQENIQPNPPSSGFIGILLMMSLCEQVHVYEYI
PSMRQSDLCHYHERYYDAACTLGAYHPLLYEKILIQRINTGPERDLQKKGRATLPGFSTV
DCDI----------
>Pnye_ST6GAL2
-------MLVGPLSWRACEKEAMKSSMRHWRRLMLVGILAWALFFLALLSYFLDARVD-E
PLTSAGALLSQ---HPDTRRLASIQASHQ-QHAVV----KLTLPLTTTYPRDKPEL---E
PLVKSGTPETSLEMSQNPNNLPYAVYGSQEVSR---QDYLDPQS---LAAWSSFGTENVG
S---HSDPV-VRSRERQSQTADYQNHFSNTQYQGGR-EEGDEKENED---------LGN-
-RRKNRVHSMAG-----DDFSDLEEFY--FSKSISLIKRLWKGRVSAGMLSLRLQRAMKE
YVNSNKHHVSYK--GNR-KPAQ-SAKELLCELKNQARLRTVDGLEQPFSSLGWA-RLVPS
```

LPLEQLYKKENRKS-FKTCAVVTSAGAILRSRLGKEIDAHDAVLRFNAAPTEGYERDVGN
KTTIRIINSQILANPRHRFNTSSIYKNITLVAWDPAPYAVNLHKWYTSPDYNLFGPYMEH
RKLHPDQPFYILHPSYVWQLWDVIQGNTEEDIQPNPPSSGFIGILLMMALCEQVHVYEYI
PSMRQTDLCHYHETYYDAACTLGAYHPLLYEKSLIQRINMGPVSDLERKGRVTLPGFSTV
NCDI-----------
>Onil_ST6GAL2
----------------------MKSSMRHWGRLMLVGMLAWAFVFLALLSYFLDARVD-E
PLTSAGALLSQ---HPDTRRLASIQASHQ-QHAMV----KLSLPLTTTYPRDKPEL---E
PPAKSGTPETSLEMS-------------QEASR---QDYLDPQS---LAAWSSFGTENVG
S---HSDPV-VQSRERQSQTADYQNDFSTIRYQGGK-EEDDEKENEDE-------NLGN-
-RRKNRAQSMAG-----GDFSDLEEFY--FSKSVSLVQRLWKGRVSAGMLSLRLQRAMKE
YVNSNKHHVSYK--GNR-KHAQ-SAKELLCELKNQARLRTVDGLEQPFSSLGWA-RLVPS
LSLEQLYKKENKRS-FKTCAVVTSAGAILRSRLGKEIDAHDAVLRFNAAPTEGYERDVGN
KTTIRIINSQILANPRHRFNTSSIYKNITLVAWDPAPYAVNLHKWYASPDYNLFGPYMEH
RKLHPDQPFYILHPSYVWQLWDVIQGNTEEDIQPNPPSSGFIGILLMMTLCEQVHVYEYI
PSMRQTDLCHYHETYYDAACTLGAYHPLLYEKSLIQRINMGPVSDLERKGRVTLPGFSTV
NCDI-----------
>Ssal_ST6GAL2
----------------------MKSSMRQWRRLVLVGMLAWALLFLALLSYFLDARVDNE
ALTSAGSSLSQ---HPDTRRLSSIQAASHQQHHSIM--GSRPEPAYTTFTTIYPDSEAEP
SPTSTTP-EPGLELSQSPDSGPYGTSQEGEGGSPQQQDYLDPQT---LAAWSSFGTENVG
S---HSDQAVTRSRERTAWTPSDADRGPNRTKRRSSREDEEEEEGVQTN---------NS
HRRRTTVWGRRGKRDGWGKEDDLEEYY--FSKSASVVQRLWRGSVSSEMLSPRLQRAMRD
YLSANKHHVTYK--GGR-RAAQ-SSKDLLCELKDQARLRTLDGSEQPFSSLGWA-HLVPR
LALDQLYRQGGQRG-FRSCAVVTSAGAILHSGLGKEIDSHDAVLRFNAAPTEGYVRDVGN
KTTIRIINSQILANPKNRFNTSSLYKKVTLVAWDPAPYAVNLHKWYASPDYNLFSPYVEH
RRLHPTQPFYVLHPKYVWQLWDVIQGNTQETIQPNPPSSGFIGILLMMALCEKVHVYEYI
PSLRQTDLCHYHERYYDAACTLGAYHPLLYEKSLVQRINTGPERDLKRKGRVTLPGFSTV
NCDL-----------
>Xmac_ST6GAL2
----------------------MKTSMKQWRRLLLVAMLVWVLVFLALLSYFLDARVD-E
SLTSVGSLLYQ---HPDTRRLASIQASNQ-QHAVFGLKPELALQLTTTYLRDKPGP---E
PSTSSITSKTSMEAS----QSPYVTYGSQEAGR---QEYLDPQS---LAAWSSFGTENVG
S---HSDPA-VQSRER-----VYQNQISATQYHGGE-DEDNGREDQD-----------LE
HKVRTAXVGRTG-----GESSDLEDYY--FFRSDSLVQRLWKGHASAGMLSPRLQRAMKN
YMSSNKHHVFYN--GHR-KGTQ-SAKELLCQMKAQAQLKTVDGLEEPFSSLGWA-DFVPS
HPLEQLNKQQQDRSGFKSCAVVTSAGAILRSRLGKEIDAHDAVLRFNAAPTEGYEHDVGS
KTTIRIINSQILANPSYEFNTSSIYKNITLVAWDPAPYNVNLHKWYDSPDYNLFGPYIDH
RKAHPDQPFYILHPSYVWRLWDVIQSNTQENIQPNPPSSGFIGILLMMALCEQVHVYEYI
PSMRQSDLCHYHEHYYDAACTLGAYHPLLFEKILIQRINKGLESDLRRKGRATLPGFSTI
DCGI-----------
>Pmex_ST6GAL2
----------------------MKSSMKQWRRLLLVAMLVWILVFLALLSYFLDARVD-E
SLTSVGSLLSQ---HPDTRRLASIQASNQ-QHAVFGLRPEQALQLTTTYLRDKTGP---G
PSTSSITSETSMEAS----QSPYVTYGSQEAGH---QEYLDPQS---LAAWSSFGTENVG
S---HSDPA-VQSRER-----MYQNQIGATHYHGGE-DEEDGREDQD-----------LE
PKIRTAGDGRTG-----GESSDLEDYY--FSRSASVVRRLWKGHASAGMLSPRLQRAMKD
YMSANKHHVFYN--GHR-KGSQ-SAKALLCQMKAQGQLKTVDGLEEPFSSLGWA-DFVPS
RPLELLNKRQQGRSGFKSCAVVTSAGAILRSRLGKEIDAHDAVLRFNAAPTEGYEHDVGN
KTTIRIINSQILANPKHEFNTSSIYKNITLVAWDPAPYTVDLQKWYASPDYNLFGPYIDH
RKAHPDQPFYILHPSYVWRLWDVIQSNTQENIQPNPPSSGFIGILLMMALCEQVHVYEYI
PSMRQSDLCHYHERYYDAACTLGAYHPLLYEKILIQRINTGPQSDLRRKGRATLPGFSTI
NCDI-----------
>Alim_ST6GAL2
----------------------MKSSMRQWWKLALVAMLAWVLVFFVLLSYFLDTRAN-E
PLTSSGSLLSQ---HPDTRRLTSIQASNL-QHSVLG-------SRPTMYIRETPGFG---
SLSSSSTPETSVEVSQSPNEAPYATYGSQEVNH---RDYLDPQS---LAAWSSFGTENVE
NQGFHSDPA-VQSRERTSLITEYPNHDDTTPYHDGD-EEDDGNENEE-----------LG
IRRRAPRAKRAG-----GDSANLDEYH--FSKFDSVVHRLWRGRVSANMLSPRLQQAMKE
YMSANKHSVSYK--GHR-KTSQ-SAKDMLCQMKAQAQLRTLDGSEEPFSSLGWV-DFVPP
LPLERLSRRQEKSS-LKTCAVVTSAGAMLRSRLGKEIDSHDAVLRFNSAPTDGYERDVGN
TTTIRIINSQILANPTYQFNTSSIYKNVTLVAWDPTPYSVNLHKWYTSPDYDLFGPYVDH
RKVHPDQPFYILHPSYVWKLWDLIQSNTQENIQPNPPSSGFIGILLMMALCEQVHVYEYI
PSMRQTDLCHYHERYYDAACTLGAYHPLLYEKSLIQRINTGPENDLRRKGRVTLPGFSTV
DCDI-----------
>Eluc_ST6GAL2
----------------------MKSSMRQWRRLVLMGMLAWALLFLALLSYFLDARVDSE
PGMSAGSSLAQ---HPDTRRLSSIQGAQHLHR------GSLSQPAYTAFNTSYP--ALEP
SPASTSPPSPGLELSQSPDSGPY-SSREGDGGSHQRADYLDPQA---LAAWSSVGTENVV
S---RSDRMGARSRSRTG-------RGPDRIMRQHSRNEGGEEE-EQTN---------HS
HKRRTTVGGRGREREARGKEDSSLEYY--SSKSSSVLQRLWQGSVSSGMLSPRLKHAMTN
YLNANKHRVAYK--GSR-RPAQ-TREQLLCELKDKARLRMLDGSEQPFMSLGWS-HLVPR
VNLEGTER--GQSR-FRTCAVVTSAGSILHSGLGKEIDSHDAVLRFNAAPTEGYERDVGS
KTTIRIINSQILADPKHRFNTSSLYKNMTLVAWDPAPYAVNLDKWYASPDYNLFSPYEEH
RKHHPAQPFYILHPKYVWQLWDIIQGNTKETIQPNPPSSGFIGILLMMALCEKVHVYEYI
PSLRKTDLCHYYEHYYDAACTLGAYHPLLYEKSLVQRMNVGQELDLKQKGCVTLPGFNSV
NCNL-----------
>Locu_ST6GAL2
----------------------MKSKVKQWKQLVLMGILAWIVVFLALFTYFMDFRMNVP
RVTGSLS-------HTETRKLTSIQVNNQ-------------NVMLSQSDIATPDSNSHK
KP--YNGPVLTEKTGTPNLEVWSYFENEIQNT-------------ISRDDTSAQMSTGEI
SVAAGQQHRKTQVNKHAVENFYFYEYNSAKTEHVPTVMKRSRLRRHRH------------
------RTSKT--------VEDLEEYL---FKSKSVIYKLWKGNVSSKMLSPRLQKAMKE
YLDANKHNVSYE--GKR-ASKL-SRQEVLCELKHQIKVQSLDGTERPFSKLGWK-KFVPK
ISLD--ELHPD---GFRKCAVVTSAGAMLNSSLGKEIDSHEAVVRFNAAPTIGYEKDVGN
KTTIRIVNSQIIANSIHRFNISTLYRDVILIAWDPAPYTANLQKWYENPDYDLFTPYVEY
RKKNPYQPFYILHPKFIWKMWDVIQENTHEKIQPNPPSSGFIGILIMMSLCDEVDVYEYV
PSIRQTDLCHYHEQYYDAACTLGAYHPLLYEKMLIQKVNMGTEEDLKKKGKVTLPGFRAI
NCKH-----------
>Etel_ST6GAL2

---------------------MKPNLKQWKQLMLFGIFAWGLLFLVIFIYFTDSEPADP
-APSSFS-------FLEARRLLPVQGKRR------------AIMGAIHEAAFAHGLDLG
---------DPLNVGPSHLQRWAPRRDALENEE----EYFTSQI--GRKSQSAFDQGLDD
DFFAADQPGVAGHPREVLPFT---------GALPARHAQRLRMKRRRW-----------
------KQKRS------PEFEDWDRLY--STMSRAFLYRLWKGNVSSKMLTPRLQKAMRD
YLNTNKHGVRFR--GKR-EARL-SGHQLLCELRRRVRVRTLDGKEAPFSALGWE-KHVPA
VPLS--QLHPR---GLRTCAVVMSAGAILNSSLGEEIDSHDAVLRFNSAPTRGYEKDVGN
KTTLRIINSQILTNPSHHFIDSSLYKDVILVAWDPAPYSANLNLWYKKPDYNLFTPYIQH
RQRNPNQPFYILHPKFIWQLWDIIQENTKEKIQPNPPSSGFIGILIMMSMCREVHVYEYI
PSVRQTELCHYHELYYDAACTLGAYHPLLYEKLLVQRMNTGVQEDLHRKGKVTLPGLWAV
SCPAPN-SAPHS---
>Psin_ST6GAL2
---------------------MKPNLKQWKQLMLFGLFAWGLLFLAIFIYFTDSNSAEP
-VPSSFS-------YIETKRLLPIQGKHR------------VIMGAIHDPSFSETIDRD
EALLNEDLLGSFKSGTGSVKKWIDLEDGFENEE----EFFPSQM--GTKSKGAFYQGDDN
YLFAGGPSQQHSNFQELVKFISVNEADQKRNVLQAKWTHEKRTRKRNP-----------
------AHRRS---PMFDEPDDWDGLY--STMSKSFLYRLWKGDVSSKMLNPRLQKAMKD
YLNTNKHGVRFK--GKR-NSRL-TGEQLICELKGRVNVKTIDGKEAPFSALGWE-KHVPQ
IPLG--KLYPH---GFGSCAVVMSAGAILNSSLGDEIDSHDAVLRFNSAPTRGYEKDVGN
KTTMRIINSQILTNPNHHFIDSSLYKDVILVAWDPAPYSANLNVWYKKPDYNLFTPYVQH
RRRNPNQPFYILHPKFIWQLWDIIQENTQEKIQPNPPSSGFIGILIMMSMCNEVHVYEYI
PSVRQTDLCHYHELYYDAACTLGAYHPLLYEKLLVQRMNKGLQDDLYRKGKVILPGFRSV
KCPGRENVPHL----
>Cpicbel_ST6GAL2
---------------------MKPNLKQWKQLMLFGLFAWGLLFLVIFIYFTDSNTAEP
-VPSSFS-------YIETKRLLPIQGKQR------------VIMGAIHDPSSSETIDRN
EVLLNEDLLDSFKSGTGSIKKWIDLEDGFENEE----EFFPSQV--GRKSKYAFYQGDDN
YLFAAGQPQQHSNIQELVKFISPN--DQKENVLQEKWTHEKRTKKRNP-----------
------TRRRS---QLFDESDDWDGLY--STMSKSFLYRLWKGDISSKMLTPRLQKAMKD
YLNTNKHGVRFK--GKRRNSRL-TGDQLICELKDRVHVKTIDGKEDPFSALGWE-KHVPQ
IPLG--KLYPH---GFGSCAVVMSAGAILNSSLGDEIDSHDAVLRFNSAPTRGYEKDVGN
KTTMRIINSQILTNPNHHFIDSSLYKDVILVAWDPAPYSANLNVWYKKPDYNLFTPYVQH
RRRNPNQPFYILHPKFIWQLWDIIQENTKEKIQPNPPSSGFIGILIMMSMCNEVHVYEYI
PSVRQTDLCHYHELYYDAACTLGAYHPLLYEKLLVQRMNKGLQDDLYRKGKVILPGFRSV
KCPGQNHFPHL----
>Cmyd_ST6GAL2
---------------------MKPNLKQWKQLMLFGLFAWGLLFLVIFIYFTNX-----
---SSFS-------YTETKRLLPIQGKQR------------VIMGAIHDPSSSETIDRN
EVLLNEELLDSFNLGTGSIKKWIDLEDGFENEE----EYFPSQI--GRKSKNAFYQGDDN
YLFAAGQPRQHSNIQEIVKFISPNEDDQKENVLQEKWTHEKRTKKRNP-----------
------THRRS---QLFDESDDWDGLY--STMSKSVLYRLWKGDVSSKMLNPRLQKAMKD
YLNTNKHGVRFK--GKR-NSRL-TGDQLICELKDRVHVKTIDGKEAPFSALGWE-KHVPQ
IPLG--KLYPH---GFGSCAVVMSAGAILNSSLGDEIDSHDAVLRFNSAPTRGYEKDVGN
KTTMRIINSQILTNPNHHFIDSSLYKDVVLVAWDPAPYSANLNVWFKKPDYNLFTPYVQH
RRRNPNQPFYILHPKFIWQLWDIIQENTKEKIQPNPPSSGFIGILIMMSMCNEVHVYEYI
PSVRQTDLCHYHELYYDAACTLGAYHPLLYEKLLVQRMNKGLQDDLYRKGKVILPGFRSV
KCLGRNHFPHL----
>Mzeb_ST6GAL2
-------MLVGPQSWRACEKEAMKSSMRHWRRLMLVGILAWALFFLALLSYFLDARVD-E
PLTSAGALLSQ---HPDTRRLTSIQASHQ-QHAVV----KLTLPLTTTYPRDKPEL---E
PLVKSGTPETSLEMSQNPNNLPYAVYGSQEVSR---QDYLDPQS---LAAWSSFGTENVG
S---HSDPV-VQSRERQSQTADYQNHFSNTQYQGGR-EEGDEKENED---------LGN-
-RRKNRVHSMAG-----DDFSDLEEFY--FSKSISLIKRLWKGRVSAGMLSLRLQRAMKE
YVNSNKHHVSYK--GNR-KPAQ-SAKELLCELKNQARLRTVDGLEQPFSSLGWA-RLVPS
LPLEQLYKKENRRS-FKTCAVVTSAGAILRSRLGKEIDAHDAVLRFNAAPTEGYERDVGN
KTTIRIINSQILANPRHRFNTSSIYKNITLVAWDPAPYAVNLHKWYTSPDYNLFGPYMEH
RKLHPDQPFYILHPSYVWQLWDVIQGNTEEDIQPNPPSSGFIGILLMMALCEQVHVYEYI
PSMRQTDLCHYHEAYYDAACTLGAYHPLLYEKSLIQRINMGPVSDLERKGRVTLPGFSTV
NCDI----------
>Hbur_ST6GAL2
-------MLVGPQSWRACEKEAMKSSMRHWRRLMLVGILAWALFFLALLSYFLDARVD-E
PLTSAGALLSQ---HPDTRRLASIQASHQ-QHAVV----KLTLPLTTTYPRDKPEL---E
PLVKSGTPETNLEMSQNPNNLPYAVYGSQEVSR---QDYLDPQS---LAAWSSFGTENVG
S---HSDPV-VQSRERQSQTADYQNHFSNTQYQGGR-EEGDEKENED---------LGN-
-RRKNRVHSMAG-----DDFSDLEEFY--FSKSISLIKRLWKGRVSAGMLSLRLQRAMKE
YVNSNKHHVSYK--GNR-KPAQ-SAKELLCELKNQARLRTVDGLEQPFSSLGWA-RLVPS
LPLEQLYKKENRRS-FKTCAVVTSAGAILRSRLGKEIDAHDAVLRFNAAPTEGYERDVGN
KTTIRIINSQILANPRHRFNTSSIYKNITLVAWDPAPYAVNLHKWYTSPDYNLFGPYMEH
RKLHPDQPFYILHPSYVWQLWDVIQGNTEEDIQPNPPSSGFIGILLMMALCEQVHVYEYI
PSMRQTDLCHYHETYYDAACTLGAYHPLLYEKSLIQRINMGPVSDLERKGRVTLPGFSTV
NCDI----------
>Nbri_ST6GAL2
-------MLVGPQSWRACEKEVMKSSMRHWRRLMLVGILAWALFFLALLSYFLDARVD-E
PLTSAGALLSQ---HPDTRRLASIQASHQ-QHAMV----KLTLPLTTTYPRDKPEL---E
PLAKSGTPETSLEMSQNPNNLPYAVYGSQEVSR---QDYLDPQS---LAAWSSFGTENVG
S---HSDPV-VQSRERQSQTADYQNHFSNTQYQGGK-EEDDEKENEDE-------DLGN-
-RRKNRVQSMAG-----DDFSDLEEFY--FSKSISLIKRLWKGRVSAGMLSLRLQRAMKE
YVNSNKHHVSYK--GNR-KPTQ-SAKELLCELKNQARLRTVDGLEQPFSSLGWA-RLVPS
LPLEQLYKKENKRS-FKTCAVVTSAGAILRSRLGKEIDAHDAVLRFNAAPTEDYERDVGN
KTTIRIINSQILANPRHRFNTSSIYKNITLVAWDPAPYAVNLHKWYTSPDYNLFGPYMEH
RKLHPDQPFYILHPSYVWQLWDVIQGNTEEDIQPNPPSSGFIGILLMMALCEQVHVYEYI
PSMRQTDLCHYHETYYDAACTLGAYHPLLYEKSLIQRINMGPVSDLERKGRVTLPGFSTV
NCDI----------
>Pfor_ST6GAL2
---------------------MKSSMKQWRRLLLVAMLVWILVFLALLSYFLDARVD-E
SLTSVGSLLSQ---HPDTRRLASIQASNQ-QHAVFGLRPEQALQLTTTYLRDKPGPPGPG
PSTSSITSETSMEAS----QSPYVTYGSQEAGH---QEYLDPQS---LAAWSSFGTENVG
S---HSDTA-VQSRER-----MYQNQIGATHYHGGE-DEEDGREDQD----------LE
PKIRTAGDGRTG-----GESSDLEDYY--FSRSASVVRRLWKGHASAGMLSPRLQRAMKD
YMSANKHHVFYN--GHR-KGSQ-SAKALLCQMKAEGQLKTVDGLEEPFSSLGWA-DFVPS

```
RPLELLNKRQQGRSGFKSCAVVTSAGAILHSRLGKEIDAHDAVLRFNAAPTEGYEHDVGN
KTTIRIINSQILANPKHEFNTSSIYKNITLVAWDPAPYTVDLQKWYASPDYNLFGPYIDH
RKAHPDQPFYILHPSYVWRLWDVIQSNTQENIQPNPPSSGFIGILLMMALCEQVHVYEYI
PSMRQSDLCHYHERYYDAACTLGAYHPLLYEKILIQRINTGPQSDLRRKGRATLPGFSTI
NCDI-----------
>Scamaus_ST6GAL2
MWKVLFSFTDTSRHRGAGKGIAMKPNLKQWKQLMLFGIFAWGLLFLVIFIYFTDSNTAEP
-VPSSFS-------YIETKRLLPIQGKQR------------VIMGAIHDPSFSETIDEN
EVLLNEDLLGTFKSGTGSIKKWTDLEDAFRSED----EFFPSQI--GRKSKSAFYQVSND
YLFAAGQPQSHNNLQEIAKFISADEDNPKENVLQNNWSHQRRMRRRNS-----------
------KHRRS---QMLDESDDWDGLY--STMSKSFLYKLWKGDVSSKMLNPRLQKAMKD
YLSTNKHGVRFK--GKR-NSKL-TGDQLFCELKERVHVKTIDGKEAPFSTLGWE-KHVPQ
IPLG--KLYTH---GFGSCAVVMSAGAILNSSLGSEIDSHDAVLRFNSAPTRGYEKDVGN
KTTMRIINSQILTNPSHHFVDSSLYKDVILVAWDPAPYSANLNVWYKKPDYNLFTPYVQH
RRKNPNQPFYILHPKFIWQLWDIIQENTKEKIQPNPPSSGFIGILIMMSMCSEVHVYEYI
PSVRQTDLCHYHELYYDAACTLGAYHPLLYEKLLVQRMNKGLQDDLYRKGKVILPGFKSV
KCPERNNFPHL----
>Acygdom_ST6GAL2
---------------------MKPNLKQWKQLMLFGIFAWGLLFLVIFIYFTDSNTAEP
-VPSSFS-------YIETKRLLPLQGKQR------------VIMGAIHDPSFYETIDGN
EVLLNEDLLGTFKSGTGSIKKWTDLEDAFRSED----EYFPSQL--GRKSKSAFYQVNDD
YLFAAGQPQSHSNLQEIAKFISADEDNPKENILQNNWGHQRRMRRRSA-----------
------KHRRS---KMLDESDDWDGLY--STMSKSFLYKLWKGDVSSKMLNPRLQKAMKD
YLSTNKHGVRFK--GKR-NSKL-TGDQLFCELKERVNVRTIDGKEAPFSTLGWE-KHVPQ
IPLG--KLYTH---GFGSCAVVMSAGAILNSSLGDEIDSHDAVLRFNSAPTRGYEKDVGN
KTTMRIINSQILTNPNHHFVESSLYKDVILVAWDPAPYSANLNVWYKKPDYNLFTPYVQH
RRKNPNQPFYILHPKFIWQLWDIIQENTKEKIQPNPPSSGFIGILIMMSMCDEVHVYEYI
PSVRQTDLCHYHELYYDAACTLGAYHPLLYEKLLVQRMNKGLQDDLYRKGKVILPGFKSV
KCPKRNNFPHL----
>Achrcan_ST6GAL2
---------------------MKPNLKQWKQLMLFGIFAWGLLFLVIFIYFTDSNTAEP
-VPSSFS-------YIETKRLLPLQGKQR------------VIMGAIHDPSFSETIDGN
EVLLNEDLLGTFKSGTGSIKKWTDLEDAFRNED----EFFPSQV--GRKSKSAFYQVNDD
YLFAAGQPRSHNHLQEIAKFISADEDNPKVNILQNDWSHQRRMRRRST-----------
------KHRRS---QMLDESDDWDGLY--STMSKSFLYKLWKGDVSSKMLNPRLQKAMKD
YLSTNKHGVRFK--GKR-NSKL-TGDQLFCELKERVNVKTIDGKEAPFSTLGWE-KHVPQ
IPLG--KLYTH---GFGSCAVVMSAGAILNSSLGDEIDSHDAVLRFNSAPTRGYEKDVGN
KTTMRIINSQILTNPNHHFVDSSLYKDVILVAWDPAPYSANLNVWYKKPDYNLFTPYVQH
RRKNPNQPFYILHPKFIWQLWDIIQENTKEKIQPNPPSSGFIGILIMMSMCNEVHVYEYI
PSVRQTDLCHYHELYYDAACTLGAYHPLLYEKLLVQRMNKGLQDDLYRKGKVILPGFKAV
KCPERNNFSHL----
>Apla_ST6GAL2
---------------------MKPNLKQWKQLMLFGIFAWGLLFLVIFIYFTDSNTAEP
-VPSSFS-------YIETKRLLPLQGKQR------------VIMGAIHDPSFSETIDGN
EVLLNEDLLGTFKSGTGSIKKWTDLEDAFRSED----EYFPSQL--GRKSKSAFYQVNDD
YLFAAGQPRSHSNLQEIAKFISADEDNLKENILQNNWGNQRRMRRRSA-----------
------KHRRS---QMLDESDDWDGLY--STMSKSFLYKLWKGDVSSKMLNPRLQKAMKD
YLSTNKHGVRFK--GKR-NSKL-TGDQLFCELKERVNVKTIDGKEAPFSTLGWE-KHVPQ
IPLG--KLYTH---GFGSCAVVMSAGAILNSSLGDEIDSHDAVLRFNSAPTRGYEKDVGN
KTTMRIINSQILTNPNHHFVESSLYKDVILVAWDPAPYSANLNVWYKKPDYNLFTPYVQH
RRKNPNQPFYILHPKFIWQLWDIIQENTKEKIQPNPPSSGFIGILIMMSMCDEVHVYEYI
PSVRQTDLCHYHELYYDAACTLGAYHPLLYEKLLVQRMNKGLQDDLYRKGKVILPGFKSV
KCPKRNNFPHL----
>Mund_ST6GAL2
---------------------MKPNLKQWKQLMLFGIFAWGLLFLVIFIYFTDSNTAEP
-VPSSFA-------YIETKRLLPLQGKQR------------VIMGAIHDPSFSETIDGN
EVLLNEDLLGTFKSGTGSIKKLTDLEDAFRNED----EFFRSQM--GRKSKSAFYQVNDD
YLFAAGQPRSHNHLQEIAKFISADEDNPKVNLLQSNWSHQRRMRRRST-----------
------KHRRS---QMLDESDDWDGLY--STMSKSFLYKLWKGDVSSKMLNPRLQKAMKD
YLSTNKHGVRFK--GKR-NSKL-TGDQLFCELKERVNVKTIDGKEAPFSTLGWE-KHVPQ
IPLG--KLYTH---GFGSCAVVMSAGAILNSSLGDEIDSHDAVLRFNSAPTRGYEKDVGN
KTTMRIINSQILTNPNHHFVDSSLYKDVILVAWDPAPYSANLNVWYKKPDYNLFTPYVQH
RRKNPNQPFYILHPKFIWQLWDIIQENTKEKIQPNPPSSGFIGILIMMSMCNEVHVYEYI
PSVRQTDLCHYHELYYDAACTLGAYHPLLYEKLLVQRMNKGLQDDLYRKGKVILPGFKAV
KCPKQNNSPHL----
>Scan_ST6GAL2
---------------------MKPNLKQWKQLMLFGIFAWGLLFLVIFIYFTDSNTAEP
-VPSSFS-------YIETKRLLPLQGKQR------------VIMGAIHDPSFSETIDGN
EVLLNEDLLGTFKSGTGNSKKWTVLEEAFRNED----EFFPPQL--GRKSKSAFYQVNDD
YLFAAGQPQSHNHLQEIEKFISVDVNNPKGNILQNSWSHQRRMRRRST-----------
------KHRRG---QMLDESDDWDGLY--STMSKSFLYKLWKGDVSSKMLNPRLQKAMKD
YLSTNKHGVRFK--GKR-NSKL-TGDQLFCELKERVNVKTIDGKEAPFSTLGWE-KHVPQ
IPLV--KLYTH---GFGSCAVVMSAGAILNSSLGDEIDSHDAVLRFNSAPTRGYEKDVGN
KTTMRIINSQILTNPNHHFVDSSLYKDVILVAWDPAPYSANLNVWYKKPDYNLFTPYVQH
RRKNPTQPFYILHPKFIWQLWDIIQENTKEKIQPNPPSSGFIGILIMMSMCNEVHVYEYI
PSVRQTDLCHYHELYYDAACTLGAYHPLLYEKLLVQRMNKGLQDDLYRKGKVILPGFKAV
KCPKRSNFPH-----
>Fper_ST6GAL2
---------------------MKPNLKQWKQLMLFGIFAWGLLFLVIFIYFTDSNTAEP
-VPSSFS-------YIETKRLLPLQGKQR------------VIMGAIHDPSFSENIDGN
EVLLNEDLLGTFKSGTGSIKKWTDLEDAFRNED----EFFPSQI--GRKSKSAFYQVNDN
YLFAAGQPRSHNHLQEIAKLISADEDNPKVNILQNNWSHQRRMRRRST-----------
------KHRRS---QMLDESDDWDGLY--STMSKSFLYKLWKGDVSSKMLNPRLQKAMKD
YLSTNKHGVRFK--GKR-NSKL-TGDQLFCELKERVNVKTIDGKEAPFSTLGWE-KHVPQ
IPLG--KLYTH---GFGSCAVVMSAGAILNSSLGDEIDSHDAVLRFNSAPTRGYEKDVGN
KTTMRIINSQILTNPNHHFVDSSLYKDVILVAWDPAPYSANLNVWYKKPDYNLFTPYVQH
RRKHPNQPFYILHPKFIWQLWDIIQENTKEKIQPNPPSSGFIGILIMMSMCNEVHVYEYI
PSVRQTDLCHYHELYYDAACTLGAYHPLLYEKLLVQRMNKGLQDDLYRKGKVILPGFKAV
KCPERNNFPHL----
>Cmil_ST6Gal2
```

```
----------------------MKANTKQWKHLVLIGILAWALVVLLLFIYFTDLKTDER
-PARSLR-------YTETRSLFPIQGKQR-------------VIVGNLQNSKLSPVHGEN
NLYFEDHDDDVSLLGLDSLNGQNLKWTREEDNG----EFEVTKR--VKDRKIAAHQQPPN
------------WVMEQVVNITKDANTYDEIVVQDAKPHKRAMKRQEQ------------
------SSYSVD--PFLDNLDSLDLEDLQFSKSKAVLIKLWKGNFTYNELNPRLQRAMRE
YIYENKHGVQFK--GRRGTKKL-SGDELLCELKKRVRLRTLDGNETPFSLLGWK-KYVPK
IPLSKIKLKLR---VFRKCAVVASAGAILNSSLGDEIDSHDAVMRFNAAPTKLYEQDVGS
KTTIRILNSQILANSKHNFINNALYKNIILVVWDPAPYSINLIKWYKKPDYNLFTPYLRY
RRRNPAQPFYILHPRFLWQLWDIIQENTQEKIQPNPPSSGFIGIVIMMALCDSINIYEYI
PSIRQTDLCHYHERYYDSACTLGAYHPLLYEKLLVQRMNKGTEADLYSKGRVSLPGFHSI
KCAGEK---------
>Hsap_ST6Gal2
----------------------MKPHLKQWRQRMLFGIFAWGLLFLLIFIYFTDSNPAEP
-VPSSLS-------FLETRRLLPVQGKQR-------------AIMGAAHEPSPPGGLDAR
QALPRAHPAGSFHAGPGDLQKWAQSQDGFEHK-----EFFSSQV--GRKSQSAFYPEDDD
YFFAAGQPGWHSHTQGTLGFPSPGEPGPREGAFPAAQVQRRRVKKRHR------------
------RQRRS---HVLEEGDDGDRLY--SSMSRAFLYRLWKGNVSSKMLNPRLQKAMKD
YLTANKHGVRFR--GKR-EAGL-SRAQLLCQLRSRARVRTLDGTEAPFSALGWR-RLVPA
VPLS--QLHPR---GLRSCAVVMSAGAILNSSLGEEIDSHDAVLRFNSAPTRGYEKDVGN
KTTIRIINSQILTNPSHHFIDSSLYKDVILVAWDPAPYSANLNLWYKKPDYNLFTPYIQH
RQRNPNQPFYILHPKFIWQLWDIIQENTKEKIQPNPPSSGFIGILIMMSMCREVHVYEYI
PSVRQTELCHYHELYYDAACTLGAYHPLLYEKLLVQRLNMGTQGDLHRKGKVVLPGFQAV
HCPAPSPVIPHS---
>Ptro_ST6Gal2
----------------------MKPHLKQWRQRMLFGLFAGGLLFLLIFIYFTDSNPAEP
-VPSSLS-------FLETRRLLPVQGKQR-------------AIMGAAHEPSPPGGLDAR
QALPRAHPAGSFHAGPGDLQKWAQSQDGFEHK-----EFFSSQV--GRKSQSAFYPEDDD
YFFAAGQPGWHSHSQGTLGFPSPGEPGPREGAFPAAQVQRRRVKKRHR------------
------RQRRS---HVLEEGDDGDRLY--SSMSRAFLYRLWKGNVSSKMLNPRLQKAMKD
YLTANKHGVRFR--GKR-EAGL-SRAQLLCQLRSRARVRTLDGTEAPFSALGWR-RLVPA
VPLS--QLHPR---GLRSCAVVMSAGAILNSSLGEEIDSHDAVLRFNSAPTRGYEKDVGN
KTTVRIINSQILTNPSHHFVDSSLYKDVILVAWDPAPYSANLNLWYKKPDYNLFTPYIQH
RQRNPNQPFYILHPKFIWQLWDIIQENTKEKIQPNPPSSGFIGILIMMSMCREVHVYEYI
PSVRQTELCHYHELYYDAACTLGAYHPLLYEKLLVQRLNTGTQGDLHRKGKVVLPGFQAV
HCPAPSPVIPHS---
>Rnor_ST6Gal2
----------------------MKPHLKQWRQRMLFAIFVWGLLFLAIFIYFTNSNPAAP
-MPSSFS-------FLESRGLLPVQGKQR-------------VIMGALQEPSLPRSLEPS
KVLMDGHSASPFNSWPGDPQKGDQAQDGFDNGD----EFFTSQV--GRKSQSAFYPEEDN
YFFVAGQPGLYHHRQGALGLPSPGESSWQSGPGQPKQEKLRH----PR------------
------RGSLP------EEAYDSDMLS--TSMSRAFLYRLWKGTVSSKMLNPRLQKAMRY
YMSFNKHGVRFSRRGRR-EARR-TGPELLCEMRKRVRVRTLDGKEAPFSGLGWR-PLVPG
VPLS--QLHPR---GLRSCAVVMSAGAILNSSLGEEIDSHDAVLRFNSAPTRGYEKDVGN
KTTVRIINSQILANPSHHFIDSSLYKDVILVAWDPAPYSANLNLWYKKPDYNLFTPYIQH
RLKYPTQPFYILHPKFIWQLWDIIQENTREKIQPNPPSSGFIGILVMMSMCQEVHVYEYI
PSVRQTELCHYHELYYDAACTLGAYHPLLYEKLLVQRLNTGTQADLHHKGKVVLPGFQTL
RCPVTRPNNTNT---
>Mmus_ST6Gal2
----------------------MKPHLKQWRQRMLFGIFVWGLLFLAIFIYFTNSNPAAP
-MPSSFS-------FLESRGLLPLQGKQR-------------VIMGALQEPSLPRSLDAS
KVLLDSHPENPFHPWPGDPQKWDQAPNGFDNGD----EFFTSQV--GRKSQSAFYPEEDS
YFFVADQPELYHHRQGALELPSPGETSWRSGPVQPKQ-KLLH----PR------------
------RGSLP------EEAYDSDMLS--ASMSRAFLYRLWKGAVSSKMLNPRLQKAMRY
YMSFNKHGVRFRRRGRR-EATR-TGPELLCEMRRRVRVRTLDGREAPFSGLGWR-PLVPG
VPLS--QLHPR---GLSSCAVVMSAGAILNSSLGEEIDSHDAVLRFNSAPTRGYEKDVGN
KTTVRIINSQILANPSHHFIDSALYKDVILVAWDPAPYSANLNLWYKKPDYNLFTPYIQH
RRKYPTQPFYILHPKFIWQLWDIIQENTREKIQPNPPSSGFIGILIMMSMCKEVHVYEYI
PSVRQTELCHYHELYYDAACTLGAYHPLLYEKLLVQRLNTGTQADLHHKGKVVLPGFQTL
RCPVTSPNNTHS---
>Drer_ST6Gal2
----------------------MKSSLKQWRRLALGLILVWALLFLALLSYFMESRVDDP
HAAAALS-------YTDTRRLTSLQGNPR----------TIMATHLGLATSSAPST----
----SSNTQQEQSQEENPSADPQPSPLSQEAYP-----YPDPQS---LAAWSAFGTQDVG
S---RSTGV-SRNRERQ----EYNQDSPQEDEDEEEEVIGGEEEDEE-----------GG
DEGRGRTTKRVA---RHG-SSDPHEYY--VPRYKSIVHGLWKGSLSMGMLSPRLQRAMKD
YLNNNKHGVAYR--GHR-KAKQ-SRQQVLCELKKREKIRTLDGAEMPFSKLGWQ-KIVPA
LPLSQIHR-----PGLKTCAVVTSAGAMLHSGLGKEIDSHDAVLRFNTAPTVGYERDVGN
KTTIRIINSQILANPMHRFNRSSLYKNVTLVAWDPAPYTLNLHQWYSNPDYNLFTPYMEY
RMRFPSQPFYILHPKYIWQLWDVIQANNLENIQPNPPSSGFIGILLMMSLCEEVHVYEYI
PSLRQTDLCHYHERYYDAACTLGAYHPLLYEKMLIQRMNIGSEDELKRKGKVTLPGFNKV
HCEP-----------
>Ggal_ST6Gal2
----------------------MKPNLKQWKQLMLFGIFAWGLLFLVIFIYFTDSNSAEP
-VPSSFS-------YIETKRLLPLQGKQR-------------VIMGAIHDPSFSEAIDGN
EVLLNEDLLDTFKSETGSIKKWTDLEDAFRSED----EFFPSQI--GRKSKSAFYQVNDD
YLFAAGQPMSHNSFQEIAKFISADEDNPKESILQNNWSRQRRMRRRST------------
------KHRRS---QMLDESDDWDGLY--STMSKSFLYKLWKGDVSSKMLNPRLQKAMKD
YLSTNKHGVRFK--GKR-NSKL-TGDQLFCELKERVDVKTIDGKEAPFSTLGWE-KHVPQ
IPLG--KLYTH---GFGSCAVVMSAGAILNSSLGDEIDSHDAVLRFNSAPTRGYEKDVGN
KTTMRIINSQILTNPNHHFVDSSLYKDVILVAWDPAPYSANLN-WYKKPDYNLFTPYVQH
RKKNPNQPFYILHPKFIWQLWDIIQENTKEKIQPNPPSSGFIGILIMMSMCNEVHVYEYI
PSVRQTDLCHYHELYYDAACTLGAYHPLLYEKLLVQRMNKGLQDDLYRKGKVILPGFKSV
KCPERNNFPPL----
>Trub_ST6Gal2
----------------------MKSWVRQGRRLVLVGMLAWVLLFLALLSYFLDARVN-E
PLTSTGSVLYQ---HPDTRRLASIQASQL-HNTNLGSRPELASTLTATYTRDEPRP----
----SATPEFSLEASQSPDSAPLAIYGGPEGIH---QDYLDPQS---LAAWSSFGTENIG
S---QSDPV-IQSRER-----TSQNHGSITRYRGGEEEEEEEEEEER----QENEDEDVA
NGLRNTAARKPG-----GDSSELEKYY--FSKSISVVQRLWRGHVSADMLSPRLQRAMKD
YVSANKHQVSYR--GRR-RPSQ-SAKELLCEMKAQARLQMVDGTQQPFSSLGWA-SLVPS
```

```
LPLEQLHKRPDQGS-FKSCAVVTSAGAILRSGLGREIDAHDAVLRFNAAPTEGYERDVGN
KTTIRIINSQILANPNHRFNTSSLYKDVVLVAWDPAPYTLDLHKWYASPDYNLFGPYMEH
RRAHPDQPFYILHPRYVWRLWDVIQGNTQENIQPNPPSSGFIGILLMMTLCEQVHVYEYI
PSMRQSDLCHYHERYYDAACTLGAYHPLLYEKSLIQRINTGPGSDLRRKGRVTLPGFSTV
DCDI-----------
>Btau_ST6Gal2
----------------------MKPHLKQWRQGMLCGVFAWGLFFVVIFLYFTDSSPAKP
-APSSFS-------FLETRRLLPAQGRQR-------------AIMGASEG--LPEGADLR
RGSPRGLPS-------GPLRTWAG--DGFEREQ----EFLSVQT--GRTSLSSFAPEDS-
-------------APGTSGRLFPGDPGP-EGARPPRAAPGRRAKRGPR------------
------RQSLS------ARGEDGERLY--SSMSRALLRRLWKGDASARMLHPRLQKAMGA
YLRANKHGVRFR--GRR-ASGR-SRTELLCALRGRVQVRTLDGTEPPFSALGWR-ALVPP
VPLS--RLLPR---RLRTCAVVTSAGAILNSSLGEEIDSHDAVLRFNSAPTRGYEKDVGN
KTTVRIINSQILTNPSYHFMDSALYKDVILVAWDPAPYSANLNLRYKKPDYNLFTPYVQH
RQRNPNQPFYILHPKFIWQLWDIIQENTKEKIQPNPPSSGFIGILLMMNLCGEVHVYEYV
PSVRQTDLCHYHEPYHDAACTLGAYHPLLYEKLLVQRLNVGTHGDLHRKGKVVLPGLQAV
RCPPGA---------
>Mmul_ST6Gal2
----------------------MKPHLKQWRQGMLFGIFAWGLLFLLIFIYFTDSNPAEP
-VPSSLS-------FLETRRLLPVQGKQR-------------AIMGAAHEPSPPGGLDAR
QGLSRAHPASSFHAGPGDPQKWAQSQHGFEHE-----EFFSSQV--GRKSQSAFYPEDDD
YFFAAGQPGWHSHTQGTLGFPSPREPGPRDGAFPAAQVQRRRVKKRHR------------
------RQRRS---HVLEEGDDGDRLY--SSMSRAFLYRLWKGNVSSKMLNPRLQKAMKD
YLTANKHGVRFR--GKR-ETGL-SRAQLLCQLRSRAPVRTLDGTEAPFSALGWR-RLVPA
VPLS--QLHPR---GLRSCAVVMSAGSILNSSLGEEIDSHDAVLRFNSAPTRGYEKDVGN
KTTVRIINSQILTNPSHHFIDSSLYKDVILVAWDPAPYSANLNLWYKKPDYNLFTPYIQH
RQRNPNQPFYILHPKFIWQLWDIIQENTKEKIQPNPPSSGFIGILIMMSMCREVHVYEYI
PSVRQTELCHYHELYYDAACTLGAYHPLLYEKLLVQRLNTGTQGDLHRKGKVVLPGFQTV
HCPAPSPVIPHS---
>Tnig_ST6Gal2
----------------------MKSCTRQWRRLVLAGMLAWGLLFLFLLSYFLDTRVN-E
PLTSAGSLLSQ---LPDTRRLASIQASQV-QHS----SPDLAATLTATYTRDEPGL----
----PATQEFSPETSR----APFAVYGGPEEAP---QDYLTPQS---LAAWSSFGTENIG
S---QSDPG-VQSRER-----TSQDHGSVTRYRGGEEVEEEEEEEEEGEVGQENEDGDMA
NGIRTTAGRKPG-----GDSSDLENYY--FSKSISVVQRLWQGRVSAGMLSPRLQRAMKD
YVTANKHHVSYR--GRR-SPGQ-TAKELLCELQAQARLQTVDGTEPPFSSLGWA-RLVPA
LPLDQLHQQPARGS-FNSCAVVTSAGAILRSGLGKEIDSHDAVLRFNAAPTEGYERDVGN
KTTIRIINSQILANPNHRFNSSSLYKDVVLVAWDPAPYTVDLHQWFASPDYNLFGPYVEH
RRPHPEQPFYILHPSYVWRLWNVIQGNTQENIQPNPPSSGFIGILLMMTLCEQVHVYEYI
PSVRQSDLCHYHERYYDAACTLGAYHPLLYEKSLIQRINTGPESDLRKKGRVTLPGFSAV
NCDI-----------
>Acar_ST6Gal2
----------------------MKPNLKQWKQVMLFGIFAWGLLFLVIFIYFTDSNTADA
-VPSSFS-------YIETKRLLPIQGKQR-------------AIMGAIHDPSFSESAVGN
EVLLSDDVLDSFKSGTGSIKKWTDLEDDFESEE----EFFPSQL--RRKSKSAFYQRDSD
YLFAAGQPQSQSSVQQIVKLISAEGEDQTRKAFQNNWLSQRKIKRRHT------------
------RHRRS---QMFEESDEWDGIY--STMSKSFLYRLWKGDVSSKMLNPRLQKAMKD
YLNTNKHGVRFR--GKR-NSKL-TSEQLFCELKARVNIRTIDGKEAPFSILGWE-KHVPQ
ISLN--KLYPN---GFGSCAVVMSAGAILNSSLGKEIDSHEAVLRFNSAPTRGYEKDVGN
KTTMRIINSQILTNPNHHFIENSLYKDVILVAWDPAPYSANLNVWFKKPDYNLFTPYVQH
RRKNPNQPFYILHPKFIWQLWDIIQENTKEEIQPNPPSSGFIGILIMMSMCHEVHVYEYI
PSVRQTDLCHYHELYYDAACTLGAYHPLLYEKLLVQRMNKGFQDDLYRKGKVILPGFRSV
KCPGHNRFPYT----
>Tgut_ST6Gal2
----------------------MKPNLKQWKQLMLFGIFAWGLLFLVIFIYFTDSNTAEP
-VPSSFS-------YIETKRLLPLQGKQR-------------VIMGAIHDPSFSETIDGN
EVLLNEDLLGTFKSGTVNSKKWTVLEEAFRNED----EFFPPQL--GRNSKSAFYQVNDD
YLFAAGQPQSH--LQEIEKFISADINNPKGNILQNNWSHQRRTRRRST------------
------KHRRS---QMFDESDDWDGLY--STMSKSFLYKLWKGDVSSKMLNPRLQKAMKD
YLSTNKHGVRFK--GKR-NSKL-TGDQLFCELKERVNVKTIDGKEAPFSTLGWE-KHVPQ
IPLV--KLYTH---GFGSCAVVMSAGAILNSSLGDEIDSHDAVLRFNSAPTRGYEKDVGN
KTTMRIINSQILTNPNHHFVDSSLYKDVILVAWDPAPYSANLSQWYKKPDYNLFTPYVQH
RRKNPTQPFYILHPKFIWQLWDIIQENTKEKIQPNPPSSGFIGSLIMMSMCNEVHVYEYI
PSVRQTDLCHYHELYYDAACTLGAYHPLLYEKLLVQRMNKGLQDDLYRKGKVILPGFKAI
KCPKRNNFPHL----
>Mdom_ST6Gal2
----------------------MKPNLKQWKQLMLFGIFAWGLLFLVIFIYFTDSNTVEP
-VPSSFS-------FVETKRILPIQGKQR-------------SIMGAMHDPSFSATIDGN
EVLLNGDLFDSFNSGPNSLQKWTEMKDGLENE-----EFFPSQM--RKKSPRIFYQGEDD
YFFTMGQPMSHSHGQQMVKFITLSEEDQKGGIRHN----QKRIISR--------------
-------HRRS---QLP---HNWDRLY--STMSKSFLYRLWKGNVSSKMLNPRLQKAMKD
YLTTNKHRVRFR--GKR-NSKL-TREQLLCELRDRVSVRTLDGKEAPFSDLGWE-KHVPQ
EPLS--KLYPQ---GLGSCAVVMSAGAILNSSLGEEIDSHDAVLRFNSAPTHGYERDVGN
KTTMRIINSQILTNPNHHFIDSSLYKDVILVAWDPAPYSANLHMWYKKPDYNLFTPYIQH
RRKNPNQPFYILHPKFIWQLWDIIQENTKEKIQPNPPSSGFIGILIMMSLCKEVHVYEYI
PSVRQTDLCHYHELYYDAACTLGAYHPLLYEKLLVQRMNQGIRDDLYRKGKAILPGFRAI
SCPAPNHFPNP----
>Drer_ST6Gal2-r
------------MVVQDSRLLRVQHAMKQSAQVLLLALLVWMLLALLVFTYFADFQLN--
---------------QNPKTVSSFNSESS-------------------------------
---RSTYSQHSGR-------------------------AIKSSS---NARWGVFAERRGP
Y---QRRQVFLPPHAK------EYFYFRKFDRRGLGVKKSKIIPPED-------------
-------NDRDD--------------Y--FSNPWSVVRGLWKGHVSSRMLSAKLQRAMKD
HVHTNKHRVLYK--GQR-KLNK-SKQELLCQMKEQAQIRTLNGTEQPFADLGFK-QLVSP
-PLQQKYR---------TCAVVASAGAILNSSLGHEIDSHDAVLRFNAAPSEGYERDVGS
KTTIRIMNSQILANPKYRFGSSLLYKNITLVAWDPAPYNIDLHKWFLHPDYDLFTPYITH
RKAFPEQPFYILHPSFIWKLWDIVQSITEENIQPNPPSSGFIGIILMMNLCEKIHVYEYI
PSMRQTDLCHYHELQYDMACTFGAYHPLLYEKLLVKRMSTASEEDLRKKGKVTLPGFSKI
KCLL-----------
>Ocun_ST6Gal2
```

```
----------------------MKPHLKQWRQRMLFGIFAWGLLFSVVFIYFTDSNPAEP
-VPSSFS-------FLETQGLLPVQGKQR-------------AIMGALHEPSSPGGADAS
RALPGAQVAGAFHSGPGDPQQWAQVPARFEDED----EFFASQV--GRKSQSAFYPEDYD
SLFAAARPGWPSHTQGTLGLPSPGAPGQ----------SPHREKRRHR------------
------RQRRSR--APLDEGEDGDRLA--ASMSRAFLYRLWKGNVSSKMLNPRLQKAMRD
YLAANKHGVRFR--GPR-AARR-SRAQLLCELRRRAQVRTLNGTEAPFAALGWR-RLVPA
VPLS--QLHPR---GLRTCAVVMSAGAILNSSLGEEIDSHDAVLRFNSAPTRGYEKDVGN
RTTMRIINSQILTNPNHHFVDSSLYKDVILVAWDPAPYSANLNLWYKKPDYNLFTPYIQH
RQRNPNQPFYILHPKFIWQLWDIIQENTKEKIQPNPPSSGFIGILIMMSLCREVHVYEYI
PSIRQTELCHYHELYYDAACTLGAYHPLLYEKLLVQRLNMGTQGDLHRKGKVVLPGFQAV
QCPAPNPADPHS---
>Oana_ST6Gal2
----------------------MKPNLKQWKQLMLFGIFAWGLLFLAIFIYFADSNPTAP
-VPSTF--------YVEPRRLLPVQGKQR-------------AIMGAVPEPAFPEGEARS
-----ETRCESLLCADLPDSLWPEPEDAGDIEE----EFFSPQVQRGRESPRAHDPALGD
SSFTADRPGPRGLARRGASLVSAGE----QGLFRSGRVPRKRIKKRRS------------
------KRGRS---RPPEEADEWERLYS-SAMSRSFLYRLWKGNVSSRMLNPRLQKAMKD
YLSINKHGVRFQ--GPR-NGRL-TGEQLLCQLRDRVHVRTLDGKEAPFSALGWD-KHVPR
TPLA--KLYPR---GFGSCAVVMSAGAILNSSLGAEIDAHDAVLRFNSAPTRGYEKDVGN
KTSLRIINSQILTNPSHHFIDNSLYKDVILIAWDPAPYSANLNVWYKKPDYNLFTPYVQH
RRRYPSQPFYILHPKFIWQLWDIIQENTREKIQPNPPSSGFIGILIMMSMCREVHVYEYI
PSVRQTDLCHYHELYYDAACTLGAYHPLLYEKLLVQRMNRGERDDLYRKGKVILPGFRAL
RCPAPD-HLPHS---
>Cpor_ST6Gal2
----------------------MKPHLKQWRQCMLFGIFAWGLLFLAIFIYFTDSNPAAA
-APSALS-------FLETRRLLPVQGKQR-------------AIMGAAHEP-APR-----
-----VHPGGRAHAVPADPQEVAGSQGGPRDED----EFFTSQV--GRRSQSAFYPEDYD
YFFLGSEPGRRGRALGVLGLASPGQLQRKTR----------------G------------
------RSSLS------PRGLDGDRLA--APLPGVLLRRLWTGEASARMLSPRLQKAMRD
YVRANKHGVRYR---------------LSCCARSRLSPRS-----------GGR-SLVPA
TALS--QLHP----GLRSCAVVMSAGAILNSSLGAEIDAHDAVLRFNSAPTRGYEKDVGN
KTTIRIINSQILANPGYHFIDSTLYKDVILVAWDPAPYSSNLYLWYKKPDYNLFTPYVQH
RQRHPAQPFYILHPKFIWQLWDIIQENTKEKIQPNPPSSGFIGIVIMMSMCDEVHVYEYI
PSVRQTELCHYHELYYDSACTLGAYHPLLFEKLLVQRLNMGSQADLYRKGKVVLPGFRAA
QCPEPGPGDARS---
>Mmur_ST6Gal2
----------------------MKPHLKQWRQRMLFGIFAWGLFFLVIFIYFTDSNPAEP
-APSSFS-------FLETRRLLPVQGKQR-------------VIMGAMHEPASPGSMDAD
PALPAGRPAGPFHVGPGDLQQWAQAQDDFENE-----DFFSSQV--ERKSQNAFYAEDDA
YFFAAGQPGWHSHTQGTLGFSSPGEPGQKQRALPAGQVQSVRVKDRHR------------
------RQRRT---HVLEVDDESR-----PFMSRALLYQLWKGNVSSKMLSPRLQKAMKA
YLTANKHGVRFR--GRR-AAAL-SRAELLCRLRSRVRVRTLDGTEAPFSALGWQ-RLVPA
VPLS--QLHPR---GLRSCAVVMSAGAILNSSLGEEIDSHDAVLRFNSAPTRGYEKDVGN
KTTMRIINSQILTNPNHHFIDSSLYKDVILVAWDPAPYSSNLNLWYKKPDYNLFTPYIQH
RQKNPNQPFYILHPKFIWQLWDIIQENTKEKIQPNPPSSGFIGILIMMSMCREVHVYEYI
PSIRQTELCHYHELYYDAACTLGAYHPLLYEKLLVQRLNTGSQGDLHRKGKVVLPGFRAV
RCPAPNSAGPHS---
>Pabe_ST6Gal2
----------------------MKPHLKQWRQRMLFGIFAWGLLFLLIFIYFTDSNPAEP
-VPSSLS-------FLETRRLLPVQGKQR-------------AIMGAAHEPSPPGGLDAR
QALPRVHPAGSFHAGPGDLQKWAPSQDGFEQK-----EFFSSQV--GRKSQSAFYPEDDD
YFFAAGQPGWHSHTQGTLGFPFPGEPGPREGAFPAAQVQRRRLKKRHR------------
------RQRRS---HVLEEGDDGDRLY--SSMSRAFLYRLWKGNVSSKMLNPRLQKAMKD
YLTANKHGVHFR--GKR-EAGL-SRAQLLCQLRSRARVRTLDGTEAPFSALGWR-RLVPA
VPLS--QLHPR---GLRSCAVVMSAGAILNSSLGEEIDSHDAVLRFNSAPTRGYEKDVGN
KTTVRIINSQILTNPSHHFIDSSLYKDVILVAWDPAPYSANLNLWYKKPDYNLFTPYIQH
RQRNPNQPFYILHPKFIWQLWDIIQENTKEKIQPNPPSSGFIGILIMMSMCREVHVYEYI
PSVRQTELCHYHELYYDAACTLGAYHPLLYEKLLVQRLNTGTQGDLHRKGKVVLPGFQAV
HCPAPSPVIPHS---
>Ggor_ST6Gal2
----------------------MKPHLKQWXQRMLFGIFAWGLLFLLIFIYFTDSNPAEP
-VPSSLS-------FLETRRLLPVQGKQR-------------AIMGAAHEPSPPGGLDAR
QALPRAXPAGSFHAGPGDLQKWAQSQDGFEHK-----EFFSSQV--GRKSQSAFYPEDDD
YFFAAGQPGWHSHTQGTLGFPSPGEPGPREGAFPAAQVQRRRVKKRHR------------
------RQRRS---HVLEEGDDGDRLY--SSMSRAFLYRLWKGNVSSKMLNPRLQKAMKD
YLTANKHGVRFR--GKR-EAGL-SRAQLLCQLRSRARVRTLDGTEAPFSALGWR-RLVPA
VPLS--QLHPR---GLRSCAVVMSAGAILNSSLGEEIDSHDAVLRFNSAPTRGYEKDVGN
KTTVRIINSQILTNPSHHFIDSSLYKDVILVAWDPAPYSANLNLWYKKPDYNLFTPYIQH
RQRNPNQPFYILHPKFIWQLWDIIQENTKEKIQPNPPSSGFIGILIMMSMCREVHVYEYI
PSVRQTELCHYHELYYDAACTLGAYHPLLYEKLLVQRLNTGTQGDLHRKGKVVLPGFQAV
HCPAPSPVIPHS---
>Stro_ST6Gal2
----------------------MKPNLKQWKQFMLFGICAWGLLFLVIFVYFTDSNSVEP
-VPSAFS-------YVESKKHFPLQGKQR-------------AIMGAHQDQLFSYAIDDQ
D-LLKEGILDSFIVGPGSMKKMAGADNYFESEQ----EFIMSKK--TQKSTSNNHEDDDD
EIYLH---------KNIDSVSGKKAPAYGKRYYHDTQRQHKKIRRNMQ------------
------RKKQH----MIEDSYDWNGFS--SSMSKSFLQKLWKGNVSSKMLTPRLQKARRE
YLRANKLGVNFN--GKQNSRKL-NPQELLCVLKDRAQVKTLDGKDAPFSSLGWE-KYFPK
IALN--KLYPH---GFSTCAVVSSAGAILNSSLGAEIDSHDAVLRFNSAPTRNYEKDVGN
KTTLRIINSQILTNPNHHFTDSSLYKDVTLIAWDPSPYYADLHMWYHKPDYNLFPPYEKH
RKRNPDQPFYILHPKFTWELWKIIQENSNEKIQPNPPSSGFIGILIMMSMCRTVHVYEYI
PSYRQTDLCHYHEQYYDAACTLGAYHPLLYEKMLIQRINQGTEDNLLRKGKVILPGFSSI
HCPIKDHIT------
>Olat_ST6Gal2
----------------------MRFSMRQWRKLVLAAILAWALLFLGLLSYFLDNRVE-E
PLTPAGSLVSQ---HSDTRRLTSIQSSQQQQQPVPGLRSEQGLNSIRTSHGNQPEAG---
--VLSSSETPGNMVNHIPSSSPYQTYGSQEANH---QNDLDPQS---LAAWSSFGTQNVD
S---NFNIA-SQHRER-----ASQSIFSNN-------VEDEELPNEIS------------
----PLVERRAD-----ADN---------------VVQHMWRGTVSSGMLSPRLKRAMND
YINANKHHVSYQ--RHR-KVAR-SAKELLCQMKNQSQLRTVDGSEQPFSSLGWA-DFVPL
```

```
VPLQRWNKQRGGRS-FRTCAVVSSAGAILHSGLGKEIDSHDAVLRFNAAPTEGYEQDVGT
KTTIRIINSQILANPKHEFKTSSIYKNITLVAWDPAPYTLNLDEWFASPDYDLFGPYVEH
RKNHAEQLFYILHPSYLWQLWDLIQSNTQEKIQPNPPSSGFIGILTMMALCDKLHVYEYI
PSMRQTDLCHYHENYYDAACTLGAYHPLIYEKNLIRRINLGSEKDLLKKGRVTLPGFSTL
TCGA-----------
>Gacu_ST6Gal2
----------------------MKSSMRQWRRLVLLGMLAWVLLFLGLLSYFLDARVN-E
PLTSVGSLFSQ---HPDTRRLASIQASHQ-QHANLGPRPELASTLTTTHPGVEPET---R
PSASSATPQLSLDMSHSPKSAHYAVYGSHETSR---PDYLDPQS---LAAWSSFGTENVG
S---HSDPS-VQSRER---TSQYENHISTSRYRGDGEEEEEEEDDERD-------NVDEE
LRKRTPAGRRVG-----DDSADLEEYY--FSESGSVVQRLWRGRVSAGMLSPRLQRAMKD
YVSANKHRVSYT--GHR-RAAQ-SANELLCQMKEQARLRTVDGSEQPFSSLGWA-RLVPS
RPLERLHKRQDRSS-FKTCAVVTSAGAILRSGLGKEIDAHDAVLRFNAAPTEGYERDVGS
KTTIRIINSQILANPKHRFNTSSIYKNVTLVAWDPAPYTVDLHKWYAGPDYNLFGPYIEH
RKLHPDQPFYILHPSYVWRLWDVIQGNTQENIQPNPPSSGFIGILLMMALCEQLHVYEYI
PSIRQTDLCHYHERYYDAACTLGAYHPLLYEKSLIQRIHTGPKNDLRWKGRVTLPGLSTV
NCDI-----------
```

Data S4. SDP prediction results are shown below; MSA used is the one present in supplementary data C obtained with 101 ST6Gal sequences (48 ST6Gal I and 53 ST6Gal II). The header contains information about the parameters used in the server, followed by a table with the SDPs predictions sorted by scores. A detailed explanation of the output format can be found at Help section on the SPEER web server (http://www.hpppi.iicb.res.in/ss/help.html).

```
Number of columns in the alignment = 615
Number of sequences in the alignment = 101
Number of supplied subfamilies = 2
Subfamily 1 consists of 48 sequences
Subfamily 2 consists of 53 sequences
 Gap percentage for each columns selected = 20%
SPEER-SERVER scoring term weights:-
Relative entropy term weight = 1
PC property distance term weight = 1
Evolutionary rate term weight = 0
SPEER-SERVER predicted scores for columns containing 20% gaps are shown below.
Columns are sorted based on SPEER-SERVER Score
         SPEER-SERVER Result
########################################################################
# Column    %ID      Score    Z-score       P-value         TYPE #
########################################################################
     307         47.7     5.892    3.104          0.001          TypeII
     426         48.6     5.395    2.842          0.002          TypeII
     539         49.7     5.328    2.807          0.003          TypeII
     540         47.6     5.261    2.771          0.003          TypeII
     553         49.7     5.120    2.697          0.004          TypeII
     586         49.7     4.946    2.605          0.005          TypeII
     448         38.9     4.803    2.530          0.006          TypeII
     576         47.6     4.692    2.472          0.007          TypeII
     387         49.5     4.655    2.452          0.007          TypeII
     280         46.8     4.532    2.387          0.008          TypeII
     522         48.8     4.453    2.346          0.010          TypeII
     358         47.8     4.307    2.269          0.012          TypeII
     555         47.9     4.262    2.245          0.012          TypeII
     480         48.5     4.190    2.207          0.014          TypeII
     41          33.7     3.966    2.089          0.018          TypeI
     23          35.9     3.877    2.042          0.021          TypeI
     389         39.9     3.850    2.028          0.021          TypeII
     297         39.6     3.834    2.020          0.022          TypeII
     55          31.6     3.780    1.991          0.023          TypeI
     552         49.0     3.758    1.979          0.024          TypeII
     24          32.6     3.636    1.915          0.028          TypeI
     54          31.4     3.515    1.851          0.032          TypeI
     431         38.9     3.514    1.851          0.032          TypeII
     283         37.3     3.422    1.803          0.036          TypeI
     304         28.3     3.296    1.736          0.041          TypeI
     543         19.7     3.168    1.669          0.048          MC
     432         36.5     3.123    1.645          0.050          TypeI
     388         42.7     3.118    1.643          0.050          TypeII
     435         37.0     3.097    1.631          0.051          TypeI
     473         39.2     3.087    1.626          0.052          TypeI
     573         33.4     3.061    1.612          0.053          TypeI
     446         37.1     3.033    1.598          0.055          TypeI
     282         27.4     3.030    1.596          0.055          MC
     51          34.6     3.027    1.594          0.055          TypeI
     300         36.5     2.933    1.545          0.061          TypeI
     476         34.5     2.845    1.499          0.067          TypeI
     478         18.1     2.841    1.497          0.067          MC
     352         35.6     2.743    1.445          0.074          TypeI
     383         35.2     2.659    1.401          0.081          TypeI
     440         29.4     2.653    1.398          0.081          TypeI
     80          28.8     2.590    1.364          0.086          TypeI
     339         21.8     2.559    1.348          0.089          MC
     29          33.7     2.538    1.337          0.091          TypeI
     497         20.2     2.537    1.336          0.091          MC
     521         40.7     2.534    1.335          0.091          TypeII
     589         34.1     2.519    1.327          0.092          TypeI
```

45	32.1	2.331	1.228	0.110	TypeI
583	30.9	2.289	1.206	0.114	TypeI
52	34.1	2.270	1.196	0.116	TypeI
588	25.5	2.179	1.148	0.125	MC
434	34.4	2.145	1.130	0.129	TypeI
56	17.5	2.048	1.079	0.140	
114	21.6	1.981	1.044	0.148	
285	26.7	1.939	1.021	0.154	MC
85	31.7	1.909	1.006	0.157	TypeI
26	20.4	1.814	0.956	0.170	MC
414	54.3	1.775	0.935	0.175	TypeII
317	31.7	1.738	0.915	0.180	
508	34.2	1.732	0.912	0.181	TypeI
298	31.8	1.676	0.883	0.189	TypeI
57	17.7	1.503	0.792	0.214	
525	37.8	1.465	0.772	0.220	TypeI
342	31.2	1.406	0.741	0.229	
50	25.0	1.380	0.727	0.234	MC
88	20.8	1.327	0.699	0.242	
111	21.8	1.300	0.685	0.247	
420	31.2	1.268	0.668	0.252	TypeI
509	15.2	1.240	0.653	0.257	MC
83	23.8	1.199	0.632	0.264	MC
273	32.9	1.143	0.602	0.274	
361	11.6	1.100	0.579	0.281	
77	18.6	1.085	0.572	0.284	
82	19.2	1.075	0.566	0.286	MC
461	41.2	1.010	0.532	0.297	TypeI
345	34.7	0.996	0.525	0.300	
48	22.6	0.989	0.521	0.301	MC
43	38.7	0.968	0.510	0.305	TypeI
362	28.9	0.931	0.490	0.312	TypeI
545	50.0	0.888	0.468	0.320	TypeI
279	22.0	0.769	0.405	0.343	MC
58	19.0	0.756	0.398	0.345	
496	32.2	0.744	0.392	0.348	MC
172	23.9	0.728	0.383	0.351	
447	19.8	0.667	0.351	0.363	MC
86	26.2	0.642	0.338	0.368	MC
479	16.4	0.590	0.311	0.378	MC
357	20.0	0.557	0.293	0.385	MC
408	31.4	0.544	0.287	0.387	MC
371	10.3	0.542	0.285	0.388	
291	20.4	0.533	0.281	0.389	MC
554	24.9	0.519	0.274	0.392	MC
127	11.7	0.509	0.268	0.394	
580	19.9	0.502	0.265	0.396	MC
322	16.1	0.443	0.233	0.408	
356	12.8	0.442	0.233	0.408	MC
320	11.4	0.410	0.216	0.415	
343	36.6	0.394	0.208	0.418	
168	15.4	0.357	0.188	0.425	
462	59.0	0.344	0.181	0.428	TypeI
511	17.1	0.306	0.161	0.436	MC
126	10.6	0.305	0.161	0.436	
562	57.1	0.298	0.157	0.438	TypeI
171	12.1	0.294	0.155	0.438	
141	12.0	0.283	0.149	0.441	
139	9.7	0.267	0.141	0.444	
336	50.7	0.251	0.132	0.447	TypeI
278	15.7	0.225	0.118	0.453	
146	13.7	0.219	0.115	0.454	
44	26.4	0.208	0.110	0.456	MC
284	23.2	0.204	0.108	0.457	MC
459	30.6	0.201	0.106	0.458	MC
275	22.9	0.197	0.104	0.459	
272	17.5	0.184	0.097	0.461	
587	41.5	0.158	0.083	0.467	TypeI
577	56.6	0.153	0.080	0.468	TypeI
25	30.0	0.150	0.079	0.469	MC
441	18.5	0.144	0.076	0.470	MC
351	48.5	0.136	0.072	0.471	
412	17.0	0.136	0.071	0.472	MC
467	19.7	0.085	0.045	0.482	MC
170	19.7	0.079	0.042	0.483	
415	59.9	0.060	0.032	0.487	TypeI
593	54.1	0.059	0.031	0.488	TypeI
289	53.3	0.033	0.017	0.493	TypeI
316	14.1	0.032	0.017	0.493	
140	13.4	0.013	0.007	0.497	
390	26.5	0.008	0.004	0.498	MC
364	15.0	-0.020	-0.011	0.504	MC
436	13.7	-0.040	-0.021	0.508	MC
178	13.7	-0.059	-0.031	0.512	
176	14.2	-0.074	-0.039	0.515	
271	22.4	-0.080	-0.042	0.517	
128	13.9	-0.080	-0.042	0.517	
442	51.1	-0.088	-0.046	0.519	TypeI
148	12.7	-0.089	-0.047	0.519	
143	10.6	-0.155	-0.082	0.533	

556	43.9	-0.164	-0.086	0.534	TypeI
131	17.0	-0.168	-0.088	0.535	
142	23.1	-0.174	-0.092	0.536	
460	32.6	-0.180	-0.095	0.538	MC
376	27.4	-0.182	-0.096	0.538	
42	18.3	-0.185	-0.098	0.539	MC
49	32.9	-0.207	-0.109	0.543	MC
337	19.8	-0.215	-0.113	0.545	MC
136	10.8	-0.216	-0.114	0.545	
584	17.5	-0.218	-0.115	0.546	MC
84	20.9	-0.250	-0.132	0.552	MC
475	21.6	-0.263	-0.139	0.555	MC
463	17.3	-0.313	-0.165	0.566	MC
169	15.4	-0.320	-0.169	0.567	
392	24.1	-0.327	-0.172	0.568	MC
340	53.3	-0.339	-0.178	0.571	TypeI
575	64.5	-0.342	-0.180	0.572	TypeI
452	53.4	-0.367	-0.193	0.577	TypeI
493	53.8	-0.374	-0.197	0.578	TypeI
147	11.3	-0.378	-0.199	0.579	
451	57.0	-0.380	-0.200	0.579	TypeI
112	17.7	-0.391	-0.206	0.582	
483	25.8	-0.401	-0.211	0.584	MC
79	34.1	-0.408	-0.215	0.585	MC
331	31.7	-0.438	-0.231	0.591	MC
302	36.3	-0.440	-0.232	0.592	MC
506	39.7	-0.449	-0.236	0.593	MC
344	18.2	-0.452	-0.238	0.594	
321	15.0	-0.458	-0.241	0.595	
138	14.7	-0.474	-0.250	0.599	
115	21.3	-0.477	-0.251	0.599	
495	28.2	-0.480	-0.253	0.600	MC
548	53.8	-0.492	-0.259	0.602	TypeI
133	9.4	-0.493	-0.260	0.603	
416	26.9	-0.498	-0.262	0.603	MC
535	56.7	-0.498	-0.262	0.603	TypeI
310	18.7	-0.518	-0.273	0.608	MC
110	11.0	-0.523	-0.275	0.609	
530	55.7	-0.551	-0.290	0.614	TypeI
346	15.4	-0.565	-0.297	0.617	
144	11.8	-0.573	-0.302	0.619	
175	15.3	-0.584	-0.307	0.621	
308	30.8	-0.609	-0.321	0.626	MC
464	24.5	-0.609	-0.321	0.626	
532	29.5	-0.610	-0.321	0.626	MC
87	33.4	-0.613	-0.323	0.627	MC
274	11.4	-0.629	-0.331	0.630	
378	32.3	-0.635	-0.335	0.631	MC
472	49.2	-0.638	-0.336	0.632	TypeI
276	18.3	-0.640	-0.337	0.632	MC
277	28.9	-0.664	-0.350	0.637	MC
132	14.2	-0.684	-0.360	0.641	
27	36.4	-0.692	-0.364	0.642	MC
349	45.4	-0.697	-0.367	0.643	
363	61.1	-0.702	-0.370	0.644	TypeI
28	43.9	-0.710	-0.374	0.646	MC
427	47.3	-0.712	-0.375	0.646	TypeI
113	20.1	-0.724	-0.381	0.648	
504	62.2	-0.726	-0.383	0.649	TypeI
533	28.9	-0.732	-0.386	0.650	MC
437	43.3	-0.751	-0.396	0.654	TypeI
395	21.7	-0.785	-0.413	0.660	MC
326	16.1	-0.786	-0.414	0.661	
312	23.4	-0.791	-0.417	0.662	MC
296	57.7	-0.793	-0.418	0.662	TypeI
129	27.6	-0.811	-0.427	0.665	
334	21.3	-0.818	-0.431	0.667	MC
335	26.3	-0.826	-0.435	0.668	MC
53	24.9	-0.829	-0.437	0.669	MC
78	45.7	-0.839	-0.442	0.671	
529	35.8	-0.844	-0.445	0.672	MC
145	21.3	-0.856	-0.451	0.674	
524	64.8	-0.877	-0.462	0.678	TypeI
503	39.3	-0.884	-0.466	0.679	MC
514	67.5	-0.893	-0.471	0.681	TypeI
130	14.0	-0.908	-0.478	0.684	
499	49.7	-0.930	-0.490	0.688	MC
137	15.9	-0.932	-0.491	0.688	
484	47.0	-0.938	-0.494	0.689	TypeI
469	69.1	-0.955	-0.503	0.692	TypeII
299	44.1	-0.976	-0.514	0.696	MC
46	51.9	-0.983	-0.518	0.698	TypeI
327	26.1	-1.008	-0.531	0.702	
338	53.8	-1.026	-0.540	0.705	TypeI
570	62.4	-1.028	-0.542	0.706	TypeI
550	71.7	-1.073	-0.565	0.714	TypeII
303	23.4	-1.094	-0.576	0.718	MC
332	66.6	-1.113	-0.586	0.721	TypeI
558	49.8	-1.114	-0.587	0.721	TypeI
287	57.7	-1.141	-0.601	0.726	TypeI

456	65.2	-1.146	-0.603	0.727	TypeI
598	26.4	-1.158	-0.610	0.729	MC
288	24.8	-1.159	-0.611	0.729	
502	63.7	-1.189	-0.627	0.735	TypeI
360	13.1	-1.195	-0.629	0.735	MC
468	27.9	-1.196	-0.630	0.736	MC
582	24.3	-1.198	-0.631	0.736	MC
458	71.4	-1.269	-0.668	0.748	TypeII
486	15.4	-1.311	-0.691	0.755	MC
444	69.5	-1.332	-0.702	0.759	TypeI
270	19.8	-1.335	-0.703	0.759	
457	70.8	-1.348	-0.710	0.761	TypeI
599	19.8	-1.406	-0.740	0.770	MC
319	22.3	-1.406	-0.741	0.771	
536	68.8	-1.409	-0.742	0.771	TypeI
399	40.2	-1.411	-0.743	0.771	MC
350	21.1	-1.418	-0.747	0.772	
134	11.3	-1.435	-0.756	0.775	
424	44.6	-1.452	-0.765	0.778	MC
600	32.0	-1.453	-0.766	0.778	MC
47	25.9	-1.461	-0.770	0.779	MC
601	21.4	-1.471	-0.775	0.781	MC
353	96.1	-1.479	-0.779	0.782	TypeII
301	98.0	-1.480	-0.779	0.782	TypeII
293	98.0	-1.485	-0.782	0.783	TypeII
359	98.0	-1.486	-0.783	0.783	TypeII
581	98.0	-1.486	-0.783	0.783	TypeII
347	98.0	-1.525	-0.804	0.789	
507	67.6	-1.527	-0.804	0.789	TypeI
579	96.1	-1.538	-0.810	0.791	TypeII
485	98.0	-1.549	-0.816	0.793	TypeII
292	54.4	-1.564	-0.824	0.795	TypeI
578	34.2	-1.577	-0.831	0.797	MC
413	64.7	-1.580	-0.832	0.797	TypeI
295	60.3	-1.596	-0.841	0.800	TypeI
329	55.2	-1.601	-0.843	0.800	MC
465	98.0	-1.603	-0.844	0.801	TypeII
423	98.0	-1.614	-0.850	0.802	TypeII
135	32.9	-1.628	-0.858	0.804	
449	40.1	-1.628	-0.858	0.804	MC
177	22.9	-1.633	-0.860	0.805	
534	90.4	-1.676	-0.883	0.811	TypeII
328	90.4	-1.744	-0.918	0.821	TypeII
592	59.6	-1.754	-0.924	0.822	TypeI
333	48.2	-1.776	-0.936	0.825	MC
377	29.2	-1.812	-0.954	0.830	
354	25.2	-1.818	-0.958	0.831	MC
315	69.8	-1.824	-0.961	0.832	
482	32.1	-1.863	-0.982	0.837	MC
348	96.1	-1.864	-0.982	0.837	
311	40.3	-1.891	-0.996	0.840	
421	94.1	-1.917	-1.010	0.844	TypeII
89	36.3	-1.939	-1.021	0.846	
411	79.7	-1.966	-1.036	0.850	TypeII
574	83.4	-1.967	-1.036	0.850	TypeII
341	33.3	-1.989	-1.048	0.853	
546	85.3	-2.042	-1.076	0.859	TypeII
500	81.4	-2.060	-1.085	0.861	TypeII
433	47.6	-2.069	-1.090	0.862	MC
443	71.2	-2.108	-1.110	0.867	TypeI
526	44.8	-2.137	-1.126	0.870	MC
591	50.5	-2.139	-1.127	0.870	MC
547	54.0	-2.155	-1.135	0.872	MC
294	88.7	-2.223	-1.171	0.879	TypeII
401	88.7	-2.244	-1.182	0.881	TypeII
404	83.1	-2.273	-1.197	0.884	TypeII
466	76.9	-2.276	-1.199	0.885	TypeII
597	83.6	-2.306	-1.215	0.888	TypeII
585	92.2	-2.307	-1.215	0.888	TypeII
286	92.3	-2.388	-1.258	0.896	TypeII
569	94.1	-2.388	-1.258	0.896	TypeII
488	90.4	-2.416	-1.273	0.898	TypeII
477	92.3	-2.423	-1.276	0.899	TypeII
595	90.4	-2.445	-1.288	0.901	TypeII
491	94.1	-2.530	-1.333	0.909	TypeII
510	96.1	-2.595	-1.367	0.914	TypeII
474	94.1	-2.605	-1.372	0.915	TypeII
418	98.0	-2.641	-1.391	0.918	TypeII
528	98.0	-2.647	-1.395	0.918	TypeII
397	98.0	-2.651	-1.396	0.919	TypeII
393	98.0	-2.652	-1.397	0.919	TypeII
602	98.0	-2.662	-1.402	0.920	
566	98.0	-2.667	-1.405	0.920	TypeII
492	98.0	-2.670	-1.407	0.920	TypeII
417	96.1	-2.671	-1.407	0.920	TypeII
490	96.1	-2.681	-1.412	0.921	TypeII
450	98.0	-2.700	-1.422	0.923	TypeII
594	98.0	-2.705	-1.425	0.923	TypeII
396	98.0	-2.732	-1.439	0.925	TypeII
513	98.0	-2.743	-1.445	0.926	TypeII

512	98.0	-2.744	-1.445	0.926	TypeII
290	98.0	-2.744	-1.446	0.926	TypeII
515	98.0	-2.746	-1.446	0.926	TypeII
